# Acknowledgment to Reviewers of *International Journal of Environmental Research and Public Health* in 2021

**DOI:** 10.3390/ijerph19031895

**Published:** 2022-02-08

**Authors:** IJERPH Editorial Office

**Affiliations:** MDPI AG, St. Alban-Anlage 66, 4052 Basel, Switzerland

Rigorous peer-reviews are the basis of high-quality academic publishing. Thanks to the great efforts of our reviewers, *International Journal of Environmental Research and Public Health* was able to maintain its standards for the high quality of its published papers. Thanks to the contribution of our reviewers, in 2021, the median time to first decision was 20 days and the median time to publication was 42 days. The editors would like to extend their gratitude and recognition to the following reviewers for their precious time and dedication, regardless of whether the papers they reviewed were finally published:
A. Belén del RíoJustyna MazurekAalap ChokshiJustyna MiszczykAapo HuovilaJustyna MrózAarno DietzJustyna Opydo-SzymaczekAaron BachJustyna PawlakAaron D. SciasciaJustyna RójAaron DallmanJustyna RubaszekAaron LawsonJustyna WitkowskaAaron M. EakmanJustyna Zdunek-WielgołaskaAaron R. AshbrookJuta KroičaAbay AsfawJuvenal Rodríguez-ReséndizAbbas SmileyJuyeon OhAbbey ThomasJu-Yeon UhmAbdelbary ElhissiJuyeun LeeAbdelghani BenyoucefJu-Yi ChenAbdelhadi BouchikhiJuzar AliAbdelhaleem KhaderJyh-Fei LiaoAbdelhamid KerkadiJyh-Jeng WuAbdellah ChehriJyh-Miin LinAbdellatif ElghaliJyotirmoy DasAbdolnasser SadeghkhaniJyu-Lin ChenAbdonas TamošiunasK. H. Katie ChanAbdou HachaniK. J. CerankowskiAbdul Majeed SalmasiK. Thomas LiawAbdul Mohin SajibKa Hei LuiAbdul Rashid AzizKa KimAbdul WaheedKa LinAbdulhameed Al-GhabkariKacper NijakowskiAbdulkadir AtalanKadri SeppaAbdullah AkpinarKaeko KameiAbdullah Al HamidKai ErenliAbdullah AlharthiKai HuangAbdullah AlsharefKai J. JonasAbdullah WahbehKai LiuAbdulrahman BasahelKai SchuhAbed AgbaryaKai Sing SunAbed Al Waheed HawilaKai W. MüllerAbedalrhman AlkhateebKai WillführAbee L. BoylesKai YangAbel Bauero EscribanoKai ZhangAbel LermaKai ZhaoAbel López DíezKaibao NieAbel TabladaKaija AppelqvistAbel Toledano-GonzálezKai-Jen ChuangAbhigya MookherjeeKai-Jun LuoAbhijeet SinghKailu WangAbhijit ChatterjeeKailun YangAbhijit GhoshKaine GriggAbhilash PerisettiKairi KõlvesAbhirup DasKais GadhoumiAbhishek RoyChowdhuryKaisa PihlainenAbia Akebe Luther KingKaitano DubeAbiel Aguilar-GonzálezKaitao LiAbigail EasterKai-Wen ChengAbigail MorrisKaiwen ManAbigail ReiterKaja AntlejAbigail SilvaKajal KhodamoradiAbigail StevelyKajetan GrodeckiAbimbola KolawoleKalaimani ElangoAbiodun Morakinyo AdeolaKalogiannakis MichailAbiodun Olagoke AdenijiKalpana RajaAbir NagataKalpita BanerjeeAbraham DibKalyana Chakravarthy PentapatiAbraham García-AliagaKamal MajiAbraham López VivancosKamaljeet SinghAbraham P. BuunkKam-Chau WuAbraham RudnickKamel FantazyAbraham Wall MedranoKamil BarańskiAbrahan MoraKamil GillAbreham Berta AneseyeeKamil JurczyszynAbu Reza Md Towfiqul IslamKamil KoszelaAcadia W. BuroKamil MichalikAchilleas BoutsiadisKamil PíchaAchmad SyafiuddinKamil SzewczakAchraf AmmarKamil ZaworskiAchraf CohenKamila Czepczor-BernatAda Fung Wai-TungKamila Litwic-KaminskaAda ScupolaKamila Pokorska-NiewiadaAdalgiza FornaroKamila RasovaAdam BenkwitzKamila ŘasováAdam BulleyKamila Rybczyńska-TkaczykAdam C. MillarKamol DeyAdam CiszkiewiczKamyar AsadipooyaAdam CohenKamyar HasanzadehAdam ColeKan SaitoAdam CzarneckiKanade ItoAdam DaragóKanae MureAdam FrankKanae TarunoAdam GordonKanako NoritakeAdam HegeKanani E. TitchenAdam JajtnerKandeepan KarthigesuAdam James Ross TallmanKang DaiwenAdam KalksteinKang Hao CheongAdam KeathKang SongAdam LewisKang WangAdam MetelskiKanghyeok YangAdam O. GoldsteinKangwei LiAdam OlivieriKanike Raghavendra KumarAdam PawlewiczKankoé SallahAdam ReevesKanokporn PinyopornpanishAdam RosińskiKantha DayaramAdam SulkowskiKanthee AnantapongAdam T. CarpenterKao-Chin ChenAdams BodomoKaori EndoAddo BoafoKaori KitagawaAdebowale OgunjirinKaoruko SeinoAdeboye AzeezKaoruko ShimizuAdekunle OkeKaowen Grace ChangAdel AltiKapil AedmaAdela AndryskovaKapil D. PatelAdela BadauKapo WongAdela GoleaKara P. WisemanAdela Magdalena CiobanuKara SkeltonAdelaida AvinoKaram Daljit SinghAdélaïde ChesnayKarel AllegaertAdele HoughtonKaren A. Vinall-CollierAdele SaterianoKaren CarlisleAdeleye Ayoade AdeniranKaren F. RossAdelle McArdleKaren FisherAdem ÖcalKaren Fowler-WattAdewuyi Ayodele AdeyinkaKaren GaleaAdeyinka AdebayoKaren HardeeAdhikarimayum Lakhikumar SharmaKaren JakubowskiAdil El MidaouiKaren KopciukAdilson MarguesKaren KormuthAdilson MarquesKaren L. HarpsterAdina BrahaKaren L. PrestegaardAdina FrumKaren LambAdina MaeirKaren LevyAdina SchneeweisKaren LillerAdina Turcu-StiolicaKaren LindsayAdis PuškaKaren Marie KeptnerAditya Rio PrabowoKaren McCollAdolfo RomeroKaren MorganAdoración Carmen Castro GraciaKaren S. ByrdAdoración Díaz LópezKaren SiedleckiAdrià ArboixKaren Walker-BoneAdriaan Albert MarkusKaren WebbAdrian Cantemir CalinKaren WillisAdrian CumminsKaren ZwiAdrián CurtoKari B. JensenAdrian Elmi-TeranderKari Bjerke Batt-RawdenAdrian Emil LazarescuKarien LabuschagneAdrián González-MarrónKarim BarigouAdrián Gutiérrez SerpaKarim ChamariAdrian KellerKarin ConinxAdrian ŁukowskiKarin EnskärAdrian OoKarin HugeliusAdrian OpreKarin MeissnerAdrian Pavel PugnaKarin OechsleAdrián Sánchez-MontalváKarin SkillAdrián Varela SanzKarin StanzelAdriana Alventosa BañosKarina AllenAdriana AubertKarine PanicoAdriana Burlea-SchiopoiuKarissa PeyerAdriana DornellesKarl AndriessenAdriana HenriquesKarl BayerAdriana HernándezKarl Erik LundAdriana KaplánováKarl Frederick Braekkan PayneAdriana LisKarl G. StonecipherAdriana Peña Pérez NegronKarl GoodkinAdriana PotraKarl HeleAdriana S. MiuKarl HusaAdriana SferraKarl Otfried Tfried SchwabAdriana Tiron TudorKarla A. RomeroAdriano De LeveranoKarla ArmentiAdriano FriganovicKarla De JesusAdriano M. PimentaKarla G. CedanoAdriano PiattelliKarla Paola García PelagioAdriano SantosKarl-Anton HillerAdriano SaquetKarlena Lara-OteroAdriano TagliabracciKarlo Malave-LlamasAdrie VerhoevenKarol A. MooreAdrien BreimanKarol DąbrowskiAdrienne AlayliKarol FlisikowskiAdrienne K. SalmKarol KonaszewskiAdrienne Vanessa LevayKarol KrólAely ParkKarol SteckiewiczAfia Zubair RajaKarol SzylukÁfrica Calvo-LluchKaroliina S. KaasalainenAfsane RiaziKarolina BrylÁgata AranhaKarolina Dudzic-GyurkovichAgata BalińskaKarolina Goździewska-HarłajczukAgata Carmela NicolosiKarolina HansenAgata DąbrowskaKarolina JablonskaAgata GabryelskaKarolina KosaAgata GawlakKarolina KossakowskaAgata KociaKarolina KotAgata M. NiewczasKarolina KowalczykAgata Markowska-SzczupakKarolina KowalskaAgata MarzecKarolina KrysinkaAgata Mesjasz-LechKarolina KrysinskaAgata MichalakKarolina ŁagowskaAgata MikolajczykKarolina NowakAgata Pawłat-ZawrzykrajKarolina OgrodnikAgata StanekKarolina OsowieckaAgata Szymańska-PulikowskaKarolina PituraAgata TrzcionkaKarolina PuławskaAgata WawrzyniakKarolina Skonieczna-ŻydeckaAgime GerbetiKarolina Szymaniec-MlickaAglaia StampoltzisKarolina TalarAglaja StirnKarolina TkaczAgnė SlapšinskaitėKarolina WieszczyckaAgnė UlytėKarolina WiszumirskaAgnes E. DoddsKarsten BeckerAgnès HagègeKarsten Elmose-ØsterlundAgnes JanovszkyKarsten PlotzAgnes Kilonzo-NthengeKartik AnandAgnes LacroixKashi Raj BhattaraiAgnes PeterfalviKashif ZiaAgnes SanthaKasia CampbellAgnese De MarioKasper SalinAgness TemboKassapa EllepolaAgnessa KozakKassiani TheodorakiAgneta Malmgren FängeKasthuri MaheshAgnieszka A. Bojarska-JunakKastytis SmigelskasAgnieszka Anna Olszewska-GuizzoKata CsekoAgnieszka BaranskaKatajun LindenbergAgnieszka BarczakKatariina Ala-RämiAgnieszka BartoszekKatariina KoistinenAgnieszka BemKatarina PrnjakAgnieszka BiałekKatarina RoguljAgnieszka BieńKatarína VilinováAgnieszka BieńkowskaKatarina VukojevicAgnieszka BitkowskaKatarina WijkAgnieszka BusKatarzyna Anna DylagAgnieszka Bzikowska-JuraKatarzyna BaczynskaAgnieszka Chlon-DominczakKatarzyna Beata CywkaAgnieszka CieślaKatarzyna BroczekAgnieszka CuprysKatarzyna ChebaAgnieszka Ćwirlej-SozańskaKatarzyna Chudy-LaskowskaAgnieszka CzerwińskaKatarzyna CzechAgnieszka DawidowiczKatarzyna DomaszewskaAgnieszka Dołhańczuk-ŚródkaKatarzyna Dubas-JakóbczykAgnieszka Drosdzol-CopKatarzyna DurniatAgnieszka DrozdzikKatarzyna DylewskaAgnieszka DudziakKatarzyna GoralczykAgnieszka FilipekKatarzyna GrocholewiczAgnieszka GawlikKatarzyna HojanAgnieszka GenowskaKatarzyna HolcmanAgnieszka GniadekKatarzyna IgnatowiczAgnieszka Gromkowska-MelosikKatarzyna JachAgnieszka J. SzczepekKatarzyna JandaAgnieszka JagodaKatarzyna KaczyńskaAgnieszka Jankowicz-SzymańskaKatarzyna KajdanekAgnieszka JankowskaKatarzyna KakarekoAgnieszka JastrzębskaKatarzyna Kocur-BeraAgnieszka JaszczakKatarzyna KodziszewskaAgnieszka JaworowskaKatarzyna KotfisAgnieszka KarczmarczykKatarzyna Kwiecień-JaguśAgnieszka Kijo-KleczkowskaKatarzyna LachowiczAgnieszka KłopotowskaKatarzyna Leśniewska-NapierałaAgnieszka Kozioł-KozakowskaKatarzyna LeszczynskaAgnieszka KujawskaKatarzyna ŁubiechAgnieszka KulikKatarzyna M. MiszczyńskaAgnieszka LasotaKatarzyna Marciniak-ŁukasiakAgnieszka Lecka-AmbroziakKatarzyna MarkiewiczAgnieszka ŁyśKatarzyna Mocny-PachońskaAgnieszka MarekKatarzyna MorkaAgnieszka Medyńska-JuraszekKatarzyna NabrdalikAgnieszka MicekKatarzyna Nowakowska-LipiecAgnieszka Mierek-AdamskaKatarzyna NowomiejskaAgnieszka MlynarskaKatarzyna Okulicz-KozarynAgnieszka Napiórkowska-KrzebietkeKatarzyna PalusAgnieszka Olszewska-GuizzoKatarzyna PawlewiczAgnieszka OperatKatarzyna Pietrucha UrbanikAgnieszka OrkuszKatarzyna Piwowar-SulejAgnieszka PacKatarzyna PlebanczykAgnieszka Pajdak-StósKatarzyna PrzybyłaAgnieszka PiekaraKatarzyna PrzybylowiczAgnieszka Policht-LatawiecKatarzyna RędzińskaAgnieszka PregowskaKatarzyna RolfAgnieszka SapaKatarzyna Sienkiewicz-MałyjurekAgnieszka SkowronKatarzyna SikorskaAgnieszka SmalecKatarzyna SkrypnikAgnieszka SpringerKatarzyna Stala-SzlugajAgnieszka StacherzakKatarzyna StasiukAgnieszka Świdnicka-SiergiejkoKatarzyna StaszakAgnieszka Szlagatys-SidorkiewiczKatarzyna Sterkowicz-PrzybycieńAgnieszka Tomczyk-WarunekKatarzyna StyszkoAgnieszka TruszkowskaKatarzyna SutkowskaAgnieszka TrystulaKatarzyna ŚwiąderAgnieszka WareńczakKatarzyna SygitAgnieszka WerenowskaKatarzyna SzalonkaAgnieszka WilkowskaKatarzyna SzramowiatAgnieszka Wojewodzka-WiewiorskaKatarzyna TomaszekAgnieszka Wojtczuk-TurekKatarzyna TworekAgnieszka ZawiejskaKatarzyna WartalskaAgnieszka ŻelaźniewiczKatarzyna WątorAgnieszka Zembrón-ŁacnyKatarzyna WolnickaAgostino CarboneKatarzyna Zaborowska-SapetaAgostino GaudioKate A. DuchownyÁgoston TemesiKate ChatfieldAgota Giedrė RaišienėKate DaviesÁgota HorelKate EarlyÁgota KunKate FrazerÁgueda Cervera-GaschKate K. ChappellAgustín Aibar-AlmazánKate KingsburyAgustín AriñoKate M. ChittyAgustín Díaz-ÁlvarezKate MaslinAgustín Javier Simonelli-MuñozKate ParkerAgustín Llopis-GonzálezKate SangAgustín Rodríguez-EstebanKate Schwartzkopf-PhiferAhmad Reshad OsmaniKate SeymourAhmad SalmanKate StephenAhmad Taufek Abdul RahmanKatelyn F. RommAhmadreza Shirvani DastgerdiKatelyn HollidayAhmar RaufKatelyn RommAhmed AbdallaKateřina BerkováAhmed AbdeenKaterina BougiatiotiAhmed AbdelghanyKaterina GiouriAhmed AbdelradyKaterina GlumbikovaAhmed Al-BayatiKaterina GrafanakiAhmed Al-ImamKateřina IvanováAhmed BakillahKateřina JančaříkováAhmed GadKaterina KavalidouAhmed HieawyKaterina PapadimitriouAhmed KhaledKaterina PsarraAhmed M. SayedKaterina StamatelatouAhmed MostafaKateřina ValentováAhmet BaydurKateryna Lysenko-RybaAhmet TanhanKath SellickAi Ni TeohKatharina AlackAida Cadellans-ArrónizKatharina DiehlAida KhakimovaKatharina HanselAida MetoKatharina HüfnerAida PitarchKatharina K. RallAidan CroninKatharina KlausAidan McGlincheyKatharine BrotonAidan RocheKatharine C. ReynoldsAidin SalamzadehKatharine WalterAikaterini BougiatiotiKatherina HeinrichsAikaterini KonstantinidiKatherine A. BirchenallAila AholaKatherine ChenAilsa G. NivenKatherine CollinsAiman AliKatherine ConantAimee PinkKatherine DarlingAimei MaoKatherine E. McDonellAïmen KhacharemKatherine H. IngramAimilia KarapostoliKatherine HertleinAine AventinKatherine JacksonAinhoa Fernández-AtutxaKatherine K. M. StavropoulosAinhoa IzaguirreKatherine Ka Pik ChangAinhoa Ulibarri OchoaKatherine KochAini FarmaniaKatherine LeungAinoa Biurrun GarridoKatherine Lynn O’ShaughnessyAinoa Roldán AliagaKatherine M. RossAiping LiuKatherine Ott WalterAiqin ShiKatherine PiccoloAiri HakkarainenKatherine S. SalamonAirlane AlencarKatherine SchaumbergAirlane Pereira AlencarKatherine SemrauAirton C. MartinsKatherine TheallAishwarya IyerKathi R. TrawverAiste DirzyteKathleen BorgmannAitana Fernández-SogorbKathleen CarterAitor CocaKathleen ChellAitor Garay-SánchezKathleen DeSantis KlinichAitor Oyarbide-ZubillagaKathleen FarrandAizat KhairiKathleen KevanyAjay Kumar YagatiKathleen LeemansAjay MajorKathleen MonahanAjay PalKathleen MorganAjay Pratap SinghKathleen PorterAjit Kumar KarnaKathleen RatajczakAjjampura C. VinayakaKathrin FeigeAkansha SinghKathrin KirchnerAkebe Luther King AbiaKathryn A. CarrollAkhalesh Kumar ShakyaKathryn BowlesAkhila VeerubhotlaKathryn GreeneAkifumi EguchiKathryn K. OstermaierAkihiko HataKathryn M. LeifheitAkihiko KatayamaKathryn MckayAkihiro HosoyaKathryn NicholsonAkihiro MasuyamaKathryn Ries TringaleAkihiro ShiinaKathryn SindenAkihiro TamuraKathy KnightAkihisa HataKathy LewisAkihito HagiharaKathy McKayAkiko SuzukiKathy Merlock JacksonAkio FujiwaraKathy TannousAkio KimuraKathy WiningsAkira IshidaKathyayini P. GopalakrishnaAkira KanekoKati KuittoAkira KyanKátia Cândido CarvalhoAkira MonjiKatia ComteAkira ShibanumaKatia IskandarAkira UmemuraKatia Laura SidaliÁkos M. LőrinczKatia M. Costa-BlackAkosua O. AddoKatie CurranAkpovire OduaranKatie FrenisAkshay SharmaKatie HeinrichAkshaya Srikanth BhagavathulaKatie M. HeinrichAkter HossainKatie M. VinopalAku VisuriKatie MacLureAlain FreyKatie McGillAlain MassartKatie McQuaidAlain TchagangKatie MorganAlaleh DadvariKatie N. BrownAlan BeckKatie WynneAlan BonderKatiuscia MannaroAlan CrouchKatja BoehmAlan D. ChristianKatja I. BraamAlan HayesKatja PynnönenAlan J. NimmoKatri JalavaAlan KellyKatrien De WildeAlan KinlawKatrien LuijkxAlan LillKatrin CunitzAlan M. BeckKatrin LangAlan S. KaufmanKatrin Schaper-GerhardtAlan W. GertlerKatrin ZiserAlana Kasahara NevesKatrina Bell McDonaldAlana KluczkovskiKatrina HutchinsonAlane B. O’ConnorKatrina LambertAlan-Miguel ValdezKatrine KrygerAlastair N. GossKatriona O’SullivanAlba Carrero-PlanellsKatsuhiko SuzukiAlba Elena Martinez SantosKatsuhiro ItoAlba García-CidKatsuki TsuchiyamaAlba González De La RozKatsuko KajiyaAlba Hernández-MartínezKatsuo OshimaAlba Ibáñez GarcíaKaty CampbellAlba Marcos-DelgadoKaty GreenlandAlba Martínez-GarcíaKaushi S. T. KanankegeAlba Nicolás-BoludaKavindra Kumar KesariAlba Paris-AlemanyKavita SharmaAlba RoldanKavitha PalaniappanAlban Fouasson-ChaillouxKaWang LiAlbère J. A. KökeKay CurrieAlbérico RosárioKay HamerAlbert BoonstraKay (Nobuko) HonguAlbert F. SmithKayako SakisakaAlbert NienhausKayla BakerAlbert Pagès BachKayode Kolawole EluwoleAlbert Qianyi FuKayoko TakahashiAlbert RubbenKazi Parvez FattahAlbert Sanchez-NiuboKazi TamaddunAlbert SerràKazimierz KarolewskiAlbert SeseKazuhiko KibayashiAlbert WabneggerKazuhiko KotaniAlbert YeungKazuhiro P. IzawaAlberto A. AmarillaKazuhisa KodaAlberto Adanero VelascoKazuki FukuiAlberto ÁlvarezKazuki IdeAlberto Arnedo-PenaKazuki ShimizuAlberto BarbieriKazunari OnishiAlberto BeharKazunori IkegamiAlberto BorraccinoKazunori NagasakaAlberto BosinoKazuo KatohAlberto CellaKazushi NumataAlberto Cerezo-NarváezKazuya InokiAlberto ChighineKazuya InoueAlberto Comesaña-CamposKazuya MorikawaAlberto Férriz-ValeroKazuyuki KasaharaAlberto ForteKe TianAlberto Frutos Pérez-SurioKearney MonicaAlberto GambelliKebede KefeniAlberto Gómez-MármolKebogile MokwenaAlberto González-GarcíaKees DoevendansAlberto J. Martín-RodríguezKehinde ObamiroAlberto López-MartínezKei NakagawaAlberto Marcos Heredia-RizoKei NakajimaAlberto MatteelliKeiko OgawaAlberto MavilioKeiko UnnoAlberto ModeneseKeisuke FujiiAlberto Moreno DoñaKeisuke KokubunAlberto Nuviala-NuvialaKeisuke KuwaharaAlberto Quílez-RobresKeisuke MiyauchiAlberto Rodríguez-ArchillaKeisuke MurakamiAlberto Romero-AniaKeisuke ShioseAlberto Ruiz-ArizaKeita HiraiAlberto SardellaKeith ChristensenAlberto SardiKeith FriedmanAlberto TazioliKeith JoinerAlbin WagenerKeith OrdAlbina GazizulinaKeith PecorAlbina KepalaiteKelechi NjokuAlbina Kinga MoscickaKelley GraydonAlbina OrlandoKelly AlexanderAlda TronconeKelly Cristina TonelloAldina VenerosiKelly CukrowiczAldo Arranz-LópezKelly De JesusAldo BertazzoliKelly E. JohnsonAldo Bruno GiannìKelly HoltAldo CostaKelly KingonAldo Franco DragoniKelly MunkittrickAldo José Fontes-PereiraKelly-Ann BowlesAldo MuroKelsey HudsonAldo Muro Jr.Kelsey UfholzAldo WinklerKelvin C. FongAldona KubicaKelvin KongAldona Maria PiwkoKelvin T. W. NgAldona PiwkoKelvin WallsAldona ZawojskaKemal GörürAlejandra Alonso-CalveteKemmyo SugiyamaAlejandra AlvaradoKen BataiAlejandra Benitez Donají ArciniegaKen Ing Cherng OngAlejandra Donají Benítez ArciniegaKen KaripidisAlejandro Alvarado-LassmanKen KiyonoAlejandro AmillanoKen NahAlejandro Canedo-GarcíaKen RobertsAlejandro Castañeda-MirandaKen ShiratoAlejandro De La Hoz SerranoKen TakahashiAlejandro GarcíaKendra KattelmannAlejandro Martínez-RodríguezKendra WilliamsAlejandro Moreno-RangelKeng-Fu HsuAlejandro Muñoz-LopezKengo FujiiAlejandro Pérez-CastillaKengo IshiharaAlejandro Rodríguez-FernándezKengo NakamuraAlejandro Ros-GálvezKênia Mara Baiocchi De CarvalhoAlejandro Ruiz-PadilloKenichi GotoAlejandro Sánchez-PayKenji DomaAlejandro Santos-LozanoKenji ShimazoeAlejandro SanzKenji TakahashiAlejandro Sanz-ParisKenneth A. EatonAlejandro Silva-PalaciosKenneth A. KnappAlejandro Vega-MuñozKenneth BradburyAleksandar RadicKenneth C. LandAleksandar ValjarevićKenneth Steven PlanteAleksandar VišnjićKenneth SutherlandAleksander Jerzy OwczarekKenneth VerbovenAleksander KrólKenneth YoungAleksander LotkoKennio Ferreira-PaimAleksander PanasiukKenshirou ShiraishiAleksander TedstoneKenta FurutaniAleksandr Byron StefaniakKenta IitaniAleksandr N. IshmatovKenta MoritaAleksandr RyakhovskyKenta MurotaniAleksandra BawiecKentaro IwataAleksandra BryndalKentaro MisakiAleksandra BurgielKentaro TojoAleksandra GawelKeren GrinbergAleksandra Gaworska-KrzemińskaKerr GrahamAleksandra GulcKerroum DerbalAleksandra JarońKerry O’BrienAleksandra Jawiarczyk-PrzybyłowskaKerstin M. PalombaroAleksandra KlichKerstin WitteAleksandra KnezevKesha Baptiste-RobertsAleksandra KołotaKeshav Raj PaudelAleksandra KołtuniukKesheng ShuAleksandra KosanicKęstutis BaltakysAleksandra KristoKęstutis RimaitisAleksandra M. BondžićKęstutis ValančiusAleksandra NowyszKeumseok KohAleksandra PawlaczykKeunBaDa SonAleksandra PfeiferKeunhye LeeAleksandra Piechota-PolanczykKeun-Yeong JeongAleksandra RadeckaKevimy AgossaAleksandra RogowskaKevin BottomleyAleksandra SędzikowskaKevin C. MooreAleksandra StupakKevin CarriereAleksandra SuchaneckaKe-Vin ChangAleksandra SuwalskaKevin ChenAleksandra SzydłowskaKevin Chien-Chang WuAleksandra SzylińskaKevin ClaassenAleksandra UruskaKevin CoulouresAleksandra WilkKevin D. DamesAleksi VarinenKevin DanielsAleksy KwilinskiKevin DarbyAlena FurdovaKevin FlahertyAlena OulehlovaKevin FonsecaAlenka SkerjancKevin FujiAleš UrbanKevin GlintonAlessandra BaldiKevin JeanAlessandra BorsettiKevin K. ByonAlessandra CostanzaKevin KantonoAlessandra De MatteisKevin LanzaAlessandra Di CagnoKevin M. KellyAlessandra DonatoKevin ModestoAlessandra DubbiniKevin MurrayAlessandra FermaniKevin PatersonAlessandra FerramoscaKevin PottieAlessandra FiorettiKevin T. LeichtAlessandra JagerKeyang LiuAlessandra LintasKeyla Vargas-RománAlessandra N. BazzanoKeyne CharlotAlessandra PaunczKezban Yagci SokatAlessandra PrioreKhadga Bahadur ShresthaAlessandra PrioreschiKhaled Al-KassimiAlessandra RenziniKhaled MukaddamAlessandra SannellaKhaled QanudAlessandra SinopoliKhaled ShaabanAlessandra SpennatoKhalid KoserAlessandra ValerioKhan GareeAlessandra ZicariKhara SauroAlessandro AmbrosiKhashayar AfshariAlessandro AndreucciKhatia AntiaAlessandro AntonelliKhaula KhatlaniAlessandro ArduinoKhayriyyah Mohd HanafiahAlessandro ArenaKheng Siang NgAlessandro BoscoKhondokar Mizanur RahmanAlessandro CavalloKhosrow RezvaniAlessandro CicolinKhushboo MunirAlessandro De SireKhushminder RaiAlessandro Del VecchioKhushwant Singh BhullarAlessandro FancelluKhyber AlamAlessandro FeolaKi Han SongAlessandro FiorettiKi Nam JinAlessandro FrolliKiana Jafari MeimandiAlessandro GiorgettiKiara LewisAlessandro H. Nicolai RéKichul ShinAlessandro IeraciKiemute OyiboAlessandro Lo PrestiKien NguyenAlessandro MalobertiKieu Ngoc LeAlessandro Mandurino-MirizziKieu The Loan TrinhAlessandro MarroniKiffer CardAlessandro MartiniKihong HongAlessandro MatteKihong ParkAlessandro MazzoniKihye HanAlessandro MeduriKijin KimAlessandro MenozziKikombo NgoyAlessandro MianiKikuo IkarashiAlessandro N. VargasKim BlasdellAlessandro NardiKim BlomqvistAlessandro NotaKim BloomfieldAlessandro PerrellaKim BulkeleyAlessandro PezzoliKim ClarkAlessandro PorrovecchioKim DooleyAlessandro PresentatoKim L. StansburyAlessandro RampelloKim LichtveldAlessandro RizzoKim LowellAlessandro RodolicoKimberley Brownlee BrownleeAlessandro SanduzziKimberley ShoafAlessandro SapienzaKimberley WangAlessandro ScanoKimberly A. SullivanAlessandro SolipacaKimberly DionAlessandro UmbricoKimberly E. StephensAlessandro VittoriKimberly G. FuldaAlessandro ZorziKimberly KellyAlessia ArcaroKimberly LewisAlessia CogatoKimberly Rapp HartsonAlessia D’AndreaKimihiro HinoAlessia Di BlasioKimitake SatoAlessia FazioKimmo VirtanevaAlessia GalloKimon ChristanisAlessia GrigolettoKimon DivarisAlessia RenziKin Sun ChanAlessia SpadaKine JohansenAlessia TognettoKing Fai HungAlessia TropeaKinga GawelAlessio BocediKinga Huminska-LisowskaAlessio CappelliKinga KimicAlessio ContiKinga LinowieckaAlessio Danilo ComisKinga MusiałAlessio FacchinKinga Nagy-PércsiAlessio GiubellinoKinga PolanskaAlessio Giuseppe MorgantiKinga SzopińskaAlessio GoriKinga TurzoAlessio MasiKingsley E. AghoAlessio MatizKira BacalAlessio PorrecaKira LanckerAlessio RossiKiran Maini GerhardssonAletta MillenKiran NandalikeAlex A. OlmosKirk MillerAlex AlbertKirsi M. IsoherranenAlex BorodinKirsten BerdingAlex Buoite StellaKirsten SpencerAlex C. VargheseKirsten Staggs AlmbergAlex CostleyKirstin VachAlex G. StewartKirsty J. BoltonAlex HanKirsty OrmstonAlex Harley CrispKirtikar ShuklaAlex J. Van DuinenKishor HadkhaleAlex LiberKishu RanjanAlex MichelsKisoo ParkAlex MojonKisook KimAlex Pauvolid-CorrêaKi-Sun LeeAlex PintoKit WongÀlex SanzKitaoka YuAlex ViguerieKitsaras GeorgeAlex Wing Hong ChinKiwon LeeAlexander Achalandabaso-OchoaKiyoshi SaigusaAlexander BerezinKiyoshi YoshinoAlexander BiesKjell B. EsserAlexander BungeKjell Hansson MildAlexander ChanKlára DaďováAlexander E. PlatonovKlara SaczukAlexander G. MathioudakisKlasien HorstmanAlexander GovarisKlaske Van KammenAlexander H. MaassKlaus BoehnkeAlexander HemprichKlaus Dieter StillerAlexander IvannikovKlaus DieterichAlexander Ja’acob-GorbanovichskyKlaus FuchsAlexander KerstenKlaus GratzAlexander KeulKlaus GründlerAlexander KirpichKlaus KayserAlexander KraemerKlaus KunzmannAlexander LarcombeKlaus L. P. VasconcellosAlexander M. ShestopalovKlaus Peter LattéAlexander MacKenzieKlaus ZiererAlexander MininKlavdia Oleschko LutkovaAlexander PelyasovKleoniki Natalia PetrouAlexander PottKleopatra NikolopoulouAlexander RymanovKneginja RichterAlexander SboevKnut Lehre SeipAlexander SchenkKnut NeumannAlexander SchulanKnut SkulbergAlexander SeifertKnut-Inge FostervoldAlexander SerguninKobra EtminaniAlexander StonerKoç AhmetAlexander T. H. WuKoch AndreasAlexander TestaKoen De SchrijverAlexander WaitsKoen PonnetAlexander WongKoffi DjamanAlexandr CeasovschihKoh MizunoAlexandr ParlesakKohei MakitaAlexandr V. KrylovKohki OkadaAlexandra AgostiniKoichi NaitoAlexandra AlbuquerqueKoji OnomotoAlexandra BourdotKoji OtaniAlexandra DevineKoji TakahashiAlexandra EconomouKoji UchiyamaAlexandra FernandesKoji WadaAlexandra Ferreira-ValenteKoji YamatsuAlexandra FoscolouKökönyei GyöngyiAlexandra HennesseyKo-Lin KuoAlexandra HorobetKo-Ling ChanAlexandra Ioana MihăilescuKollarigowda RavichandranAlexandra IoanidKomali KantamaneniAlexandra Jiricka-PürrerKon Hee KimAlexandra JitǎreanuKongtae RaAlexandra KrollKonrad A. SzychowskiAlexandra LaurentKonrad FurmanczykAlexandra M. PreisserKonrad S. JankowskiAlexandra MateiKonstantin B. IlijevićAlexandra MavroeidiKonstantin VolchoAlexandra PereiraKonstantina KatselaAlexandra Pérez-FerreirósKonstantina VasilakopoulouAlexandra Sofia MendesKonstantinos A. GatzoulisAlexandra SolomouKonstantinos ArgyropoulosAlexandra TragakiKonstantinos AzisAlexandra UllstenKonstantinos ChristopoulosAlexandra V. KulinkinaKonstantinos DimitriouAlexandre BarbosaKonstantinos DritsasAlexandre Fonseca BrandãoKonstantinos G. KyriakoulisAlexandre Gonzalez-RodriguezKonstantinos IoannouAlexandre LamasKonstantinos KafetsiosAlexandre PalmaKonstantinos KompotisAlexandre RibeiroKonstantinos KotsisAlexandre Souto MartinezKonstantinos PapadimitriouAlexandre SzaboKonstantinos PlakasAlexandre VieiraKonstantinos SidiropoulosAlexandrina MunteanKonstantinos TourlakopoulosAlexandro FortunatoKonstantinos VergosAlexandros MaziotisKontxi Martinez De San VicenteAlexandros NikitasKoos Jaap Van ZwietenAlexandros PsarrisKoral JeromeAlexandros RovasKorawit OrkpholAlexandru BodislavKory ZimneyAlexandru BurlacuKoshy KoshyAlexandru SoriciKostas KasiotisAlexey ChubarovKosuke InoueAlexey DudarevKota KodamaAlexey GusevKotaro ShideAlexey MelnikovKotomi SakaiAlexey SarapultsevKouichi MiuraAlexey V. RakovKouji H. HaradaAlexey VoinovKouji MiyazakiAlexis BelianinKounosuke TomoriAlexis DescathaKoustuv DalalAlexis GalanisKoutarou SueokaAlexis L. MrazKovalik Jean-PaulAlexis Le FaucheurKow-Tong ChenAlexis LionKoyin ChangAlexis M. TemkinKrassimir MarkovAlexis PapathanassisKrim K. LaceyAlexis SantosKrishan K. VermaAlfaz MahmoodKrishan SoodAlfio MarazziKrishna GullaAlfonso Alvarado-LorenzoKrishna HortAlfonso Chaves-MonteroKrishna Mohan SurapaneniAlfonso De La Rubia RiazaKrishna RokaAlfonso García-MongeKrishnan GanapathyAlfonso Gastélum StrozziKristaps JurjansAlfonso InfanteKristen M. NishimiAlfonso J. Gil-LopezKristen N. SobbaAlfonso Maria PonsiglioneKristen PageAlfonso Martínez-MorenoKristen Pogreba-BrownAlfonso Martínez-NovaKristen R. BreitAlfonso MastropietroKristen RappazzoAlfonso Meneses MonroyKristian LarsenAlfonso Morales UrzuaKristijan SkokAlfonso Penichet-TomásKristin HoranAlfonso Salgado RuizKristin JergerAlfonso Vargas MacíasKristin M. EcclesAlfred NordmannKristin SainaniAlfredo CiniglioKristina BučarAlfredo G. CasanovaKristina GorsetaAlfredo Gea SanchezKristina KjaerheimAlfredo Rosado MuñozKristina LindvallAlhassan Yakubu AlhassanKristina LožienėAli AhmadianKristina MarintsevaAli Al KaissiKristina MattssonAli Al-AhmadKristina MeyerAli BehnoodKristina PennistonAli BoolaniKristina PerošAli DaneshkhahKristina RamanauskienėAli DaraeiKristina RapuanoAli Ebadi TorkayeshKristina TryggAli Enes DingilKristina VatchevaAli FarnoudKristina WanyonyiAli HassanKristina WhitworthAli Hassan SodhroKristina Žardeckaitė-MatulaitienėAli JozaghiKristine GebbieAli Khosravanipour MostafazadehKristine L. WerlingAli MobasheriKristine M. KrajnakAli MoghtaderiKristine SorensenAli Sadeghi HabibabadKristján KristjánssonAli Shariq ImranKristy LangermanAlice BonomiKrisztián KisAlice GiontellaKrisztian MagoriAlice HorowitzKrisztián NyesteAlice LeeKrisztina KovácsAlice M. TurnerKrisztina MarthaAlice MadoniaKrisztina PohóczkyAlice MannocciKritika PoudelAlice NoblinKrystal LynchAlice NormandKrystal WarmothAlice R. VillalobosKrystian HeffnerAlice StreetKrystian ObolewskiAlice Yee-Men JonesKrystian SkubaczAlicia AguilarKrystyna GutkowskaAlicia Arenas MorenoKrystyna Kazimierowicz-FrankowskaAlicia Ayerdi GotorKrystyna PawlasAlicia Blanco-GonzalezKrystyna PoznańskaAlicia BoltKrystyna RejmanAlicia Cuesta GómezKrzystof TomasiewiczAlicia KraayKrzysztof BłażejczykAlicia Louise RihnKrzysztof ChmielowiecAlicia Mohedano SorianoKrzysztof FornalskiAlicia NunezKrzysztof FrączekAlicia Orea-GinerKrzysztof GórnickiAlicia PeñalbaKrzysztof GorynskiAlicia Ramírez-OrellanaKrzysztof HermanAlicia Rodríguez-BarberoKrzysztof J. CzarnockiAlicia SendrowskiKrzysztof JóźwiakowskiAlicia TamaritKrzysztof JurekAlicia TejeraKrzysztof KaczmarekAlicja BortkiewiczKrzysztof KarpowiczAlicja DomagalaKrzysztof KilianAlicja Dziuba-SłoninaKrzysztof KowalAlicja FormaKrzysztof KrukowskiAlicja KalusKrzysztof KrystaAlicja KicińskaKrzysztof KuttAlicja KluczykKrzysztof LetachowiczAlicja Kot-NiewiadomskaKrzysztof MalikAlicja NychKrzysztof OtrembaAlicja PecioKrzysztof PańcikiewiczAliki-Eleni FarmakiKrzysztof PękalaAlin Laurenţiu TatuKrzysztof PiaskowskiAlina BadulescuKrzysztof R. KarszniaAlina ButuKrzysztof RogatkaAlina CernasevKrzysztof Sas-NowosielskiAlina ChiracuKrzysztof SchabowiczAlina DimaKrzysztof StanisławskiAlina Huzui-StoiculescuKrzysztof SzaflikAlina KuryłowiczKrzysztof SzamałekAlina MomotKrzysztof SzyfterAlina ShevorykinKrzysztof WięckowskiAlina Simona RusuKsenija Rener-SitarAlina SturzaKsenija Tušek-BuncAlina SvechkinaKuang-Chi ChenAlina TrifanKuang-Chung TsaiAlina ZurekKuanhui XiangAline Alves FerreiraKuan-Yin LinAline Boveto SantamarinaKuan-Ying HsiehAline Macedo De OliveiraKuei-Hui ChanAlireza AbouhosseinKuei-Pin KuoAlireza AfshaniKuen-Chang HsiehAlireza BabaeiKuen-Cheh YangAlireza DaneshkhahKuen-Yuh WuAlireza FarzampourKulothungan GunasekaranAlireza NiliKumail K. MotianiAlireza SahebgharaniKumi HirokawaAlisa BoutinKun JiaAlison EastmanKunal DhimanAlison HalfordKunHo LeeAlison HerrmannKun-Hsien TsaiAlison K. VenturaKun-Hua LeeAlison LaneKunihiko NakaiAlison McFaddenKuniyoshi ToyoshimaAlison MunroKun-Lin TsaiAlison OrmsbyKun-Ling TsaiAlison R. CoelhoKun-Tsung LeeAlison Sally PoultonKun-Yi HsinAlison Snyder ValierKuo YuanAlison TovarKuo-Chuan LinAlison WoodKuo-Hsun WenAlissa Der SarkissianKuo-Hu ChenAlisson BorgesKuo-Jung LeeAlistair GriffithsKuo-Shing ChenAlix WinterKuo-Wei SuAlix WoolardKurosch YazdiAlizée LehouxKurt A. EscobarAlla Alla PakinaKurt AmmerAlla KushnirKurt G. M. De CramerAllah DittaKurt HahlwegAllan G. HillKurt HeutschiAllan HawasKurt KotrschalAllan LeggeKurunthachalam KannanAllan Libanio PelissariKusheng WuAllan MunroKuulo KutsarAllan PaceyKwan Ok LeeAllen KrautKwan Soo ChenAllen P. UgargolKwang Woo NamAllison BarryKwangseok HongAllison FordKwisoon ChoeAllison HodgeKwok NgAllison Huff MacPhersonKwok-Kuen CheungAllison J. LopatkinKwok-Wing ChauAllison KupscoKwong Nui SimAllison LewisKwua-Yun WangAllison RossKyeongmin KwakAllison SweeneyKyle EbersoleAllison VaughnKyle K. H. TanAllysson Allex AraújoKyle MonahanAlma Mačiulytė-ŠniukienėKyle MoodyAlmotasembellah AbushabanKyle RichAlmudena Buesa-EstellézKyle SueAlmudena OrdoñezKylie GwynneAlois W. SchmalwieserKylie KingAlok ChoudharyKym RaeAlok K. VermaKymberley BennettAlok PaulKyoko AsakuraAlon BartalKyoko NagaoAlonso LucilleKyonghwa KangAloys BorgersKyoung KimAlphonsinec MukamuhirwaKyoung Shin ParkAlpo VuorioKyoungkyu JeonAltaira D. DearbornKyoungmin ParkÁlvaro Astasio PicadoKyoung-Sook JeongÁlvaro Astasio-PicadoKyriaki KalimeriÁlvaro Briz-RedónKyriaki MyrissaÁlvaro Efrén Domínguez RebolledoKyriaki-Maria FameliAlvaro Fernandez-LunaKyriakos SouliotisÁlvaro Francisco Lopes De SousaKyrre KausrudAlvaro Gallo CordovaKyung Eun JahngAlvaro García Del CastilloKyung Hee LeeÁlvaro Huerta OjedaKyung Hyun OhÁlvaro Iborra-MarcosKyung Taek RimAlvaro Lopes DiasKyungchul SongAlvaro Lopez-SamanesKyung-Eun (Anna) ChoiÁlvaro MourenzaKyunghee LeeAlvaro PérezKyung-Hun KimAlvaro VergésKyunghwa ChoAlvin J. X. LeeKyungHwa SeoAlvin TranKyung-Hyun SuhAlyona IvanovaKyungo KimAlys HavardKyung-Sook BangAlysa PomerKyungyeol (Anthony) KimAlyson J. ChroustKyung-Yong ChungAlyx TaylorKyuwan LeeAlžbeta PultznerováL. Bruno RuestAmad ZafarL. Douglas RiedAmal K. MitraL. E. Meur NolwennAmalia JiménezL. M. O. MartinsAmalia Raquel Pérez NebraL. YuAmalia SilleroLacey McCormackAman SinghLadislav BatalikAmand FührerLadislav BláhaAmanda CalebLadislav KazmerAmanda CheungLadislav ŠtěpánekAmanda CordingLady Catherine Cantor-CutivaAmanda Froehlich ChowLaetitia HauretAmanda GoodsonLaibing JiaAmanda HickeyLaila CraigheroAmanda IglesiasLaila KristoffersenAmanda Julie NevilleLaima BulotaitėAmanda Kufta JenkinsonLaima JesevičiūtėAmanda LeeLaima Jesevičiūtė-UfartienėAmanda McClainLaima SapezinskieneAmanda MisselLaio O. SemanAmanda N. Szabo-ReedLaisa Liane Paineiras-DomingosAmanda NeriniLaith AbualigahAmanda PalmerLakesha AndersonAmanda Perkins-BallLakhdar BoukerrouAmanda SonnegaLakshmi VijayakumarAmanda StaffordLaleh JamshidiAmanda WilsonLaleh YerushalmiAmandeep PabblaLama RaquelAmandio GraçaLambert SpaanenburgAmankeldi SalybekovLambros T. DoulosAmar KanekarLampros K. MichalisAmar MehtaLan JinAmar ParvateLan XiongAmarish YadavLana IvanitskayaAmaro Olimpio PereiraLana MulierAmaury SouzaLance KeeneAmaya Erro GarcésLandy LuAmber EdinoffLanny RosenwasserAmber VermeeschLan-Szu ChouAmelia BarcelliniLapaire Jean-RémiAmelia DíazLaPorchia CollinsAmelia K. SearleLara Aleluia ReisAmelia M. SilvaLara CostantiniAmélia MarchãoLara Guedes De PinhoAmelia Maria GamanLara JurkovicAmélia Simões FigueiredoLara OwenAmelia TrematerraLara TavoschiAmelia V. García-LuengoLaran ChettyAmérica Chávez-MartinezLarbi BoubchirAmerigo GiudiceLarianne CollinsAmey S. TilakLarissa A. McGarrityAmi RokachLarissa Almeida MartinsAmie LundLarissa GalattiAmílcar OliveiraLarissa Loures MendesAmin AlizadehLarissa WieczorekAmin BemanianLarrell L. WilkinsonAmin EbrahimiLarry AbelAmin Hosseinian-FarLarry NelsonAmin MobasheriLars De VroegeAmin MojiriLars DietrichAmin ShahrokhiLars GerholdAmina MunirLars KayserAminda O’HareLars RylanderAmir AkbarzadehLashan PeirisAmir BhochhibhoyaLasse LehtonenAmir GolmohamadiLasse Suonperä LiebstAmir GrinsteinLászló BerényiAmir Mohammad MalvandiLászló Csaba MangelAmir MosaviLászló EgyedAmir NafiLászló MiklósAmir S. SirajLászló ÓzsváriAmira ElasraLászló SzabóAmirul MukmininLatasha SmithAmit KumarLaura Adelaide Dalla VecchiaAmit LadaniLaura Alonso-MuñozAmit PantLaura Andreu-PejóAmit U. RaysoniLaura Antonio-ZancajoAmjad ShraimLaura Arnau SabatésAmlan HaqueLaura BarnabeiAmmar AchrafLaura BragonzoniAmna AnjumLaura BulgariuAmna JavedLaura C. Sánchez-SánchezAmnon A. BergerLaura Cabiedes MiragayaAmol S. PatwardhanLaura CarrAmorim Batista Odinea MariaLaura CarrascosaAmos DarkoLaura CataldiAmos RussakLaura CensiAmosy M’KomaLaura CerviAmparo Melián-NavarroLaura CriscuoloAmparo OliverLaura Cristina RusuAmparo Verdú-VázquezLaura De Armas-RilloAmr Abdulfattah Hausien HassanLaura Del Carmen Sánchez SánchezAmr El RefaieLaura Delgado-LobeteAmr HassanLaura Di RenzoAmritha BhatLaura E. BedfordAmund RiiserLaura E. WalkerAmutha RamadasLaura Elena MarinașAmy B. SmithLaura ElomaaAmy BestmanLaura EspínAmy BrowerLaura FioriniAmy E. LesenLaura FreemanAmy GriffinLaura Fusar-PoliAmy HinkelmanLaura Gallardo-AlfaroAmy IshamLaura GangosoAmy J. ElliottLaura GibsonAmy L. ShaverLaura GiessingAmy LiLaura GiganteAmy M. MooreLaura GilbertsonAmy Miner RossLaura GuidettiAmy Østertun GeirdalLaura HealyAmy PedenLaura HopkinsAmy TaetzschLaura I. Garay-JimenezAmy WrightLaura IacorossiAn NevenLaura IosifAna AdanLaura Jane BrubacherAna Alexandra GoraLaura Jane SahmAna Álvarez MuelasLaura JefferiesAna AmanteLaura KatusAna Amaro AgudoLaura KudrnaAna Ancheta ArrabalLaura Lacomba-TrejoAna Aurora Díaz-GavelaLaura LessardAna AzevedoLaura Lopez RomeroAna B. MasedaLaura Lopez-FernandezAna B. Sánchez-GarcíaLaura López-LópezAna BagüésLaura LossiAna Beatriz Furlanetto PachecoLaura Louise NicklinAna Belén Barragán MartínLaura M. KeaverAna Belén Fraile-BermúdezLaura MacdonaldAna Belen Navarro-PradosLaura MaltryAna Belén Santos-OlmoLaura Marcos-ZambranoAna Beleń Subirón-ValeraLaura María CompañAna Belen Tulcanaza PrietoLaura MasonAna Belen VicenteLaura Muñoz-BermejoAna Beltrán VelascoLaura NathansAna BerlinLaura NunesAna BlascoLaura OrsoliniAna BorovečkiLaura PalacioAna BujánLaura PalombiAna C. Meira CastroLaura ParteAna Carolina Barco LemeLaura PiccardiAna Carolina De CamposLaura PiniAna Catarina AlmeidaLaura PompermaierAna Catarina Vieira De CastroLaura Ponce-RoblesAna Cláudia Mesquita GarciaLaura RodriguezAna Cláudia TeodoroLaura Ruiz-EugenioAna CoelhoLaura SakkaAna ColimLaura SalvadoriAna Cordellat MarzalLaura SannaAna Corte-RealLaura SicheroAna Cristina BragaLaura SparaciAna Cristina Faria RibeiroLaura StefaniAna Cristina GonçalvesLaura Sujo-MontesAna Cristina Rodrigues LacerdaLaura SuppesAna CusumanoLaura Torres-ColladoAna DascaluLaura TosittiAna E. Z. Stroppa-MarquesLaura VicenteAna Escoto CastilloLaura VismaraAna Fernández-AraqueLaura-María Compañ GabucioAna Fernández-TenaLauren A. FowlerAna FialhoLauren A. HindtAna Filipa SilvaLauren A. TurnerAna FonsecaLauren A. ZimmaroAna Gascón-CatalánLauren DundesAna Gómez-TafallaLauren F. GoodmanAna González MarcosLauren H. LoganAna Gonzalez-Nicolas AlvarezLauren H. NaplesAna Gorini Da VeigaLauren HartsteinAna GradissimoLauren LeeAna Isabel BorgesLauren M. SmithAna Isabel Córdoba-IñestaLauren Miller-LewisAna Isabel Corregidor-SanchezLauren MurfreeAna Isabel MaldonadoLauren StaplesAna Isabel Nicolas-SilventeLauren Thomas-SealeAna Isabel NunesLauren Wood ZaseckAna Isabel PlácidoLaurence DedekenAna Isabel Ponce GeaLaurence HamardAna Isabel RibeiroLaurence Lebaron-JacobsAna Isabel SanchoLaurence ParnellAna IvaniševićLaurence Simard-ÉmondAna Julia García-MalinisLaurencia GovenderAna Justicia-ArráezLaurent CapocchiAna KatusicLaurent CarnisAna LeahuLaurent GallonAna LopesLaurent MourotAna López-DuránLaurent SuppanAna LuisLaurentiu PirteaAna Luísa RodriguesLauretta RubiniAna M. BernardosLauriane Suyin Chalmin-PuiAna M. González RamosLaurie Fields DeRoseAna M. Tur-PorcarLaurie MarrauldAna Mª MartínLauro Velazquez-SalinasAna MachadoLavern T. NyamutswaAna MalfitanoLaVerne L. BrownAna Marchena RodríguezLavinia FaleseAna Margarida Luís De SousaLavinia MoleriuAna Margarida Saraiva ValenteLavinia RutaAna María De Caso FuertesLavinia StanAna María GallardoLawrence BurnsAna María García-ArranzLawrence FoweatherAna Maria Gondim ValençaLawrence FultonAna Maria Gonzáles RamosLawrence MabasaAna Maria Mantilla HerreraLazzaro ParaggioAna María Navarro IncioLea TimmermannAna María Pérez-ZuriagaLeah M. Panek-ShirleyAna Maria Rodrigues Monteiro SousaLeah M. WuescherAna María Ruiz-Ruano GarcíaLeah RobertsAna María Salinas-MartínezLeah ShelefAna María Vázquez-CasaresLeal G. SeguraAna MonteiroLeandra UlbrichtAna Mourão MouraLeandro ÁlvarezAna NobreLeandro Alves Dos SantosAna PablosLeandro BritoAna Pastor PérezLeandro Miletto TonettoAna Paula FonsecaLeandro PereiraAna Paula RibeiroLeandro Rodríguez LiñaresAna PereiraLeanne BrownAna Pérez-EscodaLech NierostekAna PerisinLechosław PutowskiAna PicadoLeciel BonoAna R. CostaLeda SivakAna R. SepúlvedaLee BeaumontAna Remesal OrtizLee Byoung-HeeAna Rita FariasLee Greenblatt-KimronAna Rita Gonçalves CabecinhasLee HoonAna Rita MatiasLee Hwan-YoungAna Rita VieiraLee S. NewmanAna RodriguesLee Samüel WannAna Rodriguez LarradLee SohyeAna Rodriguez MeirinhosLee T. DonohueAna Rodríguez-MartínezLee TanAna Rosa Gonzalez-MartinezLee Wei LimAna Rosser LimiñanaLee WhanheeAna SampaioLee-Ching HwangAna Sofia Estevão MendesLeena MaunulaAna Sofia Pinho ColimLeevi AnnalaAna Sofia Ruivo AlvesLei GaoAna Suarez GarciaLei HanAna Teresa Ferreira-OliveiraLei TaoAna Vanesa Valero-GarcíaLei WangAna VelosoLei XingAna Victoria AriasLei ZhangAna VirgolinoLeickness Chisamu SimbayiAnabel Ramos-PlaLeidy Y. GarcíaAnabela Baptista Pereira PaulaLeif Inge MagnussenAnabela Correia MartinsLeif SvenssonAnabela Marisa AzulLeigh A. FrameAnabela RibeiroLeigh CoombesAnahid Ahmadi BirjandiLeigh M. VanderlooAnamaria BechirLeigh WardAna-Maria BolboriciLeigh WilsonAnamaria CiubaraLeila HojatAnamaria JurcauLeila MoslehAna-Maria TaloșLeila TahmooresnejadAna-Maria TănaseLeire AperribaiAna-Maria ZamfirLeis Trabazo María RosauraAnamarija ŠestakLekshmi R. NathAnanda SidartaLena HünefeldAnandha GopalanLena LaemmleAnando SenLena Lipskaya-VelikovskyAnani K. AfanouLena SerafinAnanth Kumar KammalaLena WimmerAnantha HarijithLene MikkelsenAnapaula CupertinoLénia CarvalhaisAnarul Haque MondolLenka LhotskaAnastas PhilalithisLenka MynaříkováAnastasia AnceschiLenka ŠvecováAnastasia AtabekovaLennart ReifelsAnastasia DontiLéo DutriauxAnastasia M. SnellingLeona GilbertAnastasia MitseaLeonard P. RybakAnastasia NikologianniLeonard StoicaAnastasia SaadeLeonardo Alexandre Peyré-TartarugaAnastasia StoopsLeonardo Azael García GarcíaAnastasia TasopoulouLeonardo BascoAnastasia Téllez InfantesLeonardo BecchettiAnastasiia KotlyarovaLeonardo Catalano IniestaAnastassia ZabrodskajaLeonardo CesanelliAnastazia KeiLeonardo CioccaAnat Yaskolka MeirLeonardo D’AcquistoAnatoliy GoncharukLeonardo Dos Santos LimaAnca Florentina GavriluţăLeonardo Jose Mataruna-Dos-SantosAnca GafencuLeonardo Juan Ramirez LopezAnca MehedintuLeonardo LamasAnca Mirela MelintescuLeonardo OggianoAnca Oana DoceaLeonardo PellicciariAnca PanaitescuLeonardo PuppulinAnca PopLeonas ValiusAnca-Maria ClipaLeonel Jorge Ribeiro NunesAndaleb KholmukhamedovLeonid V. AzarnertAnder VergaraLeonid V. BogachevAnders BjørnLeonid V. KalachevAnders C. BoydLeonidas IoannouAnders RasmussenLeonidas PetridisAnderson AraLeonie BrummerAnderson Martins de Souza BrazLeonie HalloAnderson Ribeiro DuarteLeonor Bacelar-NicolauAndi FebrisiantosaLeonor GallardoAndra ZvirbuleLeonor PaisAndrás BikovLeonzio FortunatoAndrás BótaLeopoldo FornerAndraz DovnikLeopoldo Medina SánchezAndré Barembaye SagnaLepiani Díaz IsabelAndre CalladoLeslaw TeperAndre ComandonLesley AndrewAndré Eduardo Biscaia LacerdaLesley IngramAndré EsserLesley K. BowkerAndré Geraldo BerezukLesley MachekaAndré Luiz Monezi AndradeLesley NewsonAndré NagalliLesley Pritchard-WiartAndré NovoLeslie J. HinyardAndré PierreLeslie KantorAndré Pimenta FreireLeslie Landaeta-DíazAndré PomboLeslie M. BeitschAndré RamalhoLeslie Ramos SalazarAndre Ricardo AlcardeLeszek ButowskiAndre RodackiLeszek KarlińskiAndré RodriguesLeszek SzablewskiAndre SilvaLeszek SzalewskiAndré SkupinLeszek WanatAndre WirriesLeta PilicAndrea AbateLeticia M. Márquez-MagañaAndrea AgugliaLetizia AppolloniAndrea AlexandreLetizia CasoAndrea AmerioLetizia GalassoAndrea Ascoli MarchettiLetizia MarsiliAndrea BaroncelliLetizia PerilloAndrea BassaniLetycja M. Sołoducho-PelcAndrea BazzoliLev EppelbaumAndrea BonassiLevi PerezAndrea BoscoLewis CarpenterAndrea BrambillaLewis HsuAndrea BuonsensoLewis J. MacGregorAndrea ButeraLewis Vibul HunAndrea C. F. FerreiraLexie SchererAndrea CassoniLeyla Angelica Sandoval-HamonAndrea CattaneoLeyla GamidullaevaAndrea CeciLi ChenAndrea Cecilia Serge RodríguezLi HeAndrea CeraseLi JinAndrea CitarellaLi LiAndrea Cívico ArizaLi TianAndrea Clemente PalmierLi YinAndrea ClementsLía Celina Méndez RodríguezAndrea CodaLia Cruz V. C. DamásioAndrea CoppolaLia DuarteAndrea CortegianiLia E. TsotsosAndrea Dall’AstaLia JiannineAndrea David Re CecconiLia VasconcelosAndrea De VitoLiana NagyAndrea Del GiudiceLiana PlesAndrea DemecoLiang HuAndrea Di CataldoLi-Ang LeeAndrea DietrichLiang-Chih ChangAndrea ErmolaoLiang-Ching ChenAndrea FarnhamLiang-Tseng KuoAndrea FontanaLian-Hua HuangAndrea FuscoLianmin ChenAndrea GabrioLianne UradaAndrea GalosiLiat HamamaAndrea GattoLibby MooreAndrea GiacomettiLibor AnsorgeAndrea GianniniLibor VelisekAndrea Gila-DíazLiborija Lugović-MihićAndrea Gonzalez-MontoroLi-Ching ChangAndrea GuazziniLícia Passos Dos Santos CruzAndrea GuridiLicia PensabeneAndrea HickersonLida FanAndrea KaifieLidewyde H. BerckmoesAndréa L. HobkirkLidia BorghiAndrea LazoLidia Chitimia-DoblerAndrea López ChaoLidia CzernickaAndrea Luise KoppitzLidia Da̧browskaAndrea LukácsLidia Gimena Domínguez-PárragaAndrea ManciniLidia LosadaAndrea ManfrinLidia MaynerAndrea Marais-PotgieterLidia MiroshnichenkoAndrea MarcinnòLidia PerencAndrea MontisciLidiane LimaAndrea NardiniLiezel HurterAndrea OkanovićLi-Fan LiuAndrea PacificiLifeng ZhouAndrea PalermoLígia Isoni AuadAndrea PammolliLigtvoet RudyAndrea Paola Rojas GilLih-Ming YiinAndrea Piano MortariLihua LiAndrea PicciLihui AnAndrea PisanelliLijian WangAndrea PuglieseLijuan FengAndrea R. TeufelbergerLijun WangAndrea RomaniLilah BesserAndrea Román-SánchezLili LeiAndrea RozemaLili O. HorváthAndrea SambriLilia González-CerónAndrea SansoneLilia LesykAndrea SantarelliLilian AzzopardiAndrea SchmeddingLilian Calderon-GarciduenasAndrea ScozzafavaLilian RigoAndrea SistiLiliana A. EnríquezAndrea SpinazzèLiliana CoriAndrea Szabó NagyLiliana CunhaAndrea ‘t MannetjeLiliana DumitracheAndrea TamburranoLiliana MâțăAndrea TickLiliana PitachoAndrea TittarelliLiliana RaduAndrea TortelliLiliana Szyszka-SommerfeldAndrea TrevisanLiliana Veronica DiaconescuAndrea VaniaLiliana Zuñiga VenegasAndrea VázquezLiliane Severino Da SilvaAndrea VergalloLiliya S. LogoydaAndrea VitaliLiliya ZiganshinaAndrea Vukic DugacLilla MakszinAndrea WerdeckerLilly AugustineAndreas ÄlgaLily House PetersAndreas BusjahnLilyan Vega-RamírezAndreas EderLimor GoldnerAndreas HohmannLin LinAndreas IhleLin ZhangAndreas JechowLina BegdacheAndreas KüttelLina Bunketorp KällAndreas KyrozisLina GubhajuAndreas OrlandLina HeierAndreas PesterLina JovarauskaiteAndreas SchulteLina SpirgienėAndreas SeidlerLina Stangvaltaite-MouhatAndreas TsatsarisLina ThorsAndreas VeglisLina YoussefAndreas Von Ameln-MayerhoferLinbo ZhaoAndreas WarnkeLinda BeckmanAndree HartantoLinda BriskmanAndreea AvasiloaieiLinda DirvenAndreea BrabeteLinda DynanAndreea Deciu RitivoiLinda G. KahnAndrei BorșaLinda GalloAndrei BuzatuLinda GoodfellowAndrei GrigorievLinda HagedornAndrei MitreaLinda J. Larson-PriorAndrei ShpakouLinda JamesAndreia CostaLinda JohanssonAndreia Martins RosaLinda Juel AhrenfeldtAndreia MóscaLinda K. KayeAndreia TeixeiraLinda Katherine JonesAndrej OpálekLinda LombiAndreja Brajša-ŽganecLinda MartinezAndrés AgudeloLinda PaternÒAndres Blanco OrtegaLinda RinehartAndrés E. MarcoletaLinda RykkjeAndres Fontalba-NavasLinda SangalliAndrés García-FlorianoLinda SaraivaAndrés García-GómezLinda SilkaAndrés Gené SampedroLindsay B. CareyAndrés Godoy-CumillafLindsay BottomsAndres Hernandez-MatamorosLindsay CrawfordAndrés J. PrietoLindsay E. YoungAndres Mendez-VazquezLindsay M. McCaryAndres Pandiella-DominiqueLindsay N. KohlerAndres Reinoso-CoboLindsay R. ArcurioAndrés Rodríguez-SeijoLindsay RosenfeldAndrés Sánchez-PradaLindsey GreviskesAndrés SantanaLindsey SmithAndressa Melina Becker Da SilvaLingling ZhangAndreu Comas-GarciaLingpeng ShanAndrew AlolaLingqiao KongAndrew B. CollinsLing-Tim WongAndrew BaileyLingwei MaAndrew BarnfieldLingxiao XieAndrew BéchardLingzhong GuoAndrew BowdleLinh N. HoangAndrew C. PoveyLinjing QiuAndrew CarswellLin-Ju KangAndrew DeanLinjun YaoAndrew EdgarLinlin XieAndrew F. StephensLinping ZhaoAndrew G. MarshallLinzhou LiangAndrew GrayLion ShahabAndrew HardingLiping SunAndrew HatchettLipsy ChopraAndrew HooymanLiron RozenkrantzAndrew James WoodLisa Alves GomesAndrew JenkinsLisa BauleoAndrew JinLisa ChristAndrew Julio BarrosLisa F. ShubitzAndrew KiraguLisa GibbsAndrew LaneLisa GoudmanAndrew LavenderLisa GrantAndrew LockeyLisa GuardoneAndrew M. SutherlandLisa HaleAndrew MartelLisa HendersonAndrew MayersLisa HuddlestoneAndrew MurphyLisa J. HardyAndrew N. PattonLisa KieselAndrew ObergLisa MartinsAndrew OmameLisa MillerAndrew P. LapointeLisa NewsonAndrew P. McGrathLisa Pescara-KovachAndrew PapadopoulosLisa QuadtAndrew PeplowLisa RobertsAndrew PikeLisa RubinAndrew PoveyLisa SanderinkAndrew S. HursthouseLisa ShookAndrew SchmidtLisa TangAndrew ShepherdLisa Tedesco TriccasAndrew ShimLisa TownsendAndrew SpringerLisa WirzAndrew StubblefieldLi-San HungAndrew TubbsLisandro RocoAndrew VakulinLisanne BergefurtAndrew WilliamsLisbeth Ehlert KnudsenAndrew WoodLise M. FrohnAndrey A. BelovLise Øen JonesAndrey K. SavtchenkoLiseane Padilha ThivesAndrey V. MaslovLisete FernandesAndrey VasilyevLisha MouAndries KosterLi-Shiue GauAndries MonyekiLishuang ShenAndrii KulikovLisiane Morelia Weide AcostaAndrzej AdamskiLisle HitesAndrzej CieslikLissa SpencerAndrzej CwynarLisse Angarita DavilaAndrzej CzamaraLiu YunAndrzej FalLiudmila I. Gerasimova-MeigalAndrzej GalbarczykLiudmila LeppikAndrzej HutorowiczLiudmila LiutskoAndrzej JanowskiLiudmila V. SpirinaAndrzej KacprzakLiudmyla V. GarmanchukAndrzej KasperskiLiuyi HaoAndrzej KlimczukLivia BurattaAndrzej KnapikLivia Ionela BobuAndrzej KulowskiLivia NastriAndrzej KurancLivia OttolenghiAndrzej LangeLivio SantosAndrzej LasotaLiviu GiurgiulescuAndrzej Marek ŚliwczyńskiLiviu SteierAndrzej MazurLiviu Stelian BeguAndrzej MichalskiLiviu-Gabriel BodeaAndrzej MiskiewiczLi-Yin ChienAndrzej MycLi-Yun SunAndrzej MyśliwiecLiyun YangAndrzej PiosikLiz SimonAndrzej PiotrowskiLiz TempleAndrzej PolańczykLiza JachensAndrzej PoszewieckiLiza LeeAndrzej RaszkowskiLizandra Garcia Lupi VergaraAndrzej SalataLizeth Guadalupe Parra-PérezAndrzej SilczukLizethly Cáceres JensenAndrzej ŚliwerskiLjiljana Markovic DenicAndrzej SlominskiLjubisa ŠaricAndrzej SokołowskiLloyd BalbuenaAndrzej SurdackiLloyd DavisAndrzej SzymkowiakLluís Costa-TutusausAndrzej TukiendorfLobo LouieAndy BeryLocati EmanuelaAndy SiddallLochan SinghAndy SiddawayLoes Van Rijn-Van GelderenAndy T. Y. LauLogan T. TrujilloAndy Wai Kan YeungLoick MenvielleAndy WilsonLois JamesAneta BacLokesh KumarAneta BrodziakLola NeufcourtAneta CegiełkaLon Van WinkleAneta NowakiewiczLoneke Blackman CarrAneta OlszewskaLong LiuAneta Ostróżka-CieślikLong SunAneta Ptak-ChmielewskaLong WeiAneta R. BorkowskaLong WuAneta ŚcieżyńskaLongina Chojnacka-OżgaAneta Słabuszewska-JóźwiakLonnie GoldenAneta SlodekLorann StallonesAneta Svozilíková KrakovskáLóránt Dénes DávidAneta WeresLorayne WoodfieldAneta WieczorekLore MetzAneta WłodarczykLoredana AntronicoAng LiLoredana BellantuonoÁngel Abós CatalánLoredana CerbaraÁngel Acevedo-DuqueLoredana Liliana HurjuiAngel Alberto Valdés-CuervoLoreen ThürmannAngel AsensioLorel BurnsAngel BarrasaLoren D. FastAngel Belzunegui-ErasoLorena Aguilar-ArnalÁngel BorregoLorena Azpiazu IzaguirreAngel Cordero AmpueroLorena Barra-BucareiÁngel Custodio Mingorance-EstradaLorena Durán-RiverollAngel De JuanasLorena Gutiérrez-HermosoAngel EnriqueLorena MarrodánAngel F. GonzálezLorena Tarriño-ConcejeroAngel LeeLorenz S. NeuwirthAngel M. KennedyLorenzo BevilacquaAngel Miramontes CarballadaLorenzo CarnevaleÁngel PaniaguaLorenzo FiaschiÁngel Prieto-FidalgoLorenzo GianquintieriAngel Romero ColladoLorenzo GittoÁngel Romero MartínezLorenzo LottiÁngel Rosa AlcázarLorenzo Mariano-JuárezÁngel Ruiz ZafraLorenzo MasiaÁngel Sabino Mirón SanguinoLorenzo PaglioneÁngel Torres-ToukoumidisLorenzo PasculliÁngel Vicario-MerinoLorenzo RumAngela AmmirabileLorenzo RusticoAngela AndreellaLorenzo TarsitaniAngela AntonucciLorenzo TonettiAngela ArmentoLorenzo VigentiniAngela BarbosaLorenzo ZamboniAngela Beale-TawfeeqLorenzo ZinoAngela C. GarinisLoreta Buksnyte–MarmieneAngela CartaLoreta StrumylaiteAngela CastoldiLoretta R. BrabinAngela Contreras CastroLori Baugh LittlejohnsAngela CostabileLori Elmore-StatonAngela DaddowLori ReynoldsAngela De LeonLori RothenbergAngela DewLori-Ann R. SacreyAngela Di PintoLoriena A. YancuraÁngela Engelmo MoricheLoris BuschAngela EvansLorna WalesAngela Georgia CaticLorraine BellÂngela GonçalvesLorraine PhillipsAngela HatteryLorraine ShackAngela JacquesLorraine TaylorAngela LombardiLorrene D. RitchieAngela LupattelliLothar FunkAngela M. Garcia SanchezLotta PalmbergAngela MertigLotta TikkanenAngela PilogalloLotte LindbergAngela Plaß-ChristlLotus S. BastAngela RepanoviciLouis De KokerAngela RidgelLouis De MesnardAngela RussoLouis Hugo FrancescuttiÁngela Sanjuán-QuilesLouis Kuoping ChaoAngela SantilioLouis MoustakasAngela SantosLouis R. NemzerAngela StufanoLouisa M. HolmesÁngeles BlancoLouise A. HorriganAngeli Christy YuLouise BroughAngelia Maleah Holland-WinklerLouise HopperAngélica Angélica Bulcão Portela-LindosoLouise HughesAngélica De AntonioLouise S. MackenzieAngelica GiulianiLouise TownsinAngelica Lo DucaLouise UnderdahlAngelica SteinLourdes AguilarAngelika Edyta CharkiewiczLourdes Canós-DarósAngelika IllgLourdes Cortes-DericksAngeliki PapanaLourdes Mateos-HernándezAngeliki SarellaLovro ŠtefanAngeline S. AndrewLoyda B. MéndezAngelisa FrascaLu WangAngelo AlbiniLua Perimal-LewisAngelo B. HookerLuana NosettiAngelo BarbatoLuana Tenorio LopesAngelo BasterisLuba Louise IvanovAngelo BellinviaĽubomíra LizákováAngelo CastelloĽubomíra TóthováAngelo DanteLubos RejfekAngelo DavalliLuc E. H. VanlinthoutAngelo DoglioniLuca ArdigòÂngelo JesusLuca BattistiAngelo SpenaLuca BruschiniAngelo V. VasiliadisLuca BusaniAngelos AlamanosLuca CavaggioniAngelos SikalidisLuca CegolonAngels NiñerolaLuca CernigliaAnh DangLuca D’AcciAnibal CoronelLuca Di GianfrancescoAníbal Maury-RamírezLuca DimuccioAniello MaieseLuca FalzoneAnika L. HinesLuca FerrariAnika LucasLuca FlesiaAnikó AngyalLuca FontanaAniko Khademi-VidraLuca FredianelliAnil GumberLuca GiacomelliAnil PoudelLuca Giovanni LocatelloAnirban MondalLuca Guglielmo PradottoAnish JantraniaLuca MacariniAnish ShivaramLuca MagistrelliAnisin AlexeiLuca MarinelliAnisor NedelcuLuca MenghiniAnita A. DaviesLuca NalboneAnita BregenzerLuca NarduzziAnita ButeraLuca OppiciAnita D’ApranoLuca Oscar Redaelli De ZinisAnita Gębska-KuczerowskaLuca PaduaAnita GibbsLuca PangrazziAnita Hurtig WennlöfLuca RagazzoniAnita Kolnhofer DerecskeiLuca RollèAnita L. SchillLuca RomagnoliAnita Lyssek-BorońLuca RossiniAnita MajchrowskaLuca RuggieroAnita MatićLuca RussoAnita MikolajczykLuca SalvatiAnita PadmanabhanunniLuca ScalfiAnita RaimondiLuca SimioneAnita Richert-KaźmierskaLuca SteardoAnita StasulaneLuca StingeniAnita VisserLuca TestarelliAnja BaumannLuca TricaricoAnja GeislerLuca ZampariniAnja Hagen OlafsenLucas A. SalasAnja HolwerdaLucas DelmonicoAnja KnöchelmannLucas Freitas De FreitasAnja KühnelLucas KohnkeAnja MenzelLucchese AlbertaAnja SchablonLucía Abenza-CanoAnja StajnkoLucia BírošováAnjana BairagiLucia BortoliniAnke Hanssen-DooseLucia BottiAnke HülsLucia BrajkovicAnkit ManglaLucia BrodosiAnkit SrivastavaLucia CarboniAnkita PunethaLucia Carmen TrincăAnkita ShuklaLucia CastelliAnkush KansalLucia ContiAnmol KumarLucia CraxìAnn BarrettLucia FazzoAnn D. DowkerLucia GalluzzoAnn E. SutherlandLucia GalvagniAnn FeeneyLucía Granados AlósAnn HemmingwayLucía Herrera TorresAnn JohnsonLucía I. LinaresAnn L. BaldwinLucia KendrováAnn L. GibsonLucia LombardiAnn L. HendrichLucia Mainero RoccaAnn LargeyLucia ManganaroAnn M. DozierLucia MangoneAnn M. ZoidisLucía MireteAnn SvenssonLucia MonacisAnn VandenbergLucia Peixoto-PinoAnn WebbLucia RatiuAnna Adamiok-OstrowskaLucía Rodríguez-ParadaAnna AdamusLucia RomoAnna Adamus-MatuszyńskaLucia RonconiAnna AftykaLucia SepeAnna AjdukLucia StančiakováAnna AnåkerLucia TabacuAnna AndruszkiewiczLucia Tomas-AragonesAnna Arnal-GómezLucía Tomás-AragonésAnna AtlanteLucia TonucciAnna Babicka-WirkusLucía Vera-HerreraAnna BagieńskaLucian SfîcăAnna BanikLucian TurcescuAnna BaraLuciana B. NucciAnna BartosiewiczLuciana CaenazzoAnna Barwińska-MałajowiczLuciana OliveiraAnna BednarekLuciana Rocha Barros GonçalvesAnna BirkováLuciano BubbicoAnna BłażewiczLuciano RomanoAnna BocchinoLuciano SilvaAnna Bocheńska-SkałeckaLucica BarbeşAnna BockLucie DrechselováAnna Bogusława Pilewska-KozakLucie LevesqueAnna BrzeckaLucie ViktorovaAnna BudzińskaLucie VnouckovaAnna CarcellerLuciele MinuzziAnna ChalkleyLucija VejmelkaAnna ChrobakLucila CamposAnna Cleta CroceLucile HouyelAnna CzarnaLucilia CardosoAnna DanielewiczLucinda BessaAnna Dewalska-OpitekLucio Camara E. SilvaAnna Di PintoLucio Di MatteoAnna Duda-MadejLucio NobileAnna DzielskaŁucja BieleninikAnna E. Van Der WathLucjian SajkowskiAnna FelnhoferLucrecia Carrera QuintanarAnna FijałkowskaLucrezia LopezAnna FlorowskaLucrezia MoggioAnna FranzLucy O. DiekmannAnna Gagat-MatułaLucy O’MalleyAnna Gajos-MichniewiczLucy PopovaAnna Gardocka-JałowiecLucy TroupAnna Garus-PakowskaLucyna OstrowskaAnna GórkaLucyna WitekAnna GrygierLudgleydson Ludgleydson Fernandes De AraujoAnna Grzywa-CelińskaLudivine MartinAnna HartonLudmila MüllerAnna HintonLudmila Silva GuimarãesAnna HošťálkováLudmila VodickovaAnna Irena SzymańskaLudmiła Zając-LamparskaAnna IrimiasLudo VanopdenboschAnna J. MilewskaLudovico PedullàAnna JackovaLudovina GalegoAnna Janas-NazeLudwig ApersAnna JędrzychowskaLudwig WagnerAnna Jupowicz-GinalskaLudwig WildtAnna Kablak-ZiembickaLuenda CharlesAnna KajdyLuenda E. CharlesAnna Karolina MatczukLufanna Ching-Han LaiAnna Klara MocovaLuigi Alberto PiniAnna KopiczkoLuigi AldieriAnna KostkaLuigi BavarescoAnna KozakLuigi BennardoAnna KoziorowskaLuigi CerboneAnna KwiatkowskaLuigi CipolloniAnna Lassia MeyerLuigi CorvoAnna Laura PalazzoLuigi De NapoliAnna LavisLuigi EspositoAnna ŁepeckaLuigi Isaia LeccaAnna LeśkówLuigi MontanoAnna LewandowskaLuigi RaritàAnna LipertLuigi SantacroceAnna Lisa AmodeoLuigi SenatoreAnna LocatelliLuigi TaraniAnna M. AddamoLuigi VetrugnoAnna MadraLuigi VimercatiAnna MainkaLuigia BruglieraAnna MajdaLuigino BarisanAnna Małgorzata KamińskaLuis A. RojasAnna Maria CostaLuis Albendín-GarcíaAnna Maria NuzzoLuis Alberto Gaitán-CepedaAnna Maria PaganoniLuis Alberto Rodríguez-PicónAnna Maria PaolettiLuis Andreu CaravacaAnna Maria RaspollLuis AnidoAnna Maria SantiagoLuís Carlos MatosAnna Maria SianiLuis Carlos Sandoval-HerazoAnna MarouškováLuis CarrascoAnna Marzec-GrządzielLuis CarúsAnna Matras-BolibokLuis Ceballos-LaitaAnna Michalak-StomaLuis ChambelAnna MichnikLuis Cobos-PucAnna MolterLuis Del Espino-DíazAnna MoniuszkoLuis Eduardo Ávila SilveiraAnna MosiołekLuis Eduardo Servín-GarcidueñasAnna MulassoLuis Enrique Roche SeruendoAnna Musz-PomorskaLuis Enrique Valdez JuárezAnna Myriam PerroneLuis Espejo-AntúnezAnna N. BerlinaLuis Espino-DíazAnna NowakLuis F. Gonzalez-CiccarelliAnna OgonowskaLuis Fernández-PozoAnna Ogonowska-SlodownikLuis Fernando ChavesAnna OgrodowczykLuís Giner-TarridaAnna OlczakLuis Gómez PérezAnna OnopiukLuis Guilherme Virgílio FernandesAnna PaczkowskaLuis Javier Cabeza RamírezAnna Paradowska-StolarzLuis Javier Capote-PérezAnna PartykaLuis Javier MárquezAnna PawlikLuis Llamas-AlonsoAnna PeaseLuis Llurda-AlmuzaraAnna PecchinendaLuis M. SilvaAnna Ploch-JankowskaLuis Manuel Cerdá SuárezAnna PodlasekLuís Manuel De Jesus LoureiroAnna Potulska-ChromikLuis Manuel Fernández-CachoAnna PsaroulakiLuis Manuel Martínez-ArandaAnna Q. YaffeeLuis Manuel Mota De SousaAnna Rask-AnderssenLuis Mariano EstebanAnna Rolewicz-KalińskaLuis Marqués MolíasAnna Rosa DonizzettiLuís MartinsAnna RosofskyLuis Miguel Cairós-VenturaAnna RóżańskaLuis Miguel Pedrero-EstebanAnna RozensztrauchLuis Miguel Romero-RodríguezAnna Rybarczyk-SzwajkowskaLuis Miguel Rondon GarciaAnna S. MakarovaLuis Miguel Sánchez LoyoAnna S. PeaseLuis MöckelAnna S. TzortziLuis MonteiroAnna Sączewska-PiotrowskaLuís MonteiroAnna Sánchez-CaballéLuis Muñoz-SaavedraAnna SaponeLuis N. CoriaAnna Schneider-KampLuís NovoAnna Ścisłowska-CzarneckaLuis Ortiz-GervasiAnna ShutalevaLuis Ortíz-JiménezAnna SikoraLuis PatinoAnna SpozLuís Pedro Ferreira RatoAnna StaniewskaLuís Pereira-da-SilvaAnna StaniszewskaLuís ProençaAnna StarshinovaLuis Rivera-GonzálezAnna StarzyńskaLuis RodrigoAnna StaszewskaLuis RodriguezAnna SurdackaLuís RoseiroAnna SykułaLuis SantosAnna Szczerba-TurekLuis SordoAnna Szóstek-MioduchowskaLuis SousaAnna TangLuis Taborda-BarataAnna TheocharidouLuís TinocaAnna TychmanowiczLuis Valero AguayoAnna Tyrańska-FobkeLuis Vicente Amador MuñozAnna Vila-MartíLuis VivancoAnna VillariniLuis Zavala-ArciniegaAnna Vittoria MattioliLuísa AiresAnna WagnerLuisa AnelliAnna WichowskaLuisa BergaminAnna WierzbickaLuisa BernardinelliAnna Winiarczyk-RaźniakLuisa Delgado LosadaAnna WłochLuisa GiariAnna Wziątek-StaśkoLuisa GorzaAnna YaffeeLuisa LimongelliAnna Zabłocka-KluczkaLuisa Losada PuenteAnna ZawadzkaLuisa QuevedoAnna ZellmaLuísa SoaresAnna ZwoździakLuisa WeinerAnna-Barbara SchlüerLuiz Augusto PerandiniAnnabel FernandesLuiz BrusacaAnnabelle DeramLuiz Carlos De AbreuAnnabelle NeallLuiz Carlos KreutzAnnalicia VaughanLuiz Eduardo Virgilio Da SilvaAnna-Liisa TammLuiz Felipe SilvaAnnalisa AnzaniLuiz Fernando De RezendeAnnalisa BargelliniLuiz Fernando Romanholo FerreiraAnnalisa CarlucciLuiz H. DuczmalAnnalisa GrandiLuiz NakamuraAnnalisa LevanteLuiz Otávio Murta JuniorAnnalisa MaraoneLuiz Pablo Cruz HervertAnnalisa PaceLuiz Ricardo De Almeida KigutiAnnalisa ZaccaroniLuiza Mesesan SchmitzAnnamaria ManciniLuiza OchnioAnnamária PallagLuiza OssowskaAnnamil Alvarez TrottaLuk JimAnnarita ColasanteLuka NovačkoAnnarita SignorielloLukas Jyuhn-Hsiarn LeeAnne Ahrendt BjerregaardLukas KaesmannAnne AndersonLukáš NajdekrAnne Beate ReinertsenLukas PeterAnne BertholdLukas SveikataAnne Christine WoolridgeŁukasz BalwickiAnne ConanLukasz BojkowskiAnne Day LeongŁukasz BorekAnne EkdahlŁukasz BorowskiAnne EvensŁukasz ChrobokAnne Faber HansenŁukasz CichockiAnne FalcouŁukasz GrussAnne HartmanLukasz HenszelAnné Hendrik VerhoefŁukasz KiszkielAnne JonesŁukasz KrystAnne JörnsŁukasz KurowskiAnne KonkleŁukasz ŁapajAnne LichtenwalnerŁukasz MamicaAnne Marie Mork Rokstad‪Łukasz MokrosAnne MerrelaarŁukasz OleksyAnne Namatsi LutomiaŁukasz PaczesnyAnne NobelsŁukasz PajchelAnne OppligerŁukasz PałkaAnne RoskenŁukasz PeszekAnne SimmenrothŁukasz PuśleckiAnne SwainŁukasz RypiczAnne TaufenŁukasz SatołaAnne WilsonŁukasz SikorskiAnneclaire De RoosŁukasz ŚwiątekAnnegret Biegel-EnglerŁukasz SzczukowskiAnnekatrin SteinhoffŁukasz SzeleszczukAnne-Laure TardyŁukasz TomczykAnnelien DuitsŁukasz WirkusAnnelies KeymeulenŁukasz WróblewskiAnnelise J. BlombergŁukasz WysoczańskiAnne-Lise K. VelezŁukasz ZapałaAnne-Louise PonsonbyŁukasz ZedlerAnne-Maria LaukkanenLuke BalcombeAnnemarie BennettLuke ConnellyAnne-Marie Perchec MerienLuke CurtisAnnemiek Van WegbergLuke DonovanAnnett Dorner-ReiselLuke HogarthAnnette B. PfahlbergLuke J. NeyAnnette MengLuke PrendergastAnnette PfahlbergLuke SienaAnnibale BiggeriLuke WoonAnnica AlmståhlLuke Y. ChenAnnica BackmanLum Peng LimAnnick Parent-LamarcheLuma AkilAnnie GendronLuminița IonescuAnnie George-PuskarLuminita NicolescuAnnie HeiderscheitLuminita OanceaAnnika AnderssonLuminita PaduraruAnnika Helgadóttir DavidsenLuminita ParvAnnis Lai-Chu FungLuna CarpinelliAnnisa TriyantiLuo LiAnnmarie ChizewskiLuo SitongAnnsofie AdolfssonLuri PeriAnn-Sofie LindbergLut TamamAnnukka VainioLuuk Van KempenAnnunziata RomeoLuxana Reynaga-OrnelasAnoop JainLuxon NhamoAnoop K. ShuklaLuyu XieAnowara BegumLuz Huntington-MoskosAnselm BräuerLuz Kelly AnzolaAnshika JainLuz Maria Sánchez-RomeroAnson Chui Yan TangLuz Reynales-ShigematsuAnthony B. BuccitelliLuzaan HamiltonAnthony E. KiszewskiLuz-María Martín-DelgadoAnthony E. SparklingLy Sy Phu NguyenAnthony HerbertLydia E. DevenneyAnthony HuangLydia FrydaAnthony KondrackiLydia GanAnthony L. KovacLydia Giménez-LlortAnthony LynnLydia HorneAnthony M. BeanLydia Liu YangAnthony MawsonLydia RepkeAnthony NataleLydie Huché-ThélierAnthony StrittmatterLyn FrancisAnthony SuraceLynda B. RansdellAntía González PereiraLynissa StokesAntigoni FakaLynn Clark CallisterAntigoni ZafirakouLynn M. GrattanAntje Büttner-TeleagăLynn N. IbekweAntoaneta EneLynn UlatowskiAntoine ChatrenetLynne GauthierAntoine LangeardLynne HampsonAntoine RimbertLynne HaneyAntoine TalarminLynne M. Z. LafaveAnton GoncharovLynsey K. RomoAnton IsaacsLynsey R. HarperAnton KasprzhitskiiLyusyena KirakosyanAnton MurzinM. Carmen Terol CanteroAnton R. KiselevM. Gloria Gallego-JiménezAnton TerasmaaM. Idoia Ugarte GurrutxagaAntonella AgodiM. Inmaculada López-NúñezAntonella ArghittuM. J. Ruzmyn VilcassimAntonella BevilacquaM. Jean KellerAntonella CostaM. KarimAntonella GaglianoM. Pilar Munuera GómezAntonella LisaM. S. ZobaerAntonella LopezM. Shamim KaiserAntonella OliveroM. Trinidad Sánchez-NúñezAntonella RomaniniM. Virtudes Alba-FernándezAntoni BorrásMª De Los Ángeles Cardero-DuránAntoni WontorczykMa De Los Ángeles Merino GodoyAntonia KaltsatouMª Lucía MoránAntonia MarsdenMaansi Bansal-TraversAntonia PelegrínMaarten C. BoslandAntonia R. G. AlvarezMaayan ShachamAntonia TerpouMacarena Gomez-LiraAntonietta IvonaMachiko R. TomitaAntonietta LiottiMaciej DobrzańskiAntonija TadinMaciej FilipiakAntonina GiammancoMaciej J. NowakAntonina N. MutoroMaciej JedlińskiAntonina SidotiMaciej JozwikAntonino CanaleMaciej KowalewskiAntonino CatalanoMaciej PawlakAntonino Di BellaMaciej PolakAntonino GalatiMaciej RomaniukAntonino ManiaciMaciej RyczkowskiAntonino SpanuMaciej SalagierskiAntonino TuttolomondoMaciej SerowaniecAntonino VallesiMaciej TomczakAntónio AbreuMaciej TysarowskiAntonio Alberto Rodríguez SousaMack ShelleyAntonio Alvarez-SousaMadalina-Gabriela IliescuAntonio AntúnezMaddalena CannitoAntonio ApicellaMadeline SprajcerAntonio AquinoMadelon L. FinkelAntonio AzaraMadhavi GaviniAntonio BaezaMadhavi Thombre KulkarniAntonio BaldassarreMadhu AtterayaAntonio BalderasMadhu Sudhan AtterayaAntonio BenedettiMadhuvanthi VijayanAntónio Bento-GonçalvesMadjda BellamriAntonio Bernardo SánchezMaegan CalvertAntonio BiondiMagaly Aceves-MartinsAntonio CaciolliMagda Guimarães De Araújo FariaAntónio CaleiroMagdalena Brzozowska-WośAntonio CañabateMagdalena Chottova DvorakovaAntonio Cano-OrellanaMagdalena Czlapka-MatyasikAntonio Carlos De Oliveira JúniorMagdalena Eriksson DomellöfAntonio Carlos GalhanoMagdalena GantnerAntonio Casas-BarragánMagdalena Gizińska-GórnaAntonio CicchellaMagdalena GórnickaAntonio CobeloMagdalena GuzyAntonio Conde-SánchezMagdalena Hagner-DerengowskaAntonio Córdoba FernándezMagdalena JancAntonio CoronatoMagdalena KorżyńskaAntonio CorteseMagdalena KostrzewaAntónio CuradoMagdalena KowalówkaAntonio De PinMagdalena Krajewska-WłodarczykAntonio DiglioMagdalena KusnierzAntónio Duarte SantosMagdalena LeszkoAntonio E. PontiroliMagdalena Lukaszewska-KuskaAntonio FernandesMagdalena M. StussAntonio Fernández HernándezMagdalena Mackiewicz-MilewskaAntonio Fernández-CastilloMagdalena Mosionek-SchwedaAntonio Fernández-MartínezMagdalena MyszuraAntonio Fernández-ParraMagdalena Paplińska-GorycaAntonio FranconeMagdalena PodbielskaAntónio Freire DiogoMagdalena Polak-ŚliwińskaAntonio Garrido-FernándezMagdalena RaczyńskaAntonio Giuliano ZippoMagdalena RaftowiczAntonio GonzalezMagdalena Ramos Navas-ParejoAntonio Granero-GallegosMagdalena RatajczakAntonio GuaitaMagdalena RyżakAntonio Hernández FernándezMagdalena SkarzynskaAntonio IovanellaMagdalena Śmiglak-KrajewskaAntonio J. OrtizMagdalena SobieskaAntonio Jesús Molina FernándezMagdalena StaszczakAntonio Jesús Ramos-MorcilloMagdalena Sycinska-DziarnowskaAntonio José CarpioMagdalena Sylwia KamińskaAntonio José Sánchez-GuarnidoMagdalena SzymańskaAntonio JuniorMagdalena Wójcik-JurkiewiczAntonio LazzarinoMagdalena WrzesińskaAntonio LeddaMagdi HabachiAntonio León García-IzquierdoMagno Conceição Das MercêsAntonio LeoneMagnolia Ngcobo-SitholeAntonio LloretMagnus AkerstromAntonio Lozano GarcíaMagnus HeitzlerAntónio Luís AmaralMagnus HultinAntónio Luís CrespiMagnus MogliaAntonio Luís Martinez-PujalteMagnus SjögrenAntonio Luque De La RosaMagnus SvartengrenAntónio M. G. BaptistaMaha A. ElbadryAntonio MaccioMaharaj SinghAntonio ManentiMahdi SeyedsalehiAntonio Manuel Amor GonzálezMaheen M. AdamsonAntonio Mario ScanuMaher AlmasriAntonio MarquesMaher Georges ElmashharaAntonio Martínez RayaMahesh KaushikAntonio Martínez-SabaterMahmoud F. SeleimanAntónio MateusMahmoud KandeelAntonio Miguel Linares-LujánMahmoud SayedAntónio Miguel MonteiroMahnaz RamezanpourAntonio Miñán EspigaresMahomet NjoyaAntonio MincioneMahsa DabaghAntonio MirijelloMahsa FaizrahnemoonAntonio MontanesMahyar Habibi RadAntonio Montero SeoaneMai MatsumotoAntonio NarzisiMai Salah El Din Mohamed HelmyAntonio NesticòMaia PonsonnetAntónio NogueiraMaidul IslamAntonio PacittoMaija PuhkaAntonio Palacios-GarciaMaike TietjensAntonio Palazón-BruMaikel RosabalAntonio Partal UreñaMaiko SakamotoAntonio Pedrero GonzálezMais JafariAntonio R. Hidalgo-MuñozMaitane PicazaAntonio Rafael Peña-SánchezMaite De BlasAntonio Ramos MartinezMaitreyee MukherjeeAntonio Roberto ZamunérMaja Cigrovski BerkovićAntonio RodanMaja Ivana Smodiš ŠkerlAntonio Rodríguez-MartínezMaja MatulicAntonio RomanelliMaja MeskoAntonio RomanoMaja RadziemskaAntonio SánchezMaja RožmanAntonio Sarría SantameraMaja TurnsekAntonio ScaranoMajed AlamriAntonio SchiattarellaMajed RefaaiAntonio Segura-FragosoMajeed AbdulAntonio Seijas-MaciasMajid KoozehchianAntonio Simone LaganàMajid MomenyAntonio SiniscalchiMakayla CordozaAntonio SpecchiaMaki Tei-TominagaAntonio TacconeMaki UmedaAntonio TessitoreMakiko KouchiAntônio Thomaz Da Matta-MachadoMakoto AokiAntonio TintoriMakoto EndoAntonio Torralba-BurrialMakoto HashimotoAntonio Víctor Martín‐GarcíaMakoto HiraoAntonio Vidal-InferMakoto KomiyaAntonio ZayasMakoto MorinagaAntonio ZumelzuMakoto ShodaAntonio-Jesús Marín-PazMaksymilian MądzielAntonios E. KoutelidakisMalcolm JoyceAntonios TasoglouMalcolm KingAntonis KambasMalcolm KooAntonis StamatakisMalcolm RileyAntoon De SchryverMalek AbduljaberAnts-Hannes ViiraMalek BatalAntti KämppiMalek El MuayedAntti MeroMalene Flensborg DamholdtAntti RätyMałgorzata Balwicka-SzczyrbaAnttonio GiordanoMałgorzata BasińskaAnu ChackoMałgorzata Biniak-PierógAnu MolariusMałgorzata BronikowskaAnu SirolaMałgorzata Buksińska-LisikAnubhav Pratap SinghMalgorzata BurekAnugrah Sabdono SudarsonoMałgorzata ĆwiekAnuj TiwariMalgorzata CyganskaAnuli NjokuMalgorzata DziembalaAnunciação VenturaMałgorzata Ewa DrywieńAnuradha SinghMałgorzata GolemanAnurag SatpathyMalgorzata GrembeckaAnusha PenumartiMalgorzata GrzesiakAnushree PriyadarshiniMałgorzata I. MichalczykAnutthaman ParthasarathyMałgorzata KarwowskaAnya SmithMalgorzata KosteckaAnzhelika VoroshilovaMałgorzata KowalAo LiMalgorzata KowalskaAoife M. DuffMałgorzata KrówczyńskaAparajita BanerjeeMałgorzata LehnerAparicio-Ugarriza RaquelMałgorzata Lesińska-SawickaAparna ShindeMałgorzata LewandowskaApoena RibeiroMalgorzata MaciukiewiczApolinaras ZaborskisMałgorzata MacudaApostolos BeloukasMałgorzata Małodobra-MazurApostolos BozikasMałgorzata Mazurek-MocholApostolos LagariasMałgorzata MizgierApostolos ZiakopoulosMałgorzata MoszakApril A. KedrowiczMałgorzata NagórskaApril Chiung-Tao ShenMałgorzata Obara-GołębiowskaApurva PatangeMalgorzata PankowskaAraceli Galiano-CoronilMałgorzata PasekAraceli Ortiz RubioMałgorzata Pawlaczyk-ŁuszczyńskaAracely Serrano-MedinaMałgorzata PihutAram MegighianMałgorzata Plechawska-WójcikArantzazu Eiguren-FernandezMałgorzata Polz-DacewiczArash DarafshehMałgorzata RutkowskaArash DerakhshanMałgorzata Schlegel-ZawadzkaArata KatayamaMałgorzata SobieszczańskaAravamudhan AjaMałgorzata SobolArdalan ShariatMałgorzata StefaniakArdesheer TalatiMałgorzata StefańskaArdjouman DiabateMałgorzata SzkupAremis VillalobosMałgorzata SztubeckaArgyris ToubekisMałgorzata Tatarczak-MichalewskaAriadna Zybek-KocikMałgorzata WójcikArianna AntonucciMalgorzata WorwagArianna DondiMałgorzata WosiekArianna D’UliziaMalgorzata WoznickaArianna ScalaMalgorzata Zakłos-SzydaArianna SmerilliMałgorzata ZiarnoAriel Orlei MichaloskiMalik AydinAriel Soares TelesMalik SallamArif RezaMalindu SandanayakeArifin Budiman NugrahaMalka MargalitAriful IslamMallikharjun KoriviArifur RahmanMallory MarshallArindam SinharoyMalte JahnAristeidis H. ZibisMalte SchwingerAristeidis PanagiotouMalte Von SzombathelyAristides I. FerreiraMalvin N. JanalAristidis TsatsakisMamtha BallaAristomenis ExadaktylosMan Tat LeiAritz AdinMan Ying AmandaAriuntuul GaridkhuuManal M. AlzghoulArja RimpelaManan RoyArjan Bastiaan Van AsMandar KhanalArjan VissinkMandeep Singh JassalArjun SenguptaMandu EkpenyongArka DattaMandy Kirkham KingArkadiusz ArtyszakMandy StahreArkadiusz ChruscielMandy TruongArkadiusz JanuszewskiMandy VogelArkadiusz LubasMandy YapArkadiusz M. KowalskiManel Valcarce-TorrenteArkadiusz NędzarekManela GlarcherArkadiusz PrzybyszManfred KöhlerArkadiusz StanulaManfred Man-Fat WuArkadiusz ŚwiadekManfred NeubergerArkadiusz UrbanekManfred SagerArkadiusz ZurawskiManfred SchmittArkady SerikovManhai LongArkaitz CastañedaMani Rouhi RadArkalgud RamaprasadMani SharifiArkers Kwan Ching WongManik SharmaArlene MannionManinder KaurArlener D. TurnerManiselvan KuppusamyArline BronzaftManish KumarArmand Grau-MartínManisha WitmansArmand KasztelanManivel RengasamyArmanda PereiraManja Kitek KuzmanArmando AliuManjusha ChintalapatiArmando Coca RojoManlio SantilliArmando Enrique Gonzalez-StuartMannava V. K. SivakumarArmando Manuel De Mendonça RaimundoManny MathuthuArmando Perez-CuetoManohar Prasad BhandariArmando Silva-AfonsoManoj A. BiniwaleArmands AuziņšManoj GovindarajuluArmel Zacharie Ekoa BessaManoj PardasaniArmida Sánchez-EscalanteManoj SharmaArmigon AkhmedovManoli Garcia-de-la-HeraArmin KrausManolis AdamakisArmin RazmjooManon BosArmin TarrahManos J. DassenakisArminda GonçalvesMansi SharmaArnal JosepMansour GhasemikaramArnaldina SampaioManuel Albornoz-CabelloArnaud PagesManuel Arias-MontielArne GüllichManuel AurelianoArne Johan NorheimManuel BelliArne LowdenManuel Campos-GarcíaArne Martin JakobsenManuel Canovas-VidalArnon BentovimManuel Castro-SanchezArnulfo Hernan Nava-ZavalaManuel Contreras-LópezArnulfo Ramos-JiménezManuel De La CruzArpan AcharyaManuel Durán-PovedaArpita BasuManuel GámezArrigo CiceroManuel García RamírezArryn A. GuyManuel GazquezArsenio PaezManuel Herrero-MontesArshad Arjunan NairManuel IsornaArshad JavedManuel J. RuizArtan HoxhaManuel Jesús Cardoso PulidoArtan HysaManuel Joaquim Sabença FelicianoArtem MelmanManuel Joaquín De Nova-GarcíaArtemi CerdaManuel Jonas RichterArtemissia-Phoebe NifliManuel José LopesArthur BlaserManuel José Rodríguez OrtegaArthur DavidManuel LinaresArthur FerreiraManuel Llorca-JañaArthur I. PetrovManuel Luís Au-Yong OliveiraArthur L. FrankManuel Marques FerreiraArtiom VolkovManuel MatosArto ReimanManuel Monfort-PañegoArtur BanachManuel MoyaArtur C. JaschkeManuel Nieto-DiazArtur CoutinhoManuel Pabón-CarrascoArtur GiełdońManuel Pardo-RiosArtur GłuchowskiManuel PatarroyoArtur GonçalvesManuel PeralboArtur HołujManuel PereaArtur PasternakManuel PereiraArtur StolarczykManuel PestanaArtur StruzikManuel RequenaArtur StrzeleckiManuel Rich-RuizArtur SzkopManuel Rodriguez PerezArtūras KilikevičiusManuel Rodríguez-HuguetArturo CasadoManuel Romero SaldañaArturo CesaroManuel Vargas-VargasArturo DurazoManuela BarretoArturo Gonzalez-GilManuela BombanaArturo Hardisson De La TorreManuela De SarioArturo J. Fernández-GonzálezManuela DeiddaArturo López CastelManuela MauceriArturo NuaraManuela OliveiraArturo SierraManuela SilvaArturo-Perdomo DavidManuela Vieira Da SilvaArul ChandranMao-Meng TiaoArul EarnestMaosen WangArunava GhoshMapuana C. K. AntonioArunkumar JayakumarMaquines Odhiambo SeweArve E. AsbjørnsenMar BennasarArwa MustafaMara LombardiArye L. HillmanMaral BabapourArzu BeklenMarc ArgilésAsadur RahmanMarc AuriacombeAsal Kamani FardMarc BeaumontAsami MatsunagaMarc DreyerAsanka BulathwattaMarc HilhorstAsbestosChiara AiroldiMarc JacquinetAsbjørn ÅrøenMarc L. De BuyzereAscânio AraújoMarc LochbaumAscanio SirignanoMarc Moreno-AriñoAscensión Doñate-MartínezMarc Perez-BurrielAscensión Palomares RuizMarc SaezAsel RyskulovaMarc SchmitterAsgeir MamenMarc SturgillAsha N. ShenoiMarc TasséAshad KabirMarc WeinsteinAsharani Pezhummoottil VasudevanMarc YesteAshim GuptaMarcel BassachsAshiqur RahmanMarcel F. KunrathAshlee CunsoloMarcel HanischAshlee CurtisMarcela Carina Rua VaraAshleigh HomerMarcela Martín-Del-CampoAshley Brooks-RussellMarcela PejchalovaAshley Elizabeth MullerMarcela Sefora NemteanuAshley EmeryMarcela Tamayo-OrtizAshley FussMarcela VizcarraAshley HazelMarcelino Pérez-BermejoAshley L. FowlerMarcell CsanádiAshley WeinbergMarcella MandaniciAshly WestrickMarcella TambuscioAshok KumarMarcello AlinoviAshok PatilMarcello BonfèAshtyn ArealMarcello CasertanoAshutosh Dhar DwivediMarcello CherchiAsier Santas TorresMarcello MocciaAsif IqbalMarcello PassarelliAsim KichlooMarcello RubertiAsim Kumar BepariMarcello SariniAsmaa Mohammed HassanMarcello VeigaAsmi ChakrabortyMarcelo Andreetta CorralAspasia GoulaMarcelo De Paula CorrêaAsrar AhmadMarcelo FerreiraAsrat AmnieMarcelo GitiranaAsri MaharaniMarcelo S. BiudesAssaf AnyambaMarcelo Sacardi BiudesAssed N. HaddadMarcelo Silva SthelAssia RiccioniMarcelo Sousa SilvaAssumpta EnsenyatMarcia SchererAsta ValackienėMarcial Velasco GarridoAsterios KampourasMarcin BogdańskiAsterios PatsiaourasMarcin BorowiczAstrid FinkMarcin BryłaAsunción CarmonaMarcin BrzezickiAta TaraMarcin ButlewskiAteequr RahmanMarcin CzopAtef MohanyMarcin FeltynowskiAthanasia PatakaMarcin GierczykAthanasia PrintzaMarcin JaneckiAthanasia XirogianniMarcin JanuszAthanasios DalamitrosMarcin KluczekAthanasios DamialisMarcin KurowskiAthanasios K. PoulopoulosMarcin MaciejczykAthanasios KampasMarcin OlkiewiczAthanasios KoutrasMarcin PelkaAthanasios KungolosMarcin PietranikAthanasios P. FassasMarcin PotrykusAthanasios PapakyriakouMarcin RelichAthanasios T. GalanisMarcin RzeszutekAthanasios TselebisMarcin SobotaAthanassios AndroutsosMarcin StraburzynskiAthanassios K. GiatropoulosMarcin WekwejtAthena YiannakouMarcin WełnickiAthina EconomouMarcin WłodarczykAthina PatelarouMarcin ZarowskiAthos TrecrociMárcio De Carvalho FormigaAtieh PoushnehMarcio EisencraftAtin AdhikariMárcio Falcão Santos BarrosoAtsuko ArakiMárcio OliveiraAtsuko NakayamaMárcio Poletti LauriniAtsushi K. KonoMarco A. RamosAtsushi KawaguchiMarco Alexandre Da Silva BatistaAtsushi KohyamaMarco Alfonso PerroneAtsuyuki SorimachiMarco AllinoviAttà NegriMarco AnnunziataAttayeb MohsenMarco Antonio Loza-MejíaAttila BorsosMarco BardusAttila DénesMarco BatistaAttila GereMarco BennardiAttila GulyásMarco BenvenutoAttila HertelendyMarco BiagiAttilio Fiorini MorosiniMarco Bruno Luigi RocchiAtul Kumar PandeyMarco CantonatiAtul Singh RathoreMarco CarmignaniAtul SinghalMarco Carnevale MiinoAubrey ArainMarco ClariAubrey JonesMarco De AngelisAubrey L. GalushaMarco Del RiccioAudrey J. MurrellMarco DemichelisAudrey LongMarco DettoriAudrey Opoku-AcheampongMarco Di NicolaAugust WrotekMarco Di SarnoAugustín StarečekMarco DucaAugustine W. KangMarco E. M. PelusoAugusto Gil Brites Andrade PascoalMarco EneaAugusto PalombiniMarco EspositoAung Kyaw ThuMarco FabbriAura RusuMarco FerrariÀurea Cartanyà-HuesoMarco G. MarianiAurea Ramírez-JiménezMarco GervasiAurelia Beatrice AbalaseiMarco GuicciardiAurelian-Petruș PlopeanuMarco GuzzoAurelie GoncalvesMarco HaidAurélie Van HoyeMarco HelbichAurelien CorneauMarco JoseAurélien DeniaudMarco La MarraAurelio ArnedilloMarco LezzeriniAurora B. LeMarco LombardiAurora Castro-MendezMarco MascarellaAurora MadariagaMarco MascittiAurora Polo RodríguezMarco Matteo CicconeAurora VassanelliMarco MilanesioAušra KazlauskienėMarco NunesAušra MarcinkevičienėMarco ParoliniAutumn MarshallMarco PereiraAviad Tur-SinaiMarco PerroneAvik ChatterjeeMarco PonzettiAvinash AujayebMarco PortelliAvinash KadamMarco PotaAvinash KolliparaMarco RaceAvinash R. PatwardhanMarco RoccettiAvril JohnstoneMarco SapienzaAyako EdahiroMarco SegattoAyako HiyoshiMarco SpadaAyako NakaneMarco Stefano DemarchiAyako SuzukiMarco SusinoAyanka WijayawardenaMarco TatulloAyça Şentop DümenMarco TerreniAydin GülsesMarco TjioeAyesha DilrukshiMarco TogniAygul KissalMarco TomiettoAyman AhmedMarco Trabucco AurilioAymen AlianMarco TreguaAyodeji IyandaMarco UchidaAyokunle OlanipekunMarco VelicognaAyon ChakrabortyMarco VeraniAyse Giz GulnermanMarco VincetiAyse KaranciMarco ZamoraAyse OzbilMarcos A. MatosAysegul AksanMarcos Fernando Xisto Braga CavalcantiAyyoob SharifiMarcos García-LópezAzad BhuiyanMarcos Gómez-PuertaAzad HaiderMarcos Jesús Iglesias MartínezAzhar AbbasMarcos José RenniAziz BachaMarcos Maynar MariñoAziz EssadekMarcos Mecías-CalvoAzmawati NawiMarcos Pazos-CouseloAzucena Arias-CorreaMarcos Rodríguez MillánAzucena Hernández MartínMarcos Vinicius Bueno De MoraisAzucena Pedraz-MarcosMarcus D’SouzaAzzurra MassimiMarcus KaulAzzurra StefanucciMarcus SchiltenwolfB. Gonçalves Galdino Da CostaMarcus SchreinerB. N. Pavan KumarMarderos SayeghB. P. ChandraMaree L. InderBabak AlizadehMarek AngowskiBabak ChehroudiMarek BiziukBabak E. SaraviMarek FolBabett RamsauerMarek FraněkBabette BaisMarek GaworskiBach Q. HoMarek GruszczyńskiBach TranMarek JasinskiBachir KassasMarek JudaBaciliza Quintero SalazarMarek KonefałBadar Nadeem AshrafMarek KonopBaek Seung-HoonMarek KrzystanekBahareh MansoorianMarek LapkaBahareh OryaniMarek MotykaBahi TakkoucheMarek OchowiakBahia HakikiMarek PetrášBahijja Raimi-AbrahamMarek PopowczakBahil GhanimMarek RejmanBaik-Ho KimMarek SkrzypskiBairbre TiernanMarek SłońskiBalachandrudu NarapusettyMarek SzajtBalaji SadhasivamMarek SzelągowskiBalamurugan RamatchandirinMarek TelejkoBalan RathakrishnanMarek VochozkaBalaraman RajanMaren GoeckenjanBalázs ÁdámMaren WeissBaltasar González-AntaMaresca Attard PizzutoBan MajeedMargalida Coll-AndreuBang-Gee HsuMargaret FitchBanvolgyi AndrasMargaret HeffernanBao TeMargaret HodginsBaojie HeMargaret MacAndrewBaptiste LignierMargaret SherburnBarbara AndersonMargaret SouzaBárbara AngelMargaret ThomasBárbara Badanta RomeroMargaret WallenBarbara BarabaschiMargareta LiljaBarbara BaranowskaMargarete C. KulikBarbara BellowsMargareth Santos ZanchettaBarbara BezerraMargaretha LarssonBarbara BorusiakMargarida Pires SimõesBarbara CalabreseMargarida VieiraBarbara ErminiMargarita FernandezBarbara FearsMargarita Ortiz-TalloBarbara GołębiewskaMargarita PanayiotouBarbara GordonMargarita Pino-JusteBarbara GronostajskaMargaux MulatierBarbara GuidiMargherita EufemiBarbara J. HoogenboomMargherita FerranteBarbara JankowiakMargherita MaranesiBarbara JarząbMargherita Micheletti CremascoBarbara JasiewiczMargherita Paola PotoBarbara JuenMargherita RampioniBarbara KosMargherita TumedeiBarbara Krochmal MarczakMargherita ZitoBarbara ŁapińskaMargie Hodges ShawBarbara MazurMargie PedenBarbara MerrillMargit DrapalBarbara MognettiMargo TurnbullBarbara MunzMargot RawsthorneBarbara NazareMargot W. M. De WaalBarbara PabianMargret Sibylle EngelBarbara PieczykolanMargrethe F. Horlyck-RomanovskyBarbara PieperMari AguileraBarbara PospiesznaMari EggersBarbara RosołekMari Lloyd-WilliamsBarbara RuffinoMaría A. Pérez-HerreroBarbara Ruszkowska-CiastekMaria Adela GrandoBarbara SchneiderMaría Adelaida Álvarez-SerranoBarbara SchumannMaria Alba SorollaBarbara SeebacherMaria Alejandra Vidales BarrigueteBarbara Siuta-TokarskaMaria Alves BarbosaBarbara ŚlusarskaMaria Alzira Pimenta DinisBarbara SocasMaria Amparo OliverBarbara SymanowiczMariá Andrée López GómezBarbara SzulczewskaMaria AndronicBarbara SzymoniukMaria Anele RomeoBarbara ToraMaria Angela PellegrinoBarbara TrinconeMaria Angela Zaccarelli-MarinoBarbara VeselkaMaria Angeles Caballero-MoraBarbara WessnerMaria Angeles Cuadrado-CenzualBarbara WhiteMaría Angeles García-Carpintero MuñozBarbara WięckowskaMaria Angeles Martinez RuizBarbara WoronkoMaria Ángeles Pérez MorenteBarbara YawnMaria Angelica Mejia CaceresBarbora DužíMaria Anna ChoukriBarbora PiknovaMaria Anna DonatiBarend W. FlorijnMaria Antonia De FrancescoBarkha YadavMaría Antonia OvalleBarnes-Josiah DeboraMaría Antonia Parra-RizoBarraza Villarreal AlbinoMaria António CastroBarry BormanMaría Aránzazu RevueltaBarry GerberMaria ArmaouBart De GeestMaria ArnedoBart HattinkMaría Asunción Lara CantúBart PijlsMaría Auxiliadora Robles‐BelloBart VisserMaria BagattiniBart VogelaarMaria Begona SanzBartholomäus WissmathMaría Belén San Pedro VeledoBartłomiej Henryk NoszczykMaria Belen YanottiBartłomiej JefmańskiMaria BenllochBartłomiej MiszukMaria Bostenaru DanBartłomiej NiespodzińskiMaría C. FuentesBartłomiej PerekMaria C. T. CangussúBartłomiej ZagrodnyMaria CamposBartosz BartniczakMaría Campo-ValeraBartosz DalewskiMaría Candelaria Barrios GonzálezBartosz JaweckiMaria Carmela GrocciaBartosz MałkiewiczMaría Carmen RicoyBartosz SawikMaría CarrataláBartosz SzulczyńskiMaría Carrillo-DíazBartosz WielgomasMaria CasagrandeBasabi ChakrabortyMaria Catena SilvestriBasanta Kumar BiswalMaria CerretaBasanta PaudelMaria Chiara MeucciBasema SaddikMaria Chiara TorricelliBashayer R. Al-MubarakMaria ChiesaBashir SalahMaria ChristouBasilio PueoMaria CiccarelliBassam AyoubMaria Concepción UnanueBassam ElgamoudiMaria ContaldoBassam HusseinMaria CooperBatista PaulaMaria Coral Falco PerezBau­meis­ter Ste­fanMaria Cristina AngeliciBayodé Roméo AdégbitèMaria Cristina BudaniBeat SchäfferMaría Cristina Martínez-FernándezBeata Bal-DomańskaMaría Cristina OrtizBeata BiesagaMaria Cristina VilaBeata Bieszk-StolorzMaría Cruz López De AyalaBeata BucheltMaría D. OdriozolaBeata Bujak-GizyckaMaria Da Gloria Teixeira De SousaBeata CałkaMaria D’AccoltiBeata CzarneckaMaria DaglaBeata DobrowolskaMaria Das Dores Sanches FormosinhoBeata Filip-PsurskaMaria De Fátima Pereira Da SilvaBeata Fornal-PieniakMaria De FrancescoBeata GavurovaMaria De Jesus Mendes Da FonsecaBeata GawryszewskaMaría De Las Nieves GonzálezBeata HoreckaMaría De Los Ángeles Ortega De La TorreBeata Joanna GawryszewskaMaría De Los Angeles Rodríguez DomenechBeata KarakiewiczMaría De Los Ángeles Valdemorosos San EmeterioBeata ŁoniewskaMaria De Lourdes PereiraBeata ŁubiankaMaria Del Carmen Casal AnguloBeata MazińskaMaría Del Carmen López-BerlangaBeata MrugalskaMaria Del Carmen Lozano EstevanBeáta NovotnáMaría Del Carmen Miranda DuroBeata OlejnickaMaria Del Carmen Ortega-NavasBeata PlutaMaría Del Carmen Pérez-FuentesBeata SadowskaMaria Del Carmen Ramos-HerreraBeata Sarecka-HujarMaría Del Carmen Sánchez CarreiraBeata SeinauskieneMaría Del Henar Pérez-HerreroBeata Skowron-MielnikMaría Del Mar Agudo RomeoBeata SperkowskaMaría Del Mar Fernández-MartínezBeata Stelmach-FitaMaría Del Mar Ferradás CanedoBeata SzluzMaría Del Mar Martín AragónBeata Zatwarnicka-MaduraMaría Del Mar Molero JuradoBeate GruberMaría Del Mar Pastor-BravoBeatrice Adriana BalgiuMaría Del Mar Sánchez-FuentesBeatrice CairoMaría Del Mar Simón MárquezBeatrice CasiniMaría Del Refugio Castañeda-ChávezBéatrice FerversMaria Do Rosário MartinsBeatrice KuhlmannMaría Dolores Díaz NogueraBeatrice ScazzocchioMaría Dolores Granado CastroBeatrice ThielmannMaria Dolores Guerra-MartínBeatrice WagnerMaría Dolores López FrancoBeatrix SélleiMaría Dolores Marrodán SerranoBeatriz Achiaga MenorMaría Dolores Martínez-AiresBeatriz ArranzMaría Dolores Redel MacíasBeatriz Bonete LópezMaría Dolores Sánchez FernándezBeatriz CasaisMaria Dos Anjos DixeBeatriz DelgadoMaria Dos Anjos PiresBeatriz FebreroMaria DzikućBeatriz GarcíaMaria Elena Crespo-LopezBeatriz Gómez-MartínMaria Elena FerreroBeatriz Helena TessMaria Elena LatinoBeatriz Lucas-MolinaMaria Elisabete NevesBeatriz MinghelliMaria Elvira CorreaBeatriz Navarro-BrazálezMaria Enrica BettinelliBeatriz Núñez AnguloMaría Esperanza RuizBeatriz PérezMaria EspositoBeatriz Rodríguez SánchezMaría Esther García BuadesBeatriz Rodríguez-RocaMaria FaulknerBeatriz Servilha BrocchiMaria FedeleBeatriz Sevilla-MoránMaria Federica TrombettaBeatriz Sibaja TeránMaria FerraraBeatriz Villarejo-CarballidoMaria FerreiraBeau BezaMaria FilekBebiana CondeMaria Francesca MilazzoBecca FeldmanMaria Francesca PiazzaBechan SharmaMaria FrosiniBecky TalynMaria Gabriela M. PinhoBeda BuchelMaria Gabriella VersoBedřich MoldanMaria Gabriella VillaniBee K. TanMaria GacekBeelee ChuaMaria GiannìBefikadu WubishetMaria Giovanna CilibertiBegoña EspejoMaria Giulia CristofaroBegoña GuiraoMaria Giuseppina PetruzzelliBegoña Martinez-JarretaMaría González-OlmoBehnam KhorshidiMaria GrajdianBehnam VafadariMaria Grazia Lourdes MonacoBehzad HeibatiMaria Grazia MatarazzoBeiyi HuMaría Huertas González-SerranoBekir BartinMaria Icíar Arteagoitia CalvoBelayat HossainMaría Isabel Amor AlmedinaBelen Alonso OrtizMaría Isabel Carretero AbellánBelén DerquMaria Isabel Dias MarquesBelen LópezMaria Isabel MármolBelén Pedregal MateosMaria Isabel Tomás-RodríguezBélgica Pacheco-BlancoMaría IzcoBelgin SeverMaria J. Casanueva-MarencoBelinda HornbyMaría J. Ibáñez-GonzálezBelkis Sulbarán-RangelMaría J. Morales-GázquezBella SavitskyMaria JakubikBen BrisboisMaria Jesus GarciaBen ClaytonMaría Jesús Núñez-IglesiasBen GrayMaria João MenesesBen SeligmannMaria João Valente Da Silva CoutoBen Y. F. FongMaria João VelezBen Z. KatzMaria José De Oliveira SantosBenedetta RagniMaría José García-PolaBenedetto BarabinoMaría José LeraBenedetto TerraciniMaría José Martínez PatiñoBénédicte M. BatrancourtMaria José Miquel-RomeroBenedikt W. PelzerMaría José Muñoz-AlférezBenedito C. PrezotoMaría José Pujalte-JesúsBeni Gomez-ZunigaMaria José Quina GaldinoBenito León Del BarcoMaria José RibeiroBenjamin AnsaMaria José SáBenjamin Arthur TurturiceMaría José Suárez-LópezBenjamin BostickMaria José VaradinovBenjamin BuckleyMaría Josefa Herrero FernandezBenjamin ChrisingerMaria Judite AlmeidaBenjamin David WeedonMaria K. SvenssonBenjamin GantenbeinMaria KapritsouBenjamín HerrerosMaria KorreBenjamin LandMaria KrechowiczBenjamin O. LaddMaria KyritsiBenjamin R. BradyMaría L. Flores-LópezBenjamin R. EvansMaria L. MateusBenjamin SeeligerMaria LagomarsinoBenjamin SteinhilberMaría LameirasBenjamin WachtlerMaria Laura AlfieriBenjamin WilliamsMaria LeeBenno BelkeMaria Leonor SilvaBenno KrachlerMaria Leticia CruzBenny S. Eathakkattu AntonyMaria Lidia MasciaBenny TjahjonoMaria LivanouBenoit BediouMaría Lourdes Gutiérrez-CarrilloBenoît BernardMaria LucaBenoit ChenaisMaría Luisa Avargues-NavarroBenoit JobinMaria Luisa BarrigónBenoît MissetMaría Luisa Del Río AraújoBenoit NemeryMaría Luisa Delgado-LosadaBenoit PuginMaria Luisa Di MartinoBenyamin AhmadniaMaria Luisa Esteban SalvadorBen-Yi LiauMaria Luisa GennariBeomseok KoMaria Luisa IndianaBeraki Bahre MehariMaria Luisa MarvánBerna RahiMaria Luisa PedditziBernadeta Lelonek-KuletaMaria Luísa Pereira SoaresBernadette AlbaneseMaria Luisa RicciBernadette FlanaganMaría Luisa Rico GómezBernard NaughtonMaria Luisa Rodero-CosanoBernardetta IzydorczykMaría Luisa Zagalaz SánchezBernardino CerdaMaría M. Morales Suárez-VarelaBernardino Javier Sánchez-AlcarazMaria Maddalena Del Gallo Di RoccagiovineBernardo J. KrauseMaria Magdalena Bujnowska-FedakBernardo LopesMaría Magdalena Fernández ValeraBernat BuscaMaria MaiaBernat BuscàMaria Manuela PeixotoBernd FrickMaria Manuela Prieto GonzalezBernd WallnerMaría Mar OrtaBernhard IglsederMaria Margarida PaixãoBernhard LangerMaría MarínBernhard MaischMaría MeloBernhard RauchMaria MenshakovaBernice RedleyMaria MichailBernie M. PaulyMaría Molinos-SenanteBert Jan Potter Van LoonMaria Montiel TroyaBerta Ausín BenitoMaria Montserrat Rivera Del ÁlamoBerta SchnettlerMaria MoudatsouBertram OpitzMaría NavarroBertrand DautzenbergMaria Nieves Pérez MarfilBertrand KaefferMaria NowakBertsche AstridMaria NyholmBeth BrodskyMaría Orosia Lucha-LópezBeth FieldsMaria PachesBeth H. ChaneyMaria PalmieriBeth Maina AhlbergMaria Paola CecchiniBeth Rose MiddletonMaria PapadakakiBethesda O’ConnellMaria PaparoupaBettina DebûMaría Patrocinio Ariza VegaBettina HöchliMaria Paula SantosBettina PollokMaria Pia FerrazBetty DoreMaria Pia Foschino-BarbaroBetty ExintarisMaria Pia MaraghiniBetty H. La FranceMaría Pilar Aparicio-FloresBeverly MayMaría Pilar MatudBeverly RoskosMaría Pilar Pecci-LloretBharani Krishna Mynampati ArunadithyaMaría Pilar Salguero-AlcañizBharat AcharyaMaria PilgunBhaven ModhaMaría Piñeiro-IglesiasBhavuk GargMaria Pokorska-ŚpiewakBhawna GuptaMaria QuattropaniBhikhari Prasad TharuMaria QuintiglianoBhola PradhanMaria R. BonsignoreBhola Shankar PradhanMaria RagostaBhubaneswor DhakalMaría Ramos-MonserratBhupal BanMaria Raquel BarbosaBiagio BaroneMaria Regina CapeloBiagio RaponeMaria RicciardiBiagio SantellaMaria Rita BiancoBianca L. GarnerMaria Rita CiteroniBianca Maria Serena InguscioMaría Roca LlorensBibhudutta PandaMaria Rosa Suñer SolerBibhuti K. SarMaria Rosaria De BlasiisBidisha RoyMaria Rosaria GualanoBiedma Ferrer José MaríaMaria Rosaria VadrucciBifeng HuMaria Rosaria VarìBigboy NgwenyaMaria RubegaBiljana KurtovićMaria Rubio AparicioBill JesdaleMaria SaintrainBilly C. L. SoMaria Salem IbrahimBilly T. W. YuMaria Salomé FerreiraBimal K. PaulMaría Salvadora Ramírez JiménezBimal Kanti PaulMaría Sánchez-ZafraBin QiuMaría Santagueda-VillanuevaBing WangMaría Sanz RemachaBing YuMaria SarapultsevaBinh Q. TranMaria ScaturroBinosha FernandoMaria SeidelBinoy KumaranMaria ShindovaBipasha AhmedMaria Shuk Yu HungBipin Kumar AcharyaMaria StrömbäckBircan ErbasMaria SuciuBirger TrollforsMaria T. PontoBirgit BabitschMaria T. Van HartenBirgit U. StetinaMaría Teresa AngueraBirgitta KerstisMaria Teresa Armúa-FernándezBirthe DinesenMaría Teresa Barral SilvaBisheng DuMaria Teresa ColominaBishnupriya SahooMaría Teresa Costado DiosBissera PilichevaMaría Teresa Cuerdo VilchesBiswajit MaharathiMaria Teresa Folgôa BatistaBjørn HiltMaría Teresa García-OrdásBjorn HolsteinMaria Teresa GuagnanoBjörn KrügerMaria Teresa HerdeiroBjörn PanteleitMaría Teresa Iglesias LópezBjörn TampeMaría Teresa LópezBlair UniackeMaría Teresa Martínez-RomeroBlake Byron WalkerMaria Teresa Murillo-LlorenteBlake C. ColclasureMaria Teresa Nestares PleguezueloBlake DensleyMaría Teresa Ocaña-MoralBlake PeckMaría Teresa Solis-SotoBlake PolandMaria Teresa TomasBlake Victor KentMaría Vanessa VillasanaBlanca Arenas-RamírezMaria VaradinovBlanca De-la-Cruz-TorresMaría Vázquez-GuimaraensBlanca Estela Bernal EscotoMaria Vicent JuanBlanca Lumbreras LacarraMaria VitaleBlanca R. LopezMaria VitielloBlanca Roman ViñasMaria WaleryBlanca Rosa García RiveraMaria WalkobingerBlanca TejeroMaria WielsøeBlanka KlímováMaría Yolanda Castellote-CaballeroBlanka StiburkovaMaria-Angelica BenczeBłażej CieślikMaría-Arántzazu Ruescas-NicolauBłażej KudłakMaria-Claudia DiaconeasaBłażej ŁyszczarzMaria-Crina RaduBlessing Akombi-InyangMariacrocetta SambitoBo PangMaría-Dolores Lanzarote-FernándezBo YangMariaelena TagliabueBo Young HongMariagabriella PuglieseBoaz BarakMaria-Giuliana VannucchiBobo Hi Po LauMariagrazia BenassiBo-Chiuan SuMariagrazia Di GiuseppeBogdan BatkoMariagrazia PiccioniBogdan CatargiMaria-Ioanna ChristodoulouBogdan Gabriel ȘlencuMaría-Jesús CavaBogdan IancuMaría-Jesús LirolaBogdan Ionel TambaMaría-Jesús Tabernero-DuqueBogdan OanceaMaría-José ArgenteBogdan Silviu UngureanuMaría-José Hernández-SerranoBogdan SkwarzecMarialaura CorrenteBoguslaw MichalikMarialaura Di TellaBogusława UrbaniakMaría-Leticia Meseguer-SantamaríaBohdan RożnowskiMarialuisa GandolfiBohumil KotlíkMarián AmbrozyBojan ŠarkanjMarian KachniarzBonggeun SongMarián Pérez-MarínBongju KimMarián RuckiBong-Ju ParkMarián SchwarzBongsuk SungMarian SimionBoniphace KutelaMarian Sorin PaveliuBonnie FournierMarian WoźniakBonnie JanzenMariana Arias AvilaBonnie K. L. MakMariana Calábria LopesBonnie KerkerMariana CernicovaBoowook KimMariana FigueiroBoredi Silas ChidiMariana NicolaeBoris B. NiraulaMariana P. SocalBoris C. Rodríguez-MartínMariana Renovato MartinsBoris EhrensteinMariana S. CastroBoris ErshovMarianela Denegri CoriaBoris GuyotMariangela De RobertisBoris JidovtseffMariangela PuciBoris PalellaMariangela ZengaBorja Herrero De La ParteMarianna BellafioreBorja Matías PompaMarianna MazzaBorja Rivero-JiménezMarianna MuravyevaBorna AbramovićMarianna TotaniBoro ŠtrumbeljMarianne MorsethBoshra ArnoutMarianne SilvaBouzas CristinaMarianne SimonsBo-Wei ZhuMariano CingolaniBoyen HuangMariano Méndez-SuárezBo-Young HongMariano Meseguer De PedroBožana Lončar BrzakMariano SastreBożena BabiarzMaría-Pilar Molina-TorresBožena CukrowskaMaria-Raquel G. SilvaBozena Dorota HrynyszynMariarita BrancaccioBożena HołaMarica BarbaritanoBożena KróliczewskaMaricarmen Leandra Lecuna TovarBożena MuszyńskaMaricarmen VizcainoBraden M. LiMaricica StoicaBradley TatarMarie Anne Eurie ForioBrady GrantMarie BraultBrahim BchirMarie Desnos-OllivierBrahmaputra MarjadiMarie EssigBralind KiriMarie HattinghBran Barral BucetaMarie JohnstonBranden BornMarie LinderBranden NemecekMarie Lisa KapellerBrandon A. KohrtMarie ThomasBrandon BurrMarie TreslovaBrandon EgglestonMarie VahterBrandon P. ReinesMarie-Claude JaurandBrandon RestrepoMarie-Claude PaquetteBranko CellerMarie-Frédérique BacquéBrecht DevleesschauwerMarie-Josée FleuryBreeanne JacksonMarieke DirksenBrenda BongaertsMarietta KissBrenda Cabrera-MendozaMarietta Van Der LindenBrenda CustodioMariia RizunBrenda F. SealsMarija BogatajBrenda GroenMarija Djekić-IvankovićBrenda Imelda Boroel CervantesMarija DumnićBrenda RoblesMarija JevticBrenda Sarahi Cervantes-LunaMarija LjubicicBrendan GearyMarija OpačakBrendan RyanMarija-Magdalena PetrinovicBrent A. BakerMarijana Despotović-ZrakićBrent PetersonMarika KarikoskiBrentyn RammMarilena PapageorgiouBret DickeyMarilène FilbetBrett BlighMarilia AndradeBrett L. HurstMarília Santos AndradeBrett LidburyMarilou OuelletBrett R. ElyMarilyn CruickshankBrett SingerMarin ŞenilăBrett ToelleMarina Aleksandrovna DarenskayaBrian Andrew Van TineMarina BorroBrian BakerMarina BuzziBrian BellMarina Cervera Alonso De MedinaBrian BieberMarina Evrard MichelBrian BossakMarina Gil-CalvoBrian C. J. MooreMarina KhazovaBrian ChicoineMarina M. S. Cabral-PintoBrian D. PooleMarina MisciosciaBrian GodmanMarina OteleaBrian HoweMarina ParoliniBrian HuangMarina PericBrian KosterMarina PiscopoBrian MosierMarina RobasBrian MurphyMarina Sánchez-RicoBrian P. LeeMarina SantaellaBrian PavilonisMarina SartiniBrian PiperMarina SofiaBrian QuayMarina TulinBrian SchumacherMarina VasiljevaBrian SchwartzMarinella CocoBrian VinerMarinella ZingaleBrian WardMarino GattoBrian WeirMarino VilovicBriana WyattMarino VilovićBrianna L. NewlandMarinus Van IJzendoornBrianna LarsenMario A. R. DantasBrianna OsetinskyMario Alfonso Murillo TovarBrice BalmerMario Alvarado-LorenzoBrid KaracanMario Álvarez-CabriaBridget HutchensMário António Cardoso MarquesBrienne N. SeinerMario BerettaBrigadier LibandaMario Bernardo-FilhoBrigitte JoergMario BilićBrigitte Le Magueresse-BattistoniMario CannataroBrijesh Kumar SinghMario Corrales-SerranoBrita SomerkoskiMario De FeliceBritt ÖstlundMario Di TragliaBritta BerglundMario FargnoliBrittanie Atteberry-AshMárió GajdácsBrittany SchulerMário Jorge CostaBrittany SmallsMario José Costa De MacedoBritton W. BrewerMario LourençoBroch Trygve BeyerMario LovrićBrody HeritageMario LucianoBronwyn DolmanMario MalerbaBronwyn MyersMário MarquesBronwyn ThompsonMario Martínez PizarroBrook BelayMario Martin-GamboaBruce Baer ArnoldMario MiccoliBruce I. RapleyMário Nuno MataBruce Jeffrey RogersMario ParisBruce JuddMario Perez SayansBruce SpittleMario RajasBruce SunderlandMário SáBruce W. CraigMario TaniBruna Coelho LopesMário ToméBruna Figueiredo ManzoMario Ulises Herrera-PérezBruna Lavinas Sayed PiccianiMario Ulises Pérez-ZepedaBrunislav MatasovićMario Valera-PozoBruno B. GiudicelliMario VersaciBruno BonnechèreMário W. L. MoreiraBruno Bueno-SilvaMariola BorowskaBruno CacopardoMariola DrozdBruno ChrcanovicMariola JaniszewskaBruno FabianoMarion EnglandBruno FerreiraMarion Kavanaugh-LynchBruno Gabriel N. AndradeMarion RussellBruno José Nievas SorianoMarion SlackBruno LemkeMarios AntonakakisBruno Macedo De SousaMarios ConstantinouBruno MagalhãesMarios RossidesBruno MarquesMarios SpanakisBruno Miguel DelgadoMarios SpiliotopoulosBruno Miguel VelosoMarisa CarvalhoBruno MilletMarisa FilipeBruno MinottiMarisa GranatoBruno S. GonçalvesMarisa GuillénBruno TiloccaMarisol BecerraBruno TrimarcoMarisol D. McDanielBruno VendittoMarisol RodríguezBruno Vieira BertonciniMarissa KalogaBruria AdiniMaristella GussoniBryan FullerMarius CiurlionisBryan J. MathisMarius ConstantinescuBryan KolbMarius RademakerBryan M. GeeMarius-Corneliu MarinasBryan McintoshMarius-Cristian PanaBryan OrmondMariusz DuplagaBryan R. BrownMariusz DylagBryan RodgersMariusz FinkielszteinBryane MichaelMariusz GoniewiczBryanne BellovaryMariusz GujskiBryanne N. BellovaryMariusz JedlińskiBryce HughesMariusz LuterekBud CooperMariusz MaciejczakBugajski PiotrMariusz NiekurzakBukola G. OlutolaMariusz SołtysikBukola UsidameMariusz SzóstakBülent ErenMariusz TopolskiBülent Okan MiçooğullarıMariusz WysokińskiBumjoon KangMariusz ZiębaBundit SornpaisarnMariusz ZielińskiBurcu AdivarMariya KovalevaBurkhard FranzMariyana SchoultzBuse Ozcan KahramanMarjan MohammadzadehBuuma MaishaMarja-Tellervo MäkinenByoungsoo KimMarjeta TerčeljByung Uk LeeMark A. BrownByung Yi KoMark ButtonByung Yong JeongMark CarewByung-Hoon KimMark D. WhitakerByung-Jik KimMark E. HarmonByungjoo ChoiMark Elisabeth Theodorus WillemsByung-Kwan SeoMark FarrarByungryul AnMark FleisherByung-Sun LeeMark ForshawC. F. LinMark G. E. KellyC. Fred AlfordMark GillC. M. Sabbir AhmedMark GoodhewCadhla FirthMark GriffithsCaijun SunMark H. MyersCai-Mei ZhengMark HectorCaio SarmentoMark HeirigsCaitlin P. McMullenMark HenricksonCaitlin VayroMark J. WilsonCaitriona CahirMark KeimCaixia LiuMark LedbetterCaleb Panapa Edward MarstersMark LindquistCaleb RasconMark M. JankoCalin CiufudeanMark MalisaCalla M. GoekeMark MankowskiCalogero CarusoMark MossCalum BennachieMark R. JohnsonCalvin LuMark RegnerusCamelia Daniela HateganMark ReybrouckCamelia Delia VoicuMark RobinsonCamelia SoponaruMark SchmidtCameron E. WebbMark SmithCameron G. EstrichMark Tambe KeboaCameron GordonMark TullyCameron McEwanMark V. PaulyCameron PeersMark V. SiracuseCameron ZachresonMark WaldronCamila Aparecida BorgesMark WardCamila Teixeira VazMark WolfsonCamilla HansenMarkel Rico-GonzálezCamilla Kin Ming LoMarketa BeitlovaCamilla MargaroliMarkéta ŠantrůčkováCamilla MazzoliMarkku LaatikainenCamilla Wikström-GrotellMarkku LöytönenCamille A. ClareMarkku TykkyläinenCamille Anne MartinaMarko KumrićCamille DieterleMarko LaaksonenCamille InquimbertMarko MatijevićCamille SanreyMarko VuletićCamlyn MasudaMarko ZdravkovićCamron Michael AminMarkos KlonizakisCandace BurtonMarkus FendtCandace S. BrownMarkus FeserCandan HizelMarkus JobstCándido InglésMarkus KleinCandy BrownMarkus KlimekCandy Lim ChiuMarkus KranzlerCao Ying-TingMarkus MandlCaradee WrightMarkus PuchingerCaralina Marín De EvsikovaMarkus ReiserCarel JansenMarkus StraußCaren J. FrostMarkus WilhelmiCarey MatherMarla Andréia Garcia De AvilaCarin Staland-NymanMarlee BowerCarina DantasMarlena DrągCarina De Fátima RodriguesMarlena RobakowskaCarina HeekeMarlene Filipa Da Natividade E. SousaCarina HellqvistMarlene HauptCarina Lundh HagelinMarlene WiggillCarita HakanssonMarlies E. C. ElfrinkCarl BorchgrevinkMarlos Rodrigues DominguesCarl FriskMarnie DownesCarl J. BassiMarquis HawkinsCarl LatkinMarquisette Glass LewisCarl LavieMarsen Garcia Pinto CoelhoCarl PaytonMarsha MorganCarla Alexandra Martins FonteMarshall ChasinCarla BergMarshall LightowlersCarla Blázquez-FernándezMarshall S. JiangCarla ComacchioMarta Anna SzychlinskaCarla Estrada-MuñozMarta BrkovicCarla Maria Batista Ferreira PiresMarta Jeruszka-BielakCarla MateusMarta LimaCarla Matos SilvaMarta Linares-ManriqueCarla MercadoMarta MakowskaCarla MouroMarta MartínezCarla Oliveira HenriquesMarta Medina GarcíaCarla PalmaMarta MuszalikCarla Patrícia Quintaneiro AntunesMarta Ortega-GasparCarla PiresMarta PelczyńskaCarla ReigadaMarta Ramon-KrauelCarla Roberta De Oliveira CarvalhoMarta Rapado-CastroCarla RoverselliMarta Regina Cezar-VazCarla SerrãoMarta RzadkiewiczCarla Sofia SilvaMarta SarkozyCarla SteccoMarta Seiz PuyueloCarla Toledo-NeiraMarta Stelmach-MardasCarla VidoniMárta VarghaCarla VillaMarta Wysocka-MincewiczCarles BarriocanalMarta ZagrebelskyCarles ManeraMarta ZiółkowskaCarlo Alberto ArtusiMarte Karoline Råberg KjøllesdalCarlo Andrea BiraghiMartha DavisCarlo BieńkowskiMartha KelesiCarlo BizMarti LindseyCarlo CervellatiMartin DodmanCarlo ContiniMartin KapitánCarlo D’AgostinoMartin KrsekCarlo D’AscenziMartin LehnertCarlo Di PaoloMartin M. EdreiraCarlo DragoMartin PienkowskiCarlo Federico Dall’OmoMartin RootCarlo Giacomo LeoMartin RožánekCarlo GrecoMartina BlaškováCarlo LaiMartina SamiotakiCarlo LajoloMartino RuggieriCarlo NavarraMarusia Rentería-VillalobosCarlo PietrasantaMary Ann JarvisCarlo RicciardiMary C. SheehanCarlo RussoMary Clare HanoCarlo SignorelliMary GouvaCarlo TorreMary HearstCarlo ZancanaroMary HrywnaCarlos A. CifuentesMary J. KastenCarlos A. M. CarvalhoMary Jo IozzioCarlos A. ManzanoMary Katherine HoyCarlos A. Martínez-HuitleMary LashleyCarlos AbreuMary McKennaCarlos Alberto Moreira-FilhoMary NolanCarlos Alfonso Tovilla-ZárateMary NthambiCarlos Andres Aguirre-NuñezMary O’ReillyCarlos AparicioMary Patricia NowalkCarlos Arcila CalderónMary YanCarlos BaixauliMaryam AbarghoueinejadCarlos BarcelóMaryam KazemiCarlos Bernal-UtreraMaryam LotfiCarlos Brotons-CuixartMaryam MalekigorjiCarlos CalderonMaryam SalehiCarlos CevallosMaryam VasefiCarlos CoboMaryAnn G. RadlinskyCarlos Cuenca-BarralesMary-Claire KennedyCarlos David Gómez-CarmonaMary-Jon LudyCarlos De Las Heras-PedrosaMaryland PaoCarlos Díaz DelgadoMarzena Filipowicz-ChomkoCarlos Díaz-CaroMarzena Jankowska-MihułowiczCarlos Durantez-FernándezMarzena MackowiakCarlos Eduardo Barros GonçalvesMasa SasagawaCarlos Eduardo Fonseca-AlvesMasaaki KonishiCarlos Eduardo FontanaMasaaki KurasakiCarlos Eduardo RaymundoMasafumi MurataniCarlos Eduardo Sanches De AndradeMasafumi TatedaCarlos Eduardo ThomazMasaharu KagawaCarlos F. SilvaMasahiro MishinaCarlos FalcãoMasahiro SakataCarlos Fernandez-LlatasMasahiro SugimotoCarlos Fernando MourãoMasahiro TanakaCarlos FerrasMasahito MoritaCarlos Franco-ParedesMasakazu HirotaCarlos Gonzalez-JuanateyMasako ShomuraCarlos H. G. MartinsMasami NozakiCarlos HernandoMasanobu KiiCarlos HervásMasanobu OkayamaCarlos Hervás-GómezMasao ToyodaCarlos J. MarquesMasaomi IkedaCarlos Javier López-GutiérrezMasaru TanakaCárlos L. Ayán PérezMasashi NakamuraCarlos Lago PeñasMasataka ShirakiCarlos LaranjeiraMasataka SumiyoshiCarlos Llanes-ÁlvarezMasataka SuwaCarlos López-de-CelisMasato HondaCarlos Luque MorenoMasatoshi OhnishiCarlos M. CoelhoMasatsugu OruiCarlos Manuel Torres AlmeidaMasaya TakahashiCarlos Mario Gutiérrez AguilarMasayoshi ZaitsuCarlos Medina-PerezMasayuki OkudaCarlos Méndez-MartínezMasayuki OzakiCarlos Mestanza-RamónMasayuki TakigawaCarlos Montero-CarreteroMasfer AlkahtaniCarlos Navarro-CuéllarMasiero StefanoCarlos O’Connor-ReinaMassimiliano ConsonCarlos OsorioMassimiliano MannoCarlos OtínMassimiliano RussoCarlos PenillaMassimo BracciCarlos Pérez-GonzálezMassimo CapocciaCarlos Pérez-PlasenciaMassimo TrottaCarlos Ramirez ReyesMat DalgleishCarlos Roberto ValencioMateus Henrique PetrarcaCarlos Román CascónMateus Rennó SantosCarlos RonceroMateusz BabickiCarlos Ruiz-NuñezMateusz CybulskiCarlos SalaveraMateusz JakubiakCarlos Salavera BordásMateusz JankiewiczCarlos SampaioMateusz JankowskiCarlos SantoyoMateusz RozmiarekCarlos Saus-OrtegaMathew J. GregoskiCarlos SequeiraMathew O’GradyCarlos SilvaMatilde Díaz HernándezCarlos SiqueiraMatilde RodriguesCarlos SoaresMatluba KhanCarlos Soubervielle-MontalvoMatt A. PriceCarlos TrenadoMatt DeLisiCarlos VasconcelosMatteo CortesiCarlos Villaseñor-MoraMatteo CrippaCarlos Vladimir Rodríguez-CaballeroMatteo De MarcoCarlos Vladimir Zambrano RodríguezMatteo PaciniCarlotta CocchettiMatteo RiccòCarlotta LunghiMatthew ArmstrongCarlotta PipoloMatthew ChrismanCarl-Philipp JansenMatthew ColeCarlton Ralph C. DanielMatthew H. SharpCarly FletcherMatthew KoehlerCarmela Anna MiglioriMatthew McGrailCarmela BravaccioMatthew R. X. DentithCarmela ComitoMatthew RogatzkiCarmela DonatoMatthew SchoeneCarmela Martinez-VispoMatthew WadeCarmela MentoMatthias EhrhardtCarmela RinaldiMatthias JentschkeCarmelia Mariana Dragomir-BălănicăMatthias KapischkeCarmelinda RuggieroMatthias KorellCarmelo BellarditaMatthias MöhnerCarmelo CalìMatthias MulazzaniCarmelo CorsaroMatthias NüblingCarmelo Lo FaroMattias GaglioCarmelo RomeoMattsson JanetCarmelo SgarlataMaura BenedettiCarmen Álvarez-NietoMaura ShramkoCarmen BarcenaMaura TomatisCarmen C. W. LimMaureen LongCarmen Capo-LugoMaureen MacNamaraCarmen CasanovasMaureen RyanCarmen Cipriano-CrespoMaurice MarsCarmen ConcertoMaurício KritzCarmen Costa-SánchezMauricio Matus-LópezCarmen Cristófol RodríguezMaurizio MarchesiniCarmen D. Álvarez-AlbeloMaurizio NaldiCarmen Daniela Quero CaleroMaurizio SibillaCarmen De KockMauro GaspariniCarmen Del Pilar Gallardo MontesMauro LombardoCarmen DinizMauro MurgiaCarmen EmbregtsMauro SoldatiCarmen Enrique-MirónMautusi MitraCarmen F. Navarro-PérezMax BarnishCarmen Ferragut FiolMax O. StephensonCarmen Fiuza-LucesMax WesternCarmen FreireMaximilian KollmussCarmen GarcésMaxwell Fordjour Antwi-AfariCarmen Gómez-BerrocalMay YehCarmen Jiménez AntonaMayanka AwasthiCarmen L. A. M. Vleggeert - LankampMaysaa NemerCarmen Llorente-CejudoMayte Benlloch OsunaCarmen López-EscribanoMayumi NakagawaCarmen LucasMayumi NishiCarmen María DomínguezMbodja MougouéCarmen Maria Gonzalez DomenechMcBean Edward ArthurCarmen Moret-TatayMd Abdul HalimCarmen Patino-AlonsoMd Abdul WazedCarmen Pérez-RodrigoMd. Ashraful Islam MollaCarmen Pilar Martí-BallesterMd Ekhtear HossainCarmen RequenaMd. Moshfekus Saleh-E-InCarmen RinaldiMd NadiruzzamanCarmen Rodríguez GarcíaMd Nazirul Islam SarkerCarmen Rodríguez JiménezMd Rashed BhuyanCarmen Ropero-PadillaMd. Shiblur RahamanCarmen RubioMd WahiduzzamanCarmen SolanoMdutshekelwa C. NdlovuCarmen SotoMeagan McCollumCarmen Suárez-SerranoMeagen RosenthalCarmen Vizoso-GómezMedina CayetanoCarmine FinelliMee Seong ImCarmine MerolaMeenakshi AhluwaliaCarmine RoccaMeg E. MorrisCarmine SummoMeg LittleCarmine ViolaMeg MoormanCarmit PadanMegan A. QuinnCarol BrayneMegan E. McGroddyCarol BurnsMegan L. UhelskiCarol C. McDonoughMegan PaceleyCarol E. BrownMegan RobertsCarol FriesenMegha AggarwalCarol J. HomkoMegumi UchidaCarol JohnstonMehdi EmamCarol KaoMehdi Pawlak-ChaouchCarol McKinstryMehran Eskandari TorbaghanCarol NashMei LiuCarol Yen Chin LinMei Shan LiewCarole C. UpshurMei TruebaCarole ComettiMei-Jin GuoCarole PelissierMeine Van NoordwijkCarolien SinoMelania DovizioCarolin KilianMelanie ConnorCarolina Alonso MonteroMelanie HandleyCarolina Archundia HerreraMelanie L. PlinsingaCarolina Bringas MolledaMelanie Reuter-OppermannCarolina G. OjedaMelchor Sanchez-MartinezCarolina Gonzálvez MaciáMeleckidzedeck KhayesiCarolina LuengoMelinda Butsch KovacicCarolina M. AzañedoMelinda MetaxasCarolina MachadoMelissa A. St HilaireCarolina N. KeimMelissa CareyCarolina Perez-HeydrichMelissa K. LadymanCarolina Piña RamirezMelissa Kitner-TrioloCarolina Ribeiro XavierMelissa S. W. YamauchiCarolina Rodríguez-LlorenteMelissa S. Y. ThongCarolina RomeroMelissa SnyderCarolina TinajeroMelissa Suk Yin ThongCarolina Vila-ChãMeng-Che TsaiCaroline A. SpiranovicMeng-Chi TangCaroline J. Dodd-ReynoldsMeng-Ting LinCaroline JagoeMercedes Llorent-VaqueroCaroline KönigMercedes Matás CastilloCaroline PhelanMercedes Rizo BaezaCaroline S. WestwoodMercedes Vernetta SantanaCaroline StrevensMercedesz OrsosCaroline SunderlandMercy MashingaidzeCaroline Walker-GleavesMeredith A. JaggerCaroll HermannMeredith GartinCarolyn AugustaMeredith GreshamCarolyn BerrymanMert SevilCarolyn E. MooreMeryem GrabskiCarolyn Gentle-GenittyMette L. BaranCarolyn HeckmanMi Yang JeonCarolyn M. RichardsonMi Young KimCarolynne MasonMi ZhouCarrie StewartMia StokmansCarrington ShepherdMian HuangCarson PattersonMiao-Ju HsuCarsten LippoldMichael AdorjanCaryn ZinnMichael BeachCasey MainsbridgeMichael BertocchiCasey PeirisMichael Bruneau JrCasey QuinnMichael CaryCashen M. BoccioMichael DeschenesCasimir MacGregorMichael K. MacKenzieCasimiro S. MunitaMichael MahanCaspar StephaniMichael McVeyCasper AgatonMichael MirekuCassandra ChaneyMichael NadorffCassandra M. JohnsonMichael OlasojiCassidy DelaneyMichael OpataCataldo DoriaMichael PrielerCatalin PopescuMichael RadinCatalina González-FortezaMichael RigbyCatalina MedinaMichael SpartalisCatalina Mihaela GrădinaruMichael TozeCatalina Sau Man NgMichael VannierCatalina Soriana SitnikovMichael WallerCatarina GodinhoMichael WeitzendorferCatarina GomesMichaela CoenenCatarina SamorinhaMichail Zografakis SfakianakisCatarina SilvaMichał BronikowskiCate ThomasMichał GieryczCaterina Adele ViganòMichał JaśkiewiczCaterina CavicchiMichał KierzkowskiCaterina FedeMichal KlichowskiCaterina Francesca GuidiMichał KrzemińskiCaterina GiannittoMichał KrzysztofikCaterina GozzoliMichał MiłekCaterina LeddaMichal OrdakCaterina TramontiMichal VágnerCath JacksonMichał WendtCatharine Ward ThompsonMichalis KoureasCatherine A. HeaneyMichalis SkordoulisCatherine A. LaBrenzMichel PérusseCatherine ArcherMichel TchuencheCatherine BaileyMichel WhiteCatherine ChanMichela TerlizziCatherine ChittleboroughMichela VecchiCatherine DausMichele AmorenaCatherine DraperMichele AndrasikCatherine FrancoisMichele MoohrCatherine HayesMichele ScipioniCatherine JordanMichelle G. NicholsCatherine L. ZemanMichelle J. TownsendCatherine LauMichelle MillerCatherine M. McAuleyMichelle ReidelCatherine MacLeodMichelle Weber RawlinsCatherine MacombeMichiaki KaiCatherine Marquis-FavreMichiel ClaessenCatherine ParadisMick Van TrotsenburgCatherine PenningtonMicol BronziniCatherine SaenzMieczysław AdamowiczCatherine SharpMieszko WieckiewiczCatherine Weikart YeckelMiguel A. Mariscal SaldañaCathleen L. DohertyMiguel A. Montero-AlonsoCathrine LinnesMiguel A. Perez-SousaCathrine ReineholmMiguel A. SimónCathriona KearnsMiguel A. Tejada GiráldezCathryn SparksMiguel A. VerdugoCathryne L. SchmitzMiguel AlcarazCátia CaneirasMiguel AmadoCátia MagalhãesMiguel Angel Alcántara-OrtigozaCátia MartinsMiguel Angel Alvarez-MonCátia SilvaMiguel Ángel CarboneroCátia VenâncioMiguel Ángel Gallard-VigilCatrinel Haught TrompMiguel Ángel Mateo PérezCayetana Ruiz-ZaldibarMiguel Ángel Navas-MartínCecelia WigalMiguel Ángel Rojo TiradoCécile BardonMiguel Angel Serrano RosaCécile QuantinMiguel Ángel Sogorb SánchezCécile RaillardMiguel García-JaénCecilia AriciMiguel González ValeiroCecilia ChanMiguel HuertaCecilia PerinMiguel Ladero GalánCecilia Simón RuedaMiguel LealCecilia SmaniottoMiguel Peixoto De AlmeidaCecília SzigetiMiguel Pic AguilarCecilia VarjuMiguel RicouCecilia VecoliMiguel Sánchez MorenoCecilie DahlMiguel TerrosoCecilie VarsiMiguel Toribio-MateasCees NoordamMiguel-Ángel García-MadurgaCeferino LópezMihaela Brindusa TudoseCelene B. MilanesMihaela Enache-ZegheruCelestino RodriguezMihaela MoscaluCelia HardingMihaela OlaruCelia Marti-GarciaMihaela OnofreiCelian RomanMihaela SimionescuCelina Salvador-GarcíaMihaela Tinca UdristioiuCéline KosterMihaela ZegreanCeline RozenblatMiharu NakanishiCéline TiffonMi-Heon RyuCelso Henrique Leite Silva JuniorMike AnastarioČeněk ŠašinkaMike ArmourCengiz AkbulutMike DanaherCenk ÇalışkanMike G. TsionasCeri PhillipsMike GoodmanCésar BerzosaMike ScottCésar Calvo-LoboMike ThelwellCésar CárdenasMikhail F. BorisenkovCesar Fernández-de-las-PeñasMikhail Yegorovich SinelnikovCésar Hidalgo-GarcíaMiki KakinumaCesar Leal CostaMikio IshiwatariCesar Lopez-CamarilloMiklós BánhidiCesare CerriMiklós HorváthCezar MorarMi-Kyoung ChoCezary KuśnierzMilada KrejciChad CriggerMilan AndrejićChad L. CrossMilan HoraChafia Touil-BoukoffaMilan KragovićChahid E. FouraliMilan TomaChahyun OhMildred MaisonetChai Hong RimMilica JankovicChaitanya GadepalliMilos PjanicChaitanya P. PuranikMiltiadis ZamparasChamara BasnayakeMina SuhChammah Judex KaundaMinan Al-EzziChan Gyu KangMinas E. PaschopoulosChan Kuan RongMinerva Codruta BadescuChan ShenMing GuoChanchal MandalMing LiuChandra DhakalMing LuoChandra Shekhar PantMing Yan ChungChandrakala Aluganti NarasimhuluMing Yu Claudia WongChandrakala GaneshMing ZhangChandrasekar RadhakrishnanMingfei PanChandrew RajakumarMing-Fu HsuChang GuoMing-Hsiung HsiaoChang Hyeong LeeMinghua LiuChang Won ChoiMingjian SunChang Won LeeMing-Lang TsengChang Yu HongMingliang ZhangChang-Chi LinMing-Quan GuoChang-Gyun RohMingxiao SuiChang-Ho JihnMingyuan ZhangChang-Hyun JinMinh Hoang NguyenChanghyun RohMinh NguyenChang-Kyoon SonMinha HongChangSeob YeoMin-Ho HanChanguk ChungMin-Hui LiChangwei ShaoMinh-Xuan TruongChangwoo ShonMin-Hyeon ParkChang-Yong JangMin-Hyu KimChang-Yu HongMiniati MarioChanpreet SinghMinjeong AnChansol HurrMinjung ShimChantal AmmiMinou Weijs - PerréeChantal Den DaasMin-Pei LinChantal FaucherMin-Seong HaChantel SloanMin-Suk KimChantelle RichmondMinxiang ZengChan-Young KwonMiodraga Stefanovska - PetkovskaChao BiMiomir MijićChao ChenMir Faeq Ali QuadriChao LuMiraj Ahmed BhuiyanChao SongMirella Gutiérrez-ArzaluzChao SunMiren AltunaChaoqi ChenMiriam Diez BoschChaoran GuoMiriam HaaksmaChao-Yu GuoMiriam HeymanChar LeungMiriam Pascual BenitoCharalambos CosterisMiriam Potocky-TripodiCharalampos AgakidisMiriam SannomiyaCharalampos GeorgiopoulosMiriam SchiffCharalampos KaragiannidisMirian Santamaría-PeláezCharalampos KarantonisMirja H. Gross-HemmiCharalampos KrommidasMirjam-Colette KempfCharalampos KyriakidesMirka MobiliaCharalampos MamoulakisMirko DuradoniCharalampos N. PitasMirko PeranoCharalampos SiristatidisMirna MihelčićCharalampos ThomasMirna ŽulecCharareh PourzandMiro JukićCharat ThongprayoonMiroslav BartakCharikleia Spyridon VrettouMiroslav FajfrChariklia Tziraki-SegalMiroslav HercegCharity AndersonMiroslav KocifajCharleen C. McNeillMiroslava NedyalkovaCharlene NielsenMiroslaw JanowskiCharles CrapoMiroslaw RuckiCharles DabonéMirosław RurekCharles HuangMirto FolettoCharles IkerionwuMiruna StanCharles M. MacVeanMiryam Martínez MartínezCharles MadewellMirza PojskicCharles MarksMisari OeCharles MpofuMitchell DwyerCharles MusselwhiteMitchell J. DowlingCharles Odilichukwu R. OkpalaMititelu-Tartau LilianaCharles PontonnierMitsuru ShimizuCharles Roberto TellesMitsuyoshi YoshidaCharles S. KamenMiyazato NaokiCharles W. LanksMi-Yeon SongCharles-Etienne BenoitMoacir MarocoloCharles-Henri DiMariaMoamen M. ElmassryCharli ErikssonMoawia KhatatbehCharlie ZhangMoeti Oriel TaioeCharlott RubachMogens Pfeiffer-JensenCharlotte ClarkMohamad KaddouraCharlotte CurrieMohamadi SarkarCharlotte JelleymanMohamed AbdelgiedCharlotte LawsonMohamed Abdo RizkCharlotte LoppieMohamed Aqiel DalvieCharlotte SteenblockMohamed F. AbdallahCharlotte Von GallMohamed Hamza El-SaeidCharlotte Wendelboe-NelsonMohamed Ibrahim El DidamonyCharly KeytsmanMohamed MahdiCharng-Cherng ChyauMohamed SassiCharoula NikolaouMohamed Yacine HaddoudChasity BrimeyerMohamed-Bassem AshourChaturaka RodrigoMohammad Abu Zafer SiddikChauren JungMohammad Al ShinwanCheerawit RattanapanMohammad AlamChellappagounder ThangavelMohammad Amin Hariri-ArdebiliChelsea KrachtMohammad Amjad KamalChelsea PelletierMohammad AvandChelsea StillmanMohammad HajizadehChen DanMohammad Hassan HodrojChen LingMohammad IranmaneshChen Sabag Ben-PoratMohammad JamshidiChen ShenMohammad Javad MohammadiChen YangMohammad KakooeiChen ZhangMohammad KarimiChen ZhaoMohammad Monirul HasanChen-Chi TsaiMohammad NajjarCheng BoonMohammad Nazmul HasanCheng PengMohammad ParsazadehCheng WangMohammad SaadCheng-Chia YangMohammad Shah JahanCheng-Chieh ChangMohammad Shokrolah ShiraziCheng-Chieh LinMohammad Sultan AlakhaliCheng-Chieh YenMohammad ValipourCheng-Fang YenMohammad ZamaniCheng-Hung TsaiMohammadreza RazvanChengpeng LuMohammed BahjaChenguang DuMohammed GagaouaChengxiang TangMohammed Khaled Al-HanawiCheng-Yang ChouMohammed M. MabkhotCheng-Yang HsiehMohammed MohsinCheng-Yen KaoMohammed Nader ShalabyCheng-Yuan HoMohammed RohaimChenxiao JiangMohan AmarasiriChen-Yi SongMohan PaudelChenyu HuangMohan RudrappaChenyu SunMohand DjeziriCheong K. ParkMohanraj ThirumalaiCheong KimMohd Dzulkhairi Mohd RaniCheonjae LeeMohmed Isaqali KarobariCherry Y. LeungMohsen SoltanifarCheryl BeselerMoïse CoëffierCheryl DelgadoMoises BetancortCheryl DissanayakeMoises Leon-JuarezCheryl Fairfield EstillMoisés MarquinaCheryl JonesMoiz VoraCheryl RegehrMojtaba EhsanifarCheryl WissehMojtaba KavianiCheuk Chi (George) TamMojtaba ShakerianChe-Yi ChouMojtaba VaismoradiChi HuynhMojtaba ZeraatpishehChia-Chu ChuMokarram HossainChia-Feng YenMoktar ChtaraChia-Hao ShihMollo Kenneth OtienoChia-Hsing HuangMomcilo JankovicChia-Hung ChouMoming LiChia-Liang HungMomotazur RahmanChiang LiuMona AshokChiao-En WuMona BoazChiara BaianoMona ElbarbaryChiara BelliaMona IonasChiara Benedetta CannataMona JaberChiara BiscariniMona K. SawhneyChiara BrulloMona VintilaChiara CadedduMonal YuwanatiChiara CantianiMonday IkhideChiara CappelliMonia VagniChiara CerlettiMonica AnselmiChiara ConchioneMónica AznarChiara ConsiglioMonica BianchiChiara CortiMonica DutcasChiara CorvinoMonica GallegoChiara DonfrancescoMonica GalloChiara GrudenMónica Gutiérrez OrtegaChiara IacovelliMonica Kazlausky EsquivelChiara LoriniMonica LaBargeChiara Maria MottaMonica MaltaChiara MartiniMónica Martínez-GómezChiara MocenniMonica MazzucatoChiara OppiMonica PalmaChiara PagliariMónica Perez-RíosChiara PalmisanoMonica PivettiChiara PazzagliMónica Ríos SilvaChiara PosarelliMonica Sorina LickerChiara RolleroMónica SousaChiara SacchiMonica SummersChiara SaraciniMonica SwahnChiara SorberaMonica TarceaChiara TurchiMónica Taveira PiresChiara VeneroniMónica TeixeiraChiara VillaMónica Teresa González-RamírezChiara VisentinMónica Viñarás AbadChia-Wei ChengMonica WhentChia-Yu ChenMónika AndokChi-Chang HuangMonika BiryloChi-Chang LiuMonika BorkowskaChi-Chuan WangMonika BronkowskaChie KogaMonika BurzynskaChie ObuchiMonika CzajaChie-Chien TsengMonika DerrienChieh-Lu LiMonika Elżbieta MachoyChieh-Yu LinMonika GarbowskaChien Chung HuangMonika GebskaChien Hua TsengMonika GrygorowiczChien Hung YehMonika JakubusChiencheng JungMònika Jiménez-MoralesChien-Cheng JungMonika Klinkhammer-SchalkeChien-Hsieh ChiangMonika Łopuszańska-DawidChien-Huei HsuMonika M. Polczynska-BletsosChien-Hung LeeMonika MattChien-Liang LinMonika MűllerováChien-Lung ChanMonika ParchomiukChien-Ming ChaoMonika PechhackerChien-Pang LeeMonika PiątkowskaChien-Shu TsaiMonika RanitiChien-Tai HongMonika RomanChien-Te LeeMonika SemwalChien-Yi ChenMonika SkowrońskaChien-Yu LinMonika TrojanowskaChih JunMonika TrząskowskaChih-Cheng YangMonika WawrzkiewiczChih-Chin HsuMonika Wieczorek-KosmalaChih-Chin LiangMonika ZdebChih-Chin YangMonique MokhaChih-Ching ChangMonokesh Kumer SenChih-Chung ChenMonserrat Termes-RiféChih-Da WuMontserrat Andrés-VillasChih-Hao ChenMontserrat Corral VarelaChih-Hao LinMontserrat GómezChih‐Hao ShenMontserrat Gómez-de-Terreros-GuardiolaChih-Hao YangMontserrat Pulido-FuentesChih-Hsin LeeMontserrat ReyesChih-Hung GuoMonzurul RoniChih-Hung KoMoonKi ChoiChih-Jen LeeMoon-Soo ParkChih-Jung YehMoramay Lopes-AlonsoChih-Ling LiouMoran PollacChih-Ming LinMorana KoludrovicChih-Wei PaiMorandi BenedettoChih-Wen WuMorena PetriniChih-Yang HuMoreno BaruffiniChihYao HouMoreno UrsinoChikako HondaMorgan AnderssonChi-Kin LawMorgan N. ClenninChing Torng LinMorgan T. JonesChing-Hua HsiehMori TaikiChing-Huang LaiMoritoshi IwagamiChing-Jin ChangMorton LippmannChing-Yao HuMosavi AmirChing-Yeh TsaiMoshe GophenChing-Yi TsaiMoshe Rav AchaChing-Yi WuMoslehpour MassoudChin-Hsuan LiuMostafa E. ShahenChi-Nien ChenMostafa ElgendyChin-Ling ChenMostafa HaghiChin-Lon LinMostafa Saidur Rahim KhanChinmay V. TikheMostafa WalyChinshan HoMotofumi SuzukiChin-Shiuh ShiehMotohide MiyaharaChintan GandhiMotohiro OkadaChinthaka PremachandraMotomu NakashimaChin-Yu LinMotoshi HiratsukaChioma Sylvia OkoroMoudatsou MariaChip HahnMouhcine TallakiChiranjibi SitaulaMouheb ChebbiChiranjit GhoshMouli RamasamyChisom OdohMoustafa HusseinChitrala Kumaraswamy NaiduMo-Yeol KangChiu-Ming HoMpho MoleteChiung Yao ChenMrudula PatelChiung-Ju LiuMucahid Mustafa BayrakChi-Yo HuangMuck-Seler DoroteaChjeng-Lun ShiehMudasir A. KhandayChloe BedardMudassir AnwarChloe Meng JiangMuddasar NaeemCho Kwong Charlie LamMuddassar SarfrazChokri KooliMudit MishraChona HannahMudit TyagiChong ChenMuhammad Abdul QayyumChong-Hwan SonMuhammad Adil RehmanChongming WangMuhammad AkramChongyu DangMuhammad Ali InamChoong Hyun KimMuhammad Amber FareedChoong-Min KangMuhammad Amer LatifChou-Ping ChiouMuhammad Ashraf JavidChowdhury MoniruzzamanMuhammad AsimChris BrownMuhammad AttiqueChris BusbyMuhammad Bashir KhanChris BuseMuhammad Junaid MunirChris CurtisMuhammad Kashif ShahidChris DeeryMuhammad MohsinChris Fife-SchawMuhammad Nauman ZahidChris GroendykeMuhammad RizwanChris HardingMuhammad SaleemChris JonesMuhammad SiddiquiChris LowbridgeMuhammad Sohail KhanChris PaciMuhammad Sohail ZafarChris PauluMuhammad YasirChris PenlingtonMuhammad ZubairChris RichterMuhammed Sedat SakatChris TangMuhammet UşakChris WalcekMuharrem AkinChris ZajchowskiMukesh Kumar SriwastvaChrista LabouliereMukesh MeenaChristelle GuibertMukesh SoniChrister AhlströmMulazim Hussain AsimChristiaan G. AbildsoMulyoto PangestuChristian AuerMunawir MunawirChristian BrinkmannMuneyuki MiyagawaChristian CavéMunjae LeeChristian EhrnthallerMurad HarashehChristian FrancoMurad KhanChristian Garcia CalavaroMurali DamaChristian HirschMurali Mohan CheepuChristian J. Cabello-AlvaradoMuralikannan MaruthamuthuChristian KaczmarekMuriel Ramírez-SantanaChristian KaunertMurod IsmailovChristian KingMurugan PattusamyChristian KonradsMurugan SubbiahChristian KrägelohMuruganandan ShanmugamChristian Lars PolteMustafa Al AukidyChristian MackeMustafa JalalChristian NapoliMutahira LoneChristian OltraMuthaiah ShellaiahChristian RaffelsbergerMuthoni MathaiChristian ReichMuthu ThiruvengadamChristian RitzMu-Xing LinChristian RoquesMwazvita T. B. DaluChristian SchachMya Myat Ngwe TunChristian ThomaMykolas Simas PoškusChristian VoirolMyles CapstickChristian WinterMyo Nyein AungChristian WöberMyoung-Ho HyunChristiana MystriotiMyoungjin KwonChristiane HassenrückMyoung-Sook LeeChristiane SchefflerMyriam Alvariñas-VillaverdeChristiane StockMyriam Guerra-BalicChristian-François RoquesMyriam RudazChristina DerksenMyrna M. T. De RooijChristina EmmanouilMyroslav SprynskyyChristina GentileMyrto KasioumiChristina GyörkösMyung Bae ParkChristina HeemskerkMyung Ja KimChristina LeysonMyung-Chul KimChristina PaxmanMyung-Gwan KimChristina Socias-MoralesMyungkeun SongChristina TassorelliMyung-Soon KwonChristina WhitehouseMzwandile Andi MabhalaChristina ZorbasN. Andrew PetersonChristine BerberichN. C. HuntChristine BrombachN. Rich NguyenChristine FerronN. Yu StepanovaChristine GrafNaba Raj AmgainChristine J. Band-SchmidtNabil EbraheimChristine KriegerNacef TaziChristine LoftusNađa BeretićChristine Lux‐BattistelliNada SheblChristine M. MermierNadav RappoportChristine Manlai KwanNadeem AkhtarChristine Maria SchwarzNadège RochatChristine ParksNader AldewikChristine St LaurentNadezda KirillovaChristine TaylorNadezhda ShmelevaChristine Tessele NodariNadezhda SivrikovaChristmal ChristmalsNadezhda Yuilevna StepanovaChristoph BiehlNadia CattaneChristoph BorzikowskyNadia LiottoChristoph BuchtaNadia NajafiChristoph BührenNádia P. KozievitchChristoph BührerNadia RaniaChristoph CentnerNadia SelimChristoph ClephasNadim AlmoshmoshChristoph HintermüllerNadim HaboubiChristoph JochumNadine TramowskyChristoph LechnerNadinne RomanChristoph LeonhardNadja KlafkeChristoph N. SeubertNadja MachadoChristoph ScopeNadzeya LaurentsyevaChristoph StachNaeem KhanChristoph StallmannNagahisa HirayamaChristoph StrumannNagalingam RajakumarChristoph ZinnerNageswara PilliChristophe ChesneauNagisa SugayaChristophe MonninNagy HenriettaChristophe WaterlotNahal HabibiChristopher A. WasNahanga VerterChristopher B. CaliffNahia Idoiaga-MondragonChristopher B. TaberNahid VatanpourChristopher BallmannNahum Medellín-CastilloChristopher BarnesNa-Hyeon (Hannah) KoChristopher E. AndersonNaiara BilbaoChristopher F. SharpleyNaiara DemnitzChristopher FangNaiara OzamizChristopher G. WilsonNai-Hua ChenChristopher GaffneyNai-Jen ChangChristopher Holzmann-LittigNaira DelgadoChristopher HurrenNaji KharoufChristopher J. WorsnopNakata YoshioChristopher J. YoungNalini Sooknanan PillayChristopher John LundstromNam Deuk KimChristopher John NeufeldtNam Il UmChristopher Keith HaddockNamhee KimChristopher KentNamhyun KimChristopher KirkNamkwen KimChristopher KobierzyckiNam-Seok JooChristopher KotarskyNan CuiChristopher L. AtkinsonNan JiangChristopher L. SkeelsNan LinChristopher LabosierNan WuChristopher LatellaNan Xu RattanasoneChristopher LoNan YangChristopher Mark HillNan ZhangChristopher OwensNana Amoah-RameyChristopher P. WhiteNancy Agmon-LevinChristopher R. SnellNancy ClarkChristopher W. CunninghamNancy DoetzelChristopher W. CutlerNancy HelouChristopher WeyhNancy Long SieberChristopher ZuidemaNancy McNaughtonChristos ArgyropoulosNancy PurdyChristos K. KontosNanda Kishore RouthuChristos LionisNandkishor M. DhawaleChristos PezirkianidisNan-Hung HsiehChristos SymeonidesNao SugikiChristy BridgesNaoki KanoChristy GreenleafNaoki NakagawaChrysi MetallidouNaoki SudoChuang-Yuan ChiuNaoko ArakawaChueh-Hung WuNaoko HikitaChueh-Lung HwangNaomi JulianaChuks Eresia-EkeNaomi TamuraChukwuma NnajiNaoro TanakaChul Sue HwangNaoya HasegawaChul-Hyun ChoNapoleón Navarro-TitoChul-Su YangNarayan Babu DhitalChu-Lun HsiehNarayana RajuChulwon KimNarayanappa AmrutaChun LiangNarcis BârsanChun Sing LaiNarelle EatherChun-Bae KimNaresh KumarChunchia ChengNarimasa KumagaiChun-Chieh KaoNarrendar RaviChandranChung Gun LeeNarumi KojimaChung Yi LiNaser AlsharairiChung-Ho E. LauNaser GolsanamiChungho LauNasim HajariChung-Jen HuangNasir MushtaqChung-Jen WangNasro Min-AllahChung-Te ChangNasser EbrahimChung-Tung J. LinNasser NajibiChung-Ying LinNasser RezzougChun-Hong LiuNassib B. BuenoChunhong ZhaoNastaran ManouchehriChun-Hou WangNatacha Jesus-SilvaChun-Hsin ChenNatale CanaleChun-Hung ChangNatale Salvatore BonfiglioChun-Jung ChenNatalia Albaladejo-BlázquezChunki KimNatalia AversanoChun-Por Wong WongNatalia Balague SerreChunsen WuNatalia BaulinaChunyi WenNatália BruckerChun-Yin YehNatalia Burgos AlonsoChunyu WangNatàlia Cantó MilàChu-Shiu LiNatalia Cezon-SerranoChutima JalayondejaNatalia ChechkoChu-Yu HuangNatalia DistefanoChyan-Long JanNatalia DłużniewskaChyi-In WuNatalia DrabińskaCid André Fidelis De Paula GomesNatalia D’SouzaCindy ChangNatalia Duque-WilckensCindy TraboulsiNatalia El-MerhieCintia Chamorro-PetronacciNatalia FumagalliCintia Micaela Chamorro-PetronacciNatalia GusmerottiCíntia PêgoNatalia Hernández-SeguraCinzia BerteaNatalia KascakovaCinzia Di MonteNatalia Kaźmierczak-WojtaśCinzia MarinaroNatalia KizilovaCinzia MasperoNatalia LauferCinzia NovaraNatalia Martín MaríaCinzia RienzoNatalia MazanowskaCiro Di NunzioNatalia Morgulec-AdamowiczCiro José Jardim De FigueiredoNatalia PawlasClaire BadenhorstNatalia RingblomClaire BlewittNatalia RiveraClaire BossennecNatalia Romero-FrancoClaire De BisschopNatalia RovellaClaire GoodfellowNatalia RyczekClaire L. MeekNatalia SabelnikovaClaire McCartanNatalia Saldaña-GarcíaClaire RooneyNatalia ShartovaClaire RostronNatalia SobolevaClaire TournyNatalia Solano PintoClaire WilsonNatalia StachowiakClara Inés Pardo MartínezNatalia SuárezClara López-MoraNatalia SzejkoClara Martinez-PerezNatalia V. YakovenkoClara SimãesNatalia WagnerClare BrownNatalia Wawrusiewicz-KurylonekClare GilbertNatalie BrownClare McKeaveneyNatalie EdelmanClare RoscoeNatalie EinClarissa BuenoNatalie ElkheirClarisse BrígidoNatalie EnninghorstClark FreifeldNatalie LandeckClark LundbergNatalie M. LeblancClaude ScheuerNatalie MichaelsClaudia A. ZaniniNatalie MuellerClaudia AlbericoNatalie SampsonCláudia AugustoNatalie SchwatkaClaudia Barros CoelhoNatalie SuffClaudia C. Calzada-MendozaNatalie Watson-BrownClaudia CampanaleNatalija LepkovaClaudia Campillo-CoraNataliya ChukhrovaClaudia CarlucciNataliya UshevaClaudia CarmassiNatalja FatkulinaClaudia CavaNatalya A. KulikovaClaudia ChiarolanzaNatalya A. ShnayderClaudia ClausNatalya GlushkovaClaudia CostaNatarajan RanganathanClaudia CovucciNatasa ZenicClaudia CurciNatascia RinaldoClaudia Daniele De SouzaNatasha BrisonClaudia DuranNatasha FranklinClaudia GallertNatasha LaytonClaudia GiacomozziNatasha ReidClaudia Guerrazzi YoungNate BreznauClaudia HiltonNathalia PadillaClaudia HunotNathalie AndréClaudia Iveth Astudillo-GarcíaNathalie GalaisClaudia KitzmüllerNathalie GuriecClaudia LermaNathalie IofridaClaudia Luevano-ContrerasNathalie OrtarClaudia MancaNathalie VermeulenClaudia Mara De Melo-TavaresNathan D. DicksCláudia Maria Ferreira PereiraNathan EddingsaasClaudia MarottaNathan Halsey SchumakerCláudia OliveiraNathan K. EvansonClaudia PieperNathan M. D’CunhaClaudia Pineda-MarinNathan MakowskiClaudia PisanuNathanael James YatesCláudia Regina Furquim De AndradeNathanael OjongCláudia Ribeiro De AlmeidaNathaniel GeyerClaudia RusNathaniel J. SzewczykClaudia SarduNathaniel Parker WestonCláudia Sirlene OliveiraNatsumi FujiwaraClaudia SomNavarro FerronatoClaudia TomozeiNavarro Gómez NoeliaClaudia TraunmüllerNaveed Iqbal RajaClaudia TrombettaNaveed SalmanClaudia TucleaNavin KaushalClaudimar Pereira Da VeigaNavneet AujlaClaudina NogueiraNavneet Kaur BaidwanClaudine FabreNayanjot Kaur RaiClaudine IrlesNayoung AhnClaudinei De Souza GuimarãesNayra Anna Martin-KeyClaudio Andre Barbosa De LiraNazareth Martínez-HerediaClaudio AzzoliniNazli KoseogluCláudio Daniel CerdeiraNdidzulafhi Innocent SinthumuleClaudio Di LoritoNeal BarnardClaudio FarinaNeal DoranClaudio GambardellaNeamtu BogdanClaudio GariazzoNebojša PetrovićClaudio Hernández-MosqueiraNectarios VidakisClaudio MirarchiNedim DurmusCláudio ParenteNeelima SatyamClaudio RobazzaNeema MoravejiClaudiu BoceanNeeraj BhandariClaudiu George BoceanNeeraj GillClaudiu MorgovanNeeru GuptaClaus NormannNefta-Eleftheria P. VotsiCleber Nunes KrausNeftali NuñezClémence PerraudinNegar OmidakhshClemens FuchsNegin A. RiaziClément GinouxNeha MehtaClément PimouguetNeha RathiClementine LabroscianoNehama LewisClemon GeorgeNehreen MajedClifford BersamiraNeil BirdClifford GoodmanNeil ColesClifton AddisonNeil OldfieldClifton HolmesNektarios KaranikasClifton J. HolmesNellie TsipouraClifton P. Bueno De MesquitaNelly Molina-FrecheroClinton WebbNelson A. De La Pena AtehortuaClive PalmerNemanja BerberClive RichardsonNemesio Villa-RuanoClodagh ToomeyNenad StevanovicClyde MartinNenita BukaloCodruta Adina BaltescuNeomi Vin-RavivColeen E. TorontoNerea Gutiérrez-FernándezColette LangosNerea Jiménez-PicónColette SmartNerijus BlažauskasColin DrummondNeringa Vilkaite-VaitoneColin EslerNermin HasanspahićColin MulliganNesta Bortey-SamColleen AldousNeven PapicColleen DoakNeven RicijašColleen Jayne SaundersNevena KulicColleen M. GallagherNeves-Amado JoãoColleen McLaughlinNeville SweijdConan McCaulNevo MargalitConcepción Blasco-RosNgaio RichardsConcepción Pérez-LamelaNgar Sze Elsa LauConcepción Soto-VidalNgianga-Bakwin KandalaConcetta PirontiNgueleu ArmelleConcetta PirroneNguyen Minh DucConcetta PolizziNiamh CoffeyConcetta RafanielloNiamh Ní BhroinConcettina FengaNibedita PatelConcetto Paolo VinciNicholas BorcherdingCong GaoNicholas CaporussoConghong HuangNicholas ChilesheConghua WenNicholas D. LawsonConn RotherNicholas FournierConnar McShaneNicholas J. HansonConnie M. WeaverNicholas K. SchiltzConnie SvobNicholas KuzikConor J. O’DeaNicholas LermaConrad SparksNicholas Peter LawsonConsolación Gómez-ÍñiguezNicholas PleaceConstantin Aurelian IonescuNicholas T. BelloConstantino Arce-FernándezNicholas V. RescinitiConstantino BalestraNicholas WiseConstantinos DavosNichole HugoConstantinos NicolaouNichole MorrisConstanza B. Gómez ÁlvarezNick ChandlerConstanze EibNick GoodwinConsuelo ChacarteguNick OsborneConxita MestresNick SlagelConxita Mestres MirallesNicklas Heine StaunstrupCoral Oliver-HernándezNicklas NeumanCord M. BrundageNickolas M. JonesCordelia Estevez-CasellasNico DraganoCordula WernerNico NagelkerkeCorinna FranklinNicodemus KitukuthaCorinna GeislerNicola A. ValenteCorinna ReaNicola Alberto ValenteCorinna WalshNicola ArmstrongCorinna Weber-SchöndorferNicola CanciCorinthias P. M. SianiparNicola CapolupoCornelia FiesslerNicola CaroneCornelie Nienaber-RousseauNicola ChristieCorrado AngeliniNicola De AngelisCorrado MagnaniNicola DivianCorrado PaganelliNicola DöringCorrea-de-Araujo RosalyNicola EpicocoCortés-Castell ErnestoNicola FaccilongoCory R. MartinNicola GentiliCosima Damiana CalvanoNicola GrayCosima PrahmNicola KimeCosimo BruniNicola LambertiCosimo PalagianoNicola LovecchioCosimo SpertiNicola Luigi BragazziCoskun Joe DizmenNicola MagnavitaCosmin VanceaNicola MarottaCosta EduardoNicola MarranoCostantino BalestraNicola Massy-WestroppCostantino SircaNicola MaureaCostanza Maria CristianiNicola ModugnoCostas S. ConstantinouNicola MondanelliCostas VarotsosNicola MontemurroCostin D. UntaroiuNicola MucciCourteney Leigh BenjaminNicola NanteCourtney A. MarshNicola PalenaCourtney DowNicola PrannoCourtney Welton-MitchellNicola RaimoCraig A. HorswillNicola RelphCraig Aaen-StockdaleNicola SambucoCraig AndersonNicola SantoroCraig CarpenterNicola SponsielloCraig D. WorkmanNicolae GicaCraig DepkenNicolae MironCraig DonnachieNicolai CraciunCraig HedbergNicolai DenzinCraig PearlNicolas BergerCraig SinclairNicolas DugréCraig TalmageNicolas MarineCraig WilkieNicolas PaulCrenguta Ileana SinisiNicolas RayCringu Antoniu IonescuNicolás Roberto Robles Perez-MonteolivaCrisanta-Alina MazilescuNicolas TurpinCristhian DuranNicolas VignaisCristi SpulbarNicolas VignierCristian BalducciNicolas-Alexander TatlasCristian CariniNicole A. HoffCristian Castillo OleaNicole BarraCristian CiureaNicole CasaliCristian DeanaNicole Cieri-HutchersonCristian FrancavillaNicole DeVilleCristian FurauNicole DitchmanCristian LieneckNicole GumportCristian Marín-PagánNicole Iroz-ElardoCristian Martínez-SalazarNicole IvesCristian Molla EsparzaNicole LandCristian MoralNicole LevitzCristián OyanadelNicole NguenhaCristian ScheauNicole RedversCristian StătescuNicole ReighCristian TimmermannNicole Roberta GiuggioliCristiana ContiNicole RübsamenCristiane BatistelaNicole S. N. YiuCristiane Helena GallaschNicole TraboldCristiano CarbonelliNicoleta ChiraCristiano FaraceNicoleta VrînceanuCristiano FidaniNicoletta De SantisCristiano PesaresiNicoletta González-CancelasCristiano ScandurraNicoletta GuerrieriCristina A. Huertas AbrilNicoletta SetolaCristina Anna Maria Lo IaconoNicoletta VegniCristina AntinozziNicolette I. Teufel-ShoneCristina BaglivoNicolle TulveCristina BaixinhoNicolò ColombaniCristina BaraleNicolò IacuzziCristina Bianca PocolNida AslamCristina Bota-AvramNidhi SinghCristina Burrola-AguilarNieves MoyanoCristina CalejaNigel BeebeCristina CarvalhoNigel D. RobbCristina CasalouNigel GoodmanCristina Castejón-RiberNigel Nigel L. WilliamsCristina Ciuluvica (Neagu)Nigel YoccozCristina CivilottiNiharika R. SamalaCristina CoutoNiina BhattaraiCristina Del CampoNiina JunttilaCristina Di TeccoNik StoopCristina Dos Santos Cardoso De SáNika Brkljača BottegaroCristina Espinosa-SempereNike FrankeCristina Fernández-PorteroNikhil SatchidanandCristina Fernández-RoviraNikita OsintsevCristina ForzatoNikola KomlenacCristina FrangeNikola NaumovCristina Galache OsunaNikolaj TravicaCristina GalvanNikolaos A. ChrysanthakopoulosCristina García AelNikolaos BarmparesosCristina García FernándezNikolaos GavanasCristina García-BravoNikolaos GkantidisCristina GiannattasioNikolaos I. RousisCristina Giménez-GarcíaNikolaos I. StilianakisCristina Gómez-CasadoNikolaos M. PapadakisCristina González‐DíazNikolaos MachairasCristina Guerra-MarmolejoNikolaos MavridisCristina Hanganu-BreschNikolaos MonokrousosCristina Hernández-QuevedoNikolaos PapanasCristina Jorge-SotoNikolaos PellasCristina Liébana-PresaNikolaos PerakakisCristina López CadenasNikolaos SolomakosCristina López De Subijana-HernándezNikolaos StamatisCristina M. Beltran ArocaNikolaos VlachadisCristina Maria Beltran ArocaNikolaos ZarasCristina MaroldaNikolas A. S. ChottaCristina MartinsNikolas KantasCristina Martin-SabrosoNikolay SolovyevCristina Mas-BarguesNikoleta PrintzaCristina MazzaNikolett SzeleiCristina MenescardiNikolina Jukić PeladićCristina MennittiNikos AndritsosCristina MerlaNikos AntonopoulosCristina MollicaNikos BarkasCristina MonteiroNikos FatourosCristina MontiNikos KapitsinisCristina MuñizNikos NikolettosCristina NoriegaNikos ViazisCristina NunesNilaksh GuptaCristina PeñaNilakshi BaruaCristina Pérez CorbellaNilakshi T. WaidyatillakeCristina Petisco-RodríguezNilesh PatelCristina PetrucciNilesh WashnikCristina Rey-ReñonesNiloofar OrdouCristina Rivera-PicónNils EnglerCristina Romero-BlancoNils HaneklausCristina Sánchez-RomeroNils LudwigCristina Senin-CalderonNils TjadenCristina Sotomayor-CastilloNilton AlvesCristina SousaNilufeur McKayCristina TeixeiraNilza CostaCristina Toca PérezNima Afshar-MohajerCristina TruzziNima GharahdaghiCristina VassalleNima SayyadiCristina Velasco VegaNimish GuptaCristina-Mihaela LăcătușuNimit AgarwalCristine D. DelnevoNina BausekCristóbal Macías VillalobosNina DrejerskaCristoforo ComiNina IshorstCristoforo PomaraNina IvanovskaCsaba CenteriNina ReinholtCsaba KorenNina TsydenovaCsanád SzabóNina TumosaCudore L. SnellNina VøllestadCui GuoNina ZhuCuilan LiNina-Katri GustafssonCuiqing ZhongNing DingCurtis FennellNing QuCynthia BlantonNing-Kuang ChuangCynthia E. MuñozNirah ShomerCynthia F. CorbettNiraj Prakash JoshiCynthia HadenfeldtNirit Putievsky PilosofCynthia IsleyNirmala Thomas MyersCynthia K. MaupinNisha Mukherjee BellingerCynthia WanNisha NaickerCynthia WilkesNishan Kumar BiswasCyprian KozyraNishel Mohan ShahCyril GenevoisNishimura TomoyoshiCyril NicolasNitika KumariCyro AlbuquerqueNittari GiulioCyrus MotamedNivaldo T. SchieflerCzesław GiemzaNívea Adriana Dias PonsDa Yang TanNlandu NgatuDaan F. OostveenNnaemeka OdoDa-An HuhNnamdi NnoliDaan KamphuisNneji Lotanna MicahDacia Dalla LiberaNoboru KanekoDaegyu YangNoboru MotomuraDaekeun KimNobuaki HamazakiDae-Ki KangNobuaki MichihataDae-Kwang KimNobuaki MoriyamaDafna HalperinNobuaki TottoriDag Øivind MadsenNobuhiko SuganumaDagfinn MatreNobuhiko TanakaDagmar ČámskáNobukazu FujimotoDagmar PavluNobuo YoshinariDagmar PavlůNobutaka KuriharaDagmara Budnik-PrzybylskaNobuto NakanishiDagmara I. Struminska-ParulskaNoel LeighDagmara MigutNoelia BajalesDagmara Mirowska-GuzelNoelia Carbonell BernalDa-Hong WangNoelia Fernández-RoucoDai PuNoelia González-GálvezDaiana A. Quintiliano ScarpelliNoelia María Martín EspinosaDaiana Priscila Rodrigues-de-SouzaNoelia Zagalaz-AnulaDaichi SugawaraNoemí Ávila ValdésDaichi YamashitaNoemi FaeddaDaiji NagayamaNoemi GiannettaDaisuke KatoNoemí López-EjedaDaisuke MachidaNoemi Serra-PayaDaisuke NonakaNoemia Teixeira De Siqueira-FilhaDaisuke SatoNofel LagrosasDaisy Aparecida Do Nascimento RebelattoNojan AliahmadDaiva JurevicieneNolan HerssensDajana Kučić GrgićNomfundo MoroeDajun QinNonhlanhla TlotlengDak KopecNor Azlida Mohd NorDalal Hammoudi HalatNor Kamaliana Binti KhamisDalbert Matos MascarenhasNora EstgfaellerDale A. DickinsonNora Repo-SaeedDale ChapmanNora Suleiman-MartosDale L. ZimmermanNora SydoDale ManteyNorbert BeckerDale Wilson ChapmanNorbert QuadfliegDaleen Van Der MerweNorbert SiposDalia PerkumienėNorbert TripoltDalia SmailienėNorbert TuśnioDalia StreimikieneNorberto QuilesDalila BurinNoreen BreakeyDalila ScaturroNoreen GleesonDalius JatužisNoreen RossiDaluwathu Mulla Gamage PreethichandraNoriaki MaedaDami KoNorihiko NaritaDamian BorysNorihiro NishidaDamián Fernández RodríguezNorio YamamotoDamián Iglesias GallegoNoritaka KatataniDamian PiotrowskiNoriteru MoritaDamian ZasinaNoriyuki MatsukawaDamiano FormentiNorm ArcherDamiano MeninNorma De PiccoliDamien BonnardNorma G. Padilla-EguiluzDamien CateauNorma P. TavakoliDamien McElvennyNorman TempleDamien PeyronnetNorman Van RhijnDamien VitielloNorzagaray Campos MarianoDamien YoungNounagnon AgbanglaDamir ZubacNoureddin Nakhostin AnsariDan Frederick OrchertonNoureddine BouaïchaDan JonesNourtan AbdeltawabDan KassNowicki AndrzejDan Lawson CrouseNuan Ping CheahDan RobbinsNúbio Gomes FilhoDan RomerNuno CoutoDan TchernovNuno CrespoDan VickNuno Domingos GarridoDana AlonzoNuno DurãesDana BadauNuno FernandesDana Carmen ZahaNuno GarridoDana DlouháNuno LoureiroDana Gabriela FestilaNuno Manuel Sessarego Marques Da CostaDana LawrenceNuno MoraisDana Mowls CarrollNuno V. BritoDan-Andrei Sitar-TăutNunzia IaccarinoDan-Cristian DabijaNunzia LinzaloneDane WolfNunzia NappoDangdang ShaoNupur VermaDanguole SatkunskieneNuray DemirelDani MorenoNúria AlmironDanica MichaličkováNúria Casquero-ModregoDaniel A. BoullosaNuria CodinaDaniel A. GriffithNuria Novas CastellanoDaniel Adrian LunguNuria Padrós-FloresDaniel B. SchwabNurudeen Olalekan OlosoDaniel BadulescuNurul Hawani Binti IdrisDaniel BellNury Pérez-HernándezDaniel BoatengNuttanan WichitaksornDaniel BorgesNuttavikhom PhanthuwongpakdeeDaniel BoscaljonNuttida RungratsameetaweemanaDaniel Collado-MateoNyoman SutarsaDaniel CombsO. R. AndersonDaniel CruzOak-Hee ParkDaniel CuestaOana Adriana GicaDaniel DeimelOana AntalDaniel DemantOana BlagaDaniel DuneaOana Cristina PopoviciDaniel DziobOana SăndulescuDaniel Eugeniu CrunteanuObasesam OkoiDaniel FerrándezOctavian G. DuliuDaniel Ferreira PolóniaOctavian GrozaDaniel G. CooperOctavian MoldovanDaniel García IglesiasOctavian NeagoeDaniel GartnerOctávio SacramentoDaniel GaudetOctavio Vázquez-AguadoDaniel GonzálezOdeya CohenDaniel Gónzalez-BandalaOfir KatzDaniel GriffithsOgcheol LeeDaniel HindmanOhsawa SuguruDaniel Ian McSkimmingOimahmad RahmonovDaniel J. MallinsonØivind SkareDaniel J. ReidenbergOk Hyung NamDaniel JauslinOkechukwu ChukwudehDaniel JérezOksana Seroka-StolkaDaniel JuarezOksoo KimDaniel KaszubowskiOladele OgunseitanDaniel KlineOlaf JensenDaniel KönigOlaf Johan Hartmann-JohnsenDaniel KronenbergOlaf ZagolskiDaniel L. MendozaOlaia Lucas-JiménezDaniel LiberackiOlaia Martínez GonzálezDaniel LindsayOlajide O. FadareDaniel LloretOlatunde AremuDaniel LópezOlatunde S. DurowojuDaniel López-LópezOlawale FatokiDaniel López-PlazaOlchowik GrażynaDaniel M. CookOle Christian LindDaniel M. González-PérezOle WintherDaniel M. MasekameniOleg KaradutaDaniel MaposaOleg MatusovskyDaniel MauvoisinOlegas NiakšuDaniel MendozaOlena Shelest-SzumilasDaniel MontOlena TaratulaDaniel MorenoOlga A. ShvetsovaDaniel Nadal-SalaOlga Alexandra Chinita PirrolasDaniel Navas-CarrilloOlga De Cos GuerraDaniel NeunhäusererOlga DergachevaDaniel OlsenOlga Di FedeDaniel P. CassidyOlga DługoszDaniel P. JohnsonOlga GolubnitschajaDaniel P. PotaczekOlga Gomez-OrtizDaniel Patiño-GarcíaOlga IvanovaDaniel RábagoOlga JarrínDaniel RaffertyOlga JensenDaniel S. Y. HoOlga KalininaDaniel Sanchez-RodasOlga ŁawińskaDaniel Sanchez-TaltavullOlga Lopez TorresDaniel SchellerOlga MaganoDaniel SchulzeOlga María Alegre De La RosaDaniel SkinnerOlga MitinaDaniel ŚlęzakOlga N. BerdinaDaniel StjepanovicOlga PilipczukDaniel StoecklinOlga PulidoDaniel SzejbaOlga RedinaDaniel TerryOlga Roldan ReoyoDaniel TorassaOlga SerradellDaniel Torres-LagaresOlga StrizhitskayaDaniel UrbánOlga TararovaDaniel ValleroOlga V. EfremenkovaDániel VéghOlga ZubkovaDaniel W. NixonOlihe N. OkoroDaniela Acquadro MaranOlimpio MonteroDaniela BoccoliniOliver CalliesDaniela CaccamoOliver HaydenDaniela Caetano GonçalvesOliver Mendoza-CanoDaniela Cajado-CarvalhoOliver NiebuhrDaniela CialfiOliver Ramos ÁlvarezDaniela CilloniOliver SchierzDaniela CraveiroOliver WeigeltDaniela CurtiOlivia KungumaDaniela CzernochowskiOlivia LopezDaniela De Melo E. SilvaOlivia RemesDaniela DeboneOlivier AromatarioDaniela DoegeOlivier CottencinDaniela DumbrăveanuOlivier DellisDaniela HaluzaOlivier HuckDaniela IsolaOlusola OlagokeDaniela L. GomesOlusolao OloladeDaniela L. StanOluwagbemiga DadeMatthewsDaniela LoconsoleOluwatoyin OlukotunDaniela MariOluwole A. SoyinkaDaniela Maria MateiOluyemi ToyinboDaniela MartiniOlvar BerglandDaniela MeloOlympia E. AnastasiouDaniela MometeOmanjana GoswamiDaniela MorniroliOmar AlmohammedDaniela Muntele HendreşOmar Al-TabbaaDaniela OehringOmar Amador-MuñozDaniela PacellaOmar DurrahDaniela Pia Rosaria ChieffoOmar Guzman-QuevedoDaniela PicciottoOmar HahadDaniela PierannunzioOmar HamarshehDaniela R. RecchiaOmar JanehDaniela ŠincekÖmer ElmaDaniela SmirniOmer UnsalDaniela TarnitaOmid AkhavanDaniela TestoniOmni CassidyDaniela ViscontiOmolade Femi-AjaoDaniela ZamfirOmorogieva OjoDaniele CafollaOndrej JešinaDaniele CavaleriOndrej KodetDaniele CecconetOndrej PesoutDaniele ChiffiOnno BouwmeesterDaniele Della LattaOpeyemi BabatundeDaniele DetanicoOran KwonDaniele FattoriniOrestis PapakyriakopoulosDaniele GarcovichOrhan KoçakDaniele GiansantiOriana MottaDaniele La ForgiaOriol Abellan-AynesDaniele LandiOriol Cantó-NavésDaniele MancardiOriol Iborra-EgeaDaniele MasaroneOriol Turró-GarrigaDaniele MollaioliOrit UzielDaniele PeanoÖrjan B. EkblomDaniele PironiOrkideh GharehgozliDaniele PiscitelliOrlanda CruzDaniele RonsivalleOrlando VaselliDaniele SofiaOrly SaridDaniele UrsoOrna Braun-LewensohnDaniele VeclaniOrnella PunzoDaniele VergaminiØrnulf SeippelDaniel-Eduard ConstantinOrsolya Kegyes-BrassaiDaniel-Ioan CuriacOrsolya NémethDaniella Campelo Batalha Cox MooreOrsolya VargaDanielle CarlinOrtal SlobodinDanielle DavisOsama EljamalDanielle E. DyeOsamu KumanoDanielle GreenbergOsamu KushidaDanielle H. TaylorOsamu UchidaDanielle HitchOsamura NishimuraDanielle NesbittOscar AlfrancaDanielle OsborneOscar ArroganteDanielle PhilibertOscar Arteaga HerreraDanielle R. EugeneOscar De La Torre-TorresDanielle R. EvangelistaOscar FernándezDanielle Regina Da Silva GuerraOscar GalagarzaDanielle TometichOscar GarcíaDanijel IvajnšičOscar García-GarcíaDanijela Puric-MladenovicÓscar Gavín ChocanoDanijela Ristić-MedićOscar Hengxuan ChiDanila De VitoOscar Hernández-HernándezDanilo BrozovićOscar Martinez De QuelDanilo IannettaOscar Martínez-PérezDanival José De SouzaOscar Núñez EnríquezDanny OttoOscar PrietoDanúbia Da Cunha De Sá-CaputoÓscar Rapado-GonzálezDanuta GajewskaÓscar Rodríguez-NogueiraDanuta LeszczyńskaOscar Salomó-CollDanuta PirógÓscar ZurriagaDanuta Proszak-MiąsikOseweuba Valentine OkoroDanuta Szkutnik-FiedlerOskar JonssonDanuta SzpilkoOsman UlviDanuta UmiastowskaOsmano OasiDany Perwita SariOsmel La-Llave-LeonDanyelle GreeneOsmindo Rodrigues Pires JúniorDaodao HuOsnat BashkinDapeng LiOsnat KeidarDaphna Birenbaum-CarmeliOsnat Roth-CohenDaphne CheungOssi PesämaaDaphne KaklamanouOstrowski AndrzejDaquan HuangOsvaldo FaillaDara LeungOsvaldo MartinezDaria BylievaOswaldo MorenoDaria ChmielewskaOswaldo Villena CarpioDaria SikorskaOtakuye Conroy-BenDarija Lušić VukićOthmar MoserDarin ZertiOtilia CulicovDario BruzzeseOto PotlukaDario Di GiuseppeOttavia Eleonora FerraroDario Di NardoOtto LamDario MonzaniOtto LenhartDario NovakOtto RuokolainenDario PadovanOuahiba LaribiDario SiniscalcoOurania TremmaDariusz BoguszewskiOvidiu Popa-VeleaDariusz ButrymowiczOvidiu ȘerbanDariusz GraczykOvidiu TitaDariusz KałkaOxana M. DrapkinaDariusz KotlegaOyelola AdegboyeDariusz KrokOzcan EsenDariusz KucharczykÖzkan UǧurluDariusz KuszP. PrzygodzińskiDariusz ŁątkaP. V. ViswanathDariusz MilewskiPablo A. Cantero-GarlitoDariusz MlynarczykPablo A. LizanaDariusz NowakowskiPablo Blanco Martínez De MorentinDariusz PawlakPablo CaballeroDariusz SuszanowiczPablo Caballero BlancoDariusz TimlerPablo Camacho LazarragaDariusz TworzydłoPablo Cervera-GarviDariusz WieckowskiPablo Cienfuegos-SuárezDariusz WłodarekPablo EspinosaDariusz Wojciech MazurkiewiczPablo FaríasDarja JarosovaPablo Fernández De ArroyabeDarjan KarabasevicPablo Fernández-AriasDarko AntičevićPablo Gaitán-RossiDarko DimitrovskiPablo Galan-LopezDarko MekterovicPablo Galvez RuizDarla CastelliPablo José Olivares-OlivaresDarlene CavalierPablo K. ValenteDarpan ChakrabortyPablo LunaDarrell AbernethyPablo Martinez-CamblorDarrell P. WheelerPablo Monteagudo ChinerDarrell SteffensmeierPablo Olivares-OlivaresDarrell TanPablo Román LópezDarren DunningPablo RuisotoDarren E. R. WarburtonPablo Ruiz-RudolphDarren RussellPablo S. Luna-LozanoDarren WarburtonPablo Salvador-ColomaDartagnan Pinto GuedesPablo TejedoDaruka MahadevanPablo Tercedor SánchezDaryl Wayne NiedermoserPablo Ugalde-SalasDastan BamwesigyePablo Usán SupervíaDave (D. H. H.) Van KannPablo Valdés-BadillaDavid A. B. DancePablo VenegasDavid A. McentirePablo Villalobos DintransDavid A. PerkinsPa-Chun WangDavid A. WinterPaige SafyerDavid Alberto Rodríguez MedinaPal MartonDavid AparisiPalanivel KandasamyDavid Arranz SolísPallav PokhrelDavid AshleyPalma ScolieriDavid B. NewmanPalmira ImmordinoDavid Baeza MoyanoPaloma EscamillaDavid BalayssacPaloma MassóDavid BarneyPaluku BahwereDavid BaronPam ThomasonDavid BellPamayla DarbyshireDavid BerryPamela A. Harris-HamanDavid Bienvenido-HuertasPamela BeachDavid BlanePamela D. SwanDavid BrainPamela Di GiovanniDavid BroomPamela DopartDavid ButlerPamela Durán-DíazDavid C. MohrPamela HazeltonDavid CabaleiroPamela J. SchreinerDavid CarpenterPamela J. StewartDavid CatelaPamela J. TincDavid ChelazziPamela K. StrohfusDavid CingranelliPamela McCoyDavid ClabornPamela SenesiDavid Cobos-SanchizPamela SerraDavid CokerPamela Smith GuerraDavid ColecchiaPamela TozzoDavid ColstonPanagiota GaliatsatouDavid Conde-CaballeroPanagiota SpyridonosDavid CromptonPanagiota XaplanteriDavid De HitaPanagiotis G. HalvatsiotisDavid DolivoPanagiotis GkriliasDavid E. JacobsPanagiotis K. MarhavilasDavid F. UrschlerPanagiotis KaranisDavid Falcón MiguelPanagiotis KorovessisDavid FanfaniPanagiotis KourtesisDavid Freire-ListaPanagiotis MichalisDavid GilPanagiotis PeitsidisDavid GilkeyPanagiotis PentarisDavid GiménezPanagiotis StefanidisDavid HarleyPanagiotis TsimeasDavid HawkesPanayiotis VarotsosDavid HenryPanayotis A. SiskosDavid HerbertPande Putu JanuragaDavid Herrero-FernándezPang-Yun ChouDavid Hung-Tsang YenPankaj AhluwaliaDavid J. CornellPankaj KumarDavid J. LeasaPankaj SharmaDavid Juárez VarónPantaleo GrecoDavid KomatsuPanteleimon GiannakopoulosDavid KrantzPantelis KourosDavid L. MayorPantelis ZempekakisDavid LarsenPanu RantakokkoDavid LawPao-Huan ChenDavid LeanPaola ArdilesDavid LimPaola BertoliniDávid LíškaPaola BordonDávid Lóránt DénesPaola BorellaDavid LucasPaola BorrelliDavid LuquePaola DamianiDavid M. ChambersPaola De LucaDavid M. HowardPaola Ferreira BarbosaDavid M. ManninoPaola GalloDavid MalonePaola GalluzzoDavid MandelbaumPaola Gonzalo-EncaboDavid ManheimPaola GremigniDavid ManninoPaola MarcolongoDavid MartinPaola PalestiniDavid McArthurPaola ParenteDavid McDaidPaola PassafaroDavid McDowallPaola PumaDavid McVeyPaola QuaresimaDavid MhlangaPaola QuatriniDavid MoleroPaola RubilarDavid Moreno MolinaPaola SemeraroDavid N. SattlerPaola SenaDavid NalbonePaola SpagnoliDavid Naranjo-HernándezPaolina CroccoDavid NiederseerPaolo BianconeDavid NolanPaolo CameliDavid OliverPaolo CampioniDavid O’SullivanPaolo CapparèDavid P. HedlundPaolo CarosiDavid Perez NeiraPaolo CastigliaDavid Pérez-JorgePaolo CavorettoDavid Pérez-PascualPaolo ConflittiDavid PerpetuiniPaolo ContuDavid PinedaPaolo CozzolinoDavid PolyaPaolo CrippaDavid PoolePaolo De AngelisDavid Q. AndrewsPaolo DebertolisDavid RhodesPaolo Emilio PudduDavid RohdePaolo Emilio SantoroDavid RollockPaolo Francesco ManiconeDavid S. WeberPaolo GianiDavid SalmanPaolo LauriolaDavid Sancho-CantúsPaolo LeombruniDavid SchultzPaolo MadoniaDavid SchwebelPaolo MaranzanoDavid ShawPaolo Maria LeoneDavid SloukaPaolo Maria RossiniDavid T. RothPaolo MartellettiDavid ThomsonPaolo MeneguzzoDavid TurbowPaolo MontuoriDavid V. McQueenPaolo Nicola BarbieriDavid Varillas DelgadoPaolo PastorinoDavid Vásquez-VelásquezPaolo PeregoDavid VernezPaolo PigattoDavid Viejo-RomeroPaolo RavioloDavid WaynforthPaolo Riccardo BrustioDavid WelchPaolo RomaDavid WissPaolo RossoDavid WrightPaolo TondiDavide BarbieriPaolo TrucilloDavide BiancoPaolo VillaniDavide BizzocaPaolo VillariDavide BorroniPaolo ZatelliDavide BrottoPapanin PutsathitDavide CalandraParameswaran G. SreekumarDavide CavagnettoParamjit TappiaDavide FerorelliPargin BangotraDavide FirinuParham AzimiDavide GentiliniParichoy Pal ChoudhuryDavide GoriParinbhai ShahDavide LongatoParis BinosDavide MarianiParisa GazeraniDavide MarinoParise AdadiDavide OrsiniParisi AttilioDavide PataParita RatnaniDavide PisaniParmenion P. TsitsopoulosDavide ScozziParomita PainDavide Settembre BlundoParthasarathi SubramanianDavide SistiParul VarmaDavina Camargo Madeira SimoesParvez KhanDavinia Heras-SevillaPascal BessongDavinia VicentePascal GuenelDawid KoźleniaPascal IzzicupoDawid MadejPascale GauthierDawid PołapPascale MarangéDawid SigorskiPascual Pérez BallestaDawid SzpakPasquale AvinoDawit Teshome GebregeorgisePasquale Marcello FalconeDawn Branley-BellPasquale RussoDawn HollidayPasqualina BuonoDawood DesaiPasqualino Maietta LatessaDaya Ram ParajuliPasquino VittorioDe Gaulle I. ChigbuPasuk M. MahakkanukrauhDean G. SmithPat ThaneDean J. MillerPatil ShankargoudaDean NachmanPatricia A. BroderickDebadayita RahaPatrícia A. MadureiraDebaleena MajumdarPatrícia A. PereiraDebasis BandyopadhyayPatricia Acosta-VargasDebasree Das GuptaPatricia AnafiDebayan DeyPatricia BrownellDebbi StanistreetPatricia CaseyDebbie FaulknerPatricia Concheiro-MoscosoDebbie HagerPatricia De FranciscoDebebe ShawenoPatricia Della BarbaDebolina BiswasPatricia Esteban-FiguerolaDebora AnelliPatricia FindleyDébora Martins Dos SantosPatricia FuentesDebora PorriPatricia GarnicaDebora R. BaldwinPatricia GillenDebora RosaPatricia Grace-FarfagliaDebora Souza AlvimPatrícia Isabel MarquesDeborah A. ClaytonPatricia J. GarcíaDeborah AskewPatricia J. LardoneDeborah BowenPatricia K. CoyleDeborah GrayPatricia KhashayarDeborah IresonPatricia Kitsao-WekuloDeborah J. BowenPatricia MadureiraDeborah PanepintoPatricia MelgarDebra HainPatricia Nicole AlbersDebra JacksonPatricia Núñez GómezDebra MacKenziePatrícia Oliveira FiuzaDebra RickwoodPatricia P. WadowskiDebra StroineyPatrícia Raquel Silva FernandesDębska MałgorzataPatricia Recio SaboyaDecai JinPatricia SampedroDechristian BarbieriPatricia Sampedro-PiqueroDeena KempPatrícia SanchoDeepa AlexPatricia SchofieldDeepak KotiyaPatricia Segura MedinaDeepchandra SrivastavaPatrícia Silva LúcioDeepika BhattuPatrícia SoaresDefne Sunguroglu HenselPatricia Takako EndoDeilis-Ivonne Pacheco-SanzPatricia Torrijos FinciasDeirdre QuinnPatricia VermeerschDejan NikolicPatricia Vu-EickmannDe-Kuang HwangPatricio MoleroDeldicque LouisePatricio SánchezDelfín Ortega-SánchezPatricio Solis-UrraDelfina Gabriela Garrido RamosPatrick A. PalmieriDelia HendriePatrick Aboagye-SarfoDelia VîrgăPatrick BradshawDelky Meza-ValderramaPatrick DunnDelvon T. MattinglyPatrick J. SheehanDemetria McNealPatrick LazarevicDemetrio LozanoPatrick LegembreDemetrio MilardiPatrick M. JenlinkDemetrios ArvanitisPatrick MikalefDemetrios CantzosPatrick PageatDemetris AvraamPatrick PangDena FamPatrick Paulus Theodoor JeurissenDenice HigginsPatrick SeinigerDenis BourgeoisPatrick TrainorDenis CoelhoPatrick TrotzkeDenis MonginPatrick VanscheeuwijckDenis RoyPatrik AnderssonDenis SerenoPatrik DridDenisa VojtassakovaPatrik SöderbergDenise CloutierPatrik WennbergDenise CorridorePatrizia FarruggiaDenise DillonPatrizia GiannoniDenise GobertPatrizia GuidiDenise HaslwanterPatrizia MessiDenise M. ClaibornePatrizia TodiscoDenise MittenPatrizio PetroneDenise PumainPatrizio TratziDenise Shuk Ting CheungPatrycja LipińskaDenisse ParraPatrycja Misztal-OkońskaDennis AdjepongPatrycja Rogula-KopiecDennis EurichPatrycja Sosnowska-SienkiewiczDennis GörlichPatryk RzońcaDennis KeatingPatryk TarkaDennis L. SemanPatsie FrawleyDennis MurphyPattanee WinichagoonDennis RosenbergPattraporn SatitsuksanoaDennis ThomasPaul A. SalamhDennis V. CokkinosPaul AndellDeodato AssanelliPaul AylinDeQuincy A. LezinePaul BatchelorD’Ercole SimonettaPaul BrannaganDerek ChristiePaul BywatersDerek G. ShendellPaul ClarksonDerek MonroePaul ComfortDerek P. EvansPaul DelfabbroDerek PhebyPaul DourgnonDerrick R. StowellPaul E. EngelhardtDer-Sheng HanPaul EnthovenDer-Shiuan LeePaul Eric DossouDervla KellyPaul F. EganDesirée Mena-TudelaPaul FlowersDesireé Ruiz-ArandaPaul FuehrerDesirée Valera-GranPaul GallagherDespoina-Simoni AlexiouPaul GorczynskiDev RoychowdhuryPaul GrimshawDevajyoti DekaPaul HérouxDevarshi ShahPaul HibbardDevasena PonnalaguPaul J. AllisonDeven PatelPaul JagalsDevi MohanPaul Juinn Bing TanDevon McAslanPaul KadetzDewey TaylorPaul KindDeyan LinPaul LinksDezsi StefanPaul MilhamDhabia Ahmed BuainainPaul MuhleDhananjay MankarPaul N. WhiteheadDhananjay YadavPaul NormanDhanush HaspulaPaul RiskaDhiraj Kumar ChaudharyPaul RobinsonDi JinPaul RoseDi RenPaul S. AmieuxDiaa AhmedienPaul S. JansonsDiana ArabiatPaul SchulteDiana CastillaPaul SilviaDiana ContrerasPaul StrongeDiana Diaz RizzoloPaul TregouetDiana Díaz-GarcíaPaul W. C. WongDiana DoumasPaul WessonDiana GagliardiPaul Wilhelm DierkesDiana GariboPaul WillnerDiana Grigsby-ToussaintPaul WyllemanDiana HeimesPaul Zavala-RiveraDiana Jiménez-RodríguezPaula A. OliveiraDiana KwokPaula AlbuquerqueDiana L. SpeelmanPaula BeneveneDiana LungeanuPaula ChavesDiana M. DiNittoPaula Costa-UrrutiaDiana Maria VranceanuPaula Cristina Marques MartinsDiana Marin SuelvesPaula Esteban-GarcíaDiana MartinsPaula Felippe MartinezDiana MindrilaPaula Fernández GarcíaDiana N. CarvajalPaula Fernández-PiresDiana NicaPaula GomezDiana Rodríguez-HurtadoPaula Herrero-DizDiana SoeiroPaula K. BaldwinDiana ValeroPaula López-MartínezDiana-Maria CismaruPaula Lopez-OteroDianchen ZhuPaula LorgellyDiane AddiePaula Marie HelmDiane ClarkPaula MiottoDiane DepkenPaula NuñezDiane Lynn SmithPaula Peral GómezDiane MagranePaula PypłaczDiane RugePaula Rodríguez-FernándezDiane RyanPaula Rodriguez-ModroñoDiane Van StadenPaula SilvaDiane-Gabrielle TremblayPaula Skye TallmanDian-Jeng LiPaula Vaz-FernandesDianne WaltersPaula VeigaDibyendu DuttaPaula YagüeDickens Borame LeePaulette DorneyDidier LeiboviciPaulina BednarczykDiego BoerchiPaulina CorralDiego CrociPaulina Correa-BurrowsDiego GalvanPaulina Kostrzewa-DemczukDiego GiannonePaulina KrasnodębskaDiego Giulliano Destro ChristofaroPaulina LewińskaDiego Gómez-BayaPaulina MajekDiego Guimarães Florencio PujoniPaulina PobleteDiego Luis GonzalezPaulina PolakDiego ManavellaPaulina PolkoDiego Montiel RojasPaulina S. SockolowDiego RaimondoPaulina TrebskaDiego RomeroPaulina WlasiukDiego Sánchez-GonzálezPaulina Wojtyła-BucioraDiego Sesma-MartínPaulina WongDiego Tlapa-MendozaPauline GulliverDiego VasconcellosPauline TurnbullDieter SchrammPauline Van Den BergDietmar KrautwurstPaulo A. GameiroDietrich DarrPaulo Alexandre S. Armada Da SilvaDietrich KammerPaulo Augusto Ribeiro NevesDietrich PlaßPaulo BandeiraDih-Ling LuhPaulo BrancoDiletta GazzaroliPaulo CaldasDilip R. PatelPaulo Cesar CastroDillip Kumar DasPaulo De Mattos NetoDillon ChrimesPaulo DiogoDimitra KaraliPaulo E. S. MunekataDimitra LazaridouPaulo GentilDimitra Rafailia BakaloudiPaulo Henrique De Araújo GuerraDimitra TsirigotiPaulo Henrique GuerraDimitri PoddighePaulo Hilário Nascimento SaldivaDimitri RenmansPaulo InfanteDimitrios A. VrachatisPaulo J. PalmaDimitrios GiomelakisPaulo L. HenriquesDimitrios I. KoilakosPaulo LopezDimitrios KalfasPaulo MafraDimitrios KlaoudatosPaulo MascarenhasDimitrios KonstantinidesPaulo NogueiraDimitrios LamnisosPaulo PalmaDimitrios NikolaouPaulo PereiraDimitrios NikolopoulosPaulo SantiagoDimitrios PanagopoulosPaulo SantosDimitrios PapadimitriouPaulo ValeDimitrios PaparasPaulo Veloso GomesDimitrios PatouliasPaulo Vicente JoãoDimitrios PoulimeneasPavan MadanDimitrios RallakisPavani RangachariDimitrios RaptisPavel BachmannDimitrios StamovlasisPavel HasalDimitrios TsilingirisPavel KrystynikDimitris EmmanouilPavel ŽufanDimitris KaskaoutisPavla VachováDimitris StratouliasPavle BanovićDimitris VafidisPavlo LykhovydDimitris VlachopoulosPavlos TyrologouDimitris ZavrasPavol DancakDimity CrispPaweł BłoniarzDimosthenis ChochlakisPaweł BorkowskiDimosthenis KotsopoulosPaweł BrudekDimy FluyauPawel ChmielinskiDina Di GiacomoPaweł ChruścielDina HuangPaweł DębskiDina IzenstarkPawel DobrzanskiDina M. JonesPaweł GacDina PereiraPawel GawlowskiDina PetrashovaPaweł GlibowskiDinesh DeochandPaweł KalinowskiDinesh JillellaPaweł KlekaDinesh Kumar VermaPaweł KobusDing MaPaweł KonieczkaDing-Han WangPaweł KrukowDing-Quan NgPawel KwiatkowskiDinkantha KumararatnePaweł ŁawniczakDiogo Luís MarquesPaweł LipińskiDiogo MoraisPaweł LonkwicDiogo TeixeiraPawel MacekDiogo Thimoteo Da CunhaPaweł NetzelDiogo VidalPaweł PakoszDionisios GasparatosPaweł PiepioraDionisis PatirisPaweł PietkiewiczDionysia PanagouliaPaweł PiotrowskiDionysios TafiadisPawel PodsiadloDiosey Ramon Lugo-MorinPaweł PrędkiewiczDirga Kumar LamichhanePawel SiarkaDirk AerenhoutsPaweł SiudemDirk BrulandPawel StefanoffDirk DannenbergerPaweł SzymańkiDirk H. R. SpennemannPaweł WańkowiczDirk LachenmeierPawel WegrzynDirk MontagPaweł WięchDirk MüllerPaweł WierzbaDirk SchreckenbergPawel WolniewiczDirk TischlerPaweł WośDirk W. LachenmeierPaweł ZagrodzkiDivjyot KaurPayal GangulyDivya BhatiaPayam SadeghiDivya SahuPayam Safavi‐NaeiniDiya DouPayam ZarrintajDjuro KosanovicPearl KimDmitrii ShekPearl Pei LiuDmitriy SmolenskyPeder HjorthDmitry GrigoryevPedram P. SendiDmitry KovalevskyPedram RoghanchiDmitry RubanPedro A. Díaz-FúnezDmitry SkvortsovPedro A. Martin CervantesDmitry TretiakowPedro A. Moreno-SanchezDmitry ValeevPedro AciénDmitry VlasovPedro AlbarránDo Yup LeePedro AraujoDobromira ShopovaPedro BarataDochshanov M. AldenPedro C. Santana-MancillaDoerte WichmannPedro CandeiasDo-Gil LeePedro CantistaDolly BaliunasPedro Carreiro-MartinsDolores CorellaPedro CisternaDolores D. GuestPedro CruzDolores Galindo-RiañoPedro De BruyckereDolores Gallardo VázquezPedro Díaz-SimalDolores Ortiz-MasiáPedro DinisDolores Rando-CuetoPedro Femia-MarzoDolores VillalobosPedro Fernandes AnunciaçãoDolors CañabatePedro FerreiraDolors JuvinyàPedro FonsecaDomenic MarbaniangPedro FonteDomenico AielloPedro ForteDomenico DalessandriPedro Franca GoisDomenico FrattiniPedro Gil-MadronaDomenico LioPedro Gonzalez-MenendezDomenico MarinoPedro Guilherme Rocha Dos ReisDomenico SarnoPedro GullónDomingo J. Ramos-CampoPedro Hidalgo LopezosaDomingo RamosPedro Jesús Ruiz-MonteroDomingo Ribeiro-SorianoPedro José CorreiaDominic L. PalazzoloPedro José Tauler RieraDominic RoyéPedro Juan Tárraga-LópezDominik DłuskiPedro L. Pancorbo HidalgoDominik KryziaPedro LucasDominik ŁagowskiPedro M. HernandezDominik OlejniczakPedro M. S. M. RodriguesDominik RadzkiPedro MachadoDominik RothPedro Manuel Moreno-MarcosDominik SaulPedro Manuel Rodríguez-MuñozDominik StrzałkaPedro MartinhoDominik WawrzutaPedro MeloDominika CichońskaPedro MendesDominika DąbrowskaPedro Miguel Alves Ribeiro CorreiaDominika GlabskaPedro Miguel SardoDominika GłąbskaPedro Moruno-MirallesDominika GuzekPedro MunueraDominika MalińskaPedro NevesDominika MazurkiewiczPedro O. RosselDominika OchnikPedro Ostoa-SalomaDominika SkolmowskaPedro PimentelDominika WilczyńskaPedro Rangel HenriquesDominika ZającPedro RosárioDominique D. GagnonPedro Sánchez SelleroDominique De AndradePedro Sánchez-SotoDominique PopescuPedro Saramago GoncalvesDominique VannestePedro TadeuDomiziano TarantinoPedro TartajDon Hee LeePedro Ventura PuertosDonald B. GiddonPedro Vieira Da Silva MagalhaesDonald C. VoaklanderPedzisai MakoniDonald H. ShaffnerPeer Van Der HelmDonald John MacAllisterPeeyush RanjanDonald KenkelPeg SpitzerDonald LucasPeggy KayDonald MossPeggy Pik Kei ChowDonald N. RobersonPeida ZhanDonald R. LanninPei-Fung WuDonaldson ConservePeiheng GanDonatella CialdeaPei-Hsuan TsaiDonatella D’AntoniPei-Ing WuDonatella Di CorradoPeik GustafssonDonatella LippiPei-Lun (Perry) KuoDonato D’AgostinoPei-Shan HoDonato MoreaPei-Shu HoDonatus NohrPek Kei ImDong Hyun KimPeng WuDong Mug KangPengfei ZhangDong WangPenghao WangDong Woo KangPengpeng XuDong-Churl SuhPenney UptonDong-Hee RyuPenny AbbottDong-Her ShihPenny L. BurnsDonghwan GuPenny TrumanDonghwan LeePer Bendix JeppesenDonghwan ParkPer Medbøe ThorsbyDonghyuk ShinPer SöderstenDonghyun KimPeral Orts RamonDongkuk LimPer-Anders FranssonDongkyu KimPere Lavega-BurguésDong-Kyu KimPérez-Morcillo AitorDong-Min KwakPeriyasamy SivalingamDongni YePerla Jannet Jurado-GarcíaDong-Woo KangPerman GochyyevDong-Yeop KimPerminder GulaniDong-Zong HungPerng-Jy TsaiDonley StudlarPernilla OuisDonna WilsonPernille Tanggaard AndersenDonny GerkePerpetua ModjadjiDora Dodig HundrićPerran RossDora PereiraPerri DruenDora SalesPeršurić Anita Silvana IlakDoreen SchmidlPertti PasanenDorian SargentPessia Friedman RubinDorian SkrobekPetal Petersen WilliamsDorin GheorghePetar JevticDorin TibulcaPetar MitićDoris KnoblauchPete DriezenDoris LydahlPeter A. KeyelDoris RubioPeter AntesDoris RusicPeter BaasDoris Y. P. LeungPeter BaxterDorit NitzanPeter BicanDorit Segal-EngelchinPeter Bob PeerenboomDorit Zimand SheinerPeter BottenbergDorota Bielinska-WazPeter ČekanDorota CzyżowskaPeter Edward McKennaDorota DomalewskaPeter EdwardsDorota GawrylukPeter EibichDorota GroffikPeter F. MeiksinsDorota Iwaszkiewicz-GrzesPeter FineDorota KaletaPeter FranklinDorota KleszczewskaPeter GänglerDorota Klimecka-TatarPeter GlynnDorota KmiecPeter GrayDorota Kostrzewa-NowakPeter HofmannDorota KrupaPeter HricDorota LasotaPéter HudomietDorota LewczukPeter IngwersenDorota Martysiak -ŻurowskaPeter J. AspinallDorota OrtenburgerPeter JarcuskaDorota OzgaPeter Josef StauvermannDorota PorowskaPeter KaatschDorota RaczkiewiczPeter Kenneth HuggardDorota TchórzewskaPeter KerrDorota WójcikPeter KevernDorota WójtowiczPeter KochDorota WrześniokPeter KondrlaDorota ZyśkoPeter KoscakDorothea KesztyüsPeter LarsonDorothea KoppischPeter LemonDorothy DaleyPeter MapleDorothy PathakPeter MawsonDorothy TaylorPeter MilgromDörte SollePéter OláhDorte Toudal ViftrupPeter OttDorthe Varning PoulsenPeter PikoDoru DiculescuPeter ProdingerDoug VogelPeter RumneyDougho ParkPeter SchemmerDouglas BoydPeter SchmidtDouglas DaviesPeter ScholliersDouglas HanesPeter ShanklesDouglas LandsittelPeter SmithDouglas MacDonaldPeter StehleDouglas MyersPeter TörökDouglas O’ShaughnessyPeter UkpokoduDouglas WalkinshawPeter Van De VenDovile VildaPeter VidmarDoyeon WonPeter W. ChoateDra Garcia-CaroPeter W. GrandjeanDragan MirkovPeter W. SchofieldDragana GabrićPeter WakholiDragana VidovicPeter WallnerDragoș Ioan TohăneanPeter WhittakerDragos MedianPeter WilsonDragoslav NikezicPeter WinwoodDraženko GlavićPeter-Michael SackDrew HelmerPetjon BallcoDries Van GassePetko KusevDrona RasaliPetr BaďuraDuarte MarquesPetr BahenskýDuarte Miguel Henriques NetoPetr O. IlyinskiiDubravka Havaš AuguštinPetr StastnyDubravka SladićPetr SzturzDubravka Švob ŠtracPetr VajdaDuc-Hiep NguyenPetr WeissDuguleană LilianaPetra CzarniakDu-Hyeong LeePetra HorváthováDukmin KimPetra JílkováDulce FilgueiraPetra KolicDulce Maria Cairós BarretoPetra KoraćDulce MinayaPetra SurlinDumitru Constantin-TeodosiuPetra ThürmannDuncan HillPetri BockermanDuncan MerciecaPetri Rikhard BöckermanDunja DegmečićPetrona LeeDuo Wai-Chi WongPetronila OlivaDuo ZhangPetros MourouzisDurbar RayPetros MouzouridesDurdane Bayram-JacobsPetrus GantoisDušan DimićPetter RanefallDušan HrabecPetya VentsislavovaDušanka KrajnovićPhaneendra K. DuddempudiDwueng-Chwuan JhwuengPhd. José Luis Gálvez NietoDylan B. JacksonPhil AyersDylan CawthornePhil ChilibeckDylan FlawsPhil J. HandcockDylan Jack RichardsPhil RauschnabelDylan LeiPhilip AnyanwuDylan M. JohnsonPhilip ChadwickDylan SeltermanPhilip HodgsonDzintra IliškoPhilip J. CooperE. A. M. Van PuffelenPhilip LewisE. D. WilliamsonPhilip LiE. David G. McIntoshPhilip M. GallagherE. IrzmańskaPhilip R. StanforthE. Jean BucklerPhilip SamE. M. EkanayakePhilip TsengE. Mark WilliamsPhilip W. S. NewallE. N. BogdanovaPhilipp A. IlinykhE. Scott SillsPhilipp BadorrekEarl DuncanPhilipp BergerEarthea NancePhilipp DoeblerEbba SundinPhilipp KanzowEbenezer TumbanPhilipp LenzEbrahim GhaderpourPhilipp ÖhlmannEbru Ersoy TonyaloğluPhilipp Paul LobmaierEckart HanekePhilipp SischkaEckhard FrickPhilipp TreinEd W. M. T. WesterhoutPhilippa HüpenEdel EnnisPhilippe BrouquiEdeltraut KrögerPhilippe GuillaumeEdgar GutiérrezPhilippe Herman-BausiervEdgar TovillaPhilippe IrigarayEdgar Vazquez-NunezPhilippe Laval-GillyEdgaras LinkeviciusPhilippe Matthias TschollEdgaras LinkevičiusPhilippos KaripidisEdgardo SicaPhiliswa Nosizo NomngongoEdgars EdelmersPhillip OzimekEdi DefrancescoPhimon AtsawasuwanEdimansyah AbdinPhoebe GriffinEdinson Dante Meregildo RodriguezPhu DuongEdite Teixeira-LemosPhyllis AnastasioEdith ClarosPhyllis Ann Solari-TwadellEdith Cruzado TafurPhyllis GasparEdith LahnerPia Lopez-JornetEdith ReuschelPier Franco LattanziEdmarie Guzmán-VélezPier MannucciEdmund Man King LuiPier SaccoEdmund TomaszewskiPieranna ChiarellaEdmundo Bonilla GonzálezPierfrancesco FrancoEdmundo Rosales-MayorPiergiorgio DanelliEdmunds ReineksPiergiuseppe AgostoniEdna AcostaPiergiusto VitulliEdna Dias CanedoPierluigi CarbonaraEdna PatatanianPierluigi CongedoEdna PossanPierluigi CordellieriEdnardo RochaPierluigi PolitiEdoardo BraunerPiero LovreglioEdoardo FaddaPiero MaestrelliEdoardo RellaPiero MorselettoEdoardo StaderiniPiero PapiEdra LondonPiero PavoneEdsaul Emilio Pérez-GuerreroPierpaolo FerranteEdson KieuPierre GanderEduard A. KashubaPierre RoubertouxEduard BezuglovPierre-Yves DeclercqEduard Cristobal FransiPierre-Yves RobillardEduard Cristobal-FransPieter Hermanus MyburghEduard EdelhauserPieter Jj SauerEduard Gabriel CeptureanuPieter Van DamEduard InglésPietro CacialliEduard Moreno-GabrielPietro CombaEduarda Marques Da CostaPietro FerraraEduardo A. F. NilsonPietro MontemezziEduardo A. Lopez-MaldonadoPietro MorroneEduardo A. MoralesPietro MuratoriEduardo AlgrantiPietro PicernoEduardo BernabePietro PulinaEduardo BoriePietro QuaglinoEduardo DovalPietro RubegniEduardo Fernández-OzcortaPietro SalvagoEduardo GarridoPil Young JungEduardo Gutiérrez-AbejónPilar Alfageme-GarcíaEduardo Hernández-PadillaPilar Aparicio-ChuecaEduardo Jiménez-FernándezPilar Aparicio-MartinezEduardo OsunaPilar Blanco-FernandezEduardo Padilla CamberosPilar Blanco-LoboEduardo Sánchez-SánchezPilar GiraldoEduardo SanteroPilar LacasaEduardo SantosPilar Lusilla-PalaciosEduardo SiqueiraPilar Marqués-SánchezEduardo TeixeiraPilar Martin HernandezEduardo ToméPilar MatudEdurne Elgorriaga-AstondoaPilar Nieto-GilEdurne Garcia IriartePilar Pérez-RosEdvard JohanssonPilar Sainz-de BarandaEdvardas DanilaPilar ZafrillaEdwar Forero-OrtizPin HaEdward A. LawsPinakin DaveyEdward AsisPinar AvciEdward F. FitzgeraldPinar Emecen-HujaEdward FitzgeraldPin-Chao LiaoEdward FreemanPing C. MamiyaEdward HigsonPing Jen ChenEdward J. WangPing SongEdward KoifmanPing WangEdward KrocPing ZouEdward L. BoonePing-Chung LeungEdward LebakaPinghe NiEdward LoPing-Hung HsiehEdward P. HofferPing-Ting LinEdward RileyPiotr ArtiemjewEdward TowerPiotr BalazyEdward WallerPiotr BułaEdward ZimbudziPiotr ChmielarzEdwin BrandsPiotr ChomczyńskiEdwin PalacioPiotr DługoszEdwin TanPiotr DuchnowskiEdyta CharzyńskaPiotr F. BorowskiEdyta KardasPiotr FormanowiczEdyta KinelPiotr GodlewskiEdyta ŁaskawiecPiotr GołębiowskiEdyta ŁuszczkiPiotr GradziukEdyta MądryPiotr GronekEdyta NartowskaPiotr Henryk SkarzynskiEdyta PasickaPiotr HolnickiEdyta PlebankiewiczPiotr JarzynkowskiEdyta Roslon-SzerynskaPiotr KaczkaEdyta Sidorczuk-PietraszkoPiotr KapustaEdyta SpychałPiotr KarniejEeva HarjuPiotr KornetaEfrat NeterPiotr KowalskiEfstathios PettasPiotr LeszczyńskiEfstratios NikolaivitsPiotr LisEfstratios Stratos NikolaivitsPiotr LityńskiEftihia NathanailPiotr LorensEgidio BarbiPiotr MatłoszEhsan AbedinPiotr MazurkiewiczEhsan AsadollahiPiotr MerksEhsan KhanPiotr MiśkiewiczEhsan MohammadiPiotr ModzelewskiEhsan SharifiPiotr MorasiewiczEhsanul Hoque ApuPiotr Oskar CzechowskiEiichi SatoPiotr ParzychEiichi WatanabePiotr PetrykowskiEiji ArakawaPiotr PoznańskiEila KankaanpääPiotr ProchorEinar B. ThorsteinssonPiotr Robert SzkodziakEinar ThorsteinssonPiotr RybarczykEirini BathrellouPiotr SulewskiEirini OrovouPiotr SzajerskiEirini PanagopoulouPiotr SzwedaEitaro KodaniPiotr SzymaniecEithne SextonPiotr T. NowakowskiEivind AadlandPiotr TomskiEjemai EboreimePiotr TutkaEjidike IkechukwuPiotr WaleckiEkaterini GeorgiadouPiril HepsomaliEkhard E. ZieglerPirjo-Liisa RantanenEl Khalil CherifPittaluga PaolaEladio Collado-BoiraPiyapong JanmaimoolElaine BidmeadPiyawat KatewongsaElaine C. RushPiyush ShuklaElaine M. HaasePlácido A. Souza NetoElaine McCarthyPlácido Rogerio PinheiroElaine SouzaPlaton PatlakasElanor Lucy WebbPlaton TiniosElba MaurizPo Sen HuangElbert AlmazanPoay Sian Sabrina LeeEleanor DommettPo-Chang HsuEleanor KanePo-Chih ChiuEleanor L. S. LeavensPo-Ching WangElena A. GrigorievaPoh Heng ChongElena Alexandrovna VasilievaPo-Jen HsiaoElena AtienzaPoju ChangElena BotezatPo-Lin PanElena BunduchiPollie Bith-MelanderElena CancianiPolona SeličElena ChianesePooja S. JagadishElena Chover-SierraPoonam AgarwalElena CiguPoppy WatsonElena CommodariPosen LeeElena Constanta AmăricăiPoudel ShobhaElena CyrusPoyan BarabariElena D. BazhanovaPoyu ChenElena DoratoPrabhuraj D. VenkatramanElena DruicaPradeep PodilaElena Escolano-PérezPradhib VenkatesanElena Fernández-DíazPragya Sharma GhimireElena Fernández‐GarcíaPrahlad HoElena García-GarcíaPrajakta AdsulElena GladunPrakash BhuyarElena GrimacciaPramod KhoslaElena GubarPranita ShresthaElena HadjimbeiPrasad NalabothuElena Jiménez-PérezPrasad SrikakulapuElena LarochePrasanna NeelakantanElena MacchioniPrasanth PuthanveetilElena Mainer PardosPrasenjit SahaElena María Pérez GonzálezPrashant AnandElena Martínez-SanzPrashant RajputElena MoltchanovaPrashant SharmaElena Ortega-CamposPrateek M. ShresthaElena ParianiPraveen KolarElena PiacenzaPraveen KumarElena PiñeroPraveen Kumar PotukuchiElena PiraniPravin P. UpareElena Quintana-MurciPreethy S. SamuelElena RoccaPreeti ChandraElena Rodica IonescuPremachandra WattageElena SavoiaPrenilla NaiduElena SilvestriPrerna VarmaElena Sinkiewicz-DarolPriit ZingelElena StănculescuPrince DonkorElena StefanaPriscila GiavedoniElena TarcaPriscilla G. WittkopfElena TenconiPritam BoseElena TovbisPriti PednekarElena V. TchetinaPriya Darshni ShanmugarajahElena-Daniela GrigorescuPriya HalvorsenEleni KalantidouPriya J. WickramaratneEleni KortianouPriya WickramaratneEleni NikolaouPriyanka AgrawalEleni PetkariPriyanka KhareEleni VasileiouPriyanka MishraEleni VergadiPriyanka RayEleni VousouraPriyanka SandalEleonora Bielawska-BatorowiczProf. Leonie ShortEleonora GrespanProttoy HasanEleonora LauriniPrudence AtukundaEleonora PiccoPrzemyslaw CwynarEleonora RosiPrzemysław CzerniejewskiEleonora TopinoPrzemyslaw Falkowski-GilskiElettra MancusoPrzemysław GarsztkaEleuterio Sanchez RomeroPrzemysław GrodzickiElham HedayatiPrzemyslaw J. KotylaEli RistevskiPrzemysław KlapaElia Fernández-MartínezPrzemysław KołodziejElia GruesoPrzemysław Mateusz DomagałaElia Mora RíosPrzemysław NiedzielskiEliana De LucaPrzemysław PetryszakEliana PintusPrzemysław PietraszewskiEliane Aparecida CastroPrzemysław ŻuratyńskiEliane Teixeira MársicoPu DengElias DritsasPu HuangElias M. KlempererPui Fong KanElias MpofuPuiu NistoreanuElias NazarenoPura Marín SanleandroElif Inan-ErogluPurnahamsi DesaiElikanah Olusayo AdegokePushpa Raj JoshiElin BørøsundPuspa Raj PantElin EkbladhPuthearath ChanElin LundquistQammer ZaibElio BignardiQasim AlhadidiElio BorgonoviQian DiElio CastagnolaQian LiuElio VenturiniQian QinzhenElisa AmbrosiQiang YangElisa Baraibar-DiezQiangjun CaiElisa BelluzziQichang MeiElisa BenedettiQihui ChenElisa Bizetti PelaiQimin HaiElisa BustaffaQin Xiang NgElisa CairrãoQing LiElisa DelvecchioQinghua YangElisa GambettiQingyang WeiElisa I. Sánchez-RomeroQingyu ZhouElisa KallioniemiQingzhe JiaElisa LangianoQinyi LiElisa Lopez-DoladoQiyang LiuElisa PinedaQoot AlkhubaiziElisa RobertiQu ChengElisa SchaefferQuan-Hoang VuongElisa ZavattaroQuansan YangElisabet A. ForsumQuazi RahmanElisabet Fernández-GómezQuentin Durand-MoreauElisabet Roca-MillanQuin MorrowElisabeta BotezR. Christopher DoironElisabete AlvesR. Constance WienerElisabete BorgesR. Henry OlaisenElisabete P. SilvaR. Steven PappasElisabeth A. StelsonRabia HussainElisabeth ArandaRabiul IslamElisabeth FernellRachael FrostElisabeth LippertRachael PotterElisabeth PinartRachael TaylorElisabeth QuendlerRachel A. FuscoElisabeth RiegelRachel BoykanElisabeth Serrat-SellabonaRachel ChildersElisabeth Von EssenRachel DaleElisabeth Wilson-EveredRachel FlynnElisabetta BertelliniRachel J. SkowElisabetta BertolRachel JenkinsElisabetta CecconiRachel JohnsonElisabetta D’AgattaRachel LoganElisabetta De VitoRachel Mamlok-NaamanElisabetta Filomena BuonaguroRachel NesbitElisabetta LombardiRachel Rosenberg GoldsteinElisabetta M. VencoRachel SharabyElisabetta MarconiRachel SumnerElisabetta NardellaRachel TaylorElisabetta PolizziRachel ThamElisabetta SagoneRachel TooveyElisabetta SalvioniRachel WestElisabetta VersinoRachel YerburyElisaldo Mendes CordeiroRachele MacirellaElisângela Brito Pessoa VilarRachele MarianiElisângela De Souza LoureiroRachid OuaretElisardo BecoñaRachid OulahalElise Arsenault KnudsenRachid SalmiElise F. TalsmaRada K. DagherElise S. BrezisRadhakrishnan PanatalaEliseo BustamanteRadhika BordeEliseo Iglesias SolerRadhouene DogguiEliseo ValleRadim VáchaElisete DiogoRadmila JankovićEliška SovováRadoslaw B. WalczakElismauro Francisco MendonçaRadosław GacaElissa LaddRadosław GocołEliya LevingerRadosław KazimierskiEliza KalbarczykRadosław WolniakEliza TsouRadovan SlezákElizabeta B. Mukaetova-LadinskaRadovan ŠomplákElizabeth A. CowlesRadu BotgrosElizabeth A. KayeRadu C. RacovitaElizabeth A. ParkerRadzisław MierzyńskiElizabeth AndradeRaf DewilElizabeth Armstrong-MensahRafael A. CasusoElizabeth B. KlermanRafael Almendra-PeguerosElizabeth BaldwinRafael Alves GuimarãesElizabeth CañasRafael Arcángel Caparrós-GonzálezElizabeth CayananRafael BernardesElizabeth D. NobmannRafael BurgueñoElizabeth Emilie RostedRafael Casuso-PérezElizabeth EnglanderRafael Del Rio-SalasElizabeth G. BradleyRafael Escamilla-NunezElizabeth GollubRafael García-RosElizabeth H. BradleyRafael Gómez-GalánElizabeth HardingRafael J. BuralliElizabeth HatchRafael Martínez-GallegoElizabeth HicksRafael MendesElizabeth HillRafael Miranda FerreiroElizabeth HortonRafael Molina LuqueElizabeth HunterRafael Moreno-Gómez-ToledanoElizabeth J. HalsteadRafael ObregonElizabeth J. KingRafael OliveiraElizabeth K. CahoonRafael Ravina-RipollElizabeth KeidaRafael Timóteo De Sousa JúniorElizabeth KukielkaRafael Urrialde De AndrésElizabeth L. SeamanRafael Velázquez-CruzElizabeth NewnhamRafael Vila-CandelElizabeth OcholaRafael ZárateElizabeth OnyangoRafaela VasiliadouElizabeth PierothRafaella Pessoa MoreiraElizabeth RixRafał BalinaElizabeth S. EvansRafał BlazyElizabeth S. FernandesRafał BułaElizabeth Selene Gómez AcataRafał ChmaraElizabeth SokolRafal DankowskiElizabeth Solis-PérezRafał DługoszElizabeth SwedoRafał GerymskiElizabeth TarlovRafał J. DoniecElizabeth ThomasRafał JanusElizabeth UnniRafał LeśniczakElizabeth V. NewbronnerRafał ŁopuckiElizabeth VafiadakiRafal MierzwiakElizabeth Ya-Hui ChangRafal OgorekElke HertigRafał SkowronekElke HumerRafał StemplewskiElke KniselRafał SzafraniecElke NaumannRafał WójciakElkhan Richard Sadik-ZadaRafat ZrieqElkin LuisRafel Cirer-SastreElla Cohn-SchwartzRaffaele CampisiEllen CarlRaffaele La PortaEllen EngelhardtRaffaele PezzilliEllen HertzmarkRaffaele PillaEllen KemlerRaffaele ScuratiEllen Siobhan MitchellRaffaella BiagioliEllerie WeberRaffaella BrugnoniElli HeikkiläRaffaella PanzaEllie AbdiRaffaella TestoniEllie BrownRaffaella ZerbettoEllie CarmodyRaffaella ZucaroElliot BenjaminRaffaello PapadakisElliot LassonRaghavendra TirupathiElliott JonathanRaglan MaddoxEllyn R. MulcahyRagna StalsbergElnaz SafapourRaha PazokiEloisa Guerrero BaronaRahbel RahmanEloisa MacedoRahel SchomakerEloísa SevillaRahshida AtkinsEloy CondeRahul MallickElpidio Maria GarzilloRahul SehgalElsa Cristina Sacramento PereiraRahul ShekharElsa Frideborg RonningstamRai Campos SilvaElsa López-PintorRaif CergibozanElsa OrellanoRail M. ShamionovElsa PegadoRaimund GeeneElsa RibeiroRaimund WidhalmElsa T. RodriguesRaimundo Jiménez BallestaEl-Sayed E. MehanaRainer FriedrichElsebeth LyngeRainer GuskiElspeth McInnesRainer ReileElvin Thomaseo BurtonRainer Rubira-GarcíaÉlvio Rúbio GouveiaRais AnsariÉlvio Rúbio Quintal GouveiaRaja Chandra ChakinalaElvira MaranesiRaja GanesanElvira RincónRaja Singh PaulrajElvis Joacir De FrançaRaja VeerapandianElyse ClarkRajaguru VasukiElyse PassmoreRajalakshmi RamPrakashElyse WarnerRajamma MathewElysia DonovanRajan Deep AdhikariElza Maria Morais FonsecaRajan KCElzbieta Bobrowicz-CamposRajarshi DasguptaElzbieta BroniewiczRajat Emanuel SinghElżbieta CiporaRajeev DwivediElżbieta Krajewska-KułakRajeev SinglaElzbieta NowickaRajendra KhanalElzbieta PaszynskaRajendra ParajuliElżbieta PetriczkoRajib ShawElzbieta RosiakRajmohan DharmarajElżbieta RyńskaRajtarun MadangopalElżbieta Sochacka-TataraRakiya SaiduElżbieta SzymańskaRalf RisserElżbieta WierzbickaRalph HansmannElżbieta Wójcik-GrontRalph L. PiedmontElżbieta ZyskRaluca Horea-SerbanEma LeiteRam AvtarEman AnisRam BajpaiEman M. OthmanRam JagannathanEman TadrosRam SharmaEmanuel SchneckRama Prasada MohapatraEmanuela CamilliRama PulicharlaEmanuela CoppolaRama Rao KarriEmanuela GrittiRamachandran PadmavatiEmanuela Gualdi-RussoRamachandran PrakasamEmanuele BarabinoRaman GoyalEmanuele BlasiRaman VedarajanEmanuele CannizzaroRamandeep S. TakhterEmanuele CaroppoRamasamy MouliEmanuele CerrutoRamcés Falfán-ValenciaEmanuele GiorgiRamcharan Singh AngomEmanuele LeporelliRamesh KumarEmanuele Maria GiustiRamesh Kumar MuralimanoharEmanuele Maria MerloRamesh PeriasamyEmanuele PelosiRamey MooreEmanuele PerugiaRamgopal KashyapEmanuele TorriRamires TibanaEmelina López GonzálezRamiro Figueiredo CatelanEmerson SebastiãoRamjee BhandariEmet SchneidermanRamjee GhimireEmiko SenbaRamkumar SrinivasaganEmil DrápelaRamón Chacón CuberosEmilia GrzędzickaRamon FlechaEmília HroncováRamón García-PeralesEmilia I. De La Fuente-SolanaRamon LavadoEmilia IrzmanskaRamón López FacalEmília Isabel Martins Teixeira Da CostaRamon Luengo-FernandezEmilia JaneczkoRamón Montes-RodríguezEmilia OgodescuRamon Ramon-MuñozEmilia PaoneRamon Rueda LopezEmilia VassilopoulouRamón Valle CabreraEmilian ZadarkoRamona BirauEmiliano Rodríguez-SánchezRamona BongelliEmiliano SironiRamona BranEmilie CameronRamona HenterEmilie DionneRamona Işfănescu-IvanEmilie ValléeRamona NăstuțăEmilien JeannotRamūnas PovilanskasEmilien PelletierRamy Abou GhaydaÉmilien SchultzRan TianEmilija D. JensenRanadheer R. DandeEmilija WilsonRanda El BedawyEmilio Abad-SeguraRandall HolcombeEmilio AndreozziRanganath VallabhajosyulaEmilio Casariego-ValesRangaswamy D REmilio Crisol-MoyaRania W. SemaanEmilio GianicoloRanit ChatterjeeEmilio J. Delgado-AlgarraRanjeet ThakaliEmilio López-ParraRanjeev Chrysanth PulleEmilio Lozano-AguileraRanjita DhitalEmílio Prado Da FonsecaRanjita MisraEmilio SaccoRanjith KamityEmilio TrigiliRanka GodecEmily Alden HennessyRanko M. KutlešićEmily DarlingtonRaouf Senhadji-NavarroEmily EstradaRaphael AraujoEmily H. MorganRaphael HerrEmily J. HaasRaquel AlarcónEmily JankeRaquel Aparicio UgarrizaEmily JohnstonRaquel Barragán-SánchezEmily K. TarletonRaquel BoiaEmily KiesonRaquel Braz Assunção BotelhoEmily LubartRaquel Cantero-TéllezEmily RugelRaquel Costa-AlmeidaEmily S. BaileyRaquel DiezEmily ScheinfeldRaquel EscortellEmily SettyRaquel Fábrega-CuadrosEmily ShoesmithRaquel Fernández-CézarEmily SteelRaquel Ibáñez MendizábalEmily Zielinski-GutierrezRaquel L. BernardinoEmina Kristina PetrovićRaquel Lara MorenoEmma AshworthRaquel Lazaro GutierrezEmma BerryRaquel Leirós-RodríguezEmma CarlinRaquel Lozano BlascoEmma CedstrandRaquel Luengo GonzalezEmma ChiaramelloRaquel M. GuevaraEmma EspinosaRaquel MayordomoEmma H. BakerRaquel RamosEmma HockRaquel Revuelta IniestaEmma L. DavisRaquel SanchezEmma MillerRaquel Sánchez-RecioEmma MotricoRaquel Sánchez-RodríguezEmma SiesmaaRaquel Vaquero-CristobalEmma SmithRasa JankauskieneEmmanouela SdonaRasa MladenovicEmmanouil MalandrakisRasa Pilkauskaitė ValickienėEmmanouil N. KokkinakisRasa PocevičienėEmmanouil N. MagiorkinisRasa PranskūnienėEmmanouil RizosRashid AmeerEmmanuel BonnetRashid M. AnsariEmmanuel D. AdamidesRashid MehmoodEmmanuel KidandoRasmus RiviniusEmmanuel MulinRastislav StojsavljevićEmmanuel Navarro-FloresRatko RadakovicEmmanuel Obeng-GyasiRattana JariyaboonEmmanuelle AmorosRaul Alberto Carrilho CordeiroEmrah AtilganRaul Alegria-MoranEmyr BenbowRaúl Arango-MirandaEnbo MaRaúl CachoEncarnación Martínez-GarcíaRaul Cruz-CanoEncarnación Moral PajaresRaúl Cuauhtémoc Baptista RosasEncarnación Satorres PonsRaul DomínguezEnedina QuirogaRaul Felipe Palma-AlvarezEngelbert SchrammRaúl Fernández-TorresEnid J. SchatzRaul Josue Nájera-LongoriaEnio ParisRaúl Juárez-VelaEnkhjargal BatbaatarRaul Justo SanzEnnio CadumRaul Lopez-GruesoEnoch HoRaúl López-IzquierdoEnoch ParkRaúl Marticorena-SánchezEnquan XuRaúl Martos-GarcíaEnrica MarzolaRaul NavarroEnrica MiglioreRaúl Payá CastiblanqueEnrico BergamaschiRaúl Quevedo-BlascoEnrico BroccoRaul Ruiz-CeciliaEnrico BrunettiRaúl Soto-CámaraEnrico CollantoniRaúl Tárraga MínguezEnrico DestefanisRaúl VinetEnrico Fuini PugginaRauno PääkkönenEnrico M. CamporesiRavesh SinghEnrico MancinelliRavi Kumar GudipaneniEnrico MarinelliRavi Kumar KomaravoluEnrico Mario CamporesiRavinder Reddy GaddamEnrico NicosiaRavindra KumarEnrico PerriRavindranadh KoutavarapuEnrico PiccirilloRay A. YeagerEnrico ZaccheiRay CorrellEnrique ChacónRay NorburyEnrique García BengoecheaRaymond D. AdamsEnrique HernandoRaymond MoodleyEnrique Medina-AcostaRaymond PalmerEnrique Méndez BolainaRaymond RastegarEnrique Pastor SellerRaymundo Buenrostro-MariscalEnrique S. PumarRayonil CarneiroEnrique Sáez-AlvarezRazia AdamEnrique SotoRazvan SocolovEnsa JohnsonRazvan SusanEnwu LiuRebeca Monroy TorresEnza SperanzaRebeca Montes-MontesEnzo FerrariRebeca PardoEnzo IulianoRebecca BattistaEnzo SpisniRebecca BaxterEoin KingRebecca CunninghamEoin MurrayRebecca GoldsmithEoin PlantRebecca H. ChungEphraim Kwashie ThompsonRebecca HornEphrem EngidaworkRebecca J. SargissonEraldo OcchettaRebecca LindbergEran BendavidRebecca MaguireEran ElhaikRebecca Mary MeiringErdem GökerRebecca MeadErdogan DogduRebecca NowlandErgo RikmannRebecca PowellEri EguchiRebecca PrevotsEric A. EppingRebecca SargissonEric B. BrandtRebecca Susan DeweyEric BenotschRebecca TsaiEric BravermanRebekah H. NaglerEric C. FreundtRebekah R. HeltonEric CarlinRebekkah MiddletonEric DukuRedamy Perez-RamosEric E. HallReddy. P. HemachandraEric G. PostRedha TaiarEric GilRedi RahmaniEric J. R. ParteliRedmond ShamshirEric JeandidierReed MangelsEric Kam-Pui LeeRega AngeloEric KyereRegina Fölster-HolstEric MartinRegina GrazulevicieneEric NgRegina M. BuresEric PoonRegina Pana-CryanEric RischRegis BarilleEric RobitailleReham ShalabyEric SilvaRehana ShresthaEric SobolewskiRei ShimizuEric StokanReidar P. LystadErica FuhrmeisterReidun ØvstebøErica GobbiReiji KojimaErica Isa MoscaReilly R. KayserErica L. MajumderReima SuomiErica MenegattiReinald BesaluErica Y. LauReinhard StrametzErich BrennerReinier TimmanErich CosmiRemedios Lopez-LiriaErich KastenRémi CostagliolaErick Sierra-CamposRemigijus BubnysErico Fernando Pereira-SilvaRemigiusz GałęckiErico KutcharttRemigiusz KozłowskiErik AhlbergRemo MombargErik B. LarsonRenan Magalhaes MontenegroErik BehringerRenata ChałasErik BerglundRenata ČinčikaitėErik J. JonerRenata GajErik KarlssonRenata GasparinoErik NessonRenata Graciele ZanonErik StenbergRenata KarkowskaErik SuRenata KazimierczakErik UhdeRenata Marks-BielskaErika A. MontanaroRenata NestorowiczErika CioneRenata Puppin ZandonadiErika CottoneRenata RetkuteErika HernandezRenata Różycka-CzasErika IkedaRenata RutkauskaiteErika JoosRenata SiemienskaErika MoenRenata SistoErika T. EbbsRenata SolarskaErika ZelkoRenata SoliminiErika ZemkováRenata TeparićErin BouldinRenata Tobiasz-SalachErin E. SchirtzingerRenata YanbykhErin HolmesRenata ZandonadiErin SemmensRenato De-FilippisErin WillisRenato GiacominiErivan RamosRenato HeidorErja RappeRenato SommaErjia GeRenbin XiaoErlandson Ferreira SaraivaRene CapoteErmelinda De MeoRene FriedlandErminia ColucciRené J. L. MurphyErnest BaigetRene KadenErnest BladeRené Loredo PortalesErnest TyburskiRene RietraErnest UwayezuRené WesterholtErnesto A. Lagarda-LeyvaRenee CareyErnesto CaffoReneta BarnevaErnesto González-MesaRen-Fang ChaoErnesto LevaRenfeng MaErnesto LodiRen-Jay SheiErnesto MarcheggianiRenjie ChenErnesto RestrepoRenuka RamanErnesto Rodríguez-CrespoRenzhi LiuErnst Albin HansenRenzo CapitaniErpeng WangResi PucciErrol HassanReuben HowdenErshuai ZhangReza ZamaniErsilia NigroRezart HoxhajEshetu Janka WakjeraRezzy Eko CarakaEsin YorukRhona O’ConnellEsmeralda Santacruz-SalasRhonda GaradEsperanza Begoña García NavarroRhonda MattinglyEsra AkbasRianna MurrayEstanislao Gutiérrez-SánchezRianne CostelloEsteban Agulló-TomásRiasat HasanEsteban Obrero-GaitánRičardas RadišauskasEstefania NuñezRicardo AlmendraEstela M. DíazRicardo CácedaEstela Marine-RoigRicardo Cardoso CassilhasEstelio DantasRicardo Castro AlvesEstella OncinsRicardo ChalmetaEster AyllónRicardo CuevasEster D’AccardiRicardo D. SalvatoreEster GuijarroRicardo DuarteEster Teixidó CompañóRicardo EdvanEster Vidaña-VilaRicardo Francisco Reier ForradellasEstera Twardowska-StaszekRicardo GomesEsther A. BaloghRicardo Gregorio LugoEsther Ching Lan LinRicardo Hernández-RojasEsther Christiane RindRicardo IbañezEsther FitzpatrickRicardo Ibañez PérezEsther García-SánchezRicardo Iglesias-PascualEsther LázaroRicardo Iván Jeldres ValenzuelaEsther López-ZafraRicardo J. Alves De SousaEsther MaasRicardo J. FernandesEsther Martínez-GarcíaRicardo Javier Cerda RiosecoEsther Medrano-SánchezRicardo Josa-FombellidaEsther Moraleda SepúlvedaRicardo Luiz Cavalcanti De Albuquerque-JúniorEsther Sitges MaciáRicardo Moreno-RodriguezEsther SternbergRicardo Ney Oliveira CobucciEsther T. MaasRicardo Paes De Barros BertonEsther TantsisRicardo Pérez-SánchezEstrella RausellRicardo RodriguesEstrella RomeroRicardo SantosEszter DuczaRicardo SilvaEszter Mária HorváthRicardo Torres-JardónEszter SzabadosRicardo VardascaEthna ReganRicardo Ventura BaútoEti Ben SimonRicardo ZanellaEtienne MarbaixRiccardi RossanaEtienne PiguetRiccardo CorradoEto Silas FernandesRiccardo IzzoEttie M. LipnerRiccardo MazzolaEttore BidoliRiccardo MonterubbianesiEttore NapoliRiccardo PaoliniEttore VarricchioRiccardo PatriarcaEufrasio Pérez NavíoRiccardo PolosaEugen RoscaRiccardo ScalengheEugene Haydn WaltersRiccardo ScorrettiEugenia AllegraRiccardo ValdezEugénia GallardoRiccardo ZuccaEugenie DugouaRichard BellEugeniusz KodaRichard BrightEugeniusz SajewiczRichard ChristianaEui Hoon LeeRichard David HaywardEulalia SkawińskaRichard Eduardo Toro ArayaEun Hee LeeRichard FlemingEun Jee LeeRichard GunEun Jeong HwangRichard H. Parrish IIEun Ji SeoRichard HolyEun Ju LeeRichard IsralowitzEun Soo ParkRichard J. CebulaEun Young LeeRichard JenkinsonEunhee LeeRichard K. FlemingEun-Hi ChoiRichard KdolskyEun-Hi KongRichard L. WarmsEunice CarrilhoRichard L. WolfelEunjeong ChaRichard M. RyanEun-Jeong KimRichard MillsEunjoo YangRichard MolesEunyoung HanRichard MorehouseEurico Augusto Rodrigues SeabraRichard ParrishÉva ÁcsRichard PeterEva Aguaded-RamírezRichard S. FeinnEva Cifre GallegoRichard Steven PappasEva GoldfarbRichard SuminskiEva HolsingerRichard TrohmanEva Krieghoff-HenningRichard ViskupEva León ZarceñoRichard W. BohannonEva M. Medina-RodríguezRichard W. HallEva M. Santos LópezRichard W. StoffleEva María Martínez-JiménezRichard WebbEva MračkováRichard Yudi HidaEva SiegelRichardson DilworthEva-Charlotte EkströmRickelle RichardsEvan BushRida WaheedEvan CarverRie MiyazakiEvan McEwingRifat AkhterEvangelia AntoniouRiggs J. KlikaEvangelia ChrysikouRihana PeimanEvangelia KerezoudiRiitta HannonenEvangelia KouidiRiitta TurjamaaEvangelia NenaRika Ampuh HadigunaEvangelia SarantopoulouRiki TeslerEvangelos BakeasRikke GregersenEvanthia SakellariRikke JørgensenEvaristo Barrera AlgarínRima BreidokienėEvelia Franco ÁlvarezRima KregždytėEvgenia AnastasiouRinaldo PaarEvgenia CherouveimRinaldo PellicanoEvgenia OstroumovaRindone CorradoEvgenii KhailovRini Devijanti RidwanEvika KaramagioliRino BosnjakEwa FlorekRio Prasetyo LukodonoEwa HąciaRíona McArdleEwa JastezębskaRisa RobinsonEwa Joanna GodzińskaRisely Ferraz AlmeidaEwa KupcewiczRishabh Urvesh ShahEwa NiewiadomskaRishi SharmaEwa SadowyRishika SakariaEwa Szczepanska-SadowskaRita BergerEwa WasilewskaRita BiasiEwelina Czenczek- LewandowskaRita C. CroomsEwelina DziurkowskaRita Cássia Coelho De Almeida AkutsuEwelina NojszewskaRita Catalina Aquino CaregnatoEyal LewinRita ChiaramonteEzekiel GreenRita De Cássia AquinoFabián Fernández-LuqueñoRita De MatteisFabian SchwendingerRita DeeringFabrizio CedroneRita FranciscoFadal Abdullah-A. AldhufairiRita HendersonFadar Oliver OtiteRita KissFadhil Al-AboosiRita Maria Vittoria NobiliFady MansourRita MelendezFaming WangRita PatarrãoFan BruceRita Payan-CarreiraFanlei KongRita PolitoFarah IslamRita PoteyevaFárová KateřinaRita Rahoi-GilchrestFarzad AmirabdollahianRita SousaFatima El KhalloufiRitesh ChimoriyaFátima SilvaRittu HingoraniFátima Velez De CastroRizwan ShahidFay Al KhalifaRob BrooksFaysal Kabir ShuvoRob BrownFayuan WangRob EllamFederica ColliniRob VosFederica Di SpiritoRobbert HuijsmanFederico BenassiRobbin LindsayFederico CanaveseRobert A. MicklerFederico NalessoRobert Alan SloanFederico PennestrìRobert BarańskiFei LuoRobert BosFei WangRobert BoydFeili YangRobert BranstonFeilong WangRobert BrooksFelice SiricoRobert BuchananFelix Velicia-MartínRobert C. DempseyFeng LinRobert CharvatFeng-Chyi DuhRobert Cristian PurdoiuFeng-Jen TsaiRobert D. MorrisFengqing ChaoRobert D. StevensonFerdinando IellamoRobert DuPontFerenc IhaszRobert E. PittsFerenc RonkayRobert E. PykeFerenc VinczeRobert EdisFerencz BeataRobert EgermanFernanda M. SilvaRobert EllisFernando AlmeidaRobert F. MorganFernando Austria-CorralesRobert G. BestFernando CarvalhoRobert GajdaFernando LopesRobert GałązkowskiFernando P. CarvalhoRobert GawłowskiFerreira De Lucena Junior VicenteRobert GuthrieFerruccio ConteRobert HämmigFilipe RodriguesRobert HenningFiona Lugg-WidgerRobert HoerrFlorence M. WeierbachRobert J. BlountFloria PancettiRobert J. CopelandFlorian GambusRobert J. DonovanFlorian HeilmannRobert J. OrpetFlorian TheilmannRobert K. MlosekFlorin AonofrieseiRobert K. StallmanFoteini KyriaziRobert KankaFotis KitsiosRobert KotloskiFouillet CyrilRobert KrzysztofikFow-Sen ChoaRobert L. WilliamsFrances Mary JohnsonRobert LindeboomFrancesca GainoRobert M. BadeauFrancesca LareseRobert M. CariniFrancesca ManciantiRobert M. LafrenieFrancesca VichiRobert M. SiegelFrancesco BiancoRobert MitchellFrancesco BorgiaRobert MoehlerFrancesco CiodaroRobert MugerauerFrancesco Di GennaroRobert NewtonFrancesco GiallauriaRobert P. WoronieckiFrancesco InchingoloRobert PawlusińskiFrancesco NicoliRobert Peter GaleFrancesco StomeoRobert PodlasekFrancine Amaral PiubeliRobert PrillFrancine M. OvercashRobert PryceFrancisca Corpas-BurgosRobert RootFrancisca Jiménez-JiménezRobert SifrerFrancisco AlonsoRobert ŚliwowskiFrancisco Antonio Dos AnjosRobert SusloFrancisco CordobaRobert TaitFrancisco J. NievesRobert TamuraFrancisco Javier Herrero GutierrezRobert WalinderFrancisco Javier Lozano-ParraRobert WestFrancisco Javier Martín-AlmenaRobert WoodwardFrancisco Javier Povedano-MonteroRobert ZiółkowskiFrancisco Javier Rodríguez-LozanoRoberta AndreoliFrancisco Nieto EscámezRoberta BattiniFrancisco Pozo-MartinRoberta BulgariFrancisco TarazonaRoberta CapitelloFrancisco Triguero RuizRoberta CazzolaFranciszek GłówkaRoberta Cocci GrifoniFrançois Borel-HänniRoberta D’AurelioFrank BraatzRoberta De Souza SantosFrank ChenRoberta FoligniFrank LiRoberta FrontiniFranklin Wang-Ngai ChowRoberta GasparroFranz-Benjamin MocnikRoberta Hofman-CarisFred Van RaaijRoberta MerlantiFrédéric OuimetRoberta PisciaFritz-Gerald SchröderRoberta SalaFugang WangRoberta SistoFujiang HouRoberta ThompsonFumiharu TogoRoberto Alonso González LezcanoFumiya TanjiRoberto Augusto Ferriz-MartínezFung Sai-FuRoberto BaigorriFu-Sheng TsaiRoberto BarcalaG. Oliván-GonzalvoRoberto BenoniGábor GazdagRoberto BernasconiGábor MocsárRoberto CalistiGábor Szabó-SzentgrótiRoberto CannataroGabriel Claudiu TenderRoberto Carlos Castrejón-PérezGabriel Dominguez-MaldonadoRoberto CastiglioneGabriel GulisRoberto CattivelliGabriel ProdanRoberto CodellaGabriel RubinRoberto ColasantiGabriela AzocarRoberto ConsonniGabriela Chavez-CalvilloRoberto Di MarcoGabriela De CastroRoberto FornaraGabriela DimaRoberto FratiniGabriela Eleonora Moeller ChávezRoberto GeladoGabriela LinRoberto Giovanni Ramírez-ChavarríaGabriela RomanRoberto LatinaGabriela TopaRoberto LigroneGabriela WasileskiRoberto Lo GiudiceGabriele A. LandoltRoberto Martínez BeamonteGabriele Berg-BeckhoffRoberto Méndez SánchezGabriele CaviglioliRoberto MerlettiGabriele CentiniRoberto Nuño-SolinísGabriele ChiariRoberto Oliveira DantasGabriele TonniRoberto ParenteGabriella GraziusoRoberto PasiniGabriella PiscopoRoberto PierdiccaGabrielle JonesRoberto PiroGabrielle McKeeRoberto RizzoGaetano ChinniciRoberto RongoGaetano IsolaRoberto SalaGaetano MatontiRoberto Sanchez-CabreroGagan Deep SharmaRoberto SañudoGail Miriam MoraruRoberto ScicaliGailute KirdaiteRoberto Soto VarelaGal AvishaiRoberto ZefferinoGalateja JordakievaRobin A. WilsonGalina S. ZhamsuevaRobin C. Van Den HonertGamaliel BléRobin DodsonGanapathy NatarajanRobin HarrisGanapati SahooRobin HealyGang ChenRobin KobbeGang KouRobin Lewis CooperGang LiRobin PlaGang LinRobin SebastianGang PengRobin SouronGang SunRobles Blanca SanchezGangyan XuRoby GreenwaldGanisher DavlyatovRobyn A. LittlewoodGarazi Lopez De AnguiletaRobyn BurtonGarcía-Martínez InmaculadaRobyn GildenGareth DaleRobyn L. PrueittGareth JohnsonRocco Carmine ServidioGarfias MacedoRocco Di MicheleGarima DwivediRocco FrondiziGarry KuanRocco HaaseGary BoumaRocco PalumboGary BrewerRocco ServidioGary GaddisRochelle HentgesGary GlaubermanRocío CasañasGary HooperRocío Cruz-DíazGary HusbandRocio CupeiroGary J. MyersRocío De Andrés CalleGary M. RothenbergRocío De Diego CorderoGaryfallia KapravelouRocio Gallego-LosadaGaryfallia PeperaRocio Guil BozalGaurav GhagRocío Juliá-SanchisGaurav KumarRocío Llamas-RamosGaurav V. SarodeRocío Ortiz AmoGautam SrivastavaRocio Palomo-CarriónGauthaman SukumarRocío PérezGavin KearneyRocío Pineda-MartosGavin MoirRocio RodriguezGavriil George ArsoniadisRocío Romero-CastilloGayan RubasinghegeRocio Torres-ManceraGazi M. HassanRocío Valenzuela-NarváezGea Oliveri ContiRocío Vázquez-CobelaGea OlivericontiRoddy HiramGeert DomRoderick C. SliekerGeeske PeetersRodger J. ElbleGeeta DuppatiRodica Cristina ButnaruGelu OnoseRodman TurpinGema BarrientosRodney DuffetGema Pérez-RojoRodney P. JonesGema Sanchis-SolerRodolfo BuselliGemma María Gea-GarcíaRodolfo GattoGemma PratRodolfo MauceriGen NakayamaRodrigo Alejandro Hidalgo DattwylerGenee S. SmithRodrigo Braga MoruzziGenevieve BoisjolyRodrigo Elías ZambranoGennady KolesnikovRodrigo Feteira-SantosGennady OugolnitskyRodrigo García-LópezGennaro GalassoRodrigo HohlGennaro IasevoliRodrigo J. Carcedo GonzálezGennaro RuggieroRodrigo LimaGenny CarrilloRodrigo M. De CarvalhoGenovaitė LiobikienėRodrigo Martín San AgustínGeof RaynerRodrigo Poblete UmanzorGeoff LovellRodrigo Proença De OliveiraGeoffrey BabughiranaRodrigo RamalhoGeoffrey CrispRodrigo Rodrigues De FreitasGeoffrey D. KahnRodrigo SalasGeoffrey HudsonRodrigo Torres-CastroGeoffrey MadeiraRodrigo ValenzuelaGeon Ho BahnRodrigue PignelGeorg BolligRoel MerckxGeorg HalbeisenRoel Van OvermeireGeorg KrammerRoelien Du PlessisGeorg MattRogelio Bustamante-BelloGeorg SingerRogelio Flores-RamírezGeorg StüssiRogelio Gómez-GarcíaGeorg Von SchnurbeinRogelio Gónzalez-GónzalezGeorge A. GellertRoger AttwaterGeorge A. MarcoulidesRoger C. M. HoGeorge A. TsalisRoger CampdepadrosGeorge AntonogeorgosRoger ChongGeorge Calin DindeleganRoger E. ThomasGeorge CrooksRoger HoGeorge DrosatosRoger HurstGeorge E. KapellosRoger JensenGeorge E. MustoeRoger NasciGeorge H. CrockerRoger NybergGeorge JamilRoger O’SullivanGeorge JenningsRoger RamsbottomGeorge JîtcăRoger Saint-FortGeorge KorresRoger StrasserGeorge Laurenţiu Şerban-OprescuRoger WatsonGeorge LazaroiuRogério Leone BuchaimGeorge MaitiRogério RodriguesGeorge Mihai NitulescuRogie Royce CarandangGeorge MurrellRogier WoltjerGeorge N. HotosRohail HassanGeorge NotasRohan EdmondsGeorge P. SillupRohan RasiahGeorge PavlidisRohan SharmaGeorge R. KimRohit SrivastavaGeorge S. KorresRoisin LyonsGeorge TheodossiouRok CigličGeorge UmemotoRok TkavcGeorge ValasoulisRoksana MarkiewiczGeorge WooRoland DielGeorge YannisRoland GärtnerGeorge Z. KyzasRoland KochGeorges SalibaRoland Kueh Jui HengGeorges ZissisRoland Van Den TillaarGeorgia ChaselingRoland VeelkenGeorgia GomatouRolando G. Díaz-ZavalaGeorgia ManikaRolf HolleGeorgia PapoutsiRolf SøkildeGeorgia RowleyRoma JusienėGeorgina JohnstoneRomain ForestierGeorgina Mayela Núñez-RochaRoman B. LesykGeorgios A. PapadopoulosRoman CuberekGeorgios AndroutsopoulosRoman GablGeorgios BartzasRoman GabrhelíkGeorgios BenetosRoman KasparGeorgios BokiasRoman KrálikGeorgios GrivasRoman LeischikGeorgios IatrakisRomán MorenoGeorgios K. KoulinasRoman PavliukGeorgios KotzalidisRoman S. NagovitsynGeorgios MarinosRoman V. MoiseevGeorgios N. AntonoglouRoman VavrekGeorgios PapavasileiouRoman WölfelGeorgios PatsiaourasRomana Koberová IvančakováGeorgios SiorosRomana PylypchukGeorgios SylaiosRomanika OkraszewskaGeorgiy KorobeynikovRomina Alina MarcGeovani López-OrtizRomina D’AscanioGerald AranoffRomina FucàGerald ClamonRomina VivaldiGerald FerrettiRomuald ZalewskiGerald H. TomkinRomualdas MalinauskasGerald JeromeRómulo Jacobo González-GarcíaGerald MurrayRon KedemGerald ZernigRon SaulnierGeraldine TorrisiRona MacnivenGerard LevyRonald B. BrownGerard PasternakRonald BaganziGerardo Cebrián-TorrejónRonald EvansGerardo FebresRonald S. BrownGerardo Gold BouchotRonald SnarrGerardo Vázquez-MarrufoRonan LordanGerd LuppRónán O’CaoimhGerdt SundströmRonan Xavier CorrêaGergely GánicsRondy YuGerhard HobuschRonghuai HuangGerhard PölzlRongpeng ZhangGerhard VollandRoni UtriainenGerhard WinnekeRoopma WadhwaGerit PfuhlRoozbeh MirzaeiGerlinde MauererRosa AisaGermain HonvoRosa Angela FabioGermán EbenspergerRosa Angeles Vázquez-GarcíaGermán Gálvez-GarcíaRosa Cândida Carvalho Pereira De MeloGermán KopprioRosa Carla SilvaGerman PerdomoRosa D. DarlingGermano GallicchioRosa DireitoGermano RossiRosa Magallón-BotayaGermina-Alina CosmaRosa María Camacho-RuizGernot FuggerRosa Maria FanelliGero SzepannekRosa María Fuentes-RivasGerold LinkRosa María Goig MartínezGerry LushingtonRosa María López-Pintor MuñozGerson Lopes TeixeiraRosa Maria Mateos BernalGerti SziliRosa María Oliart-RosGertrude N. NyaabaRosa María Prol LedesmaGetahun Ersino LombamoRosa Maria Tapia-HaroGeun-Soo ParkRosa Martha Meda LaraGéza RipkaRosa Pilar Esteve FaubelGhassan HamraRosa Rosario-RosadoGheorge IliaRosa SammarcoGheorghe Nicusor PopRosa TorresGheorghița NistorRosa WongGhislaine MayerRosalba Argumedo-DeliraGholamali RahnavardRosalina Pisco CostaGholamreza DaryaborRosamaria Kostic-CisnerosGhulam Rasool MadniRosa-María Rodríguez-JiménezGhyslaine Bruna Djeunang DonghoRosana ShuhamaGiacinto BagettaRosangela Akemi HoshiGiacinto BarresiRosanna CousinsGiacomo BaimaRosanna GuarnieriGiacomo ChiesaRosaria BucciGiacomo FarìRosaria MeccarielloGiacomo NalliRosaria RuccoGiacomo OteriRosario G. AneraGiacomo PapottoRosario García-GiménezGiada AdelfioRosario J. MarreroGiada BenasiRosario Padial-RuzGiampaolo SantiRosario PastorGiampiera BulfoneRosario Valdez-SantiagoGiampiero MazzocchiRosaura Gonzalez-MendezGiampiero ParrinelloRose BakerGiampietro AlbertiRose BoucautGian Andrea PelliccioniRose CalixteGian Andrea RizziRose E. ConstantinoGian Franco CapraRose GilroyGian Luca MarellaRose LinesGian Luigi MarsegliaRose NjeminiGian Maria GaleazziRose-Anne LavergneGian Maria GrecoRoseli Rodrigues De MelloGian Paolo ClementeRosemary CaronGiancarlo CondelloRosemary GowranGiancarlo CuadraRosemary K. NabaweesiGiancarlo IannizzottoRosenberg J. RomeroGianCarlo PecorariRosendo BerengüiGiancarlo SperlìRoser GraneroGianfranco DamianiRoshanak MehdipanahGianfranco GalliRosie GibsonGianfranco MitacchioneRosie McEachenGiang PhamRosilda M. MussuryGianina LucaRoss BarnettGianluca CarusoRoss KoppelGianluca CatucciRoss ThomsonGianluca CiardiRossana C. ZepedaGianluca EspositoRossana Catie Bueno De GodoyGianluca LanzaRossana IzzettiGianluca M. TartagliaRossella LeopizziGianluca MiglioRossella LianiGianluca R. DamianiRossella MarmoGianluca RizzoRossella TomaiuoloGianluca SerafiniRossella TozziGianluca VergineRossiana BojinovaGianluigi BusicoRoswith RothGianluigi PastaRo-Ting LinGianmaria D’AddazioRowan MackenzieGianni Di GiorgioRowena IversGianni LoboscoRowena MerrittGianni PesRoxana Acosta GutiérrezGiannicola IannellaRoxana SiposGiannino MelottiRoxana StegaroiuGianpaolo ZammarchiRoxanne Suzette LorillaGianpiero GrecoRoy McConkeyGianpiero ZamperinRoy RobertsonGib UraipongRoy S. SmallGibson PraçaRozenn N. LemaitreGiedre SmailyteRozita HodGijsbert Van Der VoetRuaraidh DobsonGil Baptista FerreiraRubén Abraham Domínguez-PérezGil FerreiraRubén Arroyo Del BosqueGil Rodríguez-CaravacaRubén Camilo Lois-GonzálezGil SalvadorRubén Cuesta-BarriusoGil SerrancolíRúben FranciscoGilbert RamirezRuben Georges FukkinkGilberto RiveraRubén Gil-SolsonaGiles J. P. PeekRuben Jimenez-RedalGiles SioenRuben Lopez-BuenoGill A. Ten HoorRubén Muñoz PavónGill SchierhoutRubén Navarro PatónGillian B. AinsworthRubén Rivas-de-RocaGillian HatfieldRubén TriguerosGilson Adamczuk OliveiraRubén VillaresGina KruseRuchika BajajGina PiscitelloRüdiger KlapdorGina ToméRüdiger NüblingGina Yannitell ReinhardtRüdiger PryssGinevra GraviliRudolf KorinthenbergGinger KeRudolf KuklaGinny LaneRudy FoddisGintaras LabutisRuggero Andrisano RuggieriGintare KalinienėRuggero VigliaturoGintare VaznonieneRui C. CamposGioele CapilloRui ChangGioia BotessiRui DongGiordano FranceschiniRui Fonseca-PintoGiorgia BorrielloRui Jorge Garcia RamosGiorgia CapocasaleRui LiGiorgia Del FaveroRui MartinsGiorgia MiottoRui Miguel PauloGiorgia ProfumoRui NevesGiorgia ViciRui Pedro BritoGiorgina PiccoliRui Pedro FonsecaGiorgio BasileRui PoinhosGiorgio CesaraleRui SilvaGiorgio D. KotzalidisRui XueGiorgio LiguoriRuifeng HuGiorgio LombardoRuijie WangGiorgio SantoroRuilian ZhangGiorgio SmaldoneRuixue HouGiorgos P. TsironisRuiz-Herrera NoeliaGiosuè AnnibaliniRulan JiangGiovanna BarzanòRuman BanerjeeGiovanna CaparelloRune ElvikGiovanna CentorrinoRung-Tai LinGiovanna DeianaRunguo WuGiovanna PiangiamoreRunsen ZhangGiovanna RicciRunxia CaiGiovanna RizzoRun-Ze WuGiovanna TagliabueRunzhe GengGiovanna ZimatoreRuoliang TangGiovanni AudinoRuoxi WangGiovanni BarassiRupal ParekhGiovanni BarosiRupert ConradGiovanni BocciaRupert SmitGiovanni BombelliRupkatha BardhanGiovanni ChiariniRuriko YoshidaGiovanni CristalliRusan CatarGiovanni CugliariRuslan DorfmanGiovanni DalCorsoRussell EisenmanGiovanni DamianiRussell F. D’SouzaGiovanni De PergolaRussell James HoppGiovanni Dell`Aversana OrabonaRussell KabirGiovanni Freitas GomesRussell KirbyGiovanni GaleotoRussell R. PateGiovanni GerbinoRusso GiuseppeGiovanni GrandiRut Lucas-DomínguezGiovanni ImprotaRuta AdamonieneGiovanni LanteriRūta UstinavicieneGiovanni Li-DestriRute SampaioGiovanni MalerbaRute SantosGiovanni MartinottiRuth BreezeGiovanni MatareseRuth CunninghamGiovanni MatricaliRuth KaufmannGiovanni MuranoRuth L. SteinerGiovanni PaolinoRuth LunnGiovanni PecoraroRuth McCauslandGiovanni PeiraRuth McDermott-LevyGiovanni RollaRuth S. HwuGiovanni SogariRuth SnyderGiovanni TrisolinoRuth Taylor-PiliaeGiovanni VecchioRuud G. J. MeulenbroekGiridhar BabuRuut VeenhovenGisela Margarita Torres AcuñaRuxandra Sava-RosianuGisela Marta Teixeira De Sousa OliveiraRužena KrálíkováGisela Redondo-SamaRwik SenGisele ZancaRyad BouzouidjaGiseli MinattoRyad ZemouriGislaine Satyko KogureRyan AbmanGisoo ShinRyan ChapmanGiulia AgostiniRyan GiulianoGiulia BassiRyan HulteenGiulia CarusoRyan I. LoganGiulia CasuRyan LumberGiulia CatalisanoRyan Mead-HunterGiulia LambertiRyan ThalmanGiulia LausiRyan W. DobbsGiulia LorenzoniRyo HirabayashiGiulia LucertiniRyo InoueGiulia MatusaliRyo KinoshitaGiulia MenculiniRyo KohsakaGiulia MoreoRyo MaekawaGiulia PurpuraRyoma MichishitaGiulia RoderRyong Nam KimGiulia SimonettiRyosuke ShigematsuGiulia TetèRyota ShimokuraGiulia ZumboRyszard BraczkowskiGiuliana BrunoRyszard MalinowskiGiuliana VinciRyszard ŚwietlikGiulio CalcagniRyuichi OhtaGiulio DiMizioRyuichi SawaGiulio Francesco RomitiRyuji OchiaiGiulio PedriniRyusuke AeGiulio Petronio PetronioS. Cristina OanceaGiulio SenesS. KrammerGiulio ViceconteS. M. LabibGiuseppe Alessandro ScardinaS. M. Sohel MahmudGiuseppe AmorusoS. Pilar Zamora-LeónGiuseppe Angelo ZitoSa LiuGiuseppe AttanasiSa Ra LeeGiuseppe BanfiSaadet InanGiuseppe BarbatoSaahoon HongGiuseppe BattagliaSaba BattelinoGiuseppe BertozziSaba TabasumGiuseppe BorrusoSabahudin HadžialićGiuseppe CantisaniSabina Ampon-WirekoGiuseppe CiancioloSabina AngreckaGiuseppe ComentaleSabina Barrios-FernándezGiuseppe CoratellaSabina Kordana-ObuchGiuseppe CovielloSabina LicenGiuseppe D. SannaSabina PurkrtováGiuseppe De PalmaSabina SblanoGiuseppe Di MartinoSabina WagnerGiuseppe Di PietroSabina ZiembowiczGiuseppe GiudiceSabine KaiserGiuseppe GuidaSabine KestingGiuseppe LanzaSabine LemoyneGiuseppe LanzaroneSabine Panzer-KrauseGiuseppe Lo GiudiceSabine Sator-KatzenschlagerGiuseppe MagliuloSabine ZangeGiuseppe MancoSabrina BrescianiGiuseppe Marco TinaSabrina DemarieGiuseppe MarraroSabrina HermosillaGiuseppe Martino Di GiudaSabrina KastaunGiuseppe MasanottiSabrina KöchliGiuseppe MusumeciSabrina MargarucciGiuseppe NittiSabrina MolinaroGiuseppe PaceSabrina Rahman ArchieGiuseppe PalestraSabrina TabaresGiuseppe Piero GuidoSachi Nandan MohantyGiuseppe PonziniSachiko OzoneGiuseppe Potrick StefaniSachiko TakeharaGiuseppe QuagliaSachin KumarGiuseppe RicciSachin ThakurGiuseppe RinonapoliSachio TsuchidaGiuseppe Rocco CasaleSachit AnandGiuseppe SantarpinoSaddam Q. WaheedGiuseppe ScarattiSadegheh HaghshenasGiuseppe SimoneSadhana ShresthaGiuseppe T. CirellaSadhbh ByrneGiuseppe VaroneSadia HossainGiuseppe VarvaraSadok RezigGiuseppe VitoSae OchiGiuseppe ZappalàSaeed FarjamiGiuseppina CampisiSaeed KhakiGiuseppina LaganàSaeed MohammadiGiuseppina MalcangiSaeed ShaeriGiuseppina PappalardoSagar SohoniGiuseppina Pinuccia CerratoSagrario Gomez-CantarinoGiuseppina RuggieroSagrario Pérez- De La CruzGiuseppina SpanoSahar DerakhshanGiusi Antonia TotoSahar MihandoustGiustino VarrassiSaheed SabiuGizem AratSai K. RamaswamyGleice Margarete De Souza ConceiçãoSaid MekaryGlen CurrieSaida AbdiGlen NowakSai-Fu FungGlenn E. WeisfeldSaijun ZhangGlenn FinauSaioa Urrutia-GutierrezGlenn JohnsonSaionara Maria Aires Da CâmaraGlenn LedderSajjad ShiraziGloria AngelettiSakari LemolaGloria ArriagadaSakeenabi BashaGloria Bueno-LozanoSaket A. KottewarGloria González-CamposSakib-Bin AminGloria González-MedinaSakiko KanbaraGloria GuidettiSaku MäkinenGloria LuzzaniSalar ShaafGloria PascualSalik NazkiGloria Pérez-RubioSalina JantarangGloria Reig-GarciaSalla VenäläinenGnanachandrasamy GopalakrishnanSallie Beth JohnsonGniewko WięckiewiczSally Anne MortlockGobinath ShanmugamSally Fowler-DavisGodfred O. BoatengSally GuttmacherGodfrey B. WoelkSally MoyceGohari GholamrezaSally NashGoichi HagiwaraSally W. ThurstonGoki SudaSalma QasimGonçalo DiasSalvador Angosto SánchezGonzalo CruzSalvador Arias-SantiagoGonzalo Díaz-MenesesSalvador Boccaletti RamosGonzalo HaroSalvador Garcia-AyllonGonzalo Varas-DíazSalvador JaimeGopas JacobSalvador LlanaGopi McLeodSalvador NayaGopinath KrishnasamySalvador Perelló OliverGoran CirovicSalvador PérezGoran CuricSalvador Romero-ArenasGoran KišSalvatore CocuzzaGoran KuvačićSalvatore De BonisGöran LindahlSalvatore FasolaGoran SoderlundSalvatore Giovanni De SimoneGordana PavlišaSalvatore Giuseppe CocuzzaGordon HarperSalvatore LeonardiGordon J. M. PalmerSalvatore LopreviteGorka EpeldeSalvatore MonacoGorka VuletićSalvatore PirriGourav SharmaSalvatore RomanoGowtham K. AnnarapuSalvatore Santo SignorelliGoylette ChamiSalvatore ScaccoGözde Şengül AyçiçekSalvatore StrafaceGraça SoveralSalvatore ZaffinaGrace E. ShearrerSalwa G. MassadGrace Jean CampbellSam AtkinsonGrace Pui Yuk SzetoSam JohnsonGraciela Caire JuveraSam LauGraciela Padilla-CastilloSaman ShahidGrady NelsonSamaneh MadanianGrady RobertsSamantha C. SodergrenGraeme EdwardsSamantha CoulsonGraeme NorvalSamantha HueyGraham AshfordSamantha MajicGraham ChakafanaSamantha MoraisGraham R. McGinnisSamar G. MoussaGraham SmithSamar IssaGrasiele SausenSameer JoshiGrazia Daniela FemminellaSameera T. AhmedGrazia GrecoSameh W. Al-MuqdadiGrazia Lo SciutoSamer BagaeenGrazia NapoliSamuel Alexandre Almeida HonórioGraziana GaleoneSamuel BuxtonGraziano OnderSamuel DadaGraziano PinnaSamuel Fernández CarneroGrażyna BączekSamuel Fernández-SalineroGrazyna GosciniakSamuel MyersGrażyna Iwanowicz-PalusSamuel NutileGrażyna JanikowskaSamuel P. LeónGrazyna LietzauSamuel TomczykGrażyna Nowak-StarzSamuele CorteseGrażyna NowickaSamuele De PetrisGreg ElversSamuele MarinelloGreggory MaynardSamy MetyasGrégoire CattanSamy RimaGregor BurkhartSana Ullah JanGregorio Dal SassoSanaa Najeh Al-Haj AliGregorio GarciaSanaz ChamanaraGregorio GimenezSanaz KhalighiGregory CantrellSanaz NosratGregory H. AdlerSanchia ArandaGregory HeathSanda Mihaela PopescuGregory M. AnsteadSandeep JagtapGregory M. RebchookSandeep Kumar MishraGregory MitchellSandeep Sudhakaran NairGregory NeocleousSandeep VashistGregory S. SchoberSandeep YadavGregory SimonsSandesh PanthaGregory TourSandipan Roy ChowdhuryGreig TaylorSándor ValkaiGreta CapraraSandra AlhoyekGreta KrešićSandra AmadorGreta Veronica BerteselliSandra AndrušaitytėGretty VillenaSandra AppeltGrigorios L. KyriakopoulosSandra AvalosGrigorios NasiosSandra B. ProcterGrigoris AmoutziasSandra BudžakiGrzegorz CieslarSandra C. ButtigiegGrzegorz DrozdowskiSandra ChistoliniGrzegorz IgnatowskiSandra Clara SoaresGrzegorz IlewiczSandra CristinoGrzegorz KaczorSandra GavinhaGrzegorz KarońSandra GoffGrzegorz KinelskiSandra Jiménez-Del-BarrioGrzegorz MaciejewskiSandra Marcia MuxelGrzegorz MajewskiSandra McFaddenGrzegorz MichalskiSandra Oramas-RoyoGrzegorz NowickiSandra PerezGrzegorz PęczkowskiSandra PetrauskieneGrzegorz PolokSandra Racionero-PlazaGrzegorz SchroederSandra Regina AloucheGrzegorz SławińskiSandra RodriguesGrzegorz SzewczykSandra Sanmartin FeijooGrzegorz TeresińskiSandra SchüsslerGrzegorz TrybekSandra Soto-HerasGrzegorz TrzcińskiSandra ThompsonGrzegorz ZielińskiSandra VujkovGrzegorz ŻurekSandra ZampieriGuadalupe Hernández EscobedoSandro Arrufat-MartínGuadalupe Martín-Mora ParraSandro BartolomeiGuadalupe Molina TorresSandro Fernandes Da SilvaGuang XuSandro SerpaGuangai XueSandy K. WurteleGuangdi LiSandy L. FurtererGuanghua ChiSang Hoon KimGuangjie HanSang Hun KimGuangrong ShenSang Ouk WeeGuangwei HuangSang Suk KimGuan-Ting PanSangchun ChoiGuan-Zheng LiuSang‐Dol KimGudrun WagnerSangdon KimGuennadi SaikoSangeeta Chatterji ChatterjiGuenther KahlertSang-Han LeeGuey-Chuen PerngSang-Ho KimGuglielmo CampusSanghoon ByeonGuglielmo DurantiSanghoon KangGuglielmo RamieriSanghyuk BaeGuglielmo StabileSang-Joon KimGuia MorelliSang-Seok NamGuicai LiuSangu MuthurajuGuida Da PonteSanja Music MilanovicGuido CaluoriSanja Potgieter-VermaakGuido CorradiSanjay KalraGuido ErreygersSanjay KumarGuido Franco AmorettiSanjay RaoGuido LombardoSanjay SinghGuido VanhamSanjeevan MuruganandanGuido VielSanjoy SahaGuilaine JariaSanjucta DuttaGuilherme FurtadoSankaran DevimeenakshiGuilherme MalafaiaSankaran RajendranGuilherme Veiga GuimarãesSanna StrothGuillaume E. CourtoySanne F. E. RoversGuillaume GiraudSanne VeldmanGuillaume MornieuxSanneke De La RieGuillaume PelletierSano TomohitoGuillem Marca-FrancèsSanta ParrelloGuillermina Guerrero MoraSantanu MetiaGuillermo A. Cañadas-De La FuenteSanthosh MukundanGuillermo BorragánSantiago BonanadGuillermo Espinosa-ReyesSantiago GasconGuillermo Felipe López SánchezSantiago Giraldo LuqueGuillermo Garcia-GarciaSantiago M. LopezGuillermo LaheraSantiago Martínez-IsasiGuillermo MoreirasSantiago MengualGuillermo PetzoldSantiago MoraGuillermo PlazaSantiago TejedorGuillermo Ronquillo LomelíSantiago VeigaGuiming ZhangSantiago ZabaloyGuinevere GraniteSanto CatapanoGuining LuSantos VillafainaGulasekaran RajaguruSantosh DhakalGulzar ShahSantoso Cornelia Melinda AdiGun LiSaoirse Nic GabhainnGuna LeeSapana LohaniGunita DeksneSapna SharmaGunn Marit AasvangSara AlmeidaGunnar CerwénSara Al-MusharafGunnar KristensenSara B. ChadwickGunnar SæbøSara B. DonevantGunnar ToftSara BernardiGünter BärwolffSara CampagnaGunter LauxSara CervaiGünter MüllerSara ContiGunther HempelSara Contreras-MartosGüntülü AkSara Cortés AmadorGuochun WuSara CroxfordGuodong ZhangSara Degli-EspostiGuofeng ShenSara Domínguez-LloriaGuoqi QianSara E. GrineskiGuoqing HuSara Estecha QuerolGuor-Cheng FangSara FilipiakGuoxing LiSara GarfieldGuoxun ChenSara GonzalezGuoying ZhangSara Huerta-GonzálezGurupreet SethiSara IserniaGustavo Adolfo MuñozSara Larsson LönnGustavo AmorimSara Lesley WarberGustavo Augusto LacorteSara MaioGustavo DesouzartSara MaisanabaGustavo Duarte PimentelSara Malo FumanalGustavo FernandesSara MesquitaGustavo Gonzales RengifoSara Moreno CamaraGustavo MattaSara MoscatelliGustavo MeschSara MucherinoGustavo PimentelSara NamaziGustavo Ribeiro Da MotaSara NunesGustavo Rodríguez FuentesSara Ojeda-BenitezGustavo Vieira De OliveiraSara OttolenghiGustavo Zen De Figueiredo NevesSara ReisGuy TraininSara RicciardiGuzel BikbovaSara RodriguesGwen BrekelmansSara Rodriguez-CuadradoGwen D’ArcangelisSara SantiniGwénolé LoasSara SilaGwenyth R. WallenSara VertaldiGyanendra GongalSara VillaGyouhyung KyungSarabjit MastanaGyula NagySarah A. FelknorGyula SzaboSarah AherfiH. C. Ananda MurthySarah B. WhorleyH. Peter SoyerSarah BlackstoneHa HoangSarah BrowneHabil. Agnieszka Dettlaff-PokoraSarah Cohen-GogoHada Fongha IeongSarah CookHadi Akbarzadeh KhorshidiSarah DeemerHadi HasankhaniSarah DoddHadi NobariSarah E. KnowlesHadiar RahmanSarah FloydHadiyanto HadiyantoSarah FriedrichHady KeitaSarah GooddayHae Ran KimSarah HillHae Won KimSarah HydeHae Young KimSarah I. M. JanusHaein LeeSarah KellerHae-Jin KoSarah KellyHaekyung Jeon-SlaughterSarah KendalHaeryun ChoSarah Kittel-SchneiderHae-Sung NamSarah LehmanHaewon ByeonSarah LittleHae-Young KimSarah LutzHafiz M. N. IqbalSarah M. Dos AnjosHaifeng DengSarah M. RabbittHai-Hua ChuangSarah MaloneyHaijie TongSarah ManessHaiqiu HuangSarah MeyerHaitao YuanSarah MillikenHaitham KhatatbehSarah MisyakHaitham NobaneeSarah MooreHaixiao Li‪Sarah MyersHaizal HussainiSarah PriorHajime IwasaSarah RedshawHajime KamebuchiSarah RileyHajime KojimaSarah SnuggsHakjoon SongSarah SurberHale M. ThompsonSarah Treves-KaganHalil İbrahim CeylanSarah W. WhittonHalina Kowalewska-KalkowskaSarah WillisHalina StaniekSarahRose BlackHalvard BönigSarantos KapidakisHalyna MishchukSaranya PalaniswamyHamada ElwanSaranya VeluswamyHamdi ChtourouSarassunta UcciHamed BostanSarath VijayakumarHamed Najafi AlamdarloSarbjit SinghHamid BakshiSardar S. ShareefHamid ShahandehSarfaraz AdnanHamidah HussainSarfaraz Hashemkhani ZolfaniHamidreza Rabiei-DastjerdiSari CastrénHampapuram RamapriyanSaria HassanHamza El HadiSartaj Ahmad BhatHan Joo KimSaruhan MoslerHan YueSarvath AliHana M. DobrovolnySaša ĆukovićHana Mahmutefendić LučinSascha NeumannHana MohelskaSasha ScamblerHana PacaiovaSaskia FischerHanadi Y. HamadiSaskia L. Wilson-BarnesHanbai ParkSaskia Maria De GaniHanbin HuangSaskia Te VeldeHanchen YuSatabdi ChatterjeeHandan XiangSatarupa DasguptaHandi Chandra PutraSathishkumar RamalingamHang QuSatoru KobayashiHang XingSatoru MunakataHanga GalfalvySatoru SaitoHangsub ChoiSatoru TsujiiHani Amir AouissiSatoshi KumakuraHani MorganSatoshi NinomiyaHanifa BachouSatoshi OsukaHank HallegraeffSatoshi SaitoHankiz DolanSatoshi UchidaHanna BurkhalterSatoshi UekiHanna DudekSatoshi YokoseHanna GerberSatoshi YuguchiHanna Górska-WarsewiczSatu Kajander-UnkuriHanna HuebnerSatu KalliolaHan-Na KimSatu NivalainenHanna KustererSaturnino Marco LupiHanna LiberskaSatvika BurugupalliHanna M. PituchSatwinder Kaur BainsHanna Michniewicz-AnkiersztajnSaudade LopesHanna NaleczSaugato Rahman DhrubaHanna Przybyła-BasistaSaujanya KarkiHanna RozenekSauk-Hee ParkHannah BadlandSaul BloxhamHannah ClarkeSaúl Torres-OrtegaHannah GossSaulius RočkaHannah H. CovertSaumik Saumik PanjaHannah HeathSaverio CainiHannah HendersonSaverio MuscoliHannah KuperSavina SchoenhoferHannah ThorneSawako OkamotoHannah WechkunanukulSawsan A. ZaitoneHannakaisa Niela‐VilénSayed ElhoushyHannamaria KuusioSayyed Ali SamadiHanne KonradsenSayyed Fawad Ali ShahHanno ErythropelSchiavone MarcoHanno S. MeyerSchweder ToreHanns De La FuenteScott CurtisHanns MoshammerScott D. GrosseHannu LaurilaScott D. TagliaferriHan-Ping ShiScott DevenishHans BinderScott DrumHans BosmaScott M. DebbHans De CockScott M. PickettHans H. StassenScott MazzettiHans HobbelenScott McdonaldHans Van De VisScott OgletreeHans Van EyghenScott R. SandersHans WiesmethScott SpreatHans-Christian KolbergSe Jin ParkHans-Christian SiebertSeahwa WonHans-Eggert ReimersSean ClarkHans-Gert BernsteinSean CollinsHans-Guido MückeSean G. YoungHan-Shen ChenSean MacDermottHanvedes DaovisanSean RichardsHany HassaninSebastià Galmés MonroigHanyong PengSebastià Verger GelabertHao ChengSebastian BernatHao LiSebastian BöttgerHao PengSebastian Cǎlin VacHao XiongSebastian D. SchaeferHaobijam BasantaSebastian DiezHaojie LiuSebastian DittmeierHaoran WangSebastián Espoz-LazoHaorui WuSebastian GehlertHao-Yun KaoSebastian Ion CeptureanuHappy TshivhulaSebastian KlichHarada KoujiSebastian KotHarada NahokoSebastian LucasHarald StummerSebastian LuningHarapan HarapanSebastian MaderHari KandelSebastian MajewskiHari PalaniSebastian NoeHari Prasad DevkotaSebastian RuinHari S. IyerSebastián Sánchez CastilloHari S. LuitelSebastian SchnaubeltHariprasada R. Kanna ReddySebastian StanHaris PojskicSebastian StępieńHarishchandra DubeySebastiano CiccoHarold HorellSebastiano CostaHarold J. FarberSebastiano D’UrsoHarold KincaidSébastien BaillyHarri H. HakovirtaSebastien ChastinHarri HelajärviSébastien UrbenHarriet MorfSebastjan BevcHarriet OkatchSebastjan ŠkerličHarry H. X. WangSefika KiziltoprakHarsh DikshitSégolène GambertHarshit MahandraSehar ZulfiqarHarshvardhan SinghSeidu SofoHartmut F. WitteSeiji MiyauchiHaruhiko (Hal) InadaSeiji ShibataHaruhiko ImamuraSeijun LimHaruhiko SatoSeiko MiyataHaruki KoikeSeil KimHaruki ShimazuSeitaro SuzukiHarvey LinSeka LazareHarvinder Singh ChhabraSekar VijayakumarHasan AbdulrahmanSeki MasafumiHasanzadeh MahdiSelcuk KucukseymenHashem Salarzadeh JenatabadiSelene Valero-MorenoHasmun NorenSelenia Di FronsoHasnaa JalouSelicia MayraHasnan BaberSelma Adnan SaadaldinHassan DoostiSelma De CastilhoHassan El-RamadySelma Gouvêa De BarrosHassan SaghiSelvaraj JayaramanHassan VallySelvasankar MurugesanHassan ZiadaSema G. QuadirHassanah HusinSema K. AydedeHassen S. WolleboSemida SilveiraHatice AtilganSenhu WangHattaf KhalidSenjooti RoyHaukur Freyr GylfasonSenol PiskinHåvard J. HaugenSenthilkumar SankararamanHayato TsukamotoSenwei TsaiHayden HedmanSeo Hu LeeHayley ChristianSeockhoon ChungHayley PierceSeock-Jin HongHayley Smithers-SheedySeok-Ki JungHayrettin OkutSeol-A KwonHazel DaltonSeon Young HwangHazell KarenSeon Young RyuHazuki TamadaSeonaid CleareHe BuSeon-Chil KimHealAnastasius MoumtzoglouSeong Hoon BaeHeather BurrisSeong Man ParkHeather D’AngeloSeong Nam HwangHeather J. BaldwinSeong-Kyu KangHeather J. WilliamsonSeoul-Hee NamHeather K. HudsonSepanta HosseinpourHeather L. BurrowsSepehr AlizadehsalehiHeather L. TeagueSepideh KhoshnevisHeather Leduc-PessahSerafín Moral-GarcíaHeather M. A. FentonSerafín MurilloHeather M. Logan-SprengerSerajus SalaheenHeather M. PadillaSerdal TemelHeather Morris-EytonSerdar DurdyevHeather Nicole StoneSerena BianchiHeather Norman-BurgdolfSerena CavalleroHeather SprengerSerena ClarkHeather WardleSerena DattolaHeba Jafar SabbaghSerena DonatiHeberto PriegoSerena GrumiHebin LinSerena MottolaHéctor Beltrán-AlacreuSerena PetrocchiHéctor BurgosSerge BrandHector Campello-VicenteSerge BriançonHéctor CarrascoSerge PergamenchtchikovHector ChikooreSergei ChernyakHéctor García- LópezSergei IgumnovHéctor Hugo Pérez-VillarrealSergey MaksimovHéctor Jirau-ColónSergey PustylnikovHéctor Pifarré ArolasSergey SosnovskikhHector Riveros-RosasSergey Valentinovich BorisovHéctor Saldaña MárquezSergey Vasin MikhailovichHee Il LeeSergey ZhironkinHee Sun KangSergi Garcia-RetortilloHee Sun KimSergi MatasHee Young LeeSergio A. SilverioHeeSoon LeeSergio A. UsecheHeesup HanSergio Barrientos-TrigoHeewon JungSergio BernasconiHee-Young KimSergio BoniniHeidi HillmanSergio Calonge-PascualHeidi K. OrtmeyerSergio CastellanoHeidi L. DempseySergio Cinza-SanjurjoHeidi MosesonSergio ContrerasHeidrun SturmSergio Cuevas-CovarrubiasHeike WieserSergio E. RodriguezHeikki RäisänenSergio Ibarra EspinosaHeini VäisänenSergio Jiménez-RubioHeinrich GerdingSergio Jose IbáñezHeinrich WeberSergio Lins De-Azevedo-VazHeinz Friedrich SchölerSergio Lo CaputoHeinz KleinöderSergio López-GarcíaHeitor PellegrinaSergio Luján-MoraHelaine AlessioSergio Ochoa JiménezHélder LopesSergio Rios-AguilarHelder OliveiraSergio SambataroHelder RelvasSergio SantaeufemiaHelen AlmondSergio Selles-PerezHelen BranthwaiteSergio SpinatoHelen CoddSerhat AsciHelen CollinsSerkan KaradasHelen CowieSerkan UsguHelen CrabbeSeth EbersvillerHelen Ellis-CairdSeth JennyHelen GriffithsSeth M. ArmahHelen KoechlinSeth M. TarrantHelen L. ReevesSeul Ki LeeHelen LewisSeulkee HeoHelen McaneneySeung Gu ShinHelen RutlidgeSeung Hwan KimHelen ShultzSeung-Eun ChaHelena Akemi Wada WatanabeSeung-Gyu SimHelena Belchior RochaSeung-Ha LeeHelena CatarinoSeunghoo LimHelena De SolaSeunghyun HwangHelena Domínguez TorresSeungJin BaeHelena DudyczSeung-Ju KimHelena FidlerovaSeung-Nam KimHelena GapeyevaSeungwon JeongHelena Giannakaki-ZimmermannSeungwon KwonHelena JoséSeungwoo HanHelena LenasiSeung-Yong JeongHelena María Romay-BarreroSeungyong LeeHelena MartynowiczSeung-Yoon RheeHelena NadaisSevaste ChatzifotiouHelena RibeiroSevda AslanHelena SchuchSevenpri CandraHelene Diana ConnorSeverin HornungHélène GorgeSeverino Jefferson Ribeiro SilvaHélène JeulinSeverino PersechinoHelge StalsbergSevero Vázquez-PrietoHeli SiltalaSevilay Tümkaya YilmazHeli VirtanenSeyed Behzad JazayeriHelianthe KortSeyed Khosrow TayebatiHelio Junji ShimozakoSeyed Omid NabaviHelmet T. KarimSeyede Fatemeh GhoreishiHelmut FrohnhofenSe-Youn JungHelmut GreimShaaban ElnesrHelvio AffonsoShaffa HameedHeng ZhangShafie Rosli MohdHengjie ZhangShagufta GaffarHenin DolajiShahab E. SaqibHenita RahmayantiShahabaldin RezaniaHenk KoppelaarShahar Lev-AriHenna MuzaffarShaher A. I. ShalfawiHennie FisherShahid UllahHenning ArltShahieda AdamsHenning BuddeShahnaz AzizHenning StrubeltShahrokh RahbariHennning WiltsShaikh EskanderHénock Blaise Nguendo-YongsiShailendra MauryaHenri TilgaShailendra Pratap SinghHenrieta HorníkováShakila DadaHenriette Löffler-StastkaShaku NairHenriëtte PrastShalaka MulherkarHenrik C. BäckerShally NovitaHenrik KoblauchShamaila RafiqHenrique CastroShambhu YadavHenrique NascimentoShamik ChakrabortyHenrique P. NeivaShamik ShahHenrique PereiraShamika Ann MitchellHenrique SilvaShaminie AthinarayananHenry C. Y. HoShamshad KaratelaHenry Dushan E. AtkinsonShamsul Azahari Zainal BadariHenry GriffithShan JiangHenry H. L. WuShan MohammedHenry InegbedionShan SuHenry J. NussShandell ElmerHenry LukaskiShane L. RogersHenry MunyandukiShane McIverHenry PleassShane Niall O’ HigginsHenry SutantoShane SandersHenry T. PengShangfeng TangHenry WillifordShan-Huey YuHenryk DudaShanie JayasingheHenryk KrawczykShannon B. JuengstHepi H. HandayaniShannon CiganHerbert ChikafuShannon GrayHerbert L. MeiselmanShannon GruganHerbert LöllgenShannon HayesHerbert Ryan MariniShannon JarrottHerbert TomasoShannon M. RobsonHerbert WirthShannon S. D. BredinHeribert Harald FreudenthalerShanquan ChenHeriberto Antonio Pineda-EspejelShantanu Kumar PaniHermano Alexandre Lima RochaShanton ChangHersília De Andrade E. SantosShanyuanye GuanHervé MénardShaobin WangHerwig CerwenkaShaobo WangHesekia GarekaeShaojing FanHessam GolmohamadiShaoju WuHeverton DutraShaotong ZhuHicham AchebakShaouli ShahidHicham SidShaoyong LuHideaki KawabataShao-Yuan ChuangHidekazu NishimuraSharanya KalasekarHideki FujiiSharifu UraHideki HayashidaniSharma SachinHideki KitauraSharmistha MitraHideki SekiyaSharna MathieuHideki TakaiSharon AndersonHideki TakebayashiSharon BarakHideko SoneSharon CoxHidenori HaradaSharon E. BarrettHideo HashizumeSharon GrantHideo KatoSharon L. LarsonHie-Won HannSharon LawnHieyong JeongSharon Maleki FarHiginio González-GarcíaSharon ManneHigor LeiteSharon RennerHilario BecerrilSharon SmithHilary DeShongSharon StollHilary L. SurrattSharon VincentHilary Piedrahita-ValdésSharyn GaskinHilary PiercyShaun GoldfinchHilde PapeShaun SabicoHillary K. KiprutoShawn BeaudetteHilton Pereira Da SilvaShawn DrakeHimanshu DeshwalShawn J. McCoyHimanshu KathuriaShawn TalbotHina GargShayne Baker OAMHipólito PérezSheeba JacobHirayuki EnomotoSheeba ThomasHiro KishimotoSheela RaoHiroaki FunahashiSheena E. MarteniesHiroaki KawamuraSheikh AlifHirofumi ToyotaSheila FleischhackerHirokazu KimuraSheila GlennHiroki NakaharaSheila M. CampbellHiroki TeragawaSheila Sánchez CastilloHiroko KawanamiSheila SandersHiroko MiuraSheina EmraniHiroko OchiaiSheketha R. HauserHiromasa HasegawaSheldon MorrisHiromichi ItoShelia FleischhackerHiromichi NaitoShelly J. LaneHiromitsu KobayashiShen ZhangHironori KatoSheng ZhangHiroshi AkimaSheng-Dean LuoHiroshi ChureiSheng-Fan WangHiroshi KadotaniShenghong HeHiroshi KatagiriSheng-Yow HoHiroshi KinoshitaShengzhi SunHiroshi MatsumotoShenmeng XuHiroshi OkochiSheo PandeyHiroshi WatabeShereen HusseinHiroshi YokomichiSheri RowlandHiroshi YoshitakeSherif GoubranHirotaka TanabeSherri StastnyHiroto HondaSherry HambyHiroto TerasakiShervin HashemiHiroyuki NakamuraShervin MinaeeHiroyuki NodaSheryl Van NunenHiroyuki TaguchiSherzod N. TashpulatovHiroyuki WakiguchiSherzod TashpulatovHisashi YoshimotoShi ChenHisato YagiShi ShenHisayoshi OkamuraShiamaa AlmashhadaniHisham BahmadShibili NuhmaniHisham FansaShibin ChengHitomu KotaniShibly ShahrierHitoshi KondoShicheng LiHitoshi MochizukiShigeho TanakaHiu-Yee KwanShigekazu SuginoHo Hon LeungShigeki TakedaHo Manh ToanShigeki YamaguchiHo Sun ShonShigemitsu SakumaHo Ting WongShigeo IijimaHo Yin Eric LauShih-An LiuHoang Phong LeShih-Bin SuHocine BougdahShih-Chieh ChenHoi CheuShih-Chih ChenHoichi AmanoShih-Chun LungHolger FröhlichShihlung ChenHolger WatterShihoko Komine-AizawaHolger WeissShih-Tse WangHolli Loomans-KroppShih-Yi HuangHollis LaiShikha PrashadHollis O’NealShilpa DograHolly B. Herberman MashShilpa TejpalHolly GaddShin ArakiHolly J. TravisShin Jun ParkHolly KihmShin Kue RyuHomero MartinezShinae AhnHon Lon TamShinae KimHong LinShin-Cheh ChenHong ShenShing-Hwa LiuHong Sheng ChengShing-Jye ChenHong XueShinichi EgawaHongbin DengShin-Ichi NakakitaHongbo WangShinichi SobueHongchang LiShinichi YoshiokaHongchao ZhangShinichiro MorichiHonghyok KimShinichiro OchiHongjie LiuShinji OtaniHongjun LiShino Homma-TakedaHong-Man HouShinobu TakizawaHongming ShanShinsuke MizutaniHongpeng GuoShinta NishiokaHongsun GuoShintaro SengokuHongwei WanShintaro SukegawaHongwu WangShin-Ting YehHongyan ChaiShin-Tsu ChangHongyi ZhouShinwoo ChoiHongying Lilian TangShinya InazumiHongying PanShinyoung JunHong-Youl HaShiping BaiHonorata HowaniecShiran BordHon-Yi ShiShiri Shinan-AltmanHoon KimShirit Kamil-RosenbergHoppe TheresaShirley WyverHor Yan Angel LaiShiro MizunoHoracio FirminoShirra FreemanHoracio Molina-SánchezShisong RongHoracio Riojas RodriguezShiv Prakash VermaHorațiu CatalanoShivam GuptaHorea George CrisanShivendra KumarHoria HaragusShixiong ZhangHortencia Silva-JímenezShizuo KatamotoHossam A. GabbarShmuel ArnonHossein ZareSho TakahashiHosung ShinShogo OzawaHouda El-MustaphaShoji MasahiroHoward BurdettShojiro SawadaHoward WilliamsonShoshanna SofaerHrvoje BudinčevićShoshi KeisariHrvoje GrofelnikShou-Heng LiuHrvoje KarninčićShoumitro (Shoumi) DebHrvoje KlobučarShouro DasguptaHsiang-Yin ChenShovan NaskarHsiao-Chun TsengShreoshi Pal ChoudhuriHsiao-Hsien LinShreya TocaciuHsiao-Yu YangShrutilipi BhattacharjeeHsien-Wei TingShuaijun GuoHsien-Wen KuoShuang LiHsi-Hsien YangShuanning ZhengHsin-Chi KoShuanping DaiHsin-Fang ChungShubham AgrawalHsing-Chun KuoShubin YuHsin-Ta HsuehShue WangHsin-Tang LinShuen Yee LeeHsin-Yi Kathy ChengShufa DuHsin-Yi YangShu-Fen KuoHsin-Yuan ChenShuhei TakaujiHsiu-Chin HsiehShuichi IshidaHsiu-Ching ChiuShuichi UenoHsiu-Fen HsiehShuk Yi Annie HuiHsiu-Tzu Betty ChangShuko NojiriHsiu-Yueh LiuShukun ChenHsuan-Chu ChenShulamit RamonHsun-Yu ChanShu-Ling TzengHu LiuShu-Lung KuoHua QinShun WangHua-Dong YaoShungo ImaiHua-Fang LiaoShun-Hsing ChenHuajun LiangShunichi HondaHualiang LinShunsuke KidaniHuan GuShunxing LiangHuang XiaoyanShuqi ZhangHuanyu ChengShusuke HiragiHuatian WangShuwen Karen LiuHuayi HuangShuxiong ChenHubert ZiółkowskiShu-Yao TsaiHubertus HimmerichShu-Yen WanHudson Alves PintoShu-Yi LiawHudson KingstonShweta KukrejaHuei-Ling ChiuShyamal DasHuey-Hong HsiehShyamala ThirunavukkarasuHugh DevlinShyamalagauri JadhavHugh GashShyue-Cherng LiawHugh Robert WatsonSiân De BellHugh WhiteleySib GiriHugo A. KerhervéSibylle ErmlerHugo Canas-SimiãoSibylle HeilbrunnHugo Consciência SilvestreSibylle KranzHugo Leiria NevesSibylle L. HildenbrandHugo LouroSiddharth JaggavarapuHugo Machado SanchezSiddharth NarayananHugo OlmedillasSiddharth ShuklaHugo R. MartinezSidney LungHugo Saldarriaga-NoreñaSidsel BoldermoHugo SarmentoSiemowit MuszyńskiHugo SimkinSiew Mooi ChingHugo UlloaSiew Yim LohHugo Valadares SiqueiraSigal Eilat-AdarHugo Wai Leung MakSigan L. HartleyHugues Allard-ChamardSigitas UrbonavičiusHuib CornieljeSigne TomsoneHuibert BurgerSignorelli CarloHuibin LvSigrid Kusch-BrandtHui-Chi LiSigrid Müller-DeubertHui-Chuan HsuSigrid SalomonssonHui-Hsun ChiangSigríður HalldórsdóttirHuijia LiSiim MaasikamäeHuijing ShiSile A. CreedonHui-Ju LinSilke Anna Theresa WeberHui-Ju YoungSilke Von EsenweinHuijun ChihSilva JoãoHui-Ting YangSilvana Alves PereiraHumberto CarvalhoSilvana M. O. SantinHumberto Javier Rodríguez HernándezSilvana MarevaHumberto S. MachadoSilvana Maria Russo WatsonHung Vo ThanhSilvana Regina Perez OrricoHung Vuong PhamSilvana RevollarHung-Chang LiaoSilvana S. BettiolHung-Chuan PanSilvana SecinaroHung-Lung ChiangSilvana WeberHung-Pin TuSilvano DragonieriHung-Vu TranSilvano GallusHung-Yi ChuangSilverio AlarcónHunter BennettSilverio RotondiHunter MartaindaleSilvia Alejandra García-GascaHunter ParisSílvia Almeida CardosoHun-Young ParkSilvia Arribas GalagarraHuong Thu NguyenSilvia AuletHusam RjoubSilvia BecagliHussein BasmaSilvia BonettaHwa-Jung KimSilvia BrunoroHyang Yuol LeeSilvia CapuaniHyang-In Cho ChungSilvia Carrascal DomínguezHyang-Soon OhSilvia CasiniHye Young AhnSilvia CastanyHyechang RhimSilvia CeccacciHyen Oh LaSilvia ChichiarelliHyeon-Cheol KimSilvia ColnaghiHyeong Suk NaSilvia De Miguel-BilbaoHyeongsu KimSilvia DíazHye-Ryoung KimSilvia Escribano CubasHye-Ryun KimSilvia FrancisciHyo Geun ChoiSilvia GiannattasioHyo Soung ChaSilvia Helena De Carvalho Sales-PeresHyo Sun JungSilvia IvaldiHyo-Bang MoonSilvia KrummHyogo HoriguchiSílvia LopesHyojin ImSilvia MarcuHyojin KimSilvia Maria MarcheseHyokeun LeeSilvia MascoloHyoung Chul ShinSilvia Miriam CammarataHyoung Eun MoonSilvia Molina-RoldánHyoung-Kil KangSilvia MonacoHyoung-Shik ShinSilvia MonteiroHyuk-Yoon KwonSilvia Morales ChainesHyuma MakizakoSilvia MoscatelliHyun Chul JungSilvia NievaHyun Goo KimSilvia PancaniHyun Jin LeeSilvia PerzolliHyun Seok JeeSilvia PlataniaHyun Suk LeeSilvia Portero De La CruzHyunchul ChoiSilvia Postigo-ZegarraHyun-Duck KimSilvia PuiuHyung Keun AhnSilvia QuadroniHyung Rok WooSilvia RigatoHyunggoo KangSilvia RiondinoHyungjong ParkSilvia RocchiccioliHyungkyoo KimSílvia Rocha-RodriguesHyung-Mi ChoSilvia San Román MataHyungtae KimSilvia SantiniHyunGyung JooSilvia ScaglioniHyun-Hee HeoSilvia SoleHyun-Jae ChoSilvia SoléHyun-Jin MinSilvia SpivakovskyHyunju ParkSilvia SumedreaHyunjung KimSilvia Ubillos-LandaHyun-Kyoung KimSilvija ŠiljegHyunwoo HwangboSilvije DavilaIain A. BrownleeSilvio Assis De Oliveira-JuniorIain FletcherSilvio LorenzettiIain McLellanSilvio MaltagliatiIain ScottSilvio MaringhiniIan CumminsSilvio NoceraIan HaySilvio VeigaIan HodgsonSilviu RogobeteIan JohnsonSima NaminIan LahartSime VersicIan M. KatzSimeon GillIan NewhouseSimion BranIan PikeSimon BishopIan R. FalconerSimon ConroyIana MarkevychSimon DedmanIbo H. SouwerSimon FoxIbolya AndrásSimon G. PatchingIbolya FülöpSimon GriffithsIbou ThiorSimon KilbaneIbraheem KarayeSimon KrašnaIbraheem M. KarayeSimon LittlewoodIbrahim AgwaSimon MairIbrahim HassanSimon O’LearyIbrahim M. SayedSimón PeñaIbrahim SonmezSimon PorcherIbsen B. CoimbraSimon RauchIbukun OluwoyeSimon ReifIchiro WakabayashiSimon WattIchiro YamadaSimon WeigmannIciar ArteagoitiaSimona BalazsIda CariatiSimona BungauIda JärlskogSimona Cătălina ȘtefanIdiano D’AdamoSimona ČinčalováIdlir LicajSimona CrognaleIe-Bin LianSimona Ioana GhitaIeva StončikaitėSimona JancikovaIffat ElbaraziSimona Jurkovic MlakarIffath Unissa SyedSimona KutiIgal IferganSimona L. SchlerethIghli Di BariSimona LattanziIgi MoonSimona ManciniIgnacio Abásolo AlessónSimona MitevaIgnacio Alejandro Mendoza MartínezSimona NicoaraIgnacio BartoloméSimona Roxana GeorgescuIgnacio Díez LópezSimona ScainiIgnacio LijarcioSimona ȘimonIgnacio Martínez-González-MoroSimona SpanuIgnacio MontorioSimona VillaniIgnacio Ramos-VidalSimona ZaamiIgnacio RefoyoSimone A. TomazIgnacio Torres AlemanSimone BayerIgnacy KitowskiSimone BelliIgnasi LabastidaSimone BirocchiIgnasi Navarro SoriaSimone Domenico AsprielloIgnazio La MantiaSimone DonatiIgor Alexandre Da Silva DiasSimone Garcia MacambiraIgor AshchepkovSimone GillIgor DumicSimone MarconciniIgor GallaySimone MorselliIgor GrabovacSimone ParriniIgor GruićSimone PiantiniIgor Iuco Castro Da SilvaSimone PorcuIgor NestrasilSimone R. De BruinIgor PazSimone RedaelliIgor PotočnikSimone RoddaIgor R. BlumSimone ScheithauerIgor RyabovSimone SehnemIgor SepelevsSimone TomazIgor VendraminSimonetta FascettiIgor Y. IskusnykhSimons SvirskisI-Hui WuSina HafiziIina SavolainenSina M. HulkkonenIkenna Desmond EbuenyiSina ShahabIker MadinabeitiaSinghal RashiIkram Ur RehmanSiniša OpićIkuya MiyamotoSinwan CheungIl Hyo JungSiofra HarringtonIl MoonSiqin WangIlan KatzSirak Robele GariIlan MerdlerSiri-Maria KampIlana HaliwaSirpa HeinävaaraIlaria BortoneSirpa KärkkäinenIlaria CampioniSisira EdirippuligeIlaria CataldoSisira S. WithanachchiIlaria ChiricoSisitha JayasingheIlaria Di MaggioSiteng ChenIlaria GuerrieroSi-Tong ChenIlaria LegaSiu Shing ManIlaria ManiniSiu-Ming ChoiIlaria MassaSiu-Ming ToIlaria PelusoSivakumar MuttucumaruIlaria PiacentiSivakumar NuvvulaIlaria PietriniSivaraj Mohana SundaramIlaria RiboldiSiwatt PongpiachanIlaria TinazziSiya HuangIlaria Tocco TussardiSiyang YuanIlda HoxhajSkucha-Nowak MałgorzataIlektra SpandagouSlaven JozićIlenia RuggiuSlawomir FranekIlenia SpadaroSławomir GawrońskiIlias DoulamisSlawomir JarekIlias KavourasSławomir KoziełIlias MavroidisSławomir PoskrobkoIlias MigdalisSławomir RębiszIlias N. LymperopoulosSławomir ŚlaskiIlias VlachosSlawomir TobisIlir ShehuSmadar SievIl­ma­ri Määt­tä­nenSmaoui SlimIlona KopytaSmarak BandyopadhyayIlona NordSmita NairIlona PokoraSmriti NepalIlya FedotovSneha B. RaoIlyes BenlalaSneha GautamIlyong JiSneha SinghIlyoung HongSnežana MilićevićIlze BogdanovicaSnježana Mardešić-BrakusIman MomkenSo Jung MunIman Salehi HikoueiSo SasakiIman TavassolySo Yeon YooImmacolata BelvisoSobhan MoosaviImmacolata OlivaSocorro Aida Borges YáñezImran KazmiSocrates BasbasImran Khan NiaziSoe MoeImti ChoonaraSofi FristedtIn Ok SimSofia Garcia-SanJuánIna Reić-ErcegovacSofia HadjileontiadouIna RissanenSofia Karina TrommlerováIndra OverlandSofia KoukouliIndrajit RaySofia MorgadoIndrani RoySofia P. Saenz AyalaIndre BakanieneSofia PugnaloniIndrikis KramsSofia RamalhoIndrit HoxhaSofia RavaraIndu B. AhluwaliaSofia SahabIneke VergeerSofia SantosInês BaíaSofia SousaInês Carvalho RelvaSofia WaissbluthInés Casado-VerdejoSofia WasterlainInes DrenjančevićSofya AptekarInes FasolinoSohail MushtaqInes KožuhSohei KobayashiInês PáduaSohyun ChoInes Panjkota KrbavčićSo-Hyun ParkInes TestoniSoi Hoi LamInes TomašekSokhna ThiamInes VillanoSokou RozertaInga Griskova-BulanovaSokou RozetaInga V. SlesarenkoSol García-GermánInge SchmitzSolange CostaIngeborg LundSolarski MaksymilianIngegerd JohanssonSolomon AdamsIngelise AndersenSomiranjan GhoshInger BenkelSon NghiemInger KjellbergSon Thanh NguyenInger Kristensson HallströmSoňa NevšímalováInger KullSong DingIngibjörg GunnarsdóttirSong Soo LimIngo BalderjahnSong WangIngrid AnderzénSongbo WangIngrid BelčákováSongee JungIngrid FalnogaSongwon SeoIngrid GillesSongyon ShinIngunn Holden BerghSongyoung ParkInho NamSonia Brito-CostaIñigo AramendiaSonia BuchholtzInigo SoterasSónia CarabineiroInmaculada Alvarez GallardoSónia Cardoso PintassilgoInmaculada Barranco-ChamorroSonia CastilloInmaculada Carmen Lara-PalomoSonia Chien-I ChenInmaculada Corral LiriaSonia García-SeguraInmaculada García-MartínezSonia GouveiaInmaculada Mateo-RodríguezSonia Hernández CorderoInmaculada Méndez MateoSonia J. Romero MartínezInmaculada Montoya-CastillaSonia LippkeInmaculada Romero GilSonia M. OliveiraInmaculada SalcedoSónia Matilde Fonseca MateusInmaculada Sureda-GarcíaSonia MediouniInmaculada Yustres AmoresSonia Monique BramantiInna Reddy EdaraSonia NathInnocent MusondaSonia PellissierIn-Seok SongSonia RobinsonInsil JangSonia Santoveña CasalInsin KimSónia SobralInsub ChoiSónia SousaInyong KimSonia Torres-SanchezIoakim SpyridopoulosSonia ValIoan I. ArdeleanSonja BidmonIoana - Daniela DulamaSonja ForwardIoana MoldovanSonja Novak-ZezulaIoana MozosSonja RossiIoana-Andreea SioustisSonu Menachem Maimonides BhaskarIoanna Anna PapachristouSonya NaganathanIoanna NtaikouSoo Ji KimIoannis BoletisSoo-Hwan ByunIoannis CharalampopoulosSoo-Hyun SungIoannis FarmakisSooim ShinIoannis GeorgakopoulosSook-Hyun ParkIoannis KoliarakisSoomi KimIoannis KosmasSoo-Min OkIoannis KostakisSoon-Chan KwonIoannis KyrgiosSoowoong NohIoannis LiampasSopan WaghIoannis PolitisSophia AnastasiouIoannis SpyroglouSophia BarinovaIoannis SyrmpasSophia BoudjemaIoannis TsakiridisSophia CharitouIolanda CioffiSophia S. LeggettIolanda De Fátima Lopes Calvo TibérioSophie CarterIonel BostanSophie CrevecoeurIonela Gouandjika-VasilacheSophie HallIonela HoteaSophie LelorainIonut LuchianSophie PoulterIoritz Sorzabal-BellidoSophie RutterIosif MarincuSophie TaylorIosif SifakakisSo-Ra BaekIpin ChangSoram OhIrantzu Recalde-EsnozSorana BolboacaIranzu Mugueta-AguinagaSorana BucurIrena Brčić KaračonjiSoraya Pacheco-da-CostaIrena GlažarSoraya Paz MontelongoIrena JindrichovskaSoraya Ruiz PeñalverIrena NalepaSøren ChristensenIrena TušerSören MöllerIrene A. AbelaSorin George TomaIrene AgeaSorin HostiucIrene AlbarránSorin MihaliIrene AlustizaSorin VâtcăIrene Carrillo MurciaSorina AurelianIrene ChecaSorina CernaianuIrene DuranczykSoshiro OgataIrene (Eirini) KamenidouSotiris AvgoustiIrene Garcia-MolinaSou Hyun JangIrene Helena Adriana AartmanSouhail HermassiIrene Jimenez-PerezSouleymane DoucoureIrene KamenidouSoumita GhoshIrene MargaritisSoumya MazumdarIrene Martín-RubioSoundappan S. V. SoundappanIrene OliveiraSousana PapadopoulouIrene P. CarvalhoSoyeon KimIrene Portilla-TamaritSoyoung KwakIrene SalmerónŠpela BogatajIrene Sanz-CorbalánŠpela ŠalamonIrene TilikidouSpencer D. LiIrene Torres-SánchezSpencer JamesIreneu MendesSpyridon KanellakisIreneusz GórowskiSpyridon PapageorgiouIrfan A. RatherSpyros NiavisIrfan RatherSree Lekshmi Sreekumaran NairIrida TsevreniSri Ram PentakotaIrimia Mollinedo-CardaldaSrikanth GattuIrina Adriana ChiurciuSrikanth KarnatiIrina BocharovaSrinath PashikantiIrina CazacuSrinivas DivakaruniIrina CherunovaStacey Allan ReadingIrina Eduardovna SokolovskayaStacey L. P. ScroggsIrina G. BelyaevaStacia RyderIrina KlizienėStacy ClemesIrina MakarovaStacy SimsIrina NovikovaStaffan JansonIrina SeverinStåle ØsthusIrineu De Brito JuniorStamatios GiannoukosIris FeinbergStamatios PapadakisIris UdasinStanescu Ana Maria AlexandraIrma TchokhonelidzeStanimir S. IvanovIrmina Ćwieląg-PiaseckaStanislav LazopuloIrmina RostekStanislav RojíkIrving RootmanStanislav RybtsovIrwan IskandarStanislav ShmelevIryna RachylaStanislava StoyanovaIsa OkajimaStanisław BielskiIsaac AmoahStanisław HorakIsaac CanoStanisław KaliszIsaac Levi HendersonStanisław MlekoIsaac López-LavalStanislawa Bazan-SochaIsaac Machorro-CanoStanley YuIsaac Oyeyemi OlayodeStarling EmeraldIsaac RampediStav ShapiraIsaac Selva RajStavros KalogiannidisIsaac TakaidzaStavros TriantafyllidisIsabel AbreuStavros TryfonIsabel AlmodóvarStavroula NtoaIsabel Antón-SolanasStavroula TsitsifliIsabel B. RodriguesStefan AltmannIsabel C. MetzStefan BleichIsabel CastilloStefan BratosinIsabel Corrales-GutiérrezStefan DreisiebnerIsabel Del ArcoStefan FraenzleIsabel EscobarStefan HarsanyiIsabel Escobio-PrietoStefan HertlingIsabel FernandesStefan Holland-CunzIsabel GálvezStefan Kurath-KollerIsabel GomezStefan M. FroschauerIsabel HenriquesStefan Mandić-RajčevićIsabel IguacelStefan MihaicutaIsabel LegazStefan MuthersIsabel López-VerdugoStefan NaydenovIsabel LucasStefan NetschIsabel María Fernández-MedinaStefan RaithIsabel María Gómez-TriguerosStefan RöderIsabel María López-MedinaStefan SammitoIsabel Martínez SánchezStefan SchumacherIsabel MercaderStefan SerbanIsabel MiguelStefan SimmIsabel MoralesStefan TyskiIsabel PiñeiroStefan WallnerIsabel Piñeiro AguínStefan WalterIsabel ReisStefan ZlatevIsabel Rodrigo MartínStefania BalzanIsabel Rodríguez CostaStefania BonafoniIsabel Salvat SalvatStefania CellaIsabel SospedraStefania ChiappiniIsabell KoinigStefania De MediciIsabella BonacciStefania Di GangiIsabella F. S. NgStefania FugazzaroIsabella GagliardiStefania GubbiottiIsabella SorecaStefania La GruttaIsabella TorcicolloStefania LanzaIsabelle Andersson HammarStefania LeuciIsabelle CabyStefania LiscoIsabelle Durand-ZaleskiStefania ManettiIsabelle Hininger-FavierStefania MassariIsabelle SkinnerStefania OrrùIsam BsisuStefania PaduanoIsamu OkadaStefania PetcuIsao OtsukaStefania RomanoIsdore Chola ShamputaStefania RomeoI-Shian (Ivan) SuenStefania ScuriI-Shiang TzengStefania ToselliIshmael Festus JajaStefania UcchedduIshtiaq AhmedStefania ZoiaIsidora VujcicStefanie GrataleIsidoro FasolinoStefanie Hessel-PrasIsidro A. PérezStefanie KampmeierIsidro Juan MironStefanie KleyIsidro Vitoria MiñanaStefanie L. RussellIslam HusainStefanie M. HelmerIslam RafiadStefanie MacheIsmaeel YunusaStefanie SamietzIsmael Fuente-MerinoStefano AmatoriIsmael MaatoukStefano Antonio GattoneIsmael Martínez-GuardadoStefano CanduraIsmael S. Sánchez-HerreraStefano CanosaIsmail Ercument AyazliStefano CianettiIsmini E. PapageorgiouStefano CirilloIsolde Martina BuschStefano CorbellaIsraa M. ShatwanStefano Da PozzoIsrael Caraballo VidalStefano DuglioIsrael Casado-HernándezStefano EramoIsrael Fisseha FeyissaStefano FerracutiIsrael IkoyiStefano FrancoIsrael Miguel AndrésStefano GobboIsrael Sánchez-CardonaStefano LongoIsrael Villarrasa-SapiñaStefano LuzzagoIssam TanoubiStefano MarianiIssei OgasawaraStefano MartinaIstvan LaszloStefano MassagliaIstván SzűcsStefano MinistriniIstván TarrósyStefano NobileI-Ta LeeStefano PaganoÍtala M. G. MarxStefano PalermiItalo AngelilloStefano PorruItalo MeloniStefano RizzaItandehui Castro QuezadaStefano RuggieriItziar IruarrizagaStefano SalataItziar Sobrino-GarciaStefano SalgarelloItzkovich YarivStefano SalvestriniIuga MadalinaStefano SilvestriIulia LupuStefano TasselliIuliana CosteaStefano TuriIuliana VijulieStefano ZiccardiIva StuchlikovaStefanos HatzilazarouIvan Cabrera I. FaustoStefanos LeontopoulosIván CastellvíSteffano LoppiIvan ChenchevSteffen GrautoffIvan Cruz-AcevesSteffi KreuzfeldIvan Domicio Da Silva SouzaSteffi Riedel-HellerIvan Gláucio Paulino-LimaŠtefica MikšićIvan HenricoSteinn SteingrimssonIván Herrera-PecoStelios XanthosIvan Julian-RochinaStella LopezIvan KozitsinStella QuahIvan KralovStella SekulićIvan L. HandSten DreborgIvan LaiSten HellströmIván López PardoStepan KavanIvan Luzardo-OcampoŠtěpán KavanIvan MiskulinSteph HemmingsIvan PaunovićStephan BischofIvan PiresStephan HeisingerIvan S. RaginovStephan Van Der ZwaardIván Santolalla-ArnedoStéphane P. AhernIvan ŠklebarStephanie A. BorgIvan ŠošaStephanie BaudIvan UherStephanie C. MelkonianIvana Božić MiljkovićStephanie ChappelIvana CroghanStephanie CraigIvana HromatkoStephanie DauthIvana JaricStephanie DeFlorio-BarkerIvana KarmelićStephanie F. LingIvana KholováStephanie FletcherIvana Lessner ListiakovaStephanie Howard WilsherIvana MilovanovićStephanie J. TuminelloIvana Nižetić KosovićStephanie K. NishiIvana PeranStephanie KershawIvana Pikić JugovićStephanie KoernerIvana PodhorskaStephanie M. WalshIvana RumoraStephanie MailletIvana ŠkrlecStephanie PadbergIvana SutejStéphanie SaxerIvana TasicStephanie ScottIveta CimbolákováStephanie SteelsIveta UbrežiováStephanie StielIvo FoppaStephanie V. WrottesleyIvo PonocnyStephanie VallianatosIvo RegliStephen A. ContagIwao UeharaStephen BokIwona Bodys-CupakStephen C. Kolwicz Jr.Iwona Chmura-RutkowskaStephen CainIwona Czaplicka-KozłowskaStephen ContagIwona Gorzeń-MitkaStephen David MyersIwona GrobelnaStephen DuckettIwona Kantor-PietragaStephen EdwardsIwona KrzywnickaStephen FafulasIwona MalickaStephen IvesIwona Olszewska-CzyżStephen James HillsIwona Piatkowska-ChmielStephen John HoughtonIwona PielesiakStephen JosephIwona PomianekStephen KwokIwona SidorkiewiczStephen M. BlackIwona SikorskaStephen M. PopkinIwona StaniecStephen McCordIwona SulowskaStephen RatchfordIwona TraczykStephen SchwabIwona WertelStephen Siu-Yu LauIwona Wrześniewska-WalStephen StansfeldIzabela FeckaStephen TsujiIzabela KrupińskaStephen WintersIzabela LipińskaStephen WiryaIzabela Luiza Ciesielska-WróbelStergios BoussiosIzabela Małysz - CymborskaStessa Tzu-Yuan ChaoIzabela Michalska-DudekSteve BeermanIzabela RaganSteve BrowningIzabela SztangretSteve GarnerIzabela Szymczak-PajorSteve GoodmanIzabela ZakrockaSteve HyerIzabella LeckaSteve NolanIzabella UchmanowiczSteve O’HernIzhak SchnellSteve RatchfordIzhar Hyder QaziSteve ShnyderIzolde BouloukakiSteve SimpsonJ. A. P. M. De LaatSteve Simpson-YapJ. A. RathmacherSteve TaylorJ. Alberto Montoya-AlonsoSteven C. BourassaJ. Barton (Bart) CunninghamSteven ComptonJ. Calvin KouokamSteven FeldmanJ. Craig PhillipsSteven FreemanJ. De MouzonSteven HabermanJ. Dominic SmithSteven J. CassadyJ. E. Jamal MartínSteven LawyerJ. Grant McFadyenSteven LucasJ. J. M. Van DeldenSteven M. HeatonJ. K. GarrettSteven MarkowitzJ. Robin MoonSteven Paul NisticòJaana SorvariSteven PudneyJaap GlerumSteven RoodenrysJaap VeerkampSteven Ross MurrayJaap W. DeckersSteven SussmanJacek BajSteven ThygersonJacek BarcikSteven WallerJacek BoguckiSteven WuJacek BucznyStevie-Jae HepburnJacek C. SzepietowskiStewart B. KirtonJacek ChmielewskiStöcker Phil. NicolaJacek E. NyczStormi Pulver WhiteJacek JagodzińskiStuart GilmourJacek KlichStuart J. H. BiddleJacek KubicaStuart LeskeJacek LeszczyńskiStuart McLennanJacek LewandowiczStuti KokkaleraJacek MichalskiStyliani VlachouJacek MoskalewiczStylianos E. TrevlakisJacek NyczSu Hyun KimJacek OskarbskiSubhabrata MoitraJacek PietruchaSubhashis DasJacek PolechońskiSubhasish SahaJacek PyżalskiSubrata DebJacek SmerekaSudhanshu AbhishekJacek SzmaglinskiSudhir K. ThakurJacek SzymańskiSudhir KotnalaJacek ToporskiSudip BanerjeeJacek WąsowskiSudip MondalJacinda NicklasSudipta BiswasJack A. HolmanSue BuckleyJack CannonSue DevineJackie WrightSue K. ParkJackie YenerallSue KilpatrickJackline Freitas Brilhante De Sao JoseSue LukersmithJaclyn BunchSue NangJacob CaldwellSue SpaidJacob GrazerSue V. RosserJacob GroshekSuellen Da Rocha MendesJacob JordaanSuhad SbeihJacobo Rodríguez-SanzSuhail Najem AbedJacopo A. VitaleSuhas Sampat KharatJacopo PizzicannellaSuhee KimJacqueline E. ReznikSuh-Hee ChoiJacqueline KentSu-Hyun ShimJacqueline MiddletonSuin ParkJacqueline StephensSuixin LiuJacqueline W. CurtisSuiyao ChenJacques HimpensSujan GautamJacques Van LankveldSujata SapkotaJade BergSujay RayJade KettlewellSujeong KimJade Lai King WongSujin ShinJadwiga AmbroszkiewiczSujita Kumar KarJadwiga KubicaSujita NarayanJadwiga ManiewskaSukwon KimJadwiga MazurSulaiman LakohJadwiga Wójkowska-MachSuleman LazarusJae Chul KohSu-Lien LuJae Ho LeeSulimon SattariJae I. K. ShinSulistyawati SulistyawatiJae Lan ShimSumali PandeyJae Yong KimSuman DuttaJae Young LeeSumana ChintalapudiJaehee LeeSumio AkifusaJaeho ChoSumit K. ShahJae-Hong KimSumit SiddharthJaehoon SeolSumita GhoshJae-Hyeong ParkSumitaka KobayashiJaehyuck LeeSun Young AhnJaehyun ParkSun-Ae KimJae-Hyung LeeSundar RamalingamJaejong ParkSunday Adewale OlaleyeJaelim ChoSunday OnagbiyeJaemin JeongSuneel KumarJaeryong YooSunetra SaseJae-Seok KimSung Soo LimJaeson CallaSunga KongJae-Sung YooSung-Cheol KohJae-Won LeeSungchul SeoJaewook JeongSunghae JunJaeyoung KimSunghee KimJaeyoung LeeSunghyup Sean HyunJae-Young LeeSungil HanJaffar AbbasSungkyu Shaun ParkJagadish K. ChhetriSungmin LeeJagdish KhubchandaniSungok ChangJaggi UjjaldeepSungsig BangJagoda CrawfordSungsu YoukJagoda PlaczkiewiczSung-Ta LiuJa-Hoon KooSungwon ChungJai P. RaiSungwon LeeJaime Andrés BayonaSung-Woo KimJaime Aparecido CurySungyong ChoiJaime Bonnín RocaSunhee ParkJaime BoschSunitha KodidelaJaime Bustos-MartínezSunitha ZechariahJaime Cantallops RamónSun-Joo JangJaime DevineSunjoo ParkJaime EspinoSun-Ju KimJaime MadriganoSun-Kyug KangJaime OrtegonSunkyung ChoiJaime Pinilla-DominguezSun-Young KimJaime Roset CalzadaSun-Young RiehJaime Salom MorenoSupriyo RayJaime Vallés-GiménezSurendra RajpurohitJaime Vásquez-GómezSuresh KalathilJaimee MallionSuresh NarayanasamyJain PriteshSurita Du PreezJairo León-QuismondoSurya KantJairo Ortiz RevillaSurya Prakasarao KovvasuJairo Rodríguez-MedinaSusan AudinoJairo VanegasSusan Beth RifkinJaishree RamanSusan BuchananJake CampSusan CorkJakob RehrlSusan GoldsteinJakob ThannesbergerSusan GranthamJakob Werner HansenSusan HelmJakub CiazelaSusan J. JenkinsJakub DolezelSusan JansonJakub DuszczykSusan M. TarloJakub GąsiorSusan Madison-AntenucciJakub KortasSusan MagasiJakub KrejčíSusan McClementJakub M. GacSusan MirlohiJakub Maksym GrabowskiSusan P. JacupsJakub RajčániSusan P. KempJakub RybkaSusan RyanJakub SlupskiSusan SlovinJakub SoviarSusan Snow WadleyJakub StempienSusan StraussJakub SuchanekSusan TawiaJakub SwachaSusan WoskieJakub TaradajSusan XuJam KhojastehSusana Adelina Moreira Carvalho BastosJamal NaderiSusana B. BravoJames A. RobertsSusana CoimbraJames BrasicSusana DuarteJames BretzkeSusana González PérezJames C. OlesonSusana HernándezJames C. SimeonSusana Isabel Justo-HenriquesJames ConeSusana Margarida De Freitas FerreiraJames D. KeanSusana MendesJames DugdaleSusana MourãoJames G. RavinSusana PaixãoJames G. RiceSusana PasamarJames Gerard CaillierSusana RemesarJames HayesSusana RubioJames JohnstonSusana Rubio-ValdehitaJames K. C. ChenSusana SilvaJames K. RussellSusanna ChuiJames Kevin SummersSusanna GeidneJames KimballSusanna KugelbergJames Kumi-DiakaSusanna ManciniJames LeighSusanna NordinJames LettsSusanna RaisamoJames M. NaessensSusanna Salminen-PaateroJames MichaelSusanna SciomerJames MilnerSusanne AndermoJames Nicholas BaraniukSusanne AnderssonJames O’Higgins NormanSusanne ElsenJames Omondi OutaSusanne FrennertJames P. GavinSusanne Grylka-BaeschlinJames ParkerSusanne KobelJames Patrick BeirneSusanne RospleszczJames PethickSushil Kumar SinghJames PowersSusie SykesJames R. S. HockerSusie YoonJames RombiSusumu AbeJames StameySusumu ItoJames ThompsonSutanuka BhattacharjyaJames V. CoeSuzan AnwarJames W. SonneSuzana BrownJamie FerrillSuzana OtaševićJamie SmithSuzanne Adele TraskJamil HussainSuzanne BartingtonJamioł-Milc DominikaSuzanne D. ThomasJamison ConleySuzanne DallmanJan Adriaan GrawSuzanne F. JacksonJan BilskiSuzanne FitzsimmonsJan BlecharzSuzanne L. CrossJan BulecaSuzanne LinnaneJan D. ReinhardtSuzanne O’NealJan De JongeSuzanne O’NeillJan DettmersSuzanne R. KunkelJan DomaradzkiSuzanne R. SteinbaumJan DouglassSuzanne RogersJan E. AzarovSuzanne SuggsJan Fekke YbemaSuzanne VangJan FričSuzette SutherlandJan G. C. Van AmsterdamSuzuki SayakaJan HaukeSuzy Mascaro WalterJan HenzelSuzy NgomoJan HladkýSuzy WeemsJan HoflandSveinung Wergeland SørbyeJan HolešovskýSven BrembergJan Jaap H. M. ErwichSven FischerJan Jens KoltermannSven NiklanderJan K. WachterSven NolteJan KabátekSven StrickrothJan KazakSven Van LaereJan KoltermannSven-Arne SilfverdalJan KonarskiSvenja SpringerJan KotekSvetlana CherlinJan KucharskiSvetlana DrobyazkoJan KühnischSvetlana EdenJan Luxhøj StøvringSvetlana PushkarJan MarcussonSvetlana SaikovaJan MejzlikSvetlana V. MustafinaJan MuzikSwagat SharmaJan Paul Van WingerdenSwapnil BawageJan PetriczkoSwarup MitraJan PithaSwastik PhuleraJan PolcynSwee KuikJan SaevelsSweilem B. Al RihaniJan StejskalSwinburne A. J. AugustineJan StępniakSyed Amir AshrafJan StyczyńskiSyed Haris OmarJan UlfbergSyed Husain Mustafa RizviJan WitowskiSyed Imran Ali MeerzaJana AsherSyed-Abdul ShabbirJana BabjakováSygkliti-Henrietta PelidouJana GeroldSylvia KirchengastJana HeczkovaSylvia MignonJana KostalovaSylvia TerbeckJana MosseySylvia TitzeJana Říhová AmbrožováSylwester CiećwieżJanaina Antonino PintoSylwester SommerJanaka JayawickramaSylwester WereskiJanaki DeepakSylwia AndruszczakJanaki IyerSylwia BudzynskaJanani IyerSylwia ChojnowskaJanani VaradarajanSylwia JaskulskaJane Banaszak-HollSylwia KałuckaJane GaultneySylwia Kuczamer-KłopotowskaJane HarrisSylwia MałgorzewiczJane KuepferSylwia Pieńkowska-KamienieckaJane M. GannonSylwia PłaczkowskaJane M. WilliamsSylwia SiebielecJane March-McDonaldSylwia Zakowska-BiemansJane PageSyuichi ItahashiJane SimpsonSzabo Dan-AlexandruJane SimsSze Y. LiuJane VennikSzymon HoffmanJane WaldfogelSzymon Łukasz DraganJanean Dilworth-BartSzymon SkoczyńskiJanelle WagnildSzymon StereńczakJanet FranklinSzymon SzewrańskiJanet Henshall MomsenSzymon TomczakJanet KellySzymon WiśniewskiJanet M. Y. CheungT. J. MooreJanet MattssonT. R. I. CataldiJanet MichelloT. S. (Tjip) Van Der WerfJan-Hinrich MeyerT. Y. ChenJani IngramTabito KinoJani LaineTadao OokaJanice HegewaldTadas ŽvirblisJanicke Liaaen JensenTadashi ItoJanie CorleyTadashi YagiJanine Anne CampbellTadej PetreskiJanine CampbellTadeusz A. GrzeszczykJanine De ZeeuwTadeusz AmbrozyJanine GronewoldTadeusz MagieraJanine WhiteTadeusz MalewskiJanine WichmannTadeusz MoskalikJanira RoanaTadeusz PrzybyłowskiJanja TrčekTadeusz SawikJanna DickensonTadeusz SiwekJanna G. KoppeTadeusz SiwiecJanne Brammer DamsgaardTadgh McMahonJanne PetersenTae Im KimJanneke ElberseTaehoon KimJannell BazurtoTae-Hwan KimJanni AragonTaehwan ParkJannick NiortTae-Jin ChoiJannine BaileyTae-Ju ChoJan-Olof KarlssonTae-Lim YoonJanos G. PitterTaesic LeeJanos JuhaszTaesu CheongJanos M. KanczlerTaewoo NamJános SándorTaeyoung KimJanusz BohatkiewiczTafadzwa DzinamariraJanusz ĆwiklakTafadzwa NyanhandaJanusz KrzyścinTafadzwanashe MabhaudhiJanusz KsiazykTahereh MaghsoudiJanusz KwiecieńTahir MahmoodJanusz MajewskiTaichi ShimazuJanusz RakTaichi TenkumoJanusz RusekTai-Chia ChiuJanusz RymaniakTaihei ItoJanvier GasanaTai-Heng ChenJaqueline C. AvilaTaiji NoguchiJaqueline ScholzTaiki MoriJaques OosthuizenTai-Li ChenJared CarlbergTai-Long PanJared SkinnerTaimoor Hassan FarooqJari E. KarppinenTainá BittencourtJarim KimTainah P. LimaJarle AarstadTaisen IguchiJarle Løwe SørensenTaishi KayanoJarmila ČechmánkováTaisun HyunJarmila CelakovskaTajana PavicJarmin Christine YehTakaaki WajimaJaromír AstlTakafumi AbeJaromír MyslivečekTakafumi AndoJaromír ŠimonekTakaharu GotoJaroslav A. HubacekTakahiko KoyamaJaroslav DvorakTakahiro TsugeJaroslav KacetlTakahiro WadaJaroslav ŘíčanTakako YasudaJaroslava KopcakovaTakalani Grace TshitanganoJaroslava KubátováTakamasa MizunoJarosław HerbertTakamichi MorikawaJarosław Jaszczur-NowickiTakao OhtsukaJaroslaw KozubaTakashi AsakuraJarosław MalaraTakashi AzumaJarosław PasekTakashi HashimotoJarosław PinkasTakashi HiraiJaroslaw S. KardasTakashi IkegamiJarosław UglisTakashi MizushimaJaskaran Singh SethiTakashi NaruseJasmin HänelTakashi SasakiJasmina Casals TerreTakaya HigakiJasmina ZivanovicTakayuki IkedaJasmine CheungTakayuki KoikeJasna JablanTakayuki NittaJason AndersonTakehiro IwatsukiJason BrumittTakehiro MichikawaJason D. RiveraTakehiro SugiyamaJason Devoy-KeeganTakeji SaitohJason J. RamirezTakenori HarunaJason Jiunshiou LeeTakeo MukaiJason KeelerTakeshi EbaraJason MagnusonTakeshi HashimotoJason MillsTakeshi KamiyaJason ParksTakeshi MiyamaJason ReeceTakeshi NinchojiJason RotoliTakeshi YamaguchiJason S. RadowskyTakeshi YodaJason T. HarrisTaketo OkadaJason T. TzenTakshashila TripathiJason W. MarionTakuichi SatoJason WashburnTakuji AdachiJason WilsonTakuma InagawaJasper QuakTakuro TobinaJasper Van AsscheTakuya OmuraJasper VerheulTakuya UrakamiJaume VivesTali SinaiJavad AlizargarTalia Randa EsnardJavier Almela-BaezaTalkmore MarutaJavier Bustos DíazTam DinhJavier Cifuentes-FauraTamar BermanJavier Cortes-RamirezTamar KinaciyanJavier Díez-PalomarTamara BasishviliJavier E. Contreras-ReyesTamara Hew-ButlerJavier EcheverríaTamara Nunes Lima-CamaraJavier Eloy Martínez GuiraoTamara Pawlaczyk-KamienskaJavier F. BoyasTamara PecherinaJavier Fernández-SánchezTamara ReinickeJavier Ferrer TorregrosaTamara Rial RebullidoJavier Flores-FraileTamara TseJavier Frontiñán-RubioTamara WrightJavier Gamez-PayaTamás BerkiJavier HerruzoTamás KovalcsikJavier LopezTamerah HuntJavier Marco-MartínezTamis W. PinJavier Merino-AndresTamlin S. ConnerJavier Moreno-LázaroTammie KaufmanJavier Ortuño SierraTammie RonenJavier Perez-GarciaTammy AplinJavier PucheTammy KiserJavier Ramos-OrtegaTammy LiuJavier Rico-DíazTamotsu HoshinoJavier Rodrigo-IlarriTamus Zoltán ÁdámJavier Rodríguez VillanuevaTan Leng GohJavier Ruiz RamírezTan Pui YeeJavier SantabarbaraTan YigitcanlarJavier Sierra SánchezTanara Vieira SousaJavier SilvestreTancu Ana MariaJavier TarangoTancu Ana Maria CristinaJavier VelázquezTaner SarJaw-Shiun TsaiTang-Chuan WangJaw-Yuan WangTânia Araújo VielJay FriedenbergTânia Cristina De Oliveira ValenteJay MichaelsTânia LucciJay N. PatelTania ManoJay S. AlbaneseTânia SantosJaya AseervathamTânia VidalJaya JhaTanja BukovčanJayakrishnan AjayakumarTanja Grubić KezeleJayarama GunajeTanja HarrisonJayasree (Joy) BasuTanja J. MiličićJayce FarmerTanja OosthuyseJayden HunterTanmoy BhowmikJayme WaltersTanneke HerklotsJaymie MelikerTanuj WadhiJayson StoffmanTanushree DangiJayson V. PagaduanTanveer AhmadJayun HyunTanvir KhanJe Hyeok OhTanya SerisierJean A. RondalTanzil Mahmud ArefinJean ButelTao DengJean Christophe DelpechTao HuangJean Christophe GigerTao LinJean Claude De MauroyTao LongJean CouballyTao LyuJean Croce HemphillTao WangJean Ezequiel LimongiTao YangJean LimongiTao-Hsin TungJean Louis CairoliTaona HaderleinJean Luc BaillyTapan BhattacharyyaJean MukherjeeTar Choon AwJean PerriotTar IldikóJean WooTara Clinton-MchargJean Yves HeurtebiseTara M. StruttJean-Baptist Du PrelTara RamaniJean-Claude ThillTara Rava ZolnikovJeanette A. LawrenceTarannom ParhizkarJeanette M. AndradeTarek El-BialyJeanette ReyesTareq AssafJean-François BlaisTareq RasulJean-François ChenotTariq AbdulKarim AlalwanJean-Francois DaoustTarmo Johannes ValkonenJean-Francois VuillaumeTaro MatsukiJean-Gabriel ContaminTaro UraseJeanine AmmannTaroh SatohJeanine M. BuchanichTaru S. DuttJean-Jacques MonsuezTarun GhawanaJean-Luc DavideauTasadduq ImamJean-Marie ExbrayatTashara LeakJean-Michel CostesTasuku OkuiJeanne B. PercivalTatiana BegottiJeanne CartierTatiana ChristidesJean-Paul CalbimonteTatiana De Oliveira SatoJean-Paul RichaletTatiana Kondrat’evaJean-Philippe BerteauTatiana SidiropoulouJean-Philippe DroletTatiana StaritzkyJean-Pierre JesselTatiana TchemisovaJean-Pierre MichelTatiana Viktorovna KochetovaJean-Thomas BernardTatiana Y. CherkashinaJean-Yves MaillardTatiane PiuccoJeconiah Louis DreisbachTatjana FischerJed BoardmanTatjana PõlajevaJedrzej BylkaTatjana TrivicJee Hye WeeTatjana Vilibić-ČavlekJee Hyun KimTatsuki IkomaJee Soo ParkTatsuya FujikawaJee Taek KimTatsuya YamamotoJee Young KimTatsuya YoshiharaJee-Hye LeeTatyana KhudyakovaJef Van Den EndeTatyana KrupnovaJeferson SantanaTaxiarchis KatsinelosJeff BroomeTaylor Chastain GriffinJeff C. W. WangTa-Yuan ChangJeff CamkinTeatske AltenburgJeff CanarTe-Chih WongJeff GibbonsTeck-Boon TanJeff HoughtonTed McdonaldJeff TaylorTeddy Weifa YangJefferson AraujoTe-Feng YehJeffery D. LongTefera TadesseJeffrey A. BakalTegegne Tesfaye HaileJeffrey A. KeenanTehila KalagyJeffrey BaraTehila RefaeliJeffrey GambleTehmina KhanJeffrey HowardTeija KangasJeffrey HubbardTeija WaaramaaJeffrey Hugh GambleTeikichi IkuraJeffrey J. HuangTejinder Pal SinghJeffrey J. ParrTeka KhanJeffrey PengTella LanttaJeffrey R. AndolinaTelma AlmeidaJeffrey RewleyTelmo PereiraJeffrey RothschildTelung PanJeffrey S. ForsseTemitope O. SogbanmuJeffrey S. HallamTena NiseteoJeffrey S. OwenTeneasha WashingtonJeffrey WangTeng Kai OngJekaterina SchneiderTengfei LiJelena BobkinaTennley VikJelena DjokićTeodora HoinoiuJelena KovacevicTeodoro GeorgiadisJelena LoborecTepetes KonstantinosJelena MileševićTeppei TakeshimaJelena MusulinTerence A. ImberyJelena T. PetrovićTerence MoriartyJelena VranješevićTerence OngJelica Grujić-MilanovićTeresa Filomena Vieira NunesJeng-Tzong ShiauTeresa GavaruzziJen-Hao ChiTeresa Gonzalez ArteagaJenil PatelTeresa GrzelakJenna PalladinoTeresa LeãoJenni BlomgrenTeresa LeszczyńskaJennie LouiseTeresa MadureiraJennifer BlankenshipTeresa Makowiec-DąbrowskaJennifer BrayTeresa MateusJennifer BrowneTeresa MonteJennifer BunnTeresa MorlaJennifer ChippsTeresa PaivaJennifer ChopraTeresa PaolucciJennifer ColeTeresa Pérez-CanoJennifer CreeseTeresa Pozo-RicoJennifer D. RobertsTeresa PuvimanasingheJennifer DanielsTeresa RitoJennifer FirstTeresa Rubio-TomásJennifer FishTeresa SalvatoreJennifer GatzemeierTeresa Sanchez-GutierrezJennifer HockenberyTeresa Silveira LopesJennifer HoggTeresa Sławińska-OchlaJennifer J. BarbTeresa TorregrosaJennifer Jill HarmanTeresa VeraJennifer JonesTeresa WagnerJennifer L. HartwellTeresa ZwierkoJennifer L. P. ProtudjerTeresia MbogoriJennifer L. SchneiderTeresina MancusoJennifer L. WarnickTereze KatzensteinJennifer LingeTerézia RončákováJennifer LiraTeriitutea QuesnotJennifer M. GrossmanTerje DalenJennifer M. Jabson TreeTerrence J. RavineJennifer PascaliTerri DownerJennifer R. SchroederTerri PikoraJennifer RunkleTerry A. CronanJennifer SalinasTerry A. KleinJennifer SassTerry GordonJennifer SchneiderTerry HanleyJennifer T. GrierTerry J. FroggattJennifer XieTerry WotherspoonJennifer ZinkTerry/Ruoh-Nan YanJennifer ZuercherTeruhisa KomatsuJenny C. A. ReadTerumitsu HirataJenny Cubells SerraTerun DesaiJenny DrottTeruyoshi AmagaiJenny HessonTesfahun EshetieJenny MaTess CapperJenny MercerTessa CoppJenny PaananenTessa R. EnglundJenny PakTessho MaruyamaJenny WilkinsonTetsuo KidaJenq-Shyong ChanTetsuo NomiyamaJens André HammerlTetsuro KobayashiJens C. RichterTetsuya HiraishiJens HolstTetsuya ShiuchiJens PrzybillaTetsuya YumotoJentre OlsenThaíse Gonçalves AraújoJen-Yang LinThangarajah AkilanJeong Hwan LeeThangiah GeethaJeong-Hoon LimThankam S. SunilJeong-Hyun KangThanutchaporn KumrungseeJeong-Hyung ChoTharshanah ThayabaranathanJeongsoo YuThayne KowalskiJeongtae KimThayse Natacha GomesJeong‐Won HanTheano SamaraJeongwoo LeeThemistoklis TsatalasJeon-Young KangTheo ArentzeJeramy L. R. BaumTheocharis G. KonstantinidisJérémie PourchezTheodora Kunovac KallakJeremie RichardTheodora PapamitsouJérémie RiouTheodore AndronikosJeremy AireyTheodore H. WeinJérémy CoquartTheodore KotsilierisJeremy Di DomizioTheodore S. LentzJeremy HessTheodoros EleftheriadisJeremy K. WardTheodosia AdomJeremy MattsonTheodosios PalaskasJeremy MennisTheolis Barbosa BessaJeremy P. KohlitzTheresa LetoJeremy TameTheresa O’KeefeJer-Hao ChangTherese HellmanJerilynn C. PriorTherese McDonnellJer-Junn LuhThereza Maria Magalhães MoreiraJer-Ming ChangThersilla OberbarnscheidtJernej RoskerThessa HilgenkampJérôme A. J. BeckerTheyazn H. H. AldhyaniJérôme GippetThiago Antonini AlvesJérôme LaurinThiago Pavoni Gomes ChagasJerome NriaguThiago PoletoJerónimo Aragón VelaThiago VieiraJerónimo GonzálezThibaud GarcinJerónimo VidaThibaut ChapronJerre Mae TamanalThibaut DelsinneJerrie HsiehThien-An LeJerris HedgesThierry Oscar EdohJerry KoloThiha Tin KyawJerry PullanThi-Kim-Quyen VoJerzy B. GajewskiThiloththama Hiranya Kumari NawarathnaJerzy CzmochowskiThom BaguleyJerzy GębskiThomas A. BurkeJerzy GrobelnyThomas A. De PreeJerzy KaźmierczykThomas AkintayoJerzy LisekThomas ArcuryJerzy RomaszkoThomas ArmstrongJerzy RosłonThomas BaerJerzy SowaThomas BlahaJerzy WalochaThomas BöhlerJerzy Wojciech MietelskiThomas BrendlerJesper Bo NielsenThomas CampagnaroJess LuceroThomas CraemerJesse BermanThomas Davidson Jesse CaputoThomas E. EdesJesse MathesonThomas ElliotJesse PlascakThomas FischerJesse Swann-QuinnThomas FreiJessica A. WilliamsThomas G. PoderJessica Allia WilliamsThomas George CampbellJessica Ann DiehlThomas GreenfieldJessica BahorskiThomas GrischekJessica BallaThomas Hegyi HegyiJessica BurketThomas HeinzeJessica BurraiThomas HollandJessica CastagnaThomas J. GlynnJessica CastnerThomas JamiesonJessica CastonguayThomas Jürgen KlotzbierJessica Fernández-AgüeraThomas KirchnerJessica García-GonzálezThomas KleinsorgeJessica GleasonThomas M. HinckleyJessica HuaThomas M. KaiserJessica Ibarra MoraThomas M. TiefenboeckJessica J. O’KonekThomas MeashamJessica Jones-SmithThomas MonaghanJessica L. ElfThomas N. MaloneyJessica L. KingThomas NormanJessica L. MalischThomas P. McCoyJessica L. PotterThomas PlümperJessica LangThomas R. WoodJessica ManousakisThomas RigottiJessica Maria ChiccoThomas SallerJessica Ortega BarónThomas SantariusJessica PiaseckiThomas ScheidstegerJessica PurswaniThomas SchmidtJessica RayThomas Schmitz-RixenJessica RendleThomas Scot MurrayJessica RiggsThomas SkovgaardJessica SewellThomas StewartJessica SoldaviniThomas T. H. WanJessica StreitThomas TenkateJessika C. BolesThomas ThiebaultJesuk KoThomas ThurnerJesús Adrián LópezThomas Viskum Gjelstrup BredahlJesús Alejandro Prieto-AmparánThomas VolkenJesus Barrena-MartinezThomas WanJesus Canora LebratoThomas WenzelJesús DevesaThomas WittlingerJesus Enrique GarcíaThomas WolfJesús Funuyet-SalasThor Willy Ruud HansenJesús G. ZorrillaThórarinn SveinssonJesús García RubianoThorsten MühlhausenJesus Gonzalez-RubioThorsten RudroffJesus IbarluzeaThuane RozaJesús Jiménez-LópezThulasee JoseJesús Jiménez-RuizThuy LaJesús López BedoyaTiago AtalaiaJesús López BelmonteTiago Fonseca Albuquerque Cavalcanti SigahiJesús María AlvaradoTiago GonçalvesJesús María López-ArrietaTiago LimaJesús MartínezTiago OliveiraJesús Martínez-MartinezTiago Pinto RibeiroJesús Mena-ÁlvarezTiago Severo PeixeJesús Miranda PaezTian RenmaoJesús Molina-MulaTian Shyug LeeJesus Montero-MarinTianan YangJesús Montoya-MendozaTianhu LiJesús NavasTianjia GuanJesús Oliva-Pascual-VacaTianjun LuJesús PastorTianren YangJesus PelaezTianshuang GaoJesus Perez GilTianye ZhaiJesus Prieto-LloretTianying WuJesús R. HuertasTibebeselassie Seyoum KeflieJesús Ramón-LlinTiberiu-Paul NeaguJesús Saiz GaldósTiberius DicuJesús Sanz FernándezTibor ErtlJesus SecoTibor GuzsvineczJesus Seco-CalvoTibor HortobagyiJesús Siquier CollTibor KovácsJesús TresguerresTiehang WuJesus Vicente GiménezTien M. NguyenJesús-Nicasio García-SánchezTiffani HowellJetty Duffy-MatznerTiffany GrahamJi Bo WenTiffany J. Ríos-FullerJi Hyun OhTiffany JonesJi JiayuanTiffany LavisJia Ping WuTiffany M. Powell-WileyJia XueTiffany SanchezJiafen HuTigestu Alemu DesseJia-Feng ChangTihomir OrehovačkiJia-Hai LuTiija RintaJia-Hong KuoTiina M. MattilaJiajia SongTiina SavikangasJiale YangTilahun AyanouJialei DuanTilak DuttaJiali YinTill DammaschkeJialiang LiTilman ReineltJialin ZhangTilo NiemannJiaming LiuTim Alex LindskouJian HuangTim HeemsothJian LiuTim HulsenJian ZhangTim JodaJian ZhouTim M. SzewczykJianchun SunTim MarjoribanksJianfeng SunTim McMurryJiangang ChenTim PrangemeierJianghua LiuTim RahmelJiangliang JinTim SandleJiangtao DuTim TaberJiangxin GuTimmy FrawleyJiangyuan WangTimmy LiJianhui BaiTimo H. VesikariJianjun ChenTimo Homeier-BachmannJianjun WenTimoth MkilimaJiann-Chu ChenTimothée BaudequinJianping LinTimothy B. DaviesJianping MaTimothy BartrandJianwei ChengTimothy BungumJianxin LiTimothy CiesielskiJianxiong WangTimothy E. FordJianye LiuTimothy E. O’TooleJianyong WuTimothy EricksonJianyue JiTimothy FogartyJianzhao BiTimothy G. FordJianzhi LinTimothy GoodaleJiao Jiao LiTimothy HsuJiaqing WangTimothy L. HubbardJia-Ruey ChangTimothy LeeJiashun HuangTimothy LutzJiawei ChenTimothy M. BarzykJiaxi HuTimothy M. EschleJiaxu ZengTimothy MatthewsJiayan HuangTimothy O. AdekoyaJiayi JiTimothy SchmutteJiayong ZhangTimothy SlyJiazhu PanTimothy TseJidong SungTimothy W. FarrellJie (Jackie) ZhuangTimur SellmannJie LiTin LukićJie LiuTina K. JensenJie MaTina KubrakJie ShangTina LindhardJie SunTina ParadzikJieun KimTina Tičinović KurirJigar ModiTina TomazicJihad AlabdullahTina ŽagarJihong ParkTinakon WongpakaranJi-Hwan LeeTinashe ChabikwaJi-Hye KimTinashe ChuchuJihye LeeTindara CaprìJijia WangTine K. GrimholtJijun HuangTing Kin NgJikun WangTing XiaJill A. JenkinsTing ZhangJill MurphyTing-Hsun LanJillian LauerTingting JuJillian MillerTingting ZhangJillian TullisTinne DillesJillian Vinall MillerTino BucakJim ChamberlainTino PrellJim WarrenTirupapuliyur DamodaranJimena MuciñoTiscar GraellsJimmy GonzalezTito PizarroJimmy O’GormanTiwari AnujJin HeTizian RosenstockJin LiuTiziana CappelloJin Su JeongTiziana CiarambinoJin Sung RhaTiziana FilardiJin WeiTiziana GuzzoJin Yong JeonTiziano TestoriJina ChooTjhin WigunaJina OhTobias BerglerJinae LeeTobias LipekJinbao GuTobias PolzerJinbo HeTobias RomeykeJincai MaTobias RufJin-Ding LinTobias SchrippJing LiuToby J. EllmersJing LuToby M. HallJing ShiToby MündelJing WangTodd G. MorrisonJing WuTodd James AllenJing YuanTodd SchenkJing ZhangToh Yen PangJingchen ZhaoTom A. HummerJingdong LiuTom DeningJingjing TangTom Elijah VolenzoJingli LiuTom EmeryJinglong TangTom P. C. SchlosserJingping WangTom TreasureJingzhi SuTom VigilanteJinhan MoTom WalskiJinhwa JungTom WhyteJin-Hwa JungTomas G. VillaJinhyun HongTomás García CalvoJinkai XueTomas LazdauskasJinkyung Jenny KimTomas Rafael Bolaño OrtízJin-Li HuTomáš ŘiháčekJinsoo KimTomas SoukupJinyang ZhangTomas VegaJinyu XuTomáš VencúrikJiraporn LueangsakulthaiTomaso VairoJiren XuTomasz BlicharskiJiří AmbrosTomasz CudejkoJiří ČernýTomasz DudekJiří KantorTomasz GigolJiri PetrakTomasz GondekJiri PopelarTomasz GradalskiJiří SuchýTomasz GrzywaczJiro OkumuraTomasz HanćJiseun LimTomasz JanoszekJisoo SimTomasz JedynakJi-Su KimTomasz KamińskiJisun LeeTomasz KijekJitendra SinghTomasz KlekielJitka MohelnikovaTomasz KonopkaJiun-Chi HuangTomasz KuligowskiJiun-Horng TsaiTomasz M. KarpińskiJiwnath GhimireTomasz MichalskiJiwon LeeTomasz MorońJi-Won RyuTomasz NitkiewiczJiyeon JungTomasz OszakoJiyong KimTomasz PorażkoJiyoung KimTomasz RechbergerJo Anna ShimekTomasz RogozińskiJo Britt-RankinTomasz RokickiJo KatoTomasz RywikJo ReyTomasz SipkoJo SourbronTomasz SobierajskiJoachim BachnerTomasz StompórJoachim KowalskiTomasz SzmudaJoachim KroisTomasz SzulJoachim MüllerTomasz SzymborskiJoachim SchnurbusTomasz TokarekJoachim VossTomasz UrbanowiczJoan AmerTomasz W. KaminskiJoan ConwayTomasz WolnyJoan Costa-FontTomasz ŻądłoJoan CunninghamTomasz ZalewskiJoan HarveyTomasz ZaprutkoJoan Ignasi Mestre-PintoTomasz ZdziebkoJoan L. KenneyTomasz ŻelazińskiJoan QuilesTomasz ZwęglińskiJoan VicianoTomaszewski PawełJoana Bessa TopaTomer MevorachJoana CostaTomi ZlatarJoana DuarteTomislav ĆabovJoana FernandesTomislav MalvicJoana Ferreira LealTomislav MestrovicJoana GasparTomislav RukavinaJoana M. O. SantosTomiyo NakamuraJoana Pereira De Carvalho FerreiraTommasina PianeseJoana PrataTommaso BellandiJoana Proença BeckerTommaso FilippiniJoana ReisTommaso LombardiJoana Sofia Dias Pereira De SousaTommaso ProiettiJoana T. CoutinhoTommaso VitaleJoan-Francesc Fondevila-GascónTommi InkinenJoAnn OliverTomohiko IsobeJoanna A. KamińskaTomohiro TabataJoanna BadachTomokazu OhishiJoanna BaranTomoki NakamizoJoanna BogusławskaTomoki SekiguchiJoanna Bohatko NaismithTomoko ItoJoanna BzówkaTomoko KodamaJoanna ChylińskaTomoko OmiyaJoanna CzarneckaTomoko TakamiyaJoanna DipnallTomoko UekitaJoanna FelczakTomomi HigashiJoanna FronczykTomonaga UenoJoanna GaitensTomoya ItataniJoanna GodlewskaTomoyuki KobayashiJoanna GórkaTomsa RalucaJoanna HildebrandTonghua WuJoanna JabłońskaToni AltermanJoanna Janiszewska-OlszowskaToni Torres-McGeheeJoanna JaroszewiczTonia De GiuseppeJoanna Jeż-WalkowiakTonia VassilakouJoanna KaczorowskaTonino TrainiJoanna KamińskaTonko KovacevićJoanna KobzaTony SkapetisJoanna KonopińskaTony WallJoanna KorzeniowskaTony WaterstonJoanna Kos-ŁabędowiczTooba BatoolJoanna KruszewskaToomas TimpkaJoanna KulskaTope OyeladeJoanna L. FiddlerTorbjörn LedinJoanna L. MusseyTord Finne VedøyJoanna LukasikTore BonsaksenJoanna ŁukasikTöres TheorellJoanna M. HaraznyTorgeir HellstrømJoanna MazurTorkild VisnesJoanna Myszkowska-RyciakTorsten MuellerJoanna Piepiórka-StepukToru HorinouchiJoanna Pietrzak-ZawadkaToscanelli CeciliaJoanna PocztaToshiaki NakanoJoanna PodlasińskaToshihiko TashimaJoanna Popiołek-KaliszToshihiro TakashimaJoanna RakowskaToshihisa DoiJoanna RogToshimasa SoneJoanna RógToshimune KambaraJoanna RoszakToshinobu TakedaJoanna Rutecka-GóraToshiyo TamuraJoanna RymaszewskaToshiyuki IwahoriJoanna Sadłowska-WrzesińskaToshiyuki KawaiJoanna SamulToshiyuki KuriharaJoanna Śliwa-DominiakTove WästerlidJoanna SłomkoTracey CockertonJoanna SmardzTraci HayesJoanna Smarkusz-ZarzeckaTraci HongJoanna Smoluk-SikorskaTracie AddyJoanna SorraTracy DonachieJoanna StragierowiczTracy PintchmanJoanna SuliburskaTracy ReibelJoanna SzydłoTran NguyenJoanna TrafiałekTrancă SebastianJoanna TrelinskaTravis YatesJoanna WachnickaTrevin GlasgowJoanna WitkosTriantafyllia G. NikolaouJoanna Wnęk-GozdekTricia PercivalJoanna WróblewskaTridib RajkhowaJoanna ZajdaTrinidad Montero-VilchezJoanna Zielińska-SzczepkowskaTrinidad Sentandreu-MañóJoanne BrookeTrisha DunningJoanne Difrancisco-DonoghueTrishna PatelJoanne G. PattersonTrishnee BhurosyJoanne GuthrieTristan CaseyJoanne HudsonTristan WagnerJoanne LeachTroy PurdomJoanne LloydTrudie F. MilnerJoanne NewburyTrudy NormanJoanne PikeTruls GjestlandJoanne ThandrayenTrung NguyenJoanne VincentenTrung Thanh L. NguyenJoanne YipTrung TranJoannes ChliaoutakisTsang-Chuan ChangJoão Antônio Leal De MirandaTsang-Hsiang ChengJoão Batista Ferreira‐JuniorTsang-Kai HungJoão BotelhoTse-Lun ChenJoão Carlos MacedoTser-Yieth ChenJoão Cavaleiro RufoTshepo RasekabaJoão ClaudinoTsige-Yohannes HabteJoão Eduardo Marques-TeixeiraTsubasa KawasakiJoão F. C. A. MeyerTsu-Ming YehJoão Filipe Fernandes Lindo SimõesTsung-Cheng HsiehJoão Flávio Da Silveira PetruciTsung-Hsien LeeJoão Gaspar-MarquesTsung-Hsueh LuJoão GregórioTsung-Po ChenJoao GuerreiroTsung-Yu ChouJoão GuimarãesTsutomu OmatsuJoão HenriquesTsuyoshi KawakamiJoão LeitãoTsuyoshi OgataJoão Luís FernandesTsvetelina PetrovaJoão M. LopesTsvetelina VelikovaJoão M. SantosTsvetoslav GeorgievJoão MaltaTsz Wun TsangJoão Manuel Da Silva Carvalho CarvalhoTuan T. NguyenJoão Manuel Garcia Do Nascimento GravetoTuck Seng ChengJoão Manuel Graça FradeTucker BurchJoão MartinsTudor DeaconescuJoão MesquitaTuija MuhonenJoão P. L. DaherTuki AttuquayefioJoão Paulo Assis GoboTulsi PaudelJoão Paulo BritoTünde TóthJoão Paulo LealTung-Hsun HsiehJoão Paulo Mendes TribstTung-Ju WuJoão Pedro Costa-NunesTurcu-Stiolica AdinaJoão PetricaTuyen Van DuongJoão PiedadeTwylla KirchenJoão PinheiroTyagi RamakrishnanJoao Ramalho-SantosTyler J. KybartasJoao RochaTyrone PretoriusJoão Rocha VazTyrone T. LinJoão Santos BaptistaTze-Ern ChuaJoão SerranoTze-Huan LeiJoão Silva SequeiraTzeng-Ji ChenJoão Silvestre Silva JuniorTzeta HuangJoão TavaresTzong-Ru LeeJoão VasconcelosTzu Hsuan ChiangJoão Victor RochaTzuchi LeeJoão-Paulo-Mendes TribstTzu-Chieh ChouJoaquim C. G. Esteves Da SilvaTzu-Ching ShihJoaquim Calvo-LermaTzu-Chueh WangJoaquim CerejeiraTzu-Ling ChenJoaquim RossiniTzu–Yi PaiJoaquín Guillermo Ruiz PinillaTzu-Yu PengJoaquín NavajasTzu-Yuan ChaoJoaquin Paez-MoguerUchendu Eugene ChigbuJoaquín-Lorenzo BurgueraUchita VaidJob F. HarenbergUddyalok BanerjeeJobst WurlUdi LebelJocelyn JonesUdo SeedorfJochen D. SchipkeUgo CarraroJochen TöpferUgo ConsoloJochen WilhelmUgo CovaniJodi HedgesUgo FaragunaJodi StreichUgo IndraccoloJody DushayUirassu BorgesJoe JamesUjwalkumar PatilJoel AikUlderico FreoJoel BurleyUlf IsakssonJoel Ferreira Santiago JuniorUlf KallweitJoel JamesUlf L. LundbergJöel LadnerUlf MohrlokJoel MartinUlf PetterssonJoel MethorstUlf SchöttJoel N. MaslowUlises De La Cruz-MossoJoel NitzkinUloma UcheJoel R. MalinUlrika TranaeusJoel SussUlrike BuhlmannJoe-Ming LeeUlrike H. MitchellJoerg SchweizerUlrike ZitzJoeri Jan PenUma S. BhattJohan HultdinUmar BurkiJohan MolenbroekUmar HayatJohann ZwirnerUmberto GaragiolaJohanna BlakeUmberto M. MusazziJohanna FitzgeraldUmberto SolimeneJohanna FraserUmer ZamanJohanna PerssonUmesha BoregowdaJohanne HébertUmut CaginJohannes LützenkirchenUna BrittonJohannes M. LützUna MasicJohannes MüllerUnai AzcarateJohannes OrlowskiUndine LehmannJohannes PernaaUpasak DasJohannes SchobelUpen PatelJohannes SchulzeUrban BrenJohannes StadlmannUri ObolskiJohannes StraussUrska CvekJohannes Van Der KwastUrska DobersekJohannes Van DijkUrsula Ackermann-LiebrichJohannes WancataUrsula MartinezJohannes WendscheUrsula VoßJohannes ZenkUrszula CytlakJohannes ZipperleUrszula JankiewiczJohn A. CaldwellUrszula KobylinskaJohn A. MaluccioUrszula KotowskaJohn A. RathmacherUrszula LisieckaJohn A. St.CyrUrszula Markowska-ManistaJohn A. WoodsUrszula SomorowskaJohn AhnUrvish PatelJohn AllanUsama AwanJohn Amson CapitmanUsama El-AwadJohn BabrajUtako SawadaJohn BaldwinUttara RoyJohn BarentineUwe MüllerJohn BartkowskiUzun Dzhamal RedzhepJohn BartlettVaclav BuncJohn BerketaVadim YapiyevJohn BernertVahan KépénékianJohn BischofVahid NasirJohn BrydenVahid RakhshanJohn BuckleyVaida SteponavičienėJohn BuxceyVaios PeritogiannisJohn C. BarentineVaishnavi RatheeshJohn CherrieVaithinathan SelvarajuJohn CorreaVaitsa GiannouliJohn CoulterVal WongsomboonJohn D. PerryVálber César Cavalcanti RozaJohn DadzieValda RondelliJohn DurocherValdemar E. Arce GuevaraJohn Edward ThornesValder Cordeiro Barbosa FilhoJohn F. NewmanValentin BenzingJohn F. T. FernandesValentín Emilio Fernández ElíasJohn FischettiValentin Marian AntohiJohn FisherValentín Molina-MorenoJohn FlackValentin Nicolae VarlasJohn GalgianiValentina AlfonsiJohn GalvinValentina BaccoliniJohn GuentherValentina BessiJohn GunnValentina BrombinJohn H. FosterValentina Brzović RajicJohn InnesValentina BulloJohn J. GuersValentina ButvinaJohn Jairo AraujoValentina CesariJohn Jairo Villarejo MayorValentina ChkoniyaJohn (Jay) K. BainbridgeValentina ColonnelloJohn K. StanleyValentina Di DonnaJohn K. YueValentina Di FeliceJohn KratusValentina DolceJohn LambertValentina FavalliJohn M. PolimeniValentina GentiliJohn McLuskeyValentina GinevičienėJohn McNamaraValentina GiudiceJohn MolitorValentina Lucena JuradoJohn MondanaroValentina NdouJohn N. HutchinsonValentina Oana BudaJohn O’HaganValentina PiccoliJohn OngValentina RizzoliJohn OswaldValentina Rosa LaganàJohn P. LubickyValentina RovelliJohn Patrik BurkhardValentina RovielloJohn PearnValentina SpensieriJohn ProchaskaValentina TomatJohn PufferValentina VasileJohn R. BelcherValeria BellisarioJohn R. GilstadValeria Cesario TimesJohn RamseyValeria D’ArgenioJohn ReadValeria M. NurchiJohn RolloValeria MonnoJohn SieverdesValeria SaladinoJohn SnawderValeria VerrastroJohn StudleyValérian CeceJohn W. Farrell IIIValeriana GuijoJohn W. NicholsonValerie BauzaJohn W. YuenValerie BolivarJohn WainwrightValerie CainesJohn WeinsteinValerie DeMarinisJohn WildmanValerie InghamJohn ZarobellValerie MinarchickJohnannes Jacob De SoetValerie T. ChangJohnny ChanValerio CicconeJohnny PaduloValerio Dell’osteJohnny ReisValerio GallottaJohnson LokValerio LeoniJohny VerschakelenValério Monteiro-NetoJoJo WongValerio PaoliniJolanta BanasiewiczValerio RicciJolanta DąbrowskaValerio VolianiJolanta E. KowalskaValeriu Marin ȘurlinJolanta E. LosterValeriya FilipovaJolanta KarpowiczValery KuznetsovJolanta KisielewskaValmore BermúdezJolanta Klukowska-RötzlerVan Khanh NguyenJolanta Kostrzewa-JanickaVanaja KankarlaJolanta KrólVanaja Krishna NaikJolanta MackowiczVance Girard NielsenJolanta Nawrocka-RutkowskaVanda AndradeJolanta PaukVanda Maria AndradeJolanta WeaverVanesa AbuinJoLee SasakamooseVanesa Cantón-HabasJolita VveinhardtVanesa GonzálezJomar RabajanteVanessa AzevedoJon GrudaVanessa ClarkJon IrazustaVanessa Lani Zarya Gordon-DseaguJon OdlandVanessa Resende Nogueira CruvinelJon ReiersenVanessa SimondsJon Salmanton-GarcíaVanessa ZorrillaJonas KolbenschlagVangelis SakkalisJonatan García CamposVânia CostaJonathan BroadbentVânia Marilande CeccattoJonathan CaudillVânia RochaJonathan CharestVanilson LemesJonathan D. KarpVanish KumarJonathan D. NeukamVanner BoereJonathan DarbroVarpu I. S. JokimaaJonathan E. MooreVasco André Barbosa BrandãoJonathan FouldsVasco BrancoJonathan Frank AntinVasco Ribeiro SantosJonathan FreedmanVasile GherhesJonathan FusiVasile GramaJonathan HobsonVasile HateganJonathan LecureuxVasile Paul BresfeleanJonathan MaupinVasileios I. DiamantisJonathan Ospina BetancurtVasileios PapapanagiotouJonathan PatzVasiliki AssimakopoulosJonathan QuinlanVasiliki KinigopoulouJonathan SametVasiliki LeventakouJonathan SchaeferVasiliki SakellariJonathan TritterVasiliki VranaJonathon P. LeiderVasiliki ZisiJong Cheol ShinVasily SukhorukovJong In KimVass Reka AnnaJong Kook SongVassilios PanoutsakopoulosJongbae KimVasudev HaribalJongBeom LimVaughan ReesJongchul ParkVedran HadžićJonghun (jay) ParkVedran RubinićJong-Hwa JangVeerasamy YengopalJonghyun OhVelasco CimicaJonghyun ShinVelga SudrabaJonghyun YunVeljko ProdanovicJongki ChoVelma LopezJong-Ki HuhVelmurugan BalaramanJong-Min KimVen Paolo Bruno ValenzuelaJongmin LeeVendela ZetterqvistJong-Sang YounVenera GashajJongseong GwakVenera TomaselliJongsik YuVenerando Antonio RapisardaJonh Hun WooVenerando RapisardaJonhatan SilvaVenkat HariharanJonida HaxhiVenkateshwari VaradharajanJonivar SkullerudVenkateswara Reddy GogulamudiJonna WilénVenkitasamy BaskarJoo Young KimVenky ShankarJoon Kyoung KimVentura Pérez-MiraJoon Kyu LeeVera A. KrischikJoon W. ShimVera A. YakupovaJoon Young KimVera AlmeidaJoon Young LeeVera AmicarelliJoonho KoVera BrenčičJoon-Kee YoonVera L. SnezhkoJoonyoung LeeVěra OlišarováJoost BuysschaertVera SchwierzeckJoost Van Der VorstVera StaraJoost VerhoevenVered HolzmannJooyoung KimVerena AndreattaJoo-Young OheVerena EllerkampJordan M. SangVerena MenzJordi Arboix-AlióVerity TrueloveJordi Colomer FeliuVerity WainwrightJordi Franch-ParellaVern HarnerJordi GumàVerna B. McKennaJordi MogasVernon HodgeJordi NinVernon M. ChinchilliJordi PortaVeroni I. EichelsheimJördis WothgeVeronica Alonso-FerreiraJordyn Tinka WallenbornVeronica Andrea Gonzalez-LopezJörg H. StehleVeronica AranJörg KleeffVerónica Barroso-GarcíaJorge Amate-RomeraVeronica CimolinJorge BarracaVeronica De SimoneJorge Calvillo-ArbizuVeronica Elena TrombitasJorge Cancino-LópezVerônica GinaniJorge Carlos-VivasVerónica Luque-AgudoJorge Casaña-MohedoVerónica Marín-DíazJorge Cerqueiro-PequeñoVeronica MatthewsJorge E. LuzuriagaVeronica MuffatoJorge Esquiche LéonVeronica Perez-CabezasJorge Expósito LópezVeronica Perez-De La CruzJorge Fernández Del ValleVeronica SalettiJorge Fernandez HerreroVeronica SchiaritiJorge FerreiraVeronica SvärdJorge Gallardo-CamachoVeronica VelascoJorge García-UnanueVerónica Velasco-GonzalezJorge García-VillanuevaVeronica VernonJorge GonçalvesVerónica Violant-HolzJorge Góngora-RodríguezVeronika AleksandrovychJorge GuillénVeronika CapJorge Hugo VillafañeVeronika GinzburgJorge Humberto Palmeira RamosVeronika PisarenkoJorge Ivan RamírezVeronique BillatJorge Joo NagataVeronique Deschodt-ArsacJorge LizandraVéronique Louise BillatJorge LopezVeruscka LesoJorge López-FernándezVesa Cosmin MihaiJorge Luis CandiottiVesa KuikkaJorge Luis García-AlcarazVesna JacevicJorge Luis Méndez UlrichVesna JaksicJorge M. MendesVesna Vukašinovic-PešicJorge Marcos MarcosVesta SteiblieneJorge MirandaVetrano MarioJorge Moreno-FernandezViacheslav OsadchyiJorge Moya HiguerasVianney SivelleJorge NegreirosVibeke Elise AnsteinssonJorge O. López-MartínezVicenç LeónJorge OliveiraVicente Alcaraz Carrillo De AlbornozJorge PereiraVicente ClimentJorge Pérez CorralesVicente Gea-CaballeroJorge Pérez-GómezVicente Lopez-ChaoJorge R. ReyVicente MartinezJorge RevezVicente Miñana-SignesJorge RochaVicente Morales BañosJorge Rodríguez-MenésVicente Peñarroja-CabañeroJorge Rojo RamosVicente Romo PérezJorge SalasVicki G. MokuriaJorge Salas HerranzVicki HuttonJorge SobralVicki MercerJorge T. RibeiroVicki MokuriaJorge UmbelinoVicki MyersJorge Velázquez SaornilVicky ChangJorge X. Velasco-HernandezVicky WaltersJorge-Manuel DueñasVíctor Arufe-GiráldezJørgen Trankjær LauridsenVictor CheuyJoris J. Van HoofVictor E. A. GerdesJoris Van LoenhoutVictor Fernandez NascimentoJoris Van OuytselVictor GarciaJorma VirtanenVíctor García-GonzálezJor-Man HuangVíctor Gómez‐MayordomoJörn TheuerkaufVictor Gonzalez CalatayudJorunn EvandtVictor H. BenitezJos A. Van Der HageVictor Jiménez Díaz-BenitoJos TwiskVíctor José Villanueva-BlascoJose A. BragadaVictor M. González-ChordáJosé A. Cano BuendíaVictor M. JimenezJosé A. CernudaVictor Manuel Gonzalez-ChordaJose A. MartinezVictor Manuel MeseguerJosé A. Pérez-MéndezVictor MathesonJosé A. PiquerasVictor MinichielloJosé Afonso NevesVictor NorrefeldtJosé Alberto García-BernáVictor Ortiz-MallasenJosé Alberto Herrera-MeliánVíctor RincónJosé Alberto Martínez-GonzálezVictor Ruiz-GarcíaJosé Alberto Ribeiro-GonçalvesVictor ShevchukJosé Albeto Laredo-AguileraVictor TiberiusJose Alfredo HernándezVictor W. PerezJosé AlvarelhãoVictor ZaiaJose Andres Lorca MarínVictor Zuniga DouradoJosé Ángel Martínez-LópezVictoria BanyardJose Ángel Rodríguez-GómezVictoria BelousovaJosé António Ferreira PorfírioVictoria BrownJosé Antonio González SánchezVictoria G. RontoyanniJose Antonio Gonzalez-JuradoVictoria HidalgoJose Antonio Hurtado-SánchezVictoria IñigoJose Antonio Llosa FernándezVictoria L. BoggianoJose Antonio Lopez-EscamezVictoria LeclercqJosé Antonio MingoranceVictoria M. Bajo LorenzanaJosé Antonio Morales-GonzálezVictoria MacBeanJose Antonio OrtegaVictoria Mazoteras-PardoJosé Antonio Peña-RamosVictoria MeahJosé Antonio Ponce-BlandónVictoria Ramos GonzálezJosé Antonio RodríguezVictoria Soto-SanzJosé Antonio Ruiz-HernándezVictoria Valeriivna RodinkovaJosé Antonio SantosVida DemarinJose Antonio Vazquez-LopezVidar AndersenJose AscheriVidas ŽuraulisJosé Augusto SimõesViera ŠvihrováJose Aurélio FariaViet-Khoa Tran-NguyenJosé Benavente HerreraVigouroux NadineJosé Benito Bouza-RodríguezVijay IndukuriJose BetancourtVijay Kumar SharmaJosé Blanco SalasVijayalaxmi G. GuptaJosé Carlos Dias RoucoViji VijayanJosé Carlos LazaroVikas GuptaJosé Carlos Núñez PérezVikas KumarJosé Carlos RochaVikas SaxenaJosé Carlos RodríguezVikas SharmaJosé Carlos SáVikas TaankJosé Carlos Vázquez-ParraVikash AgrawalJosé Carmelo AdsuarVikki HouldenJose Carpio PinedoVikkie MustadJose Ceron-CarrascoVikram SunkaraJose CornejoVikramjit S. BajwaJosé D. UrchagaVikrant RaiJosé Daniel Jiménez-GarcíaViktor FredrichJose De Albuquerque Calasans-MaiaViktor SebestyénJose De Jesus Agustin Flores CuautleViktor TrynchukJose De Jesus RubioViktoria WahlströmJosé De Jesús Sandoval-PalomaresViktoriia VovkJosé DevísVilena KašubaJosé DóreaVilija Bite FominieneJosé Egídio Barbosa OliveiraVilija MalinauskieneJosé Enrique Moral-GarcíaVilla SimoneJosé Enrique Torres VaamondeVilma KaškonienėJosé F. Gómez-ClavelVilma XhakollariJosé Fabián Reyes RománVinay PitchikaJosé Francisco López-GilVinay ShuklaJosé Francisco Oliveira-JúniorVince ÖrdögJosé Francisco Rangel PreciadoVincent C. W. ChenJosé Francisco Rodríguez-VázquezVincent CastelainJosé Francisco Segura PlazaVincent ChuaJose Francisco Tornero-AguileraVincent DelormeJosé GamonalesVincent GroteJosé García MorosVincent Joseph HargadenJosé Garcia-ArroyoVincent Konadu TawiahJose Geraldo MillVincent NijmanJosé Gibergans-BáguenaVincent P. DiegoJosé Gómez GalánVincent PonsJose Granero-MolinaVincent Van BuulJosé Gutiérrez-PérezVincent WildingJose H. Ablanedo RosasVincente Javier Clemente-SuarezJosé Henrique PilottoVincenza CofiniJosé Hernández OrtegaVincenza GianfrediJose Ibeas-LopezVincenzo CondemiJosé Ignacio Baile AyensaVincenzo Cristian FrancavillaJosé Ignacio Calvo ArenillasVincenzo Di LeoJosé Ignacio Nazif-MunozVincenzo GallelliJosé J. Castro-TorresVincenzo MarcotrigianoJosé Jair Alves Mendes JuniorVincenzo Nicola TalesaJosé Javier Navarro PérezVincenzo NuzziJosé Javier Reyes-LagosVincenzo RandazzoJosé Javier Romero - Díaz De La GuardiaVincenzo SavarinoJosé Javier Serrano LaraVincenzo ViscoJosé Javier Zamorano-LeonVincenzo ZanonJosé Juan Cañas DelgadoVineet ThomasJosé Juan Carrión-MartínezVinko PaulićJosé L. Amaro-MelladoVinny NegiJose L. Garcia-SerranoVinod KadamJose L. García-SoidánVinodh KakkasseryJose L. Gómez-UrquizaViola Camilla ScoffoneJosé Laerte BoechatViolante MartínezJosé Leandro TristánVioleta AlarcãoJosé Lino CostaVioleta Clement-CarbonellJosé Luís AbrantesVioleta EneaJosé Luis Cueto AncelaVioleta MugicaJosé Luis FelipeViorel MihailaJosé Luis González-CastroViorela-Ligia VaideanJosé Luis LaluezaViren SwamiJosé Luis Martín-ContyVirgilio López-MoralesJose Luis MirallesVirginia AguayoJosé Luis OrtegaVirginia Carbajosa RodríguezJosé Luis Roca-GonzálezVirginia MarshallJosé Luis Rodríguez GutiérrezVirginia Martínez-RuizJosé Luis SanzVirginia Navajas-RomeroJosé Luis Sarasola Sánchez-SerranoVirginia Puyana RomeroJose Luis UbagoVirginia Ramseyer WinterJosé Luis Yagüe-BlancoVirginia RecchiaJose M. Férnandez-BataneroVirginia Sánchez-JiménezJose M. Jimenez-OlmedoVirginia W. MaclarenJose M. MestreVirginie RappeneauJosé M. RamírezViriya HantrakunJosé M. Rodríguez-FerrerVirtudes Pérez-JoverJosé Mª Cancela CarralVirve MariounneauJose Manuel Diaz-SarachagaVisakh VaikuntanathanJose Manuel García GarcíaVisalini Nair-ShallikerJosé Manuel GuaitaVishal KhairnarJosé Manuel Jaramillo OrtizVishal M. KothariJosé Manuel MendesVishalkumar G. ShelatJosé Manuel Moreno VillaresVishnu Priya MuraliJosé Manuel Muñoz RodríguezVisitación López-MirandaJosé Manuel NaranjoVisnja LezaicJosé Manuel Otero-LópezVita LesauskaitėJosé Manuel Saiz-ÁlvarezVita PostuvanJose Manuel Santos-JaenVitalii LunovJosé Manuel TomásVitaly PostoevJosé María Celaya PadillaVito GiganteJosé María Izquierdo VelascoVito M. R. MuggeoJose Maria Leon-PerezVito RizziJose María León-RubioVítor GonçalvesJosé María León-RubioVítor João Pereira Domingues MartinhoJose Maria Pereira De GodoyVítor LopesJosé Max Barbosa De Oliveira-JúniorVitor Nazário CoelhoJosé MendesVítor RaposoJosé Miguel Mansilla DomínguezVitor RoqueJosé Miguel Martínez-SanzVittorio ChecchiJosé Miguel Robles RomeroVittorio GebbiaJosé Miguel Seguí-RipollVittorio LocatelliJosé Miguel Sequí-CanetVittorio MaglioneJose N. Sancho-ChustVittorio SarcheseJose Oñate-GarzónVivek DharwalJosé Oriol Rios GonzalezVivek PrasadJose Parra-MartinezVivek Vijay ThackerJosé Pedraza ChaverriViveka Lyberg ÅhlanderJosé Pedro MartinhoVivian De WaardJosé Pérez-AlonsoVivian RamsdenJosé Pinto CasquilhoVivian VimarlundJose PreciosoViviana AndreescuJose R. AlamedaViviana Toro-IbacacheJose R. CordonViviani GomesJosé R. JiménezVivienne M. HazzardJosé Ramón Calvo-FerrerVjekoslav CigrovskiJosé Ramón CardonaVlad BurtaverdJosé Ramón-CardonaVladimir BerikovJose’ Ramos Pires MansoVladimír BurešJose RodriguezVladimir CvetkovićJosé Ruas-AraujoVladimir FilipovicJosé SantosVladimir PorochJose Sebastián Velázquez BlázquezVladimír RogalewiczJosé SilvaVladimir StevanovićJosé SuazoVladimir Tale TakšićJosé Tomás Mateos GarcíaVladislav SvozilíkJose Torres-PruñonosaVlaho BrailoJose Ueleres BragaVlasoula BekiariJosé V. PestanaVlasta BariJose V. TarazonaVlastimil ChytrýJosé ValeVo Truong Nhu NgocJose VaronaVoicu-Dan DragomirJosé VerdúVojtech IlkoJose Vicente Beltran-GarridoVolker BeckmannJose Vilaça-AlvesVolker HarthJosé YelaVolker Rudolf ZschorlichJose ZafraVrba JiriJosé-Antonio Giménez-CostaVui King Vincent-ChongJose-Benito Rosales ChavezVyacheslav DushenkovJosée LavoieVytis KopustinskasJosef BaumgartnerW. Jay ChristianJosef BotlikWadih MaaloufJosef CyrysWadim StrielkowskiJosef HalamekWafa DouziJosef KöhrleWai Phyo AungJosef KuncWai Sum LeeJosef MarousekWaldemar KociubaJosef NavrátilWaldemar SzaferskiJosef SejákWalery ŻukowJosef TröglWalid Kamal AbdelbassetJosé-Jesús Jimenez-RejanoWalid MarrouchJose-Luis Montes-BotellaWalid OueslatiJosé-Luis Rodríguez-SánchezWallace CasacaJose-Manuel Ramos-RinconWalter August LorenzJosé-María JiménezWalter BrehmJosé-María Sánchez-GonzálezWalter BusuttilJosé-Miguel Martinez-CarriónWalter CurrentiJosep EsplugaWalter DachagaJosep GallifaWalter DukićJosep Lladós MasllorensWalter GoesslerJosep Maria Ramon TorrellWalter HewerJosep Maria Ramon-MuñozWalter R. SchummJosep Maria UstrellWalter SchummJosep PetchaméWalter Y. H. LamJosep SolvesWan ShenJosep Vidal ContiWanda MączkaJoseph AttiasWanderlei Abadio De OliveiraJoseph BaumgardenWang-Chin TsaiJoseph Calvin GagnonWangdo KimJoseph Calvin KouokamWang-Kin ChiuJoseph D. LaydenWangshu MuJoseph E. UscinskiWan-Jiun ChenJoseph GuydishWann Yih WuJoseph James ValadezWarren DoddJoseph KochmanskiWasantha P. JayawardeneJoseph L. ServadioWasyl BilczakJoseph MarinoWayne RobargeJoseph OkemeWei ChenJoseph PascarellaWei DuJoseph SsenyongaWei GaoJoseph T. SteensmaWei GuJoseph TelfairWei HsuJoseph Thomas OrtegaWei LinJoseph WagmanWei LiuJosephine AlbaWei NiJosephine BlagraveWei SongJosephine HartwigWei WeiJosephine StorekWei YangJosep-Maria LosillaWei ZhangJosé-Víctor RodríguezWei-Biao LiaoJoshua A. LawsonWeibo LiuJoshua EdwardsWei-Chun LinJoshua HallWeihua DingJoshua J. GannWeijie WangJoshua JoshuaWei-Ju ChangJoshua L. SantarpiaWeilan WangJoshua LambertWeili DuanJoshua Nosa EdokpayiWeilun SunJoshua P. BaileyWeining WuJoshua Porat-DahlerbruchWeiran ShanJoshua R. HuotWei-Rong LinJosie BoothWeisheng ChiuJosie DickersonWei-Ting HsuJosipa BukicWeiwei LinJoske NautaWeiyan YinJoško ŠodaWeiyi MaJosu Solabarrieta EizaguirreWei-Ying SungJosy AugustineWeizhen DongJoung Yoon ChoiWellington AlvesJovana TodorovicWen ChenJoy FurnivalWen Han TongJoy HoneaWen ZhangJoy L. HartWenbiao HuJoy ParkinsonWen-Cheng LinJoy PerrierWen-Cheng LiuJoyce ArdittiWen-Chien LuJoyce Jpa BierboomsWendi WeimarJoyce OlusholaWendy BonythonJoyce ShirindeWendy LeeJože GunaWendy Lynne GilleardJozef KobzaWendy WuytsJózef OberWenfang LuJozef SimenkoWeng Marc LimJozef SowinskiWenhui JiangJózsef PiffkóWenjuan ChengJózsef Z. FarkasWenping LiuJr-Rung LinWensheng WangJu Hee KimWen-Tzu WuJu WangWesley KephartJuah KimWidya LestariJuan A. Fafián-LaboraWieslaw UrbanJuan A. Marin-GarciaWietske DohmenJuan A. Ortega-GarcíaWiktor JedrzejczakJuan Agustín Morón-MarchenaWilhelm BlochJuan AlberolaWillem PiebengaJuan Alfredo Jiménez-EguizabalWilliam JankowiakJuan Algar-PinillaWilliam NketsiaJuan Antonio Díaz-ManchaWilliam TigbeJuan Antonio López NúñezWilliam W. StringerJuan Antonio MorianaWillibald RuchJuan Antonio PascualWillie TanJuan Antonio Roche CárcelWilson DanielJuan Antonio Sánchez SáezWinfried SteilingJuan Antonio Valera-CaleroWing Hong ChuiJuan Ardoy-CuadrosWing Shan ChoiJuan AyalaWinifred E. NewmanJuan Bautista De SanctisWinnie Gerbens-LeenesJuan Bautista HerreroWłodzimierz KasprzakJuan C. De Agustin-AsensioWojciech BalJuan C. Meléndez MoralWojciech CichoszJuan CalmaestraWojciech DąbrowskiJuan Carlos Alvarez AlvarezWojciech EliaszJuan Carlos De La Cruz CamposWojciech GlinkowskiJuan Carlos Fernández-DomínguezWojciech J. CynarskiJuan Carlos Hernández-GómezWojciech KochJuan Carlos Marzo-CamposWojciech KoziolJuan Carlos Meléndez RodríguezWolf RogowskiJuan Carlos Peña AxtWolfgang EllermeierJuan Carlos Pomares TorresWolfgang SchröerJuan Carlos Redondo-CastánWolkersdorfer ChristianJuan Carlos Sánchez-GarcíaWon Seok LeeJuan Carlos SierraWon Sop ShinJuan ChenWon Suk ChoiJuan De Dios Benítez SilleroWon Won ChangJuan Del CosoWon-Sop ShinJuan Diego Ramos-PichardoWonsuk AnJuan E. Trinidad SegoviaWoo-Jin KimJuan Enrique Gonzálvez-VallésWoojoo LeeJuan Evaristo Callejas JerónimoWook SongJuan F. BlancoWouter MeijersJuan Francisco ColomaWui-Chiang LeeJuan Francisco Rodríguez-TestalWullianallur RaghupathiJuan Francisco Sánchez-SolerWycislik LukaszJuan Francisco Velarde-GarciaXerxes Tesoro SeposoJuan Garduño-EspinosaXiangpeng LiaoJuan GavalaXiao LuoJuan GómezXiaodong GeJuan Gómez SalgadoXiaofei LiJuan González HernándezXiaoge HanJuan González-MartínezXiaohu HuangJuan González-PascualXiaohui HanJuan J. CabréXiaojie TianJuan Jesús García-IglesiasXiaojun DengJuan Jose AcevedoXiaojun (Gene) ShanJuan José Criado-AlvarezXiaojun LuJuan José Galán DíazXiaolu GaoJuan José González-BadilloXiaomin WanJuan Luis Gonzalez-CaballeroXiaorui TianJuan Luis Hernández-ArellanoXiaoyan ChenJuan Luis Ramos-SuarezXiaoyan HuangJuan Luis Rodríguez HermosaXiaoyun LiJuan Luis Sánchez GonzálezXin CuiJuan M. García-CeberinoXin DengJuan M. MachimbarrenaXingguo ChengJuan M. SantosXinhai LiJuan M. Trujillo-TorresXinjian LiangJuan Manuel AlcántaraXinping GuanJuan Manuel Carmona-TorresXin-Tang LinJuan Manuel Martin AlvarezXinwen BaiJuan Manuel MendiveXize WangJuan Manuel Muñoz GonzálezXóchitl TrujilloJuan Manuel Muriel-SánchezXosé Somoza-MedinaJuan Manuel NuñezXu YanJuan Manuel PericasXue YangJuan Maza-SolanoXuejie DengJuan Mielgo-AyusoYachao LiJuan Miguel Barros-DiosYahui GaoJuan Miguel Fernandez CampoyYaisel J. BorrellJuan Miguel Martínez-GalianoYajuan SuJuan Milán-GarcíaYamakawa MiyaeJuan Mozas-MorenoYan GuoJuan MunizYang ChengJuan Pablo GutiérrezYang GaoJuan Pablo PizarroYang Woon ChungJuan Pedro Martínez RamónYan-Hong CuiJuan Puig-BarberàYanjie JiJuan Rodríguez MansillaYanjun RenJuan Sebastián Fernández-PradosYanping BaoJuan TorviscoYanyu SongJuan Vega-EscañoYan-Yuen PoonJuan ZaballosYaser AwadJuana Arias TrujilloYasinta Astin SokangJuana Inés Gallego-GómezYasuaki KakinokiJuana Maria Morillas RuizYasufumi ShigeyoshiJuana María Vázquez LaraYasuhiro MikiJuan-Francisco Álvarez-HerreroYasushi ObaseJuarez Bento Da SilvaYasutaka UmayaharaJucu Ioan SebastianYasuto SatoJudi MesmanYasuyuki GotoJudit SulyokYawen WangJudith A. BetoYayoi KobayashiJudith BoppYa-Yun ChengJudith D. StrawbridgeYe Hoon LeeJudith FuchsYe LiuJudith MassonYen-Chien LeeJudith RogersYen-Ming HuangJudith SligoYen-Tze LiuJudith StröhleYeong Jun JuJudith T. ZelikoffYeon-Ha KIMJudy OuYeon-Jee YooJudyta JuranekYeonsoo KimJue HouYezaz GhouriJue LiuYftach GepnerJue ZhangYi BuJuei-Tang ChengYi Hwa ChoiJuh Hyun ShinYi LiuJuhan LeeYi QiJuhani H. MäättäYi ShenJuhee LeeYi SongJuheon LeeYi YangJu-Hyeon SeongYi-Chang ChenJui-Hsiang LeeYi-Chiung HsuJulia AjejasYi-Chun ChenJulia BrodyYi-Chung ChenJulia DinaYi-Han LuJulia EnglertYiin Lih-MingJulia FussellYijun PanJulia Guerrero-GironésYi-Ming ChenJulia KönigYin Kiu LeungJulia M. MalyshYin Ping WongJulia Montserrat CodorniuYing LiJulia MurphyYing MaoJulia PhilippYing Tung ChanJulia PotterYing-Chun JhengJulia RiddellYingjie LiuJulia Wojciechowska-SolisYingleong Rigel ChanJulian C. L. LaiYingqi GuoJulian Kleine-BorgmannYing-Si LaiJulian Lindsay BurtonYino WuJulian LittleYirui LiangJulian Nevado BlancoYi-Ting LinJulián Solís García Del PozoYi-Wen ChiuJuliana Aparecida AnochiYixiang TianJuliana DushanovaYohei IkezumiJuliana F. Filgueiras MeirelesYohei SekinoJuliana Maria Faccioli SicchieriYoichi IkedaJuliana SáYoichi SakuradaJuliana Schaia RochaYoichi WatanabeJuliane HeydenreichYoichiro OginoJulianne L. PriceYoko SaitoJuliano Dal PupoYolanda Viridiana Chávez-FloresJulie A. SuhrYolande T. R. ProrogaJulie Aitken SchermerYong Il HwangJulie AyreYong Un BanJulie BeneshYongchuan TangJulie BennettYongheng GaoJulie BodinYong-Jun LinJulie BurrowsYongmi LeeJulie C. AbayomiYongming HanJulie C. LimaYongseok JeeJulie Dalgaard GuldagerYoo-Hyun UmJulie DumontYookyung BooJulie FutcherYoonhee HaJulie HenneganYoonsang KimJulie HernandezYoshihiro NoguchiJulie Laser-MairaYoshi-Ichiro KamijoJulie ParsonsYoshika SekineJulie PellerYoshiro HoraiJulie RageulYoul-Hun SeoungJulie SchweitzerYoung Ran KimJulie ShulmanYoung Sook RohJulie TownsendYoung UhJulie VaiopoulouYoung-Ki KimJulie VanderlindenYoung-Kwon SeoJulie WhitneyYoung-Min NohJulie WoodsYoungsook BaeJulien AndréaniYoun-Hee ChoiJulien MassonYounjae OhJulien S. BakerYu ChenJulieta Domínguez-SoberanesYu KumeJulio Augusto Freyre-GonzalezYu LiJúlio Barboza ChiquettoYu LiuJúlio Belo FernandesYu MengJulio Brito-SantanaYu Qi LeeJulio C. De La Torre-MonteroYuan WangJulio CostaYuan-Yuan WangJulio Díaz JiménezYu-Chao ChangJulio FernandezYu-Chi LeeJulio García-Del-JuncoYu-Ching ChouJulio López-AbánYu-Chu HuangJulio OchoaYuchun SunJulio Román Martínez-AlvaradoYueh-Hsia ChiuJulio Ruiz-PalmeroYufei CuiJulio-César De La Torre-MonteroYuichi MushimotoJulita RegulaYuichi SaitoJulius NukpezahYuji KabasawaJumin ParkYuji NagatomoJumpei SaitoYuji ShimizuJun Hee KimYuji TajiriJun LiYujiro KurodaJun MiyazakiYuka KotozakiJun NishikawaYukiko OkamiJun ShenYu-Kuei TengJun ShigemuraYulin ChenJun SuzurikawaYu-Lung ChiuJun WuYu-Min ChoJun Ying LiYun HanJun Yong ChoiYun WangJunaid Ur RehmanYunchun LiuJunayed PashaYung-An TsouJunbo GaoYung-Chieh LinJunbo WangYunjie LuoJuncai XuYunli BaiJune Andrews HorowitzYun-Seob LeeJung SunwooYun-Shiuan ChuangJung Tae KimYupeng LiJunga LeeYuri Leonidovich VasilievJung-Chih ChenYuri SadoiJung-Heun HaYuriy SlyvkaJunghwan KimYuriy SyerovJunghyun ChoiYushin KimJungkeun KimYusof KamisahJungsuk KangYusuke KatayamaJung-Woo LeeYutaka OwariJungyeon SungYuwaraj K. NarnawareJung-Youn ParkYuxiang HongJunhui JiYuxin LiJunichi ShodaYuzhong MaJunichiro YoshimotoYuzuru IsodaJunior Cesar AvanziZafer KeserJunjian GuZahra MojtahediJunjun YinZain Ul-AbdinJunki MaruyamaZandile MchizaJunkichi YokoyamaZargari Marandi RamtinJunko HondaZawiejska AgnieszkaJunming HuangZbigniew AdamskiJun-Pyo MyongZbigniew BasterJunqing TangZbysław DobrowolskiJunxiang ChenZdravko TrivicJunye JiangZeeshan Ahmad KhanJunyi HuaZe’ev PoratJuraj ChocholZengliang RuanJuraj GrencikZhanbing RenJuraj NemecZhaohui SuJurate LesutieneZhaomin LiuJure KnezZhe HanJure PražnikarZhen LiuJürgen GermannZhen WangJürgen PannekZhenbo WangJurgen SchleefZheng Zachory WeiJürgen SeitzZhengfei DaiJürgen StausbergZhenguang LiJurgis ZagorskasZhenhuan LiuJurgita Lazauskaite-ZabielskeZhi DouJuriena De VriesZhichao CaoJu-Seop KangZhihua TangJustin AllenZhipeng LiJustin ChapmanZhipeng LiuJustin DavisZhiqiang HuJustin E. HarbisonZhiqiang Kevin LuJustin MatusZhiwu WangJustin MoodyZhiyong HanJustin R. SempsrottZhiyu JiangJustin S. AntonyZhuoying WangJustin VarneyZiheng ShangguanJustin Xavier MooreZiqi LiJustine LeavyZixin WangJusto ArinesZiying LeiJustyna Berniak-WoźnyZoltán HeroldJustyna BrzezickaZoltán JákóiJustyna DunajZoltan LaknerJustyna DzięciołZoltan SimenfalviJustyna GodosZongxi QuJustyna Grudziąż-SękowskaZongye CaiJustyna KleszczZsofia LazarJustyna KozlowskaZunyao WangJustyna KujawskaZupeng ChenJustyna LeszczakZurab K. SilagadzeJustyna Liro


